# Supplementary material for: Selective synthesis of tightly- and loosely-twisted metallomacrocycle isomers towards precise control of helicity inversion motion
Source: Nat Commun. 2023 Dec 6;14:7868. doi: 10.1038/s41467-023-43658-5 (PMC10700359; doi:10.1038/s41467-023-43658-5)
Supplement: Supplementary file 1 — Supplementary Information [file 41467_2023_43658_MOESM1_ESM.pdf]

Supplementary Information

Selective synthesis of tightly- and loosely-twisted metallomacrocycle isomers towards precise control of helicity inversion motion

Tomoki Nakajima[1], Shohei Tashiro[1*], Masahiro Ehara[2] & Mitsuhiko Shionoya[1*]

[1]Department of Chemistry, Graduate School of Science, The University of Tokyo, 7-3-1 Hongo, Bunkyo-ku, Tokyo 113-0033, Japan. E-mail: tashiro@chem.s.u-tokyo.ac.jp, shionoya@chem.s.u-tokyo.ac.jp.
[2]Research Center for Computational Science, Institute for Molecular Science, Myodaiji, Okazaki, Aichi 444-8585, Japan.

1. Materials and methods ····3

2. Synthesis and characterisation of $\mathbf{1}_{\mathbf{tight}}$ and $\mathbf{1}_{\mathbf{loose}}$ ····4
2.1 Synthesis of [Pd(^{t}Bu$_2$bpy)(OH$_2$)$_2$](OTf)$_2$·(H$_2$O)$_2$ ····4
2.2 Synthesis of $\mathbf{1}_{\mathbf{tight}}$ ····6
2.3 Synthesis of $\mathbf{1}_{\mathbf{loose}}$ ····16
2.4 Definition of the two twisted isomers of **1** ····26
2.5 Characterisation of **2** ····27
2.6 Synthesis of [Pt(^{t}Bu$_2$bpy)(OH$_2$)$_2$](OTf)$_2$ ····30
2.7 Synthesis of [Pt$_2$**L**(^{t}Bu$_2$bpy)$_2$](OTf)$_4$ ····31

3. Isomerisation from $\mathbf{1}_{\mathbf{tight}}$ to $\mathbf{1}_{\mathbf{loose}}$ ····35
3.1 Theoretical analysis of the rate constant of isomerisation from $\mathbf{1}_{\mathbf{tight}}$ to $\mathbf{1}_{\mathbf{loose}}$ ····35
3.2 Time course ^{1}H NMR measurement to estimate the isomerisation rate from $\mathbf{1}_{\mathbf{tight}}$ to $\mathbf{1}_{\mathbf{loose}}$ ····36

4. Estimation of the rate of helicity inversion by EXSY NMR ····37
4.1 Theoretical analysis of the rate of helicity inversion based on ^{1}H-^{1}H EXSY NMR measurements ····37
4.2 Theoretical analysis of activation parameters of helicity inversion based on VT EXSY NMR measurements ····37

4.3 VT EXSY NMR measurements of $\mathbf{1}_{\mathbf{loose}}$ in acetone-d_6 and estimation of the rate and activation parameters of the helicity inversion ····38
4.4 EXSY NMR measurements of $\mathbf{1}_{\mathbf{tight}}$ ····42

5. Synthesis of chiral sulfoxides ····43
5.1 Synthesis of methanesulfinyl chloride ····43
5.2 Synthesis of 1,2,5,6-di-*O*-isopropylidene-α-D-glucofuranosyl (*S*)-methanesulfinate ····44
5.3 Synthesis of (*S*)-**3** ····47
5.4 Synthesis of 1,2,5,6-di-*O*-isopropylidene-α-D-glucofuranosyl (*R*)-methanesulfinate ····49
5.5 Synthesis of (*R*)-**3** ····51
5.6 Synthesis of (*R*)-methyl phenyl sulfoxide ····54
5.7 Synthesis of (*R*)-*p*-anisyl methyl sulfoxide ····57
5.8 Synthesis of (*R*)-*o*-anisyl methyl sulfoxide ····60
5.9 Synthesis of 2-propanesulfinyl chloride ····65
5.10 Synthesis of 1,2,5,6-di-*O*-isopropylidene-α-D-glucofuranosyl (*R*)-2-propylsulfinate ····66
5.11 Synthesis of (*R*)-isopropyl *p*-tolyl sulfoxide ····68

6. Asymmetric synthesis of $\mathbf{1}_{\mathbf{tight}}$ ····70
6.1 Synthesis of (*M*)-enantio-enriched $\mathbf{1}_{\mathbf{tight}}$ with (*S*)-**3** ····70
6.2 Synthesis of (*P*)-enantio-enriched $\mathbf{1}_{\mathbf{tight}}$ with (*R*)-**3** ····72
6.3 Summary of asymmetric synthesis of $\mathbf{1}_{\mathbf{tight}}$ ····74
6.4 Synthesis of $\mathbf{1}_{\mathbf{tight}}$ with (*R*)-methyl *p*-tolyl sulfoxide (2.2 equiv.) ····75
6.5 Synthesis of $\mathbf{1}_{\mathbf{tight}}$ with (*R*)-methyl *p*-tolyl sulfoxide (20 equiv.) ····76
6.6 Synthesis of $\mathbf{1}_{\mathbf{tight}}$ with (*R*)-methyl phenyl sulfoxide ····77
6.7 Synthesis of $\mathbf{1}_{\mathbf{tight}}$ with (*R*)-*p*-anisyl methyl sulfoxide ····78
6.8 Synthesis of $\mathbf{1}_{\mathbf{tight}}$ with (*R*)-*o*-anisyl methyl sulfoxide ····79

7. Evaluation of the helicity inversion rate of $\mathbf{1}_{\mathbf{tight}}$ ····80
7.1 Theoretical analysis of the rate constant of helicity inversion of $\mathbf{1}_{\mathbf{tight}}$ ····80
7.2 Time course ^{1}H NMR analysis to evaluate the racemisation rate of (*M*)-enantio-enriched $\mathbf{1}_{\mathbf{tight}}$ in acetone-d_6 ····82
7.3 Time course ^{1}H NMR analysis to evaluate the racemisation rate of (*M*)-enantio-enriched $\mathbf{1}_{\mathbf{tight}}$ in CD_2Cl_2 ····84
7.4 Time course ^{1}H NMR analysis of *rac*-$\mathbf{1}_{\mathbf{tight}}$ in CD_2Cl_2 with Δ-**4** ····85
7.5 Time course ^{1}H NMR analysis of (*M*)-enantio-enriched in CD_2Cl_2 with Δ-**4** ····86

8. Computational study ····89

9. References ····94

1. Materials and methods

Two metal sources, [Pd(^{t}Bu$_2$bpy)(OH_2)$_2$](OTf)$_2$·(H_2O)$_2$ and [Pt(^{t}Bu$_2$bpy)(OH_2)$_2$](OTf)$_2$, and sulfoxides, (*S*)- and (*R*)-**3**, (*R*)-methyl phenyl sulfoxide, (*R*)- and *rac*-*p*-anisyl methyl sulfoxide, and (*R*)- and *rac*-*o*-anisyl methyl sulfoxide were prepared according to reported procedures, and their analytical data were referred to the reported ones. Specific references for them are noted where applicable. Ligand **L** was prepared by following the reported procedure[1]. Other solvents, organic and inorganic reagents are commercially available, and were used without further purification.

NMR spectroscopic measurements were performed using a Bruker AVANCE 500 spectrometer (500 MHz for ^{1}H; 471 MHz for ^{19}F; 126 MHz for ^{13}C) and a JEOL JNM-ECX400 spectrometer (400 MHz for ^{1}H). Display of NMR spectra and deconvolution analysis were performed using an iNMR software. The chemical shifts were reported in parts per million (ppm) on the δ scale, and were referenced to tetramethylsilane (TMS, δ = 0 ppm for ^{1}H NMR) and $CDCl_3$ (δ = 77.16 ppm for ^{13}C NMR) in $CDCl_3$, acetone-d_5 (δ = 2.05 ppm for ^{1}H NMR), acetone-d_6 (δ = 29.84 ppm for ^{13}C NMR), and hexafluorobenzene (–162.0 ppm for ^{19}F NMR) in acetone-d_6, and $CHDCl_2$ (δ = 5.32 ppm for ^{1}H NMR) in CD_2Cl_2. No calibration was conducted for ^{19}F NMR measurement in CD_2Cl_2. The multiplicity of each signal for ^{1}H NMR was indicated by s (singlet), d (doublet), dd (double-doublet), t (triplet), dt (double-triplet), m (multiplet), and brs (broad-singlet). Assignment of ^{1}H and ^{13}C NMR signals was supported by 2D NMR spectroscopy. ESI-TOF mass spectra were recorded on a Micromass LCT spectrometer, in which high-resolution mass (HRMS) data were collected using a leucine enkephalin as an internal standard. UV-vis spectroscopy was performed using a JASCO V-770 spectrophotometer. CD spectra were recorded using a JASCO J-820 spectropolarimeter. Specific rotation was recorded using a JASCO P-1030 polarimeter. IR spectra were recorded on a JASCO FT/IR-4200 spectrometer using a ZnSe ATR method. Melting points were measured by YANACO MP-500D apparatus. Single-crystal X-ray diffraction (XRD) analyses were performed using a Rigaku XtaLAB P200 diffractometer under CuKα radiation with a CrysAlisPro software package and a R-AXIS RAPID II diffractometer under MoKα radiation with RAPID AUTO software package, and the obtained data were analysed using CrystalStructure and Olex2 crystallographic software packages[2] except for refinement, which was performed using SHELXL-2013 program suite[3]. TwinRotMat function in PLATON program was also used for single-crystal XRD analyses[4]. X-ray structures were displayed using a Mercury and PyMOL software packages. HPLC data were collected using a JASCO MD-4010 photodiode array detector connected with PU-4185-Binary binary RHPLC semi-micro pump, CO-4060 column oven, CD-4095 circular dichroism detector, and RI-4030 refractive index detector. Elemental analyses were conducted in Microanalytical Laboratory, Department of Chemistry, School of Science, the University of Tokyo using Vario MICRO Cube elemental analyser with addition of MgO, and creation of calibration curves and calculation of analytical values were carried out in Excel.

2. Synthesis and characterisation of 1_{tight} and 1_{loose}

2.1 Synthesis of $[Pd(^tBu_2bpy)(OH_2)_2](OTf)_2\cdot(H_2O)_2$[5,6]

$PdCl_2(MeCN)_2$ (80.1 mg, 0.309 mmol, 1.0 equiv.) and $^tBu_2bpy$ (83.5 mg, 0.311 mmol, 1.0 equiv.) were placed in a flask and dissolved in CH_2Cl_2 (20 mL). This reaction mixture was stirred at room temperature for 2 h. To this reaction mixture was added *n*-hexane to obtain a pale yellow precipitate, which was then isolated and dissolved in CH_2Cl_2 (15 mL). To this solution was added AgOTf (170.8 mg, 0.665 mmol, 2.4 equiv.), and the reaction solution was then stirred at room temperature for 1.5 h. After removal of Ag salts by filtration, the filtrate was evaporated. The resulting pale yellow solid was recrystallised from CH_2Cl_2 by slow diffusion of *n*-hexane to afford $[Pd(^tBu_2bpy)(OH_2)_2](OTf)_2\cdot(H_2O)_2$ (199.3 mg, 0.268 mmol, 86%) as pale yellow needle crystals.

^{1}H NMR (500 MHz, $CDCl_3$, 300 K): δ 8.24 (d, J = 6.5 Hz, 2H), 7.90 (d, J = 1.5 Hz, 2H), 7.66 (dd, J = 6.0, 2.0 Hz, 2H), 1.47 (s, 18H). ^{13}C NMR (126 MHz, $CDCl_3$, 300 K): δ 168.5, 156.1, 149.5, 125.3, 120.1, 36.5, 30.4. Anal. Calcd for $C_{20}H_{32}F_6N_2O_{10}PdS_2$ $\{[Pd(^tBu_2bpy)(OH_2)_2](OTf)_2\cdot(H_2O)_2\}$: C 32.24, H 4.33, N 3.76; found: C 32.03, H 4.01, N 3.76.

Crystal data for $[Pd(^tBu_2bpy)(OH_2)_2]_2(OTf)_4\cdot(CH_2Cl_2)$: $C_{41}H_{50}Cl_2F_{12}N_4O_{16}Pd_2S_4$, F_w = 1494.79, crystal dimensions 0.135 × 0.029 × 0.026 mm^3, triclinic, space group *P*-1, a = 13.7738(4), b = 15.5003(5), c = 16.4954(6) Å, α = 84.224(3), β = 67.154(3), γ = 65.395(3)°, V = 2942.94(19) Å^3, Z = 2, ρ_{calcd} = 1.687 g cm^{-3}, μ = 80.20 cm^{-1}, T = 98 K, λ(CuKα) = 1.54184 Å, $2\theta_{max}$ = 162.806°, 57057/12257 reflections collected/unique (R_{int} = 0.1374), R_1 = 0.0972 ($I > 2\sigma(I)$), wR_2 = 0.2684 (for all data), GOF = 1.029, largest diff. peak and hole 2.179/–1.103 eÅ^{-3}. CCDC deposit number 2190131.

PLAT430_ALERT_2_B Short Inter D…A Contact

Response: The short contacts come from H_2O…OTf, though hydrogen atoms of the water molecule could not be located in the difference electron density maps.

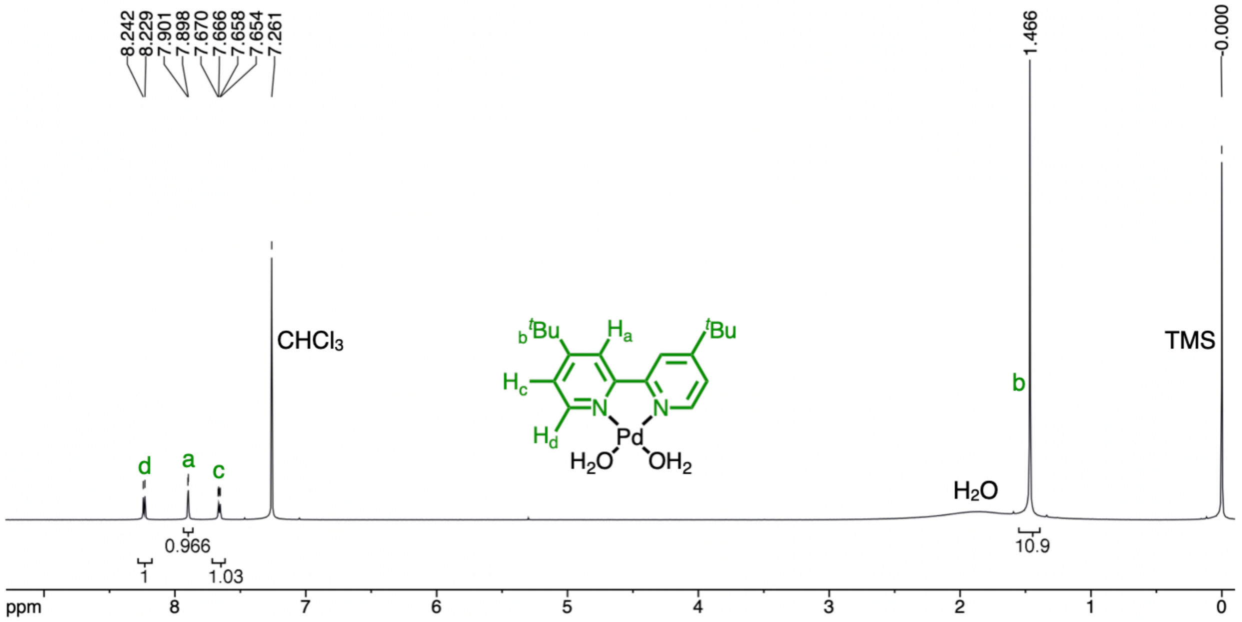

Supplementary Fig. 1 ^{1}H NMR spectrum of $[Pd(^tBu_2bpy)(OH_2)_2](OTf)_2$ (500 MHz, $CDCl_3$, 300 K).

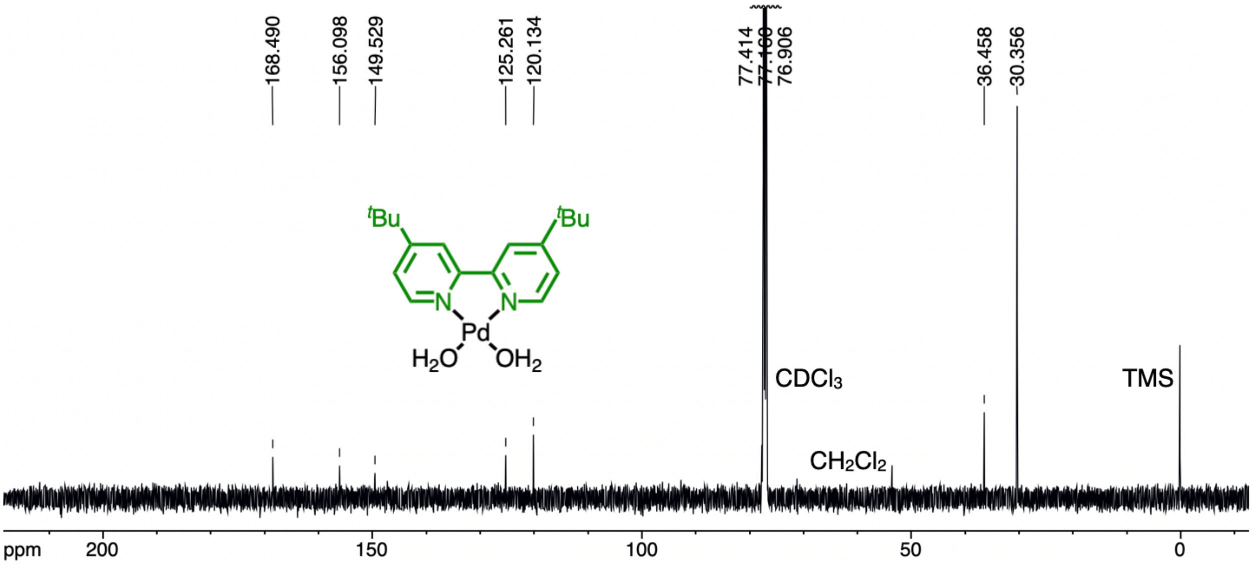

Supplementary Fig. 2 ^{13}C NMR spectrum of [Pd(^{t}Bu$_2$bpy)(OH$_2$)$_2$](OTf)$_2$ (126 MHz, CDCl$_3$, 300 K).

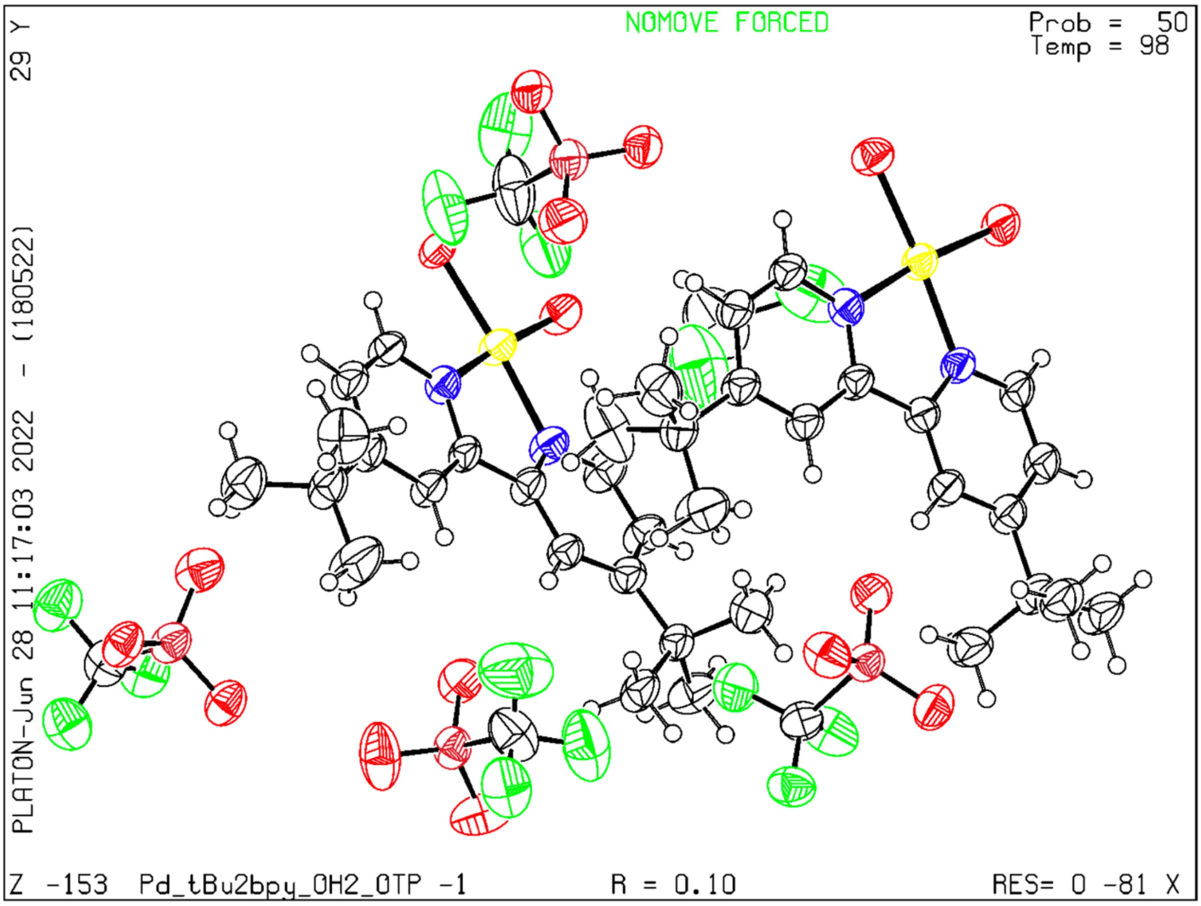

Supplementary Fig. 3 ORTEP drawing of [Pd(^{t}Bu$_2$bpy)(OH$_2$)$_2$](OTf)$_2$ at the 50% probability level. Colour: C black, N blue, O red, F yellow green, S dark red, Cl yellow green and Pd yellow. CCDC deposit number of [Pd(^{t}Bu$_2$bpy)(OH$_2$)$_2$](OTf)$_2$ is 2190131. This figure was produced by the checkCIF report of the International Union of Crystallography.

2.2 Synthesis of $\mathbf{1}_{\mathbf{tight}}$

A CH_2Cl_2 solution (1.0 mL) of **L** (10.0 mg, 15.9 µmol, 1.0 equiv.) was mixed with a CH_2Cl_2 solution (2.0 mL) of $[Pd(^tBu_2bpy)(OH_2)_2](OTf)_2{\cdot}(H_2O)_2$ (38.2 mg, 51.3 µmol, 3.2 equiv.), and then stirred at room temperature for 4 h. During the reaction, the colour of the solution was changed from pale yellow to purple in a few minutes. The reaction mixture was filtered to remove precipitates and the filtrate was evaporated. The resulting solid was washed with $CHCl_3$ and the residue was dried up under reduced pressure. The solid was recrystallised from CH_2Cl_2 by vapour diffusion of Et_2O. The obtained plate crystals were washed with a small amount of $CHCl_3$ and dried up under reduced pressure to afford tightly-twisted $\mathbf{1}_{\mathbf{tight}}$, $[Pd_3\mathbf{L}(^tBu_2bpy)_3](OTf)_6{\cdot}(H_2O)_{4.9}{\cdot}(Et_2O)_{0.15}$, (14.27 mg, 5.19 µmol, 33% yield) as a colourless solid.

Mp: > 257 °C (decomp.). ^{1}H NMR (500 MHz, acetone-d_6, 300 K): δ 9.81 (d, J = 7.0 Hz, 3H), 9.09 (d, J = 6.0 Hz, 3H), 9.00 (d, J = 6.0 Hz, 3H), 8.95 (d, J =1.5 Hz, 3H), 8.88 (d, J = 2.0 Hz, 3H), 8.85 (d, J = 3.5 Hz, 3H), 8.10 (dd, J = 5.0, 1.0 Hz, 3H), 8.03 (dd, J = 6.0, 1.5 Hz, 3H), 7.98 (d, J = 8.5 Hz, 3H), 7.80 (d, J = 5.5 Hz, 3H), 7.64 (t, J = 8.0 Hz, 3H), 7.41 (d, J = 10.5 Hz, 3H), 7.10 (t, J = 7.5 Hz, 3H), 6.94 (d, J = 7.0 Hz, 3H), 6.40 (d, J = 7.0 Hz, 3H), 5.09 (d, J = 14.5 Hz, 3H), 5.05 (d, J = 15.0 Hz, 3H), 4.89 (d, J = 7.5 Hz, 3H), 4.87 (d, J = 13.5 Hz, 3H), 4.50 (dd, J = 14.0, 11.5 Hz, 3H), 1.56 (s, 27H), 1.50 (s, 27H). ^{13}C NMR (126 MHz, acetone-d_6, 301 K): δ 168.9, 168.7, 157.8, 157.3, 152.2, 150.4, 142.0, 141.8, 134.5, 134.2, 133.4, 133.1, 132.1, 131.9, 131.2, 129.2, 128.6, 126.9, 126.6, 126.5, 123.3, 123.2, 122.9, 120.8, 118.2, 63.0, 61.3, 37.1, 37.0. The ^{13}C signals of *tert*-butyl groups were overlapped with those of acetone-d_6 and could not be identified. The TfO anion has four ^{13}C signals but only three signals were observed due to the low S/N ratio. ^{19}F NMR (471 MHz, acetone-d_6, 300 K): δ –75.4, –76.1. ^{1}H NMR (500 MHz, CD_2Cl_2, 300 K): δ 9.62 (d, J = 7.5 Hz, 3H), 9.25 (d, J = 4.0 Hz, 3H), 8.98 (d, J = 6.5 Hz, 3H), 8.73 (d, J = 6.0 Hz, 3H), 8.31 (d, J = 1.5 Hz, 3H), 8.25 (d, J = 1.5 Hz, 3H), 7.99 (dd, J = 6.0, 1.5 Hz, 3H), 7.90 (dd, J = 6.0, 2.0 Hz, 3H), 7.86 (d, J = 8.5 Hz, 3H), 7.69 (d, J = 7.5 Hz, 3H), 7.55 (t, J = 8.0 Hz, 3H), 7.40 (d, J = 10.5 Hz, 3H), 6.68 (d, J = 7.5 Hz, 3H), 6.68 (t, J = 7.5 Hz, 3H), 6.29 (d, J = 7.5 Hz, 3H), 4.83 (d, J = 11.5 Hz, 3H), 4.81 (d, J = 11.0 Hz, 3H), 4.75 (d, J = 8.0 Hz, 3H), 4.61 (dd, J = 14.0, 4.0 Hz, 3H), 4.18 (dd, J = 14.0, 11.5 Hz, 3H), 1.57 (s, 27H), 1.51 (s, 27H). IR (ATR, cm^{-1}): 3144 (br), 2967, 1618, 1417, 1248, 1155, 1028, 810, 636. UV-vis (CH_2Cl_2, 293 K, 87.3 µM): λ_{max} (nm) (ε (M^{-1} cm^{-1})) = 309.8 (3.98 × 10^4). HRMS (ESI-TOF): m/z = 1100.2498 as $[Pd_3(H_{-2}\mathbf{L})(^tBu_2bpy)_3](OTf)_3]^+$ (calcd 1100.2462). Anal. Calcd for $C_{102.6}H_{125.3}F_{18}N_{12}O_{23.05}Pd_3S_6$ $\{[Pd_3\mathbf{L}(^tBu_2bpy)_3](OTf)_6{\cdot}(H_2O)_{4.9}{\cdot}(Et_2O)_{0.15}\}$: C 44.82, H 4.58, N 6.09; found: C 44.81, H 4.58, N 6.09.

Crystal data for tightly-twisted $Pd_3\mathbf{L}(^tBu_2bpy)_3{\cdot}(OTf)_{5.08}{\cdot}(H_2O)_{6.95}{\cdot}(CH_2Cl_2)_{1.27}$ (missing triflates were not observed due to severe disorder): $C_{102.36}H_{116.55}Cl_{2.55}F_{15.25}N_{12}O_{22.20}Pd_3S_{5.08}$, F_w = 2732.41, crystal dimensions 0.131 × 0.081 × 0.031 mm^3, trigonal, space group R-3, a = 23.0889(2), c = 41.6772(6) Å, V = 19241.3(4) Å^3, Z = 6, ρ_{calcd} = 1.415 g cm^{-3}, μ = 53.78 cm^{-1}, T = 93 K, λ(CuKα) = 1.54187 Å, $2\theta_{max}$ = 144.478°, 39889/8311 reflections collected/unique (R_{int} = 0.0578), R_1 = 0.0845 ($I > 2\sigma(I)$),

wR_2 = 0.2630 (for all data), GOF = 1.113, largest diff. peak and hole 1.414/–1.123 eÅ^{-3}. CCDC deposit number 2190130.

PLAT306_ALERT_2_B Isolated Oxygen Atom (H-atoms missing?)

Response: Hydrogen atoms of water molecules could not be located in the difference electron density maps.

PLAT430_ALERT_2_B Short Inter D…A Contact

Response: The short contacts come from H_2O-TfO, though hydrogen atoms of water molecules could not be located in the difference electron density maps.

PLAT601_ALERT_2_B Unit Cell Contains Solvent Accessible VOIDS of ...125 Ang**3

Response: Some solvents and TfO anions in the large pore could not be located due to severe disordering.

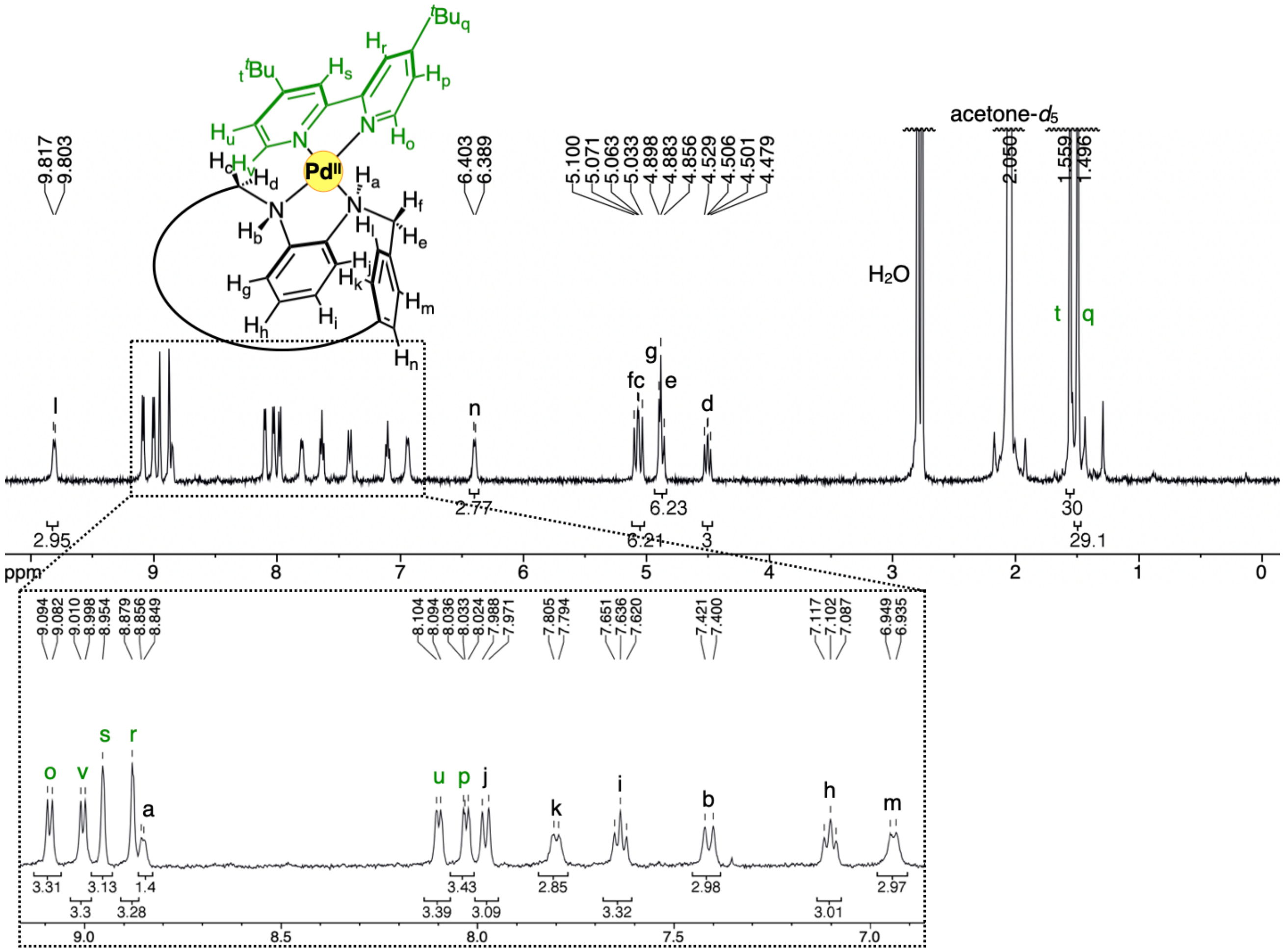

Supplementary Fig. 4 ^{1}H NMR spectrum of $\mathbf{1}_{\mathbf{tight}}$ (500 MHz, acetone-d_6, 300 K).

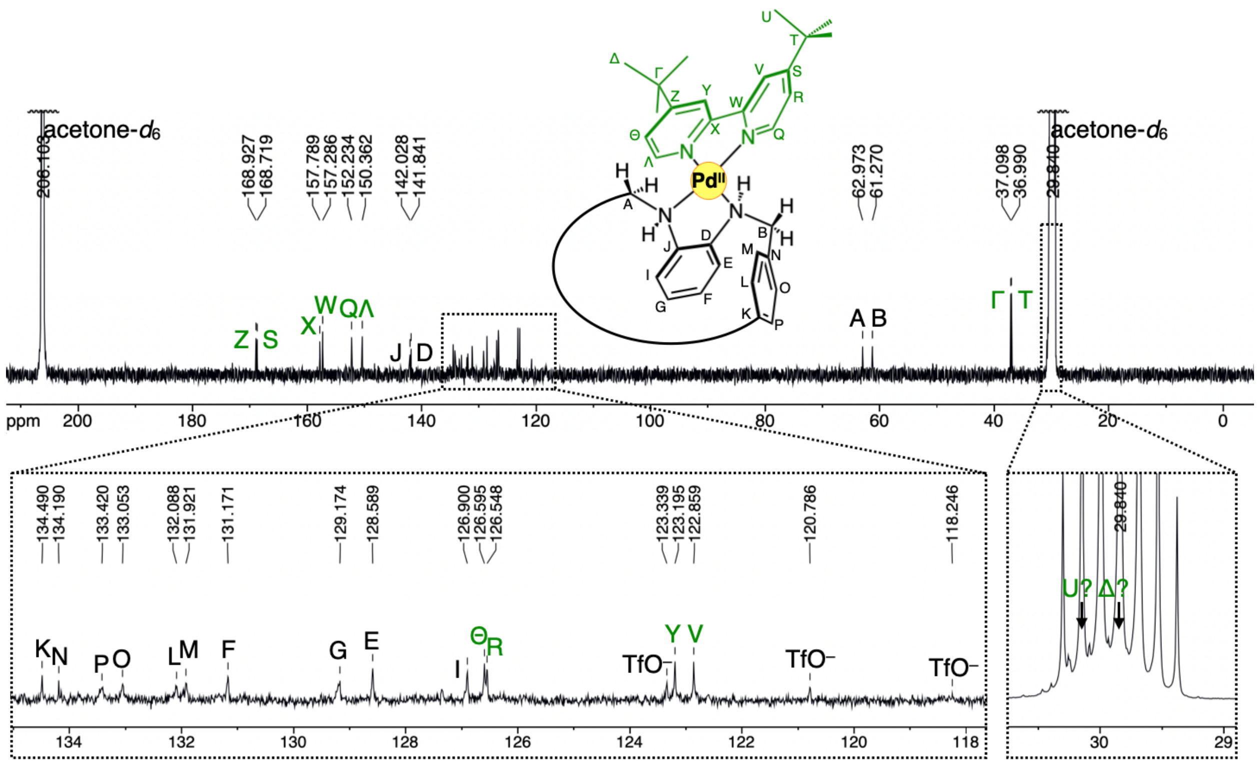

Supplementary Fig. 5 ^{13}C NMR spectrum of $\mathbf{1}_{\mathbf{tight}}$ (126 MHz, acetone-d_6, 301 K).

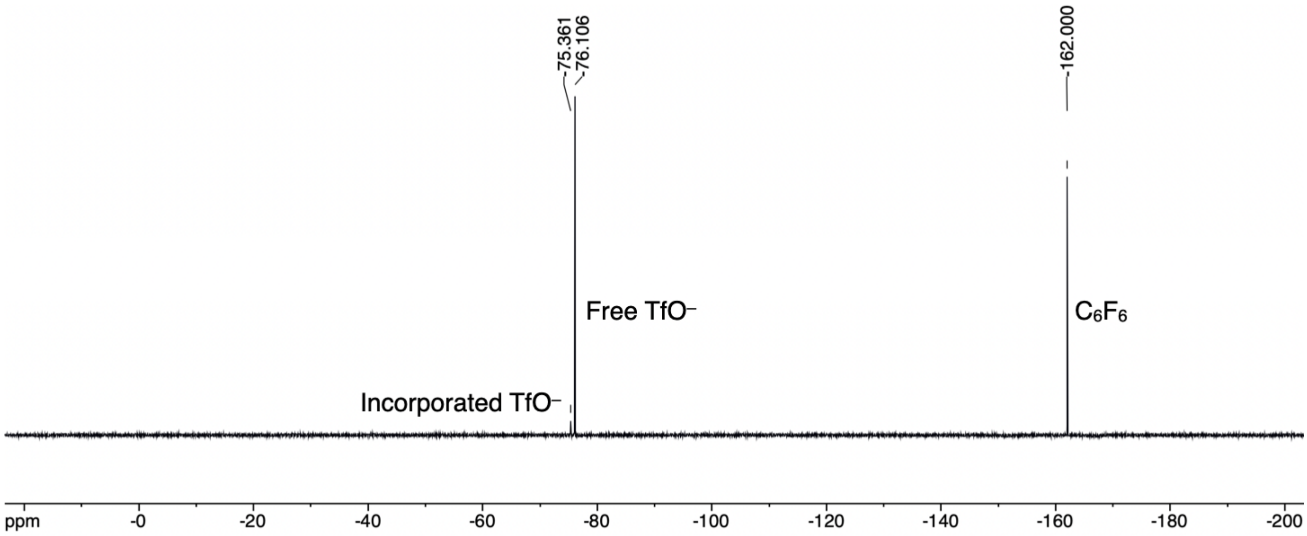

Supplementary Fig. 6 ^{19}F NMR spectrum of $\mathbf{1}_{\mathbf{tight}}$ (471 MHz, acetone-d_6, 300 K).

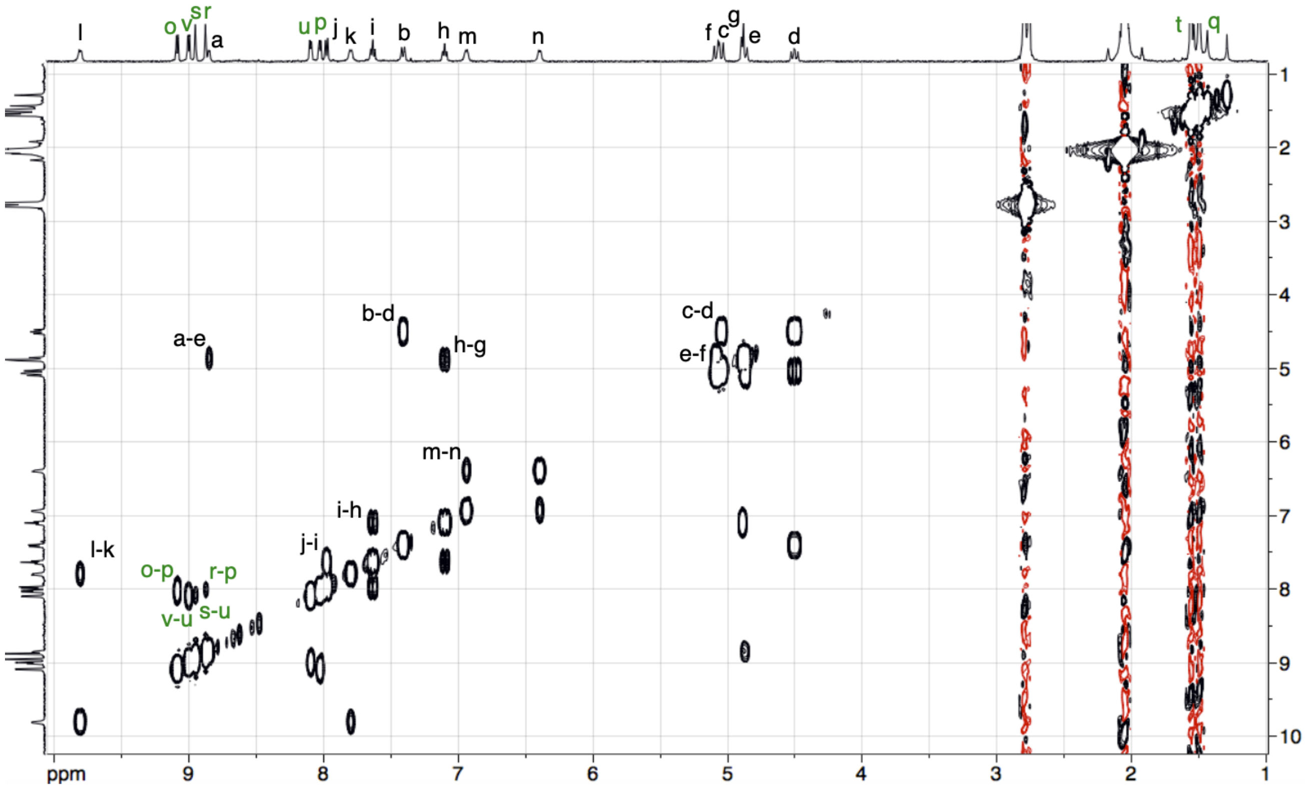

Supplementary Fig. 7 ^{1}H-^{1}H COSY NMR spectrum of $\mathbf{1}_{\mathbf{tight}}$ (500 MHz, acetone-d_6, 300 K).

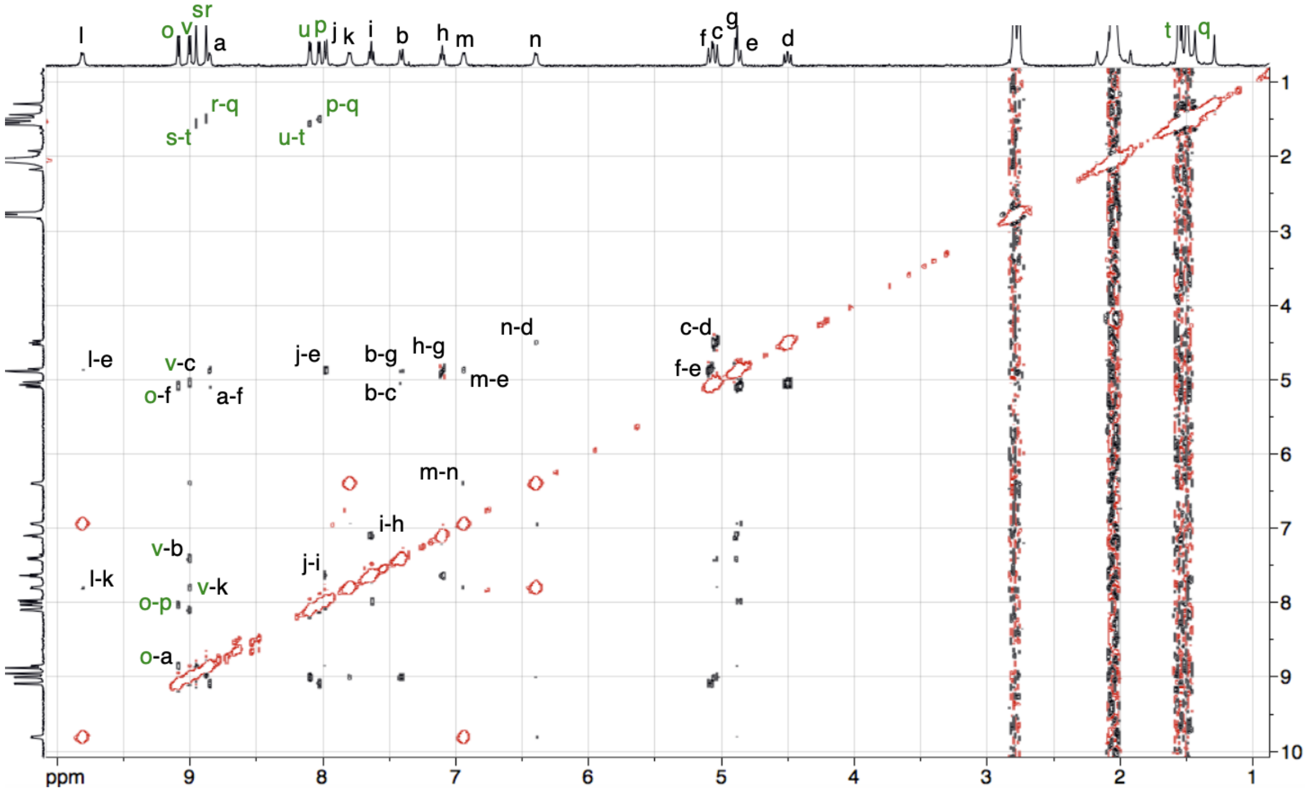

Supplementary Fig. 8 ^{1}H-^{1}H ROESY NMR spectrum of $\mathbf{1}_{\mathbf{tight}}$ (500 MHz, acetone-d_6, 300 K).

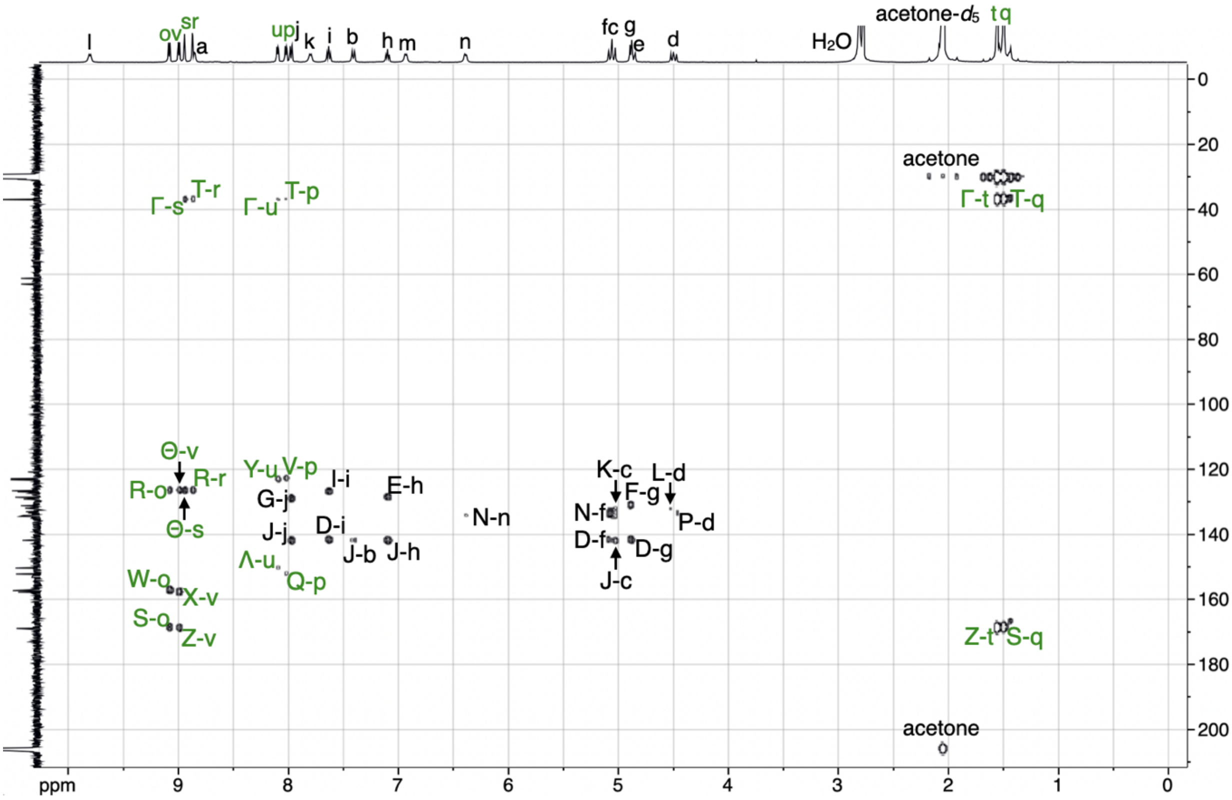

Supplementary Fig. 9 ^{1}H-^{13}C HSQC NMR spectrum of $\mathbf{1}_{\mathbf{tight}}$ (500 MHz for ^{1}H and 126 MHz for ^{13}C, acetone-d_6, 300 K).

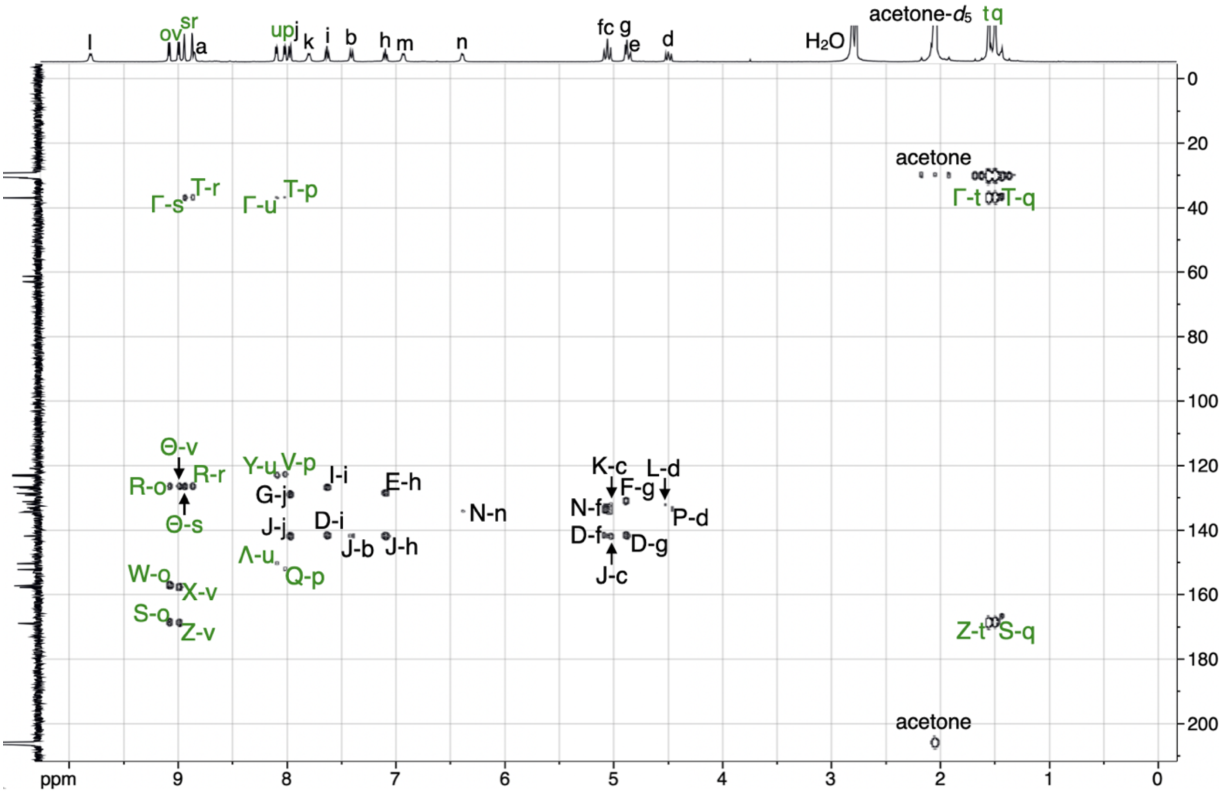

Supplementary Fig. 10 ^{1}H-^{13}C HMBC NMR spectrum of $\mathbf{1}_{\mathbf{tight}}$ (500 MHz for ^{1}H and 126 MHz for ^{13}C, acetone-d_6, 300 K).

^{1}H NMR signals of $\mathbf{1}_{\mathbf{tight}}$ in CD_2Cl_2 were also assigned by 2D NMR measurement as shown below.

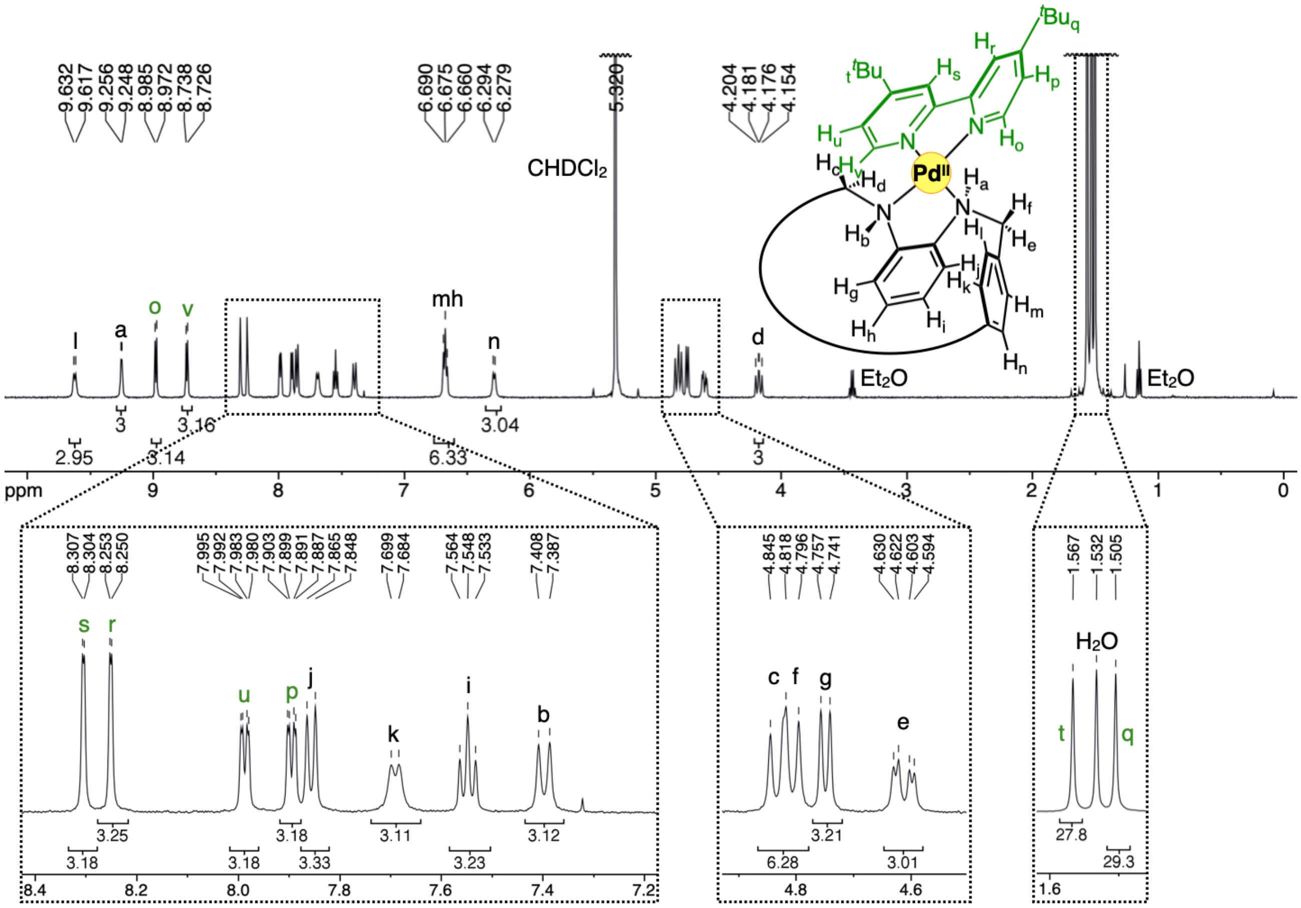

Supplementary Fig. 11 ^{1}H NMR spectrum of $\mathbf{1}_{\mathbf{tight}}$ (500 MHz, CD_2Cl_2, 300 K).

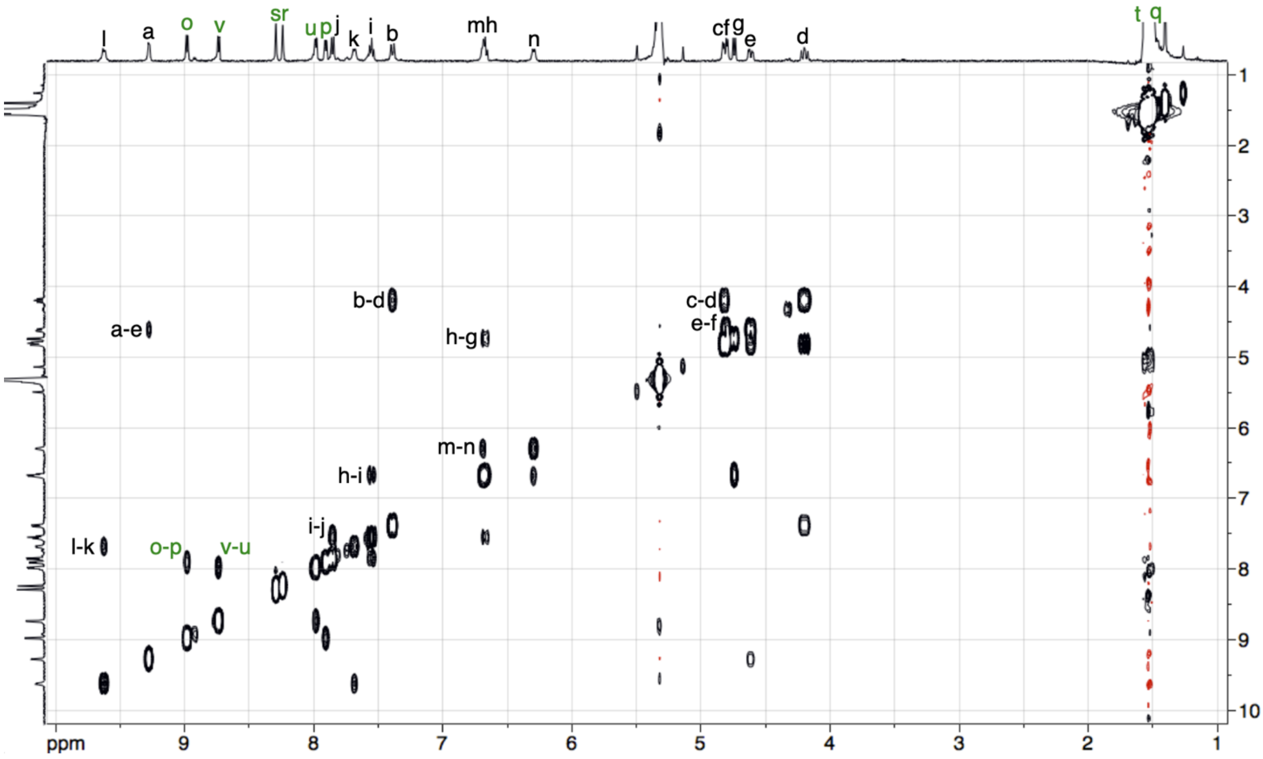

Supplementary Fig. 12 ^{1}H-^{1}H COSY NMR spectrum of $\mathbf{1}_{\mathbf{tight}}$ (500 MHz, CD_2Cl_2, 300 K).

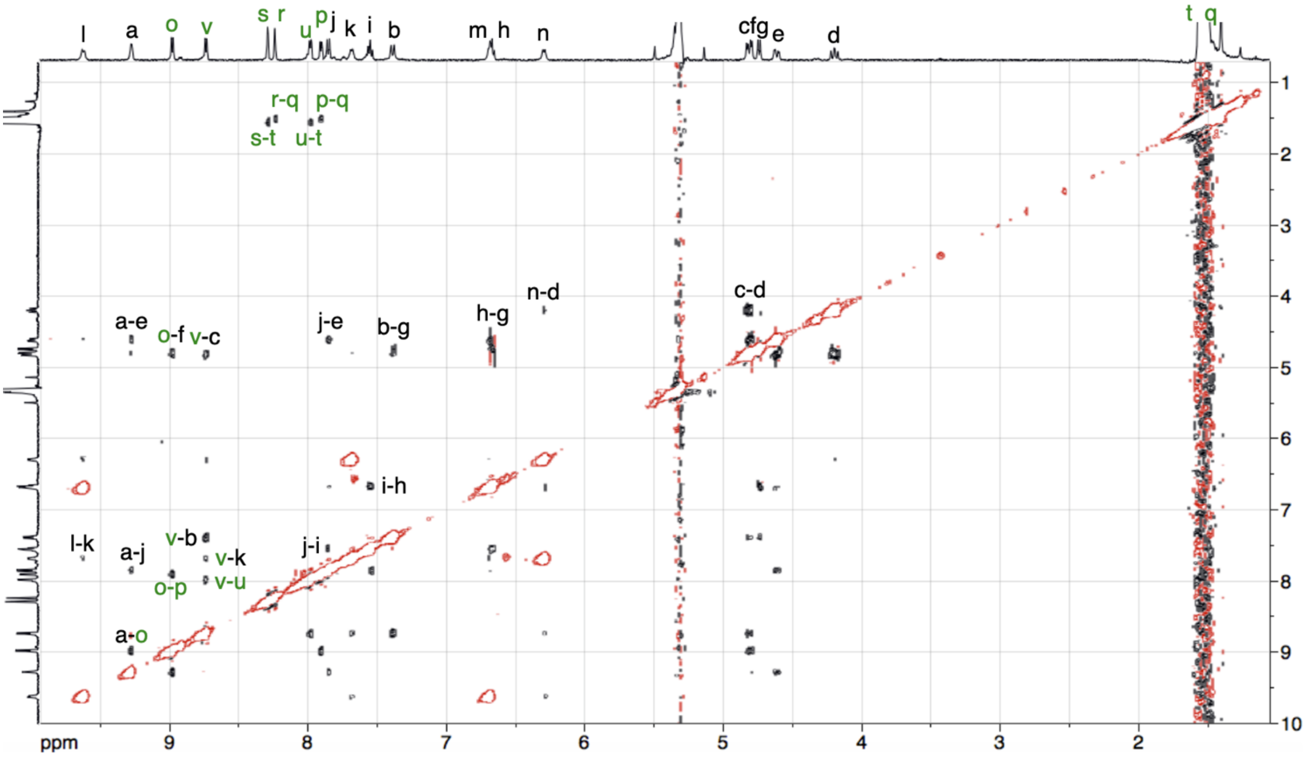

Supplementary Fig. 13 ^{1}H-^{1}H ROESY NMR spectrum of $\mathbf{1}_{\mathbf{tight}}$ (500 MHz, CD_2Cl_2, 300 K).

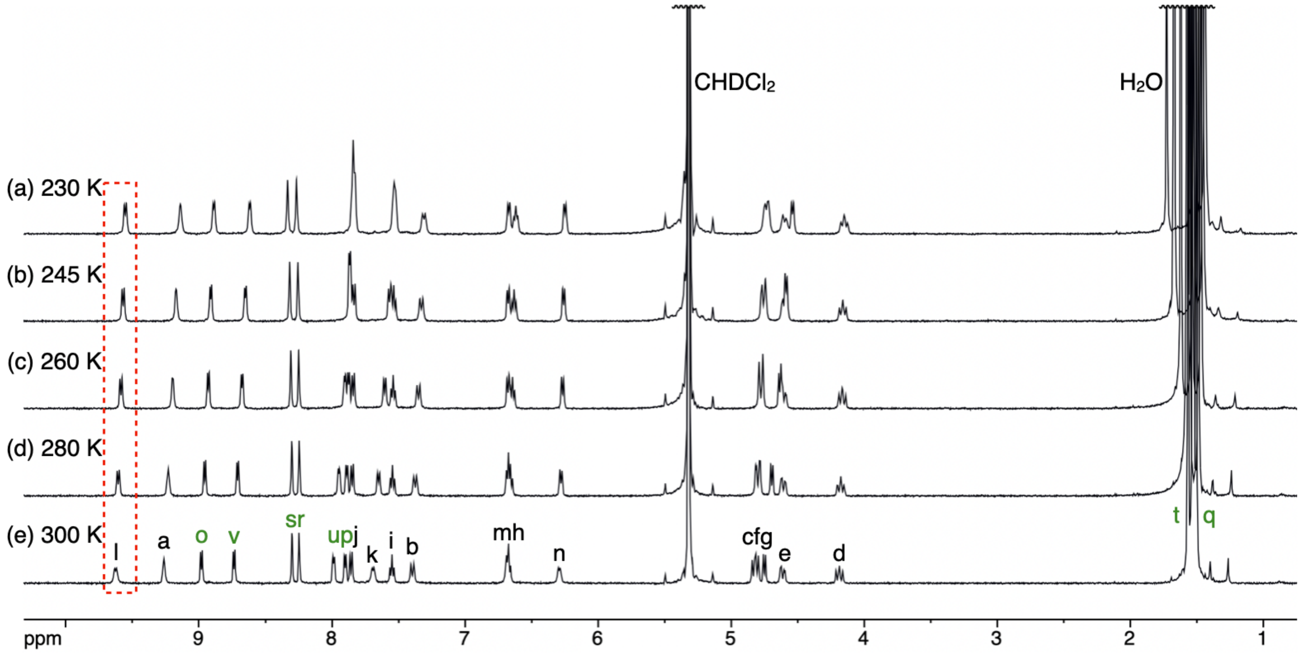

Supplementary Fig. 14 VT ^{1}H NMR spectra of $\mathbf{1}_{\mathbf{tight}}$ measured at (a) 230 K, (b) 245 K, (c) 260 K, (d) 280 K and (e) 300 K (500 MHz, CD_2Cl_2). The downfield shift of one *para*-phenylene signal (H_l) was maintained in the range from 230 to 300 K, suggesting the presence of C-H···Pd anagostic interactions even at 300 K in the solution.

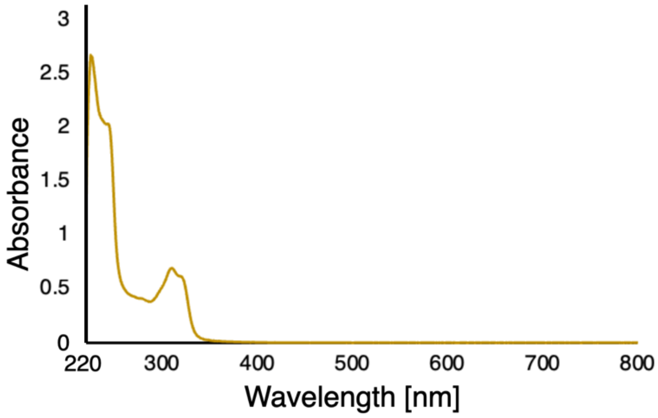

Supplementary Fig. 15 UV-vis spectrum of $\mathbf{1}_{\mathbf{tight}}$ (87.3 μM, l = 0.2 cm, 293 K in CH_2Cl_2).

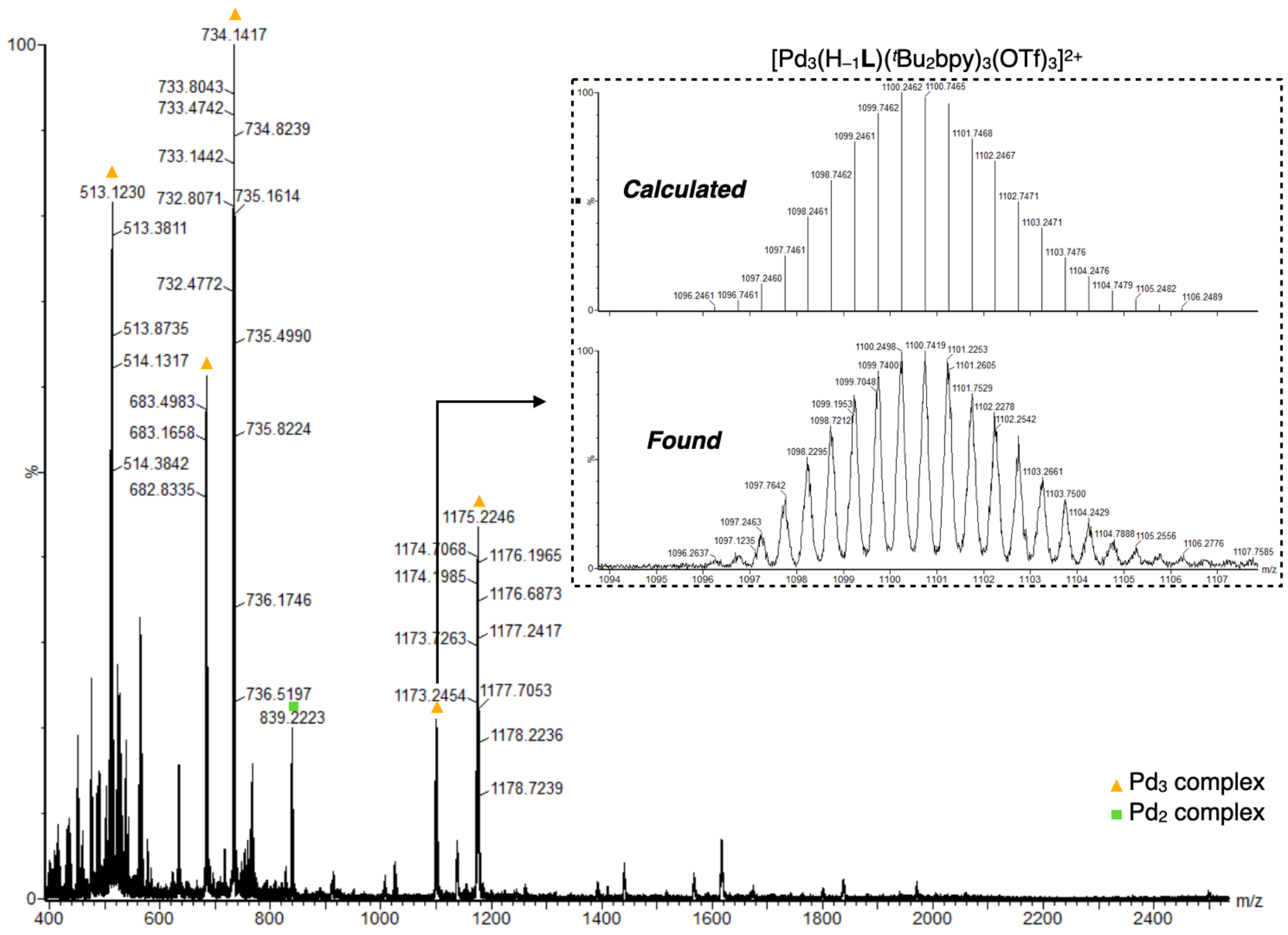

Supplementary Fig. 16 High resolution-ESI-TOF mass spectrum of $\mathbf{1}_{\mathbf{tight}}$ (positive, acetone).

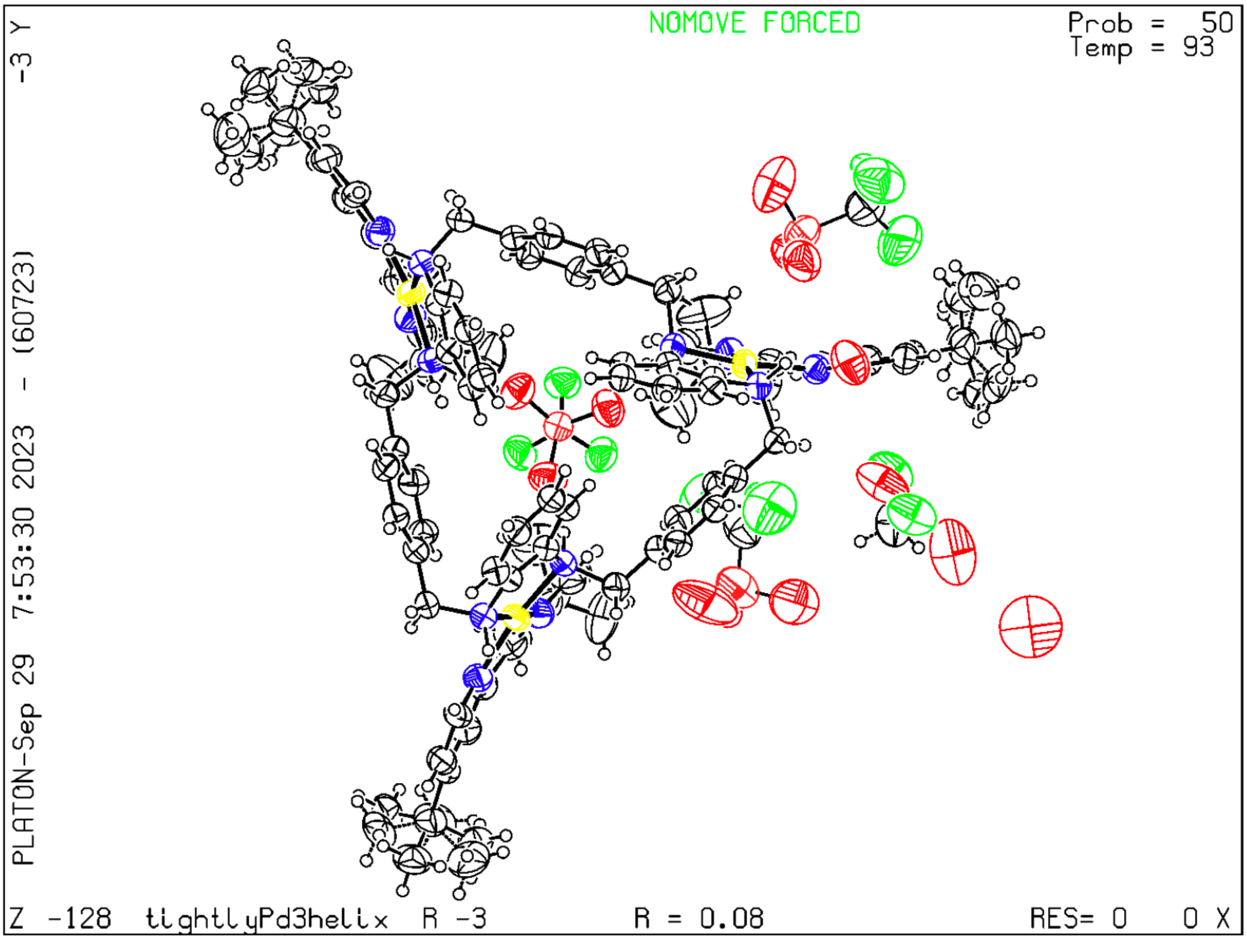

Supplementary Fig. 17 ORTEP drawing of $\mathbf{1}_{\mathbf{tight}}$ at the 50% probability level. Colour: C black, N blue, O red, F yellow green, S dark red and Pd yellow. CCDC deposit number of $\mathbf{1}_{\mathbf{tight}}$ is 2190130. This figure was produced by the checkCIF report of the International Union of Crystallography.

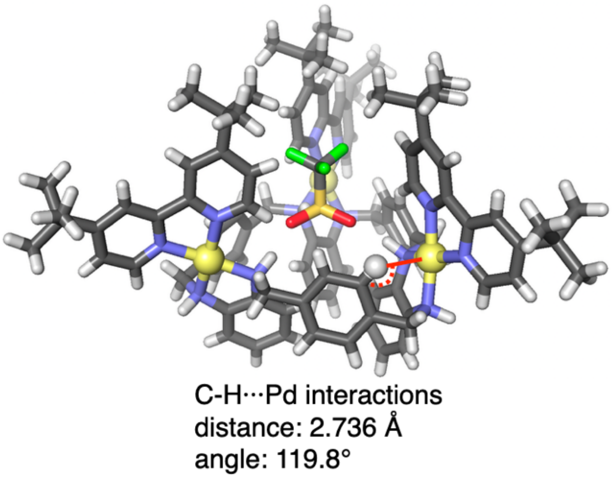

Supplementary Fig. 18 Crystal structure of $\mathbf{1}_{\mathbf{tight}}$ with the distance and angle of C-H···Pd interactions.

2.3 Synthesis of 1_{loose}

A $CHCl_3$ solution (2.2 mL) of **L** (13.2 mg, 20.9 μmol, 1.0 equiv.) was mixed with a $CHCl_3$ solution (4.7 mL) of $[Pd(^tBu_2bpy)(OH_2)_2](OTf)_2\cdot(H_2O)_2$ (25.2 mg, 33.8 μmol, 1.6 equiv.), and then stirred at room temperature for 3 h. During the reaction, a pink solid was precipitated. The resulting precipitate was collected by filtration and washed with $CHCl_3$ to obtain a dark pink solid whose main component was dinuclear metallocycle **2** (21.3 mg, 10.7 μmol, 64%), which was then suspended in CH_2Cl_2 (10 mL). To the suspension was added a CH_2Cl_2 solution (5 mL) of $[Pd(^tBu_2bpy)(OH_2)_2](OTf)_2\cdot(H_2O)_2$ (9.7 mg, 13.0 μmol, 1.2 equiv. to the dinuclear metallocycle). The reaction mixture was stirred at room temperature for 3 h, and then heated at reflux for 1.5 h. During heating, a colourless solid was precipitated. The resulting precipitate was collected by filtration and dried under reduced pressure. This solid was recrystallised from acetone by vapour diffusion of Et_2O to afford loosely-twisted $\mathbf{1}_{\mathbf{loose}}$, $[Pd_3\mathbf{L}(^tBu_2bpy)_3](OTf)_6\cdot(H_2O)_4$, (14.2 mg, 5.21 μmol, 31% in total) as colourless plate crystals.

Mp: > 272 °C (decomp.). ^{1}H NMR (500 MHz, acetone-d_6, 300 K): δ 8.99 (d, J = 6.0 Hz, 3H), 8.69 (s, 3H), 8.62 (d, J = 1.5 Hz, 3H), 8.47 (d, J = 1.5 Hz, 3H), 8.10 (d, J = 8.0 Hz, 3H), 8.00 (brs, 3H), 7.91 (dd, J = 5.5, 1.0 Hz, 3H), 7.82 (brs, 3H), 7.68 (t, J = 7.5 Hz, 3H), 7.62 (brs, 3H), 7.55 (m, 6H), 7.47 (brs, 3H), 7.19 (d, J = 5.0 Hz, 3H), 6.94 (d, J = 5.0 Hz, 3H), 6.75 (brs, 3H), 4.78 (d, J = 13.0 Hz, 3H), 4.23 (d, J = 13.0 Hz, 3H), 3.21 (d, J = 11.5 Hz, 3H), 1.88 (brs, 3H), 1.54 (s, 27H), 1.43 (s, 27H). ^{13}C NMR (126 MHz, acetone-d_6, 300 K): δ 168.6, 166.9, 158.3, 156.4, 151.4, 150.0, 145.6, 141.7, 135.1, 135.1, 134.8, 134.0, 133.8, 133.6, 131.3, 131.3, 127.3, 125.8, 125.7, 123.8, 123.2, 122.8, 120.7, 118.1, 63.1, 60.4, 36.9, 36.7, 30.8, 30.2. ^{1}H NMR signals of *para*-phenylene moieties were not fully assigned at 300 K, because the rotation of the *para*-phenylene moieties was so fast that extra cross signals derived from chemical exchange processes appeared in the ROESY spectrum and disturbed the assignment of the signals. So, the ^{1}H NMR signals of *para*-phenylene moieties were assigned by 2D ^{1}H-^{1}H COSY and ROESY NMR analyses at 270 K where the rotation of *para*-phenylene moieties was slow enough.

IR (ATR, cm^{-1}): 3459 (br), 2975, 1620, 1417, 1240, 1156, 1027, 844. UV-vis (CH_3CN, 293 K, 82.3 μM): λ_{max} (nm) (ε (M^{-1} cm^{-1})) = 311 (3.69 × 10^4). HRMS (ESI-TOF): m/z = 1100.2410 as $[Pd_3(H_{-1}\mathbf{L})(^tBu_2bpy)_3](OTf)_4]^+$ (calcd 1100.2462). Anal. Calcd for $C_{102}H_{122}F_{18}N_{12}O_{22}Pd_3S_6$ $\{[Pd_3\mathbf{L}(^tBu_2bpy)_3](OTf)_6\cdot(H_2O)_4\}$: C 45.01, H 4.52, N 6.18; found: C 45.04, H 4.52, N 6.17.

Crystal data for loosely-twisted $Pd_3\mathbf{L}(^tBu_2bpy)_3\cdot(OTf)_6\cdot(C_3H_6O)_{0.375}\cdot(H_2O)_{0.5}$: $C_{103.12}H_{116.25}F_{18}N_{12}O_{18.88}Pd_3S_6$, F_w = 2679.38, crystal dimensions 0.221 × 0.150 × 0.087 mm^3, trigonal, space group P-3c1, a = 23.8678(5), c = 29.5387(5) Å, V = 14572.9(7) Å^3, Z = 4, ρ_{calcd} = 1.221 g cm^{-3}, μ = 44.34 cm^{-1}, T = 93.15 K, λ(CuKα) = 1.54178 Å, 2θ_{max} = 134.130°, 30304/8396 reflections collected/unique (R_{int} = 0.0431), R_1 = 0.1399 ($I > 2\sigma(I)$), wR_2 = 0.3524 (for all data), GOF = 1.473, largest diff. peak and hole 6.128/–2.917 eÅ^{-3}. CCDC deposit number 2190129.

PLAT602_ALERT_2_A Solvent Accessible VOID(S) in Structure

Response: Some solvents in the large pore could not be located due to severe disordering.

PLAT097_ALERT_2_B Large Reported Max. (Positive) Residual Density … 6.13 eA^{-3}

Response: The atom type is correct. The large residual density may be due to an anomalous dispersion effect and has no chemical significance.

PLAT306_ALERT_2_B Isolated Oxygen Atom (H-atoms Missing?)

Response: Hydrogen atoms of water molecules could not be located in the difference electron density maps.

PLAT342_ALERT_3_B Low Bond Precision on C-C Bonds … 0.02019 Ang.

Response: Some solvents in the large pore could not be located due to severe disordering.

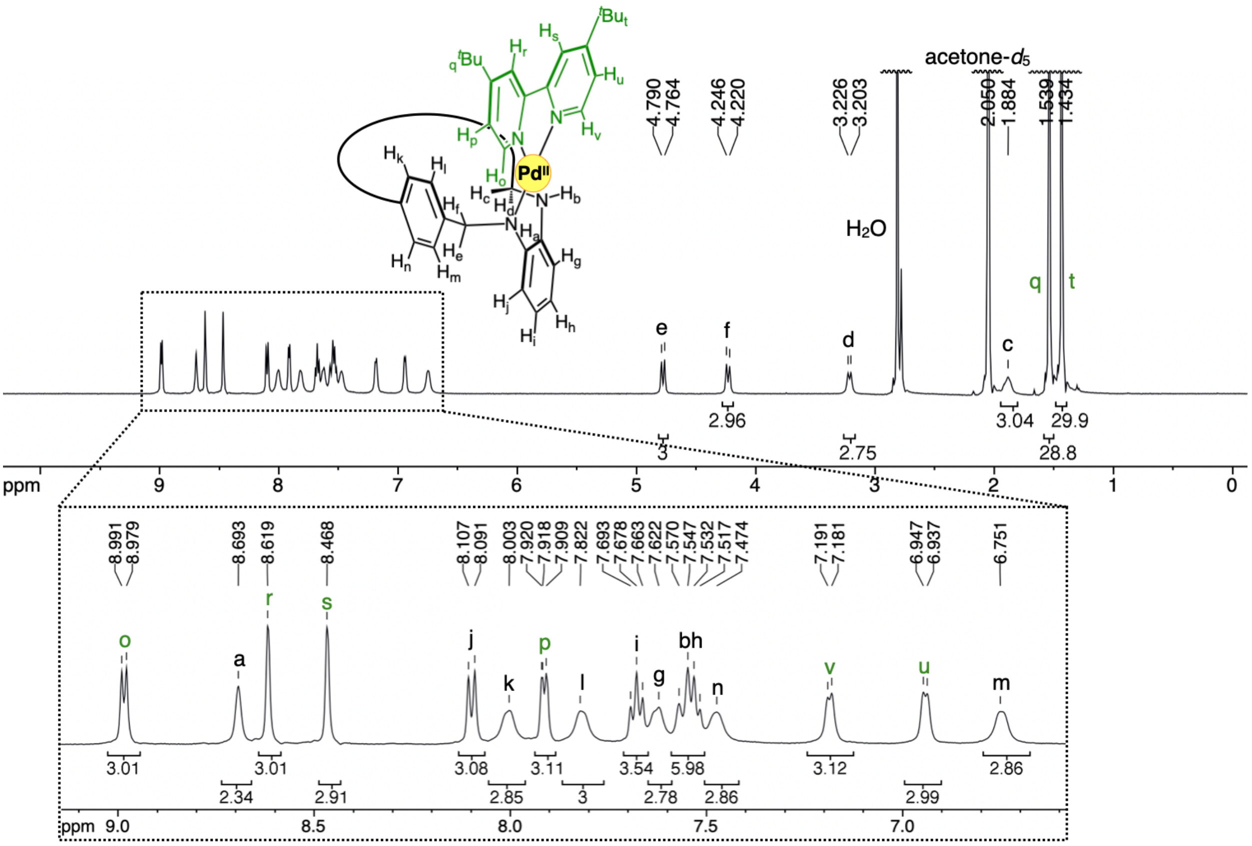

Supplementary Fig. 19 ^{1}H NMR spectrum of $\mathbf{1}_{\mathbf{loose}}$ (500 MHz, acetone-d_6, 300 K).

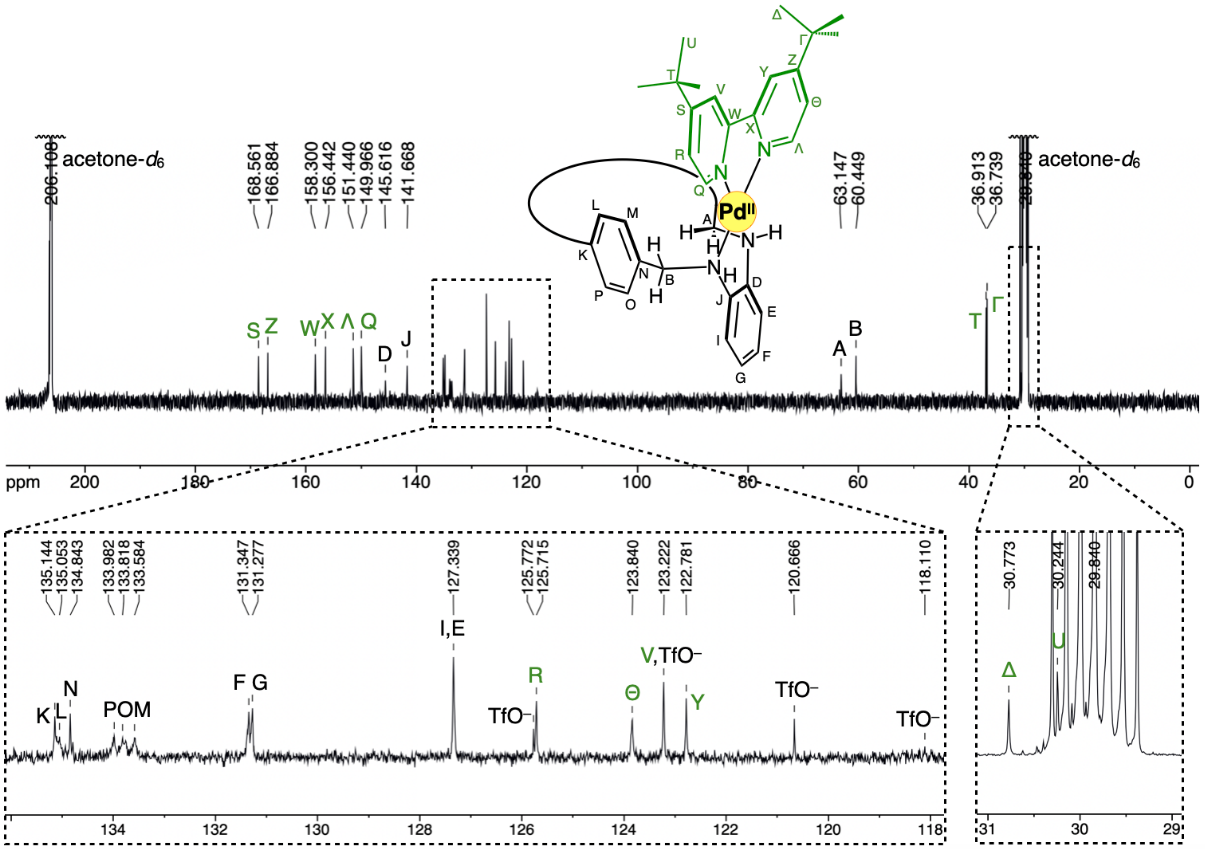

Supplementary Fig. 20 ^{13}C NMR spectrum of **1**$_{\mathbf{loose}}$ (126 MHz, acetone-d_6, 300 K).

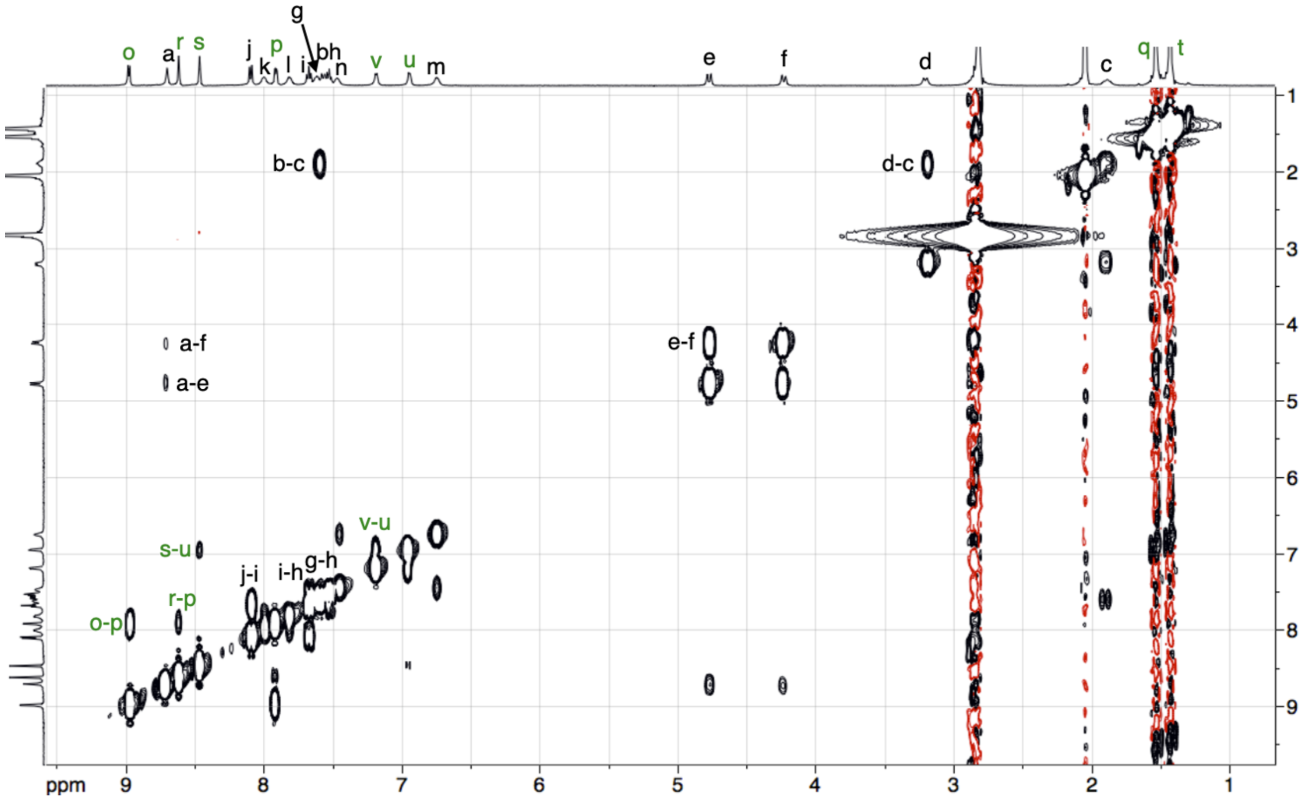

Supplementary Fig. 21 ^{1}H-^{1}H COSY NMR spectrum of **1**$_{\mathbf{loose}}$ (500 MHz, acetone-d_6, 300 K).

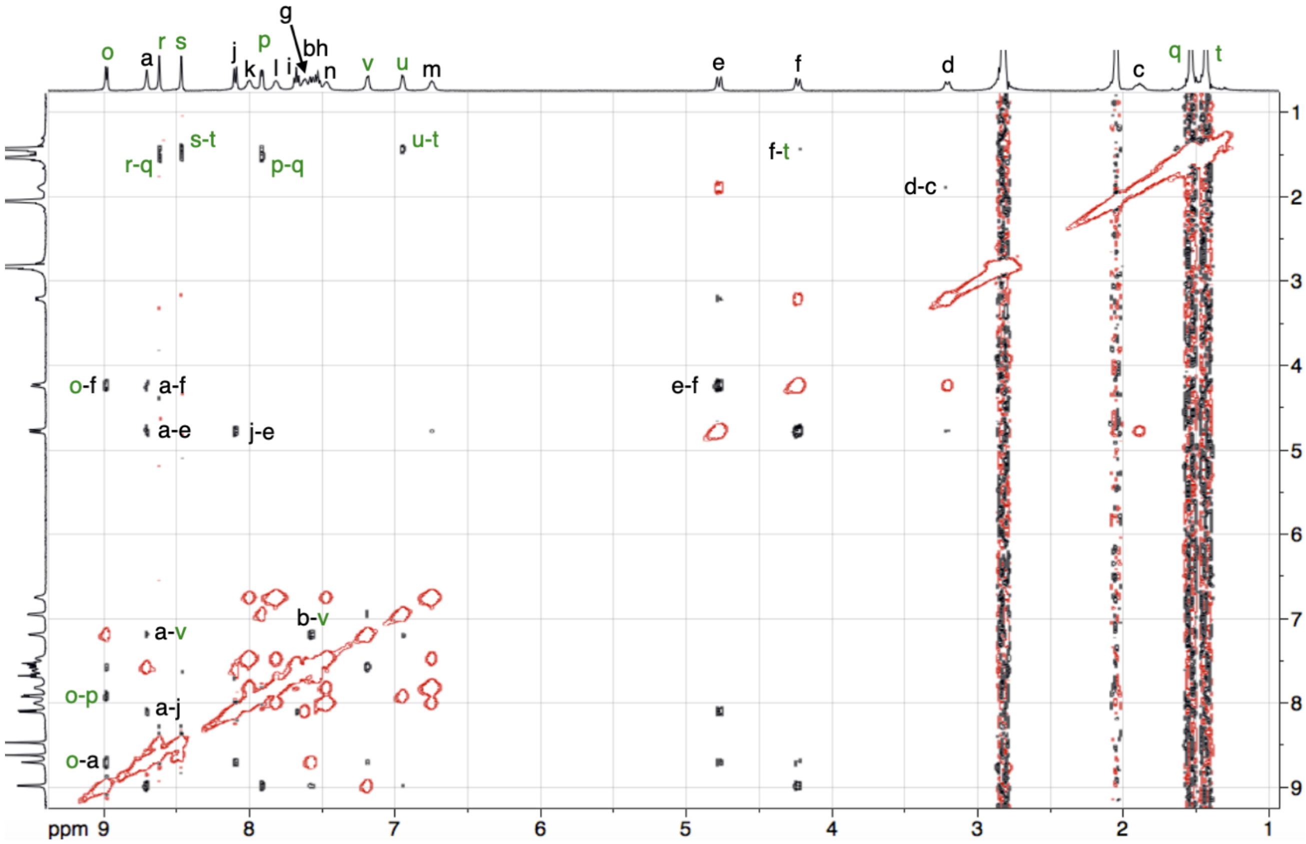

Supplementary Fig. 22 ^{1}H-^{1}H ROESY NMR spectrum of $\mathbf{1}_{\mathbf{loose}}$ (500 MHz, acetone-d_6, 300 K).

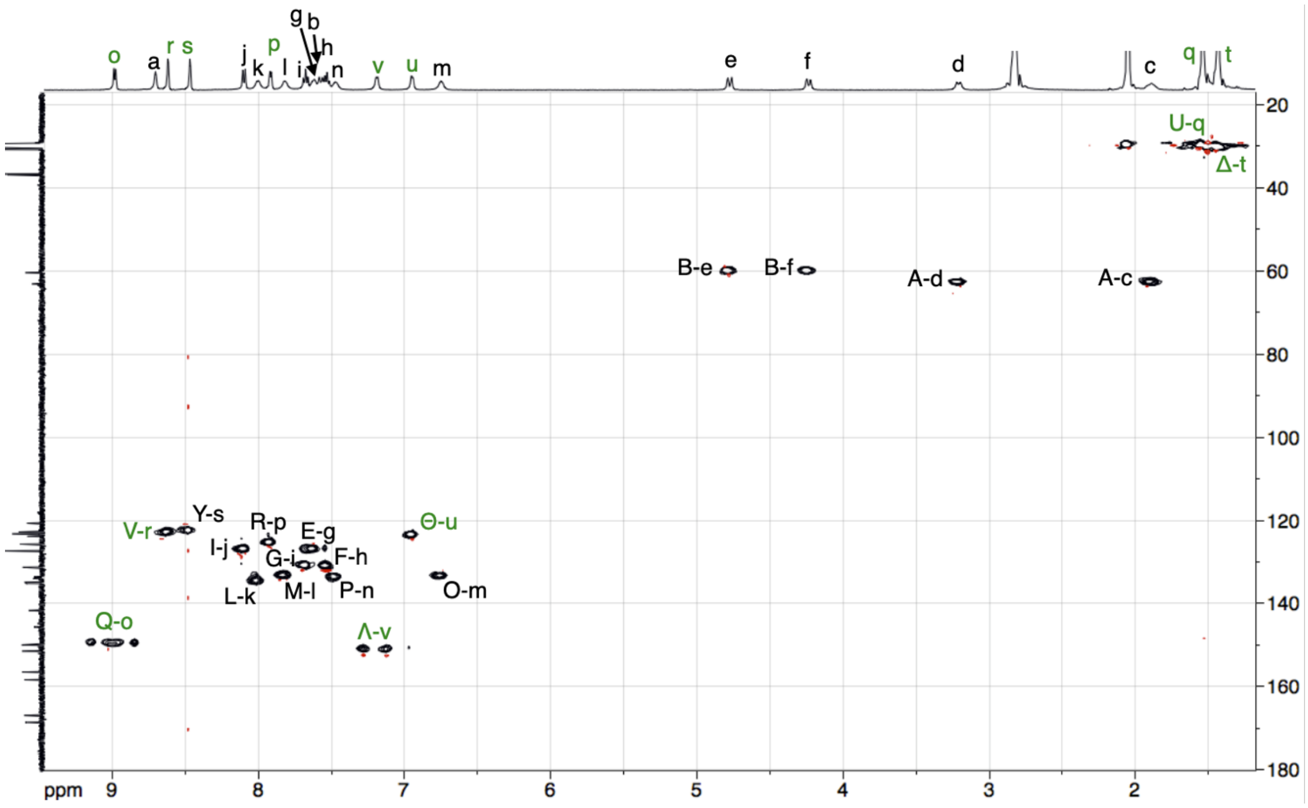

Supplementary Fig. 23 ^{1}H-^{13}C HSQC NMR spectrum of $\mathbf{1}_{\mathbf{loose}}$ (500 MHz for ^{1}H and 126 MHz for ^{13}C, acetone-d_6, 300 K).

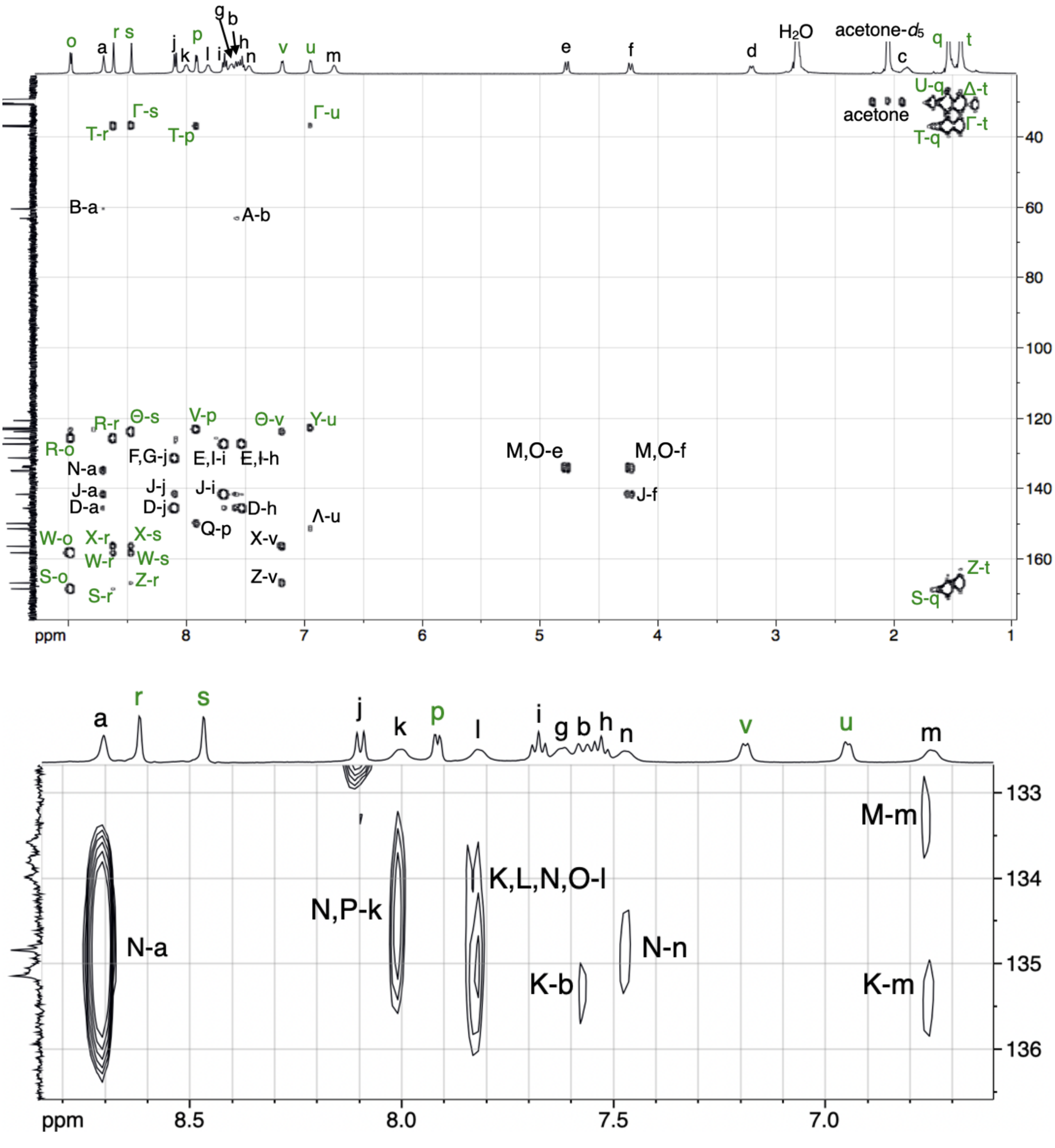

Supplementary Fig. 24 ^{1}H-^{13}C HMBC NMR spectra of $\mathbf{1}_{\mathbf{loose}}$ (500 MHz for ^{1}H and 126 MHz for ^{13}C, acetone-d_6, 300 K); (upper) overall spectrum and (lower) enlarged and amplified spectrum.

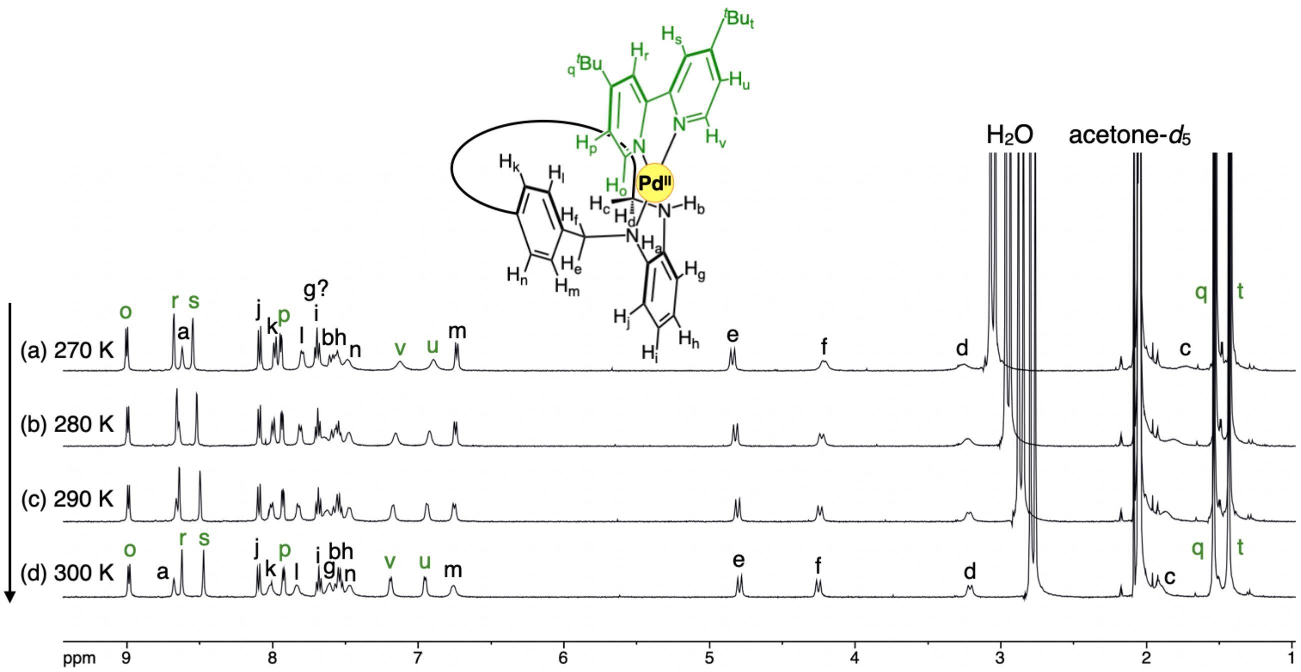

Supplementary Fig. 25 VT ^{1}H NMR spectra of **1**$_{\text{loose}}$ (500 MHz, acetone-d_6) (a) at 270 K, (b) 280 K, (c) 290 K and (d) at 300 K.

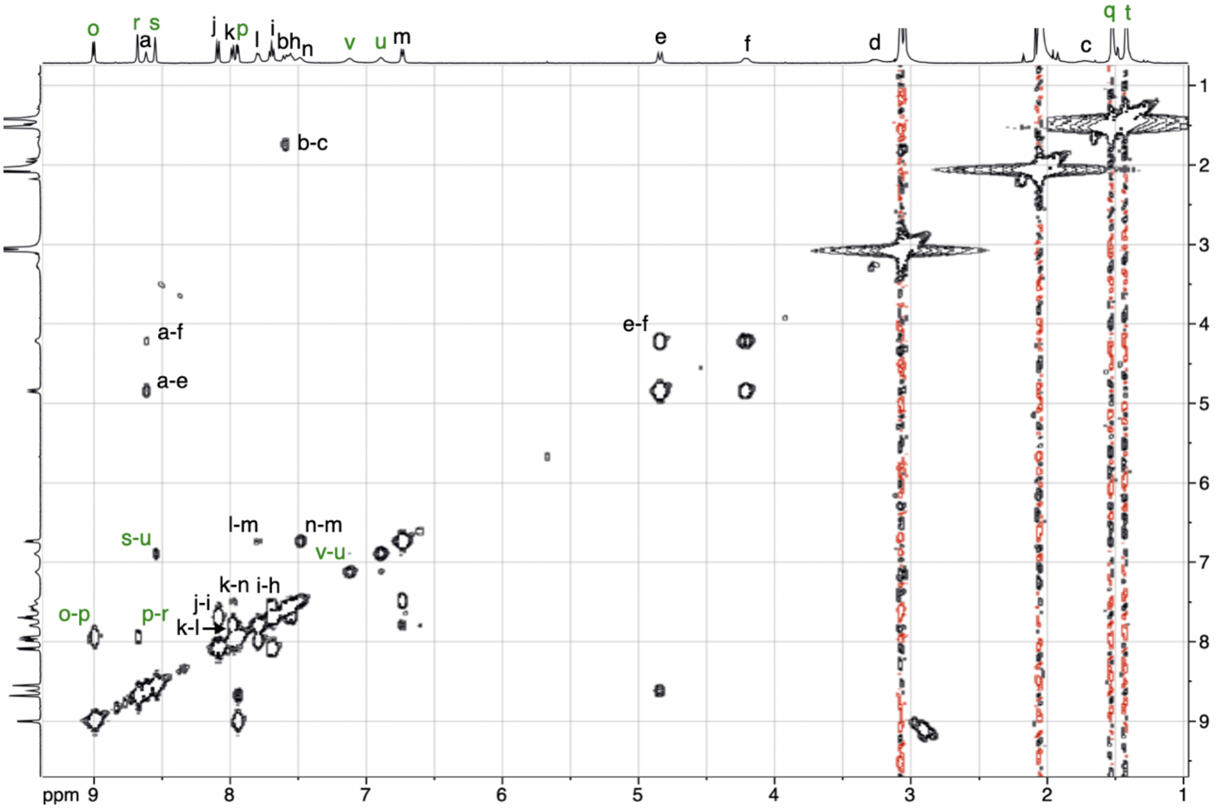

Supplementary Fig. 26 ^{1}H-^{1}H COSY NMR spectrum of **1**$_{\text{loose}}$ (500 MHz, acetone-d_6, 270 K).

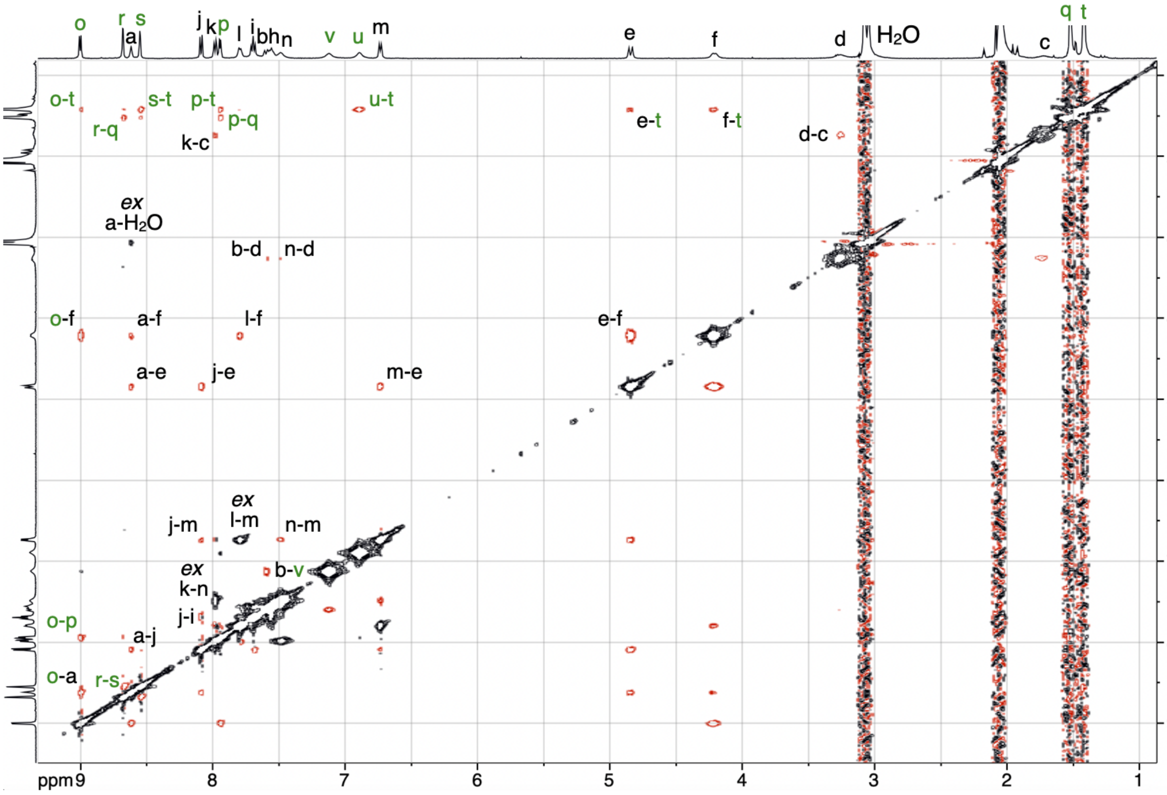

Supplementary Fig. 27 ^1H-^1H ROESY NMR spectrum of $\mathbf{1}_{\mathbf{loose}}$ (500 MHz, acetone-d_6, 270 K).

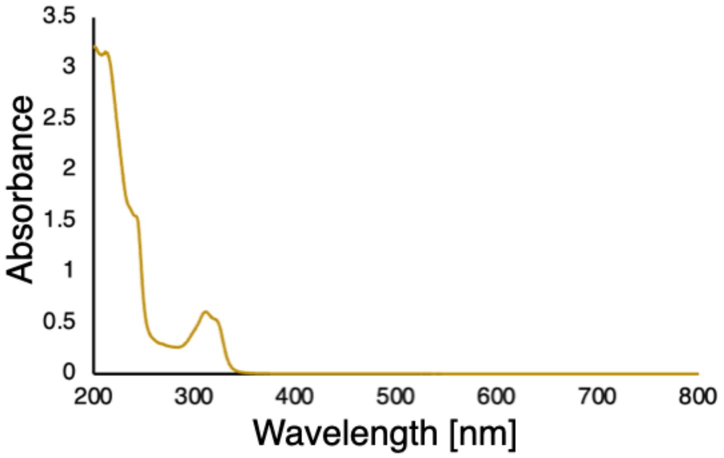

Supplementary Fig. 28 UV-vis absorption spectrum of $\mathbf{1}_{\mathbf{loose}}$ (82.3 μM, l = 0.2 cm, 293 K in MeCN).

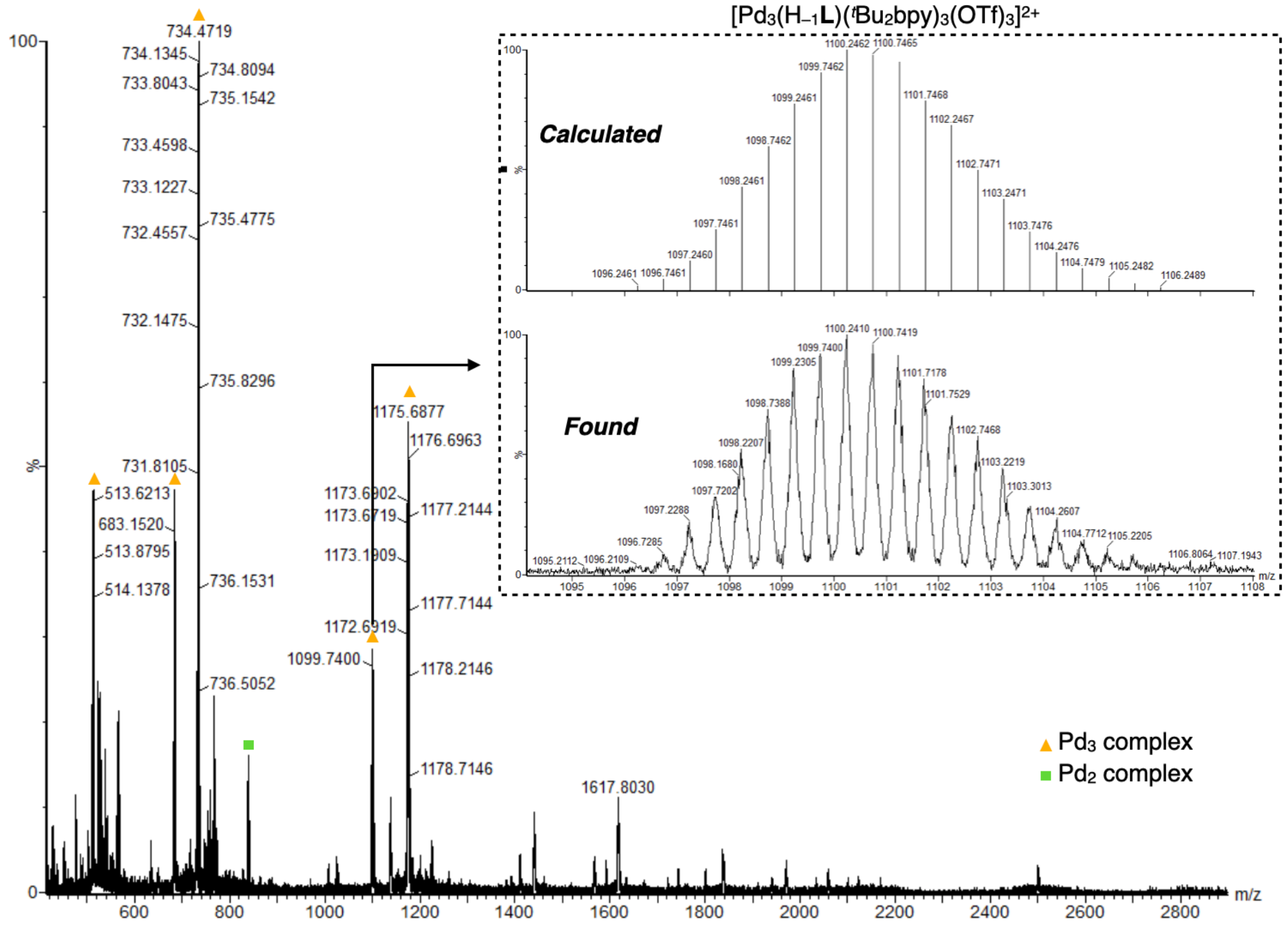

Supplementary Fig. 29 High resolution-ESI-TOF mass spectrum of $\mathbf{1}_{\mathbf{loose}}$ (positive, acetone).

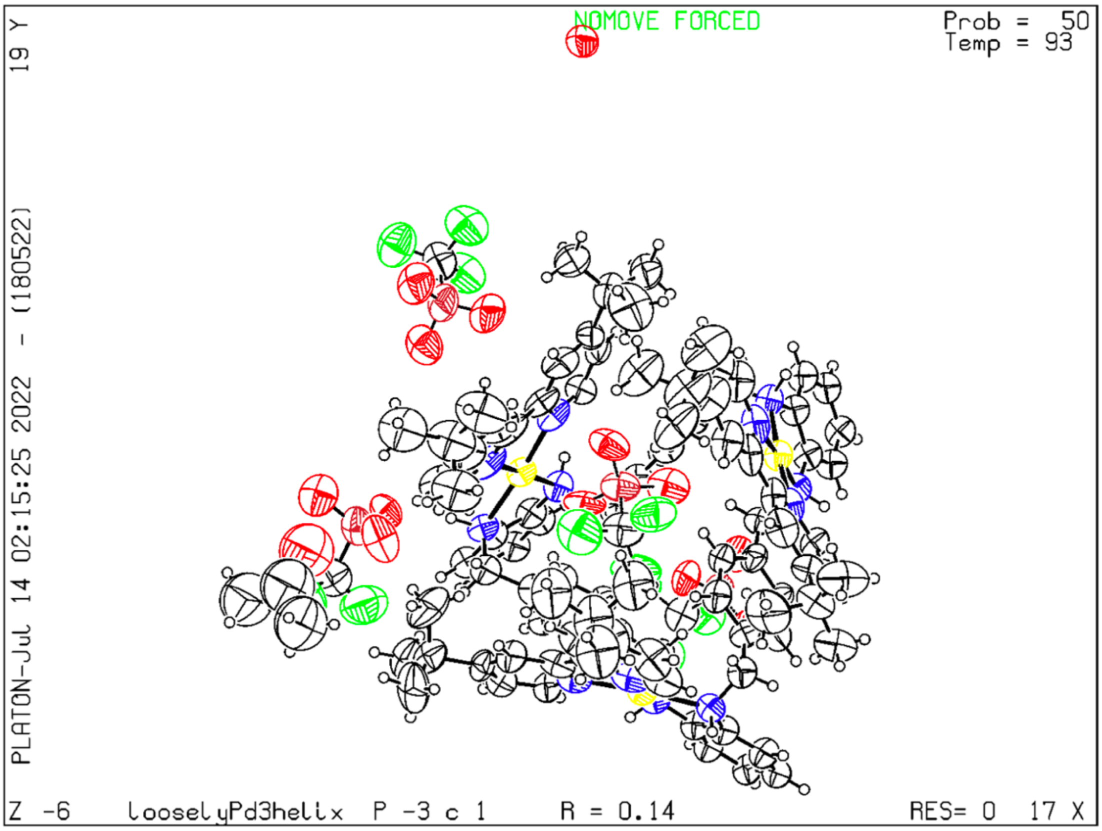

Supplementary Fig. 30 ORTEP drawing of $\mathbf{1}_{loose}$ at the 50% probability level. Colour: C black, N blue, O red, F yellow green, S dark red and Pd yellow. CCDC deposit number of $\mathbf{1}_{loose}$ is 2190129. This figure was produced by the checkCIF report of the International Union of Crystallography.

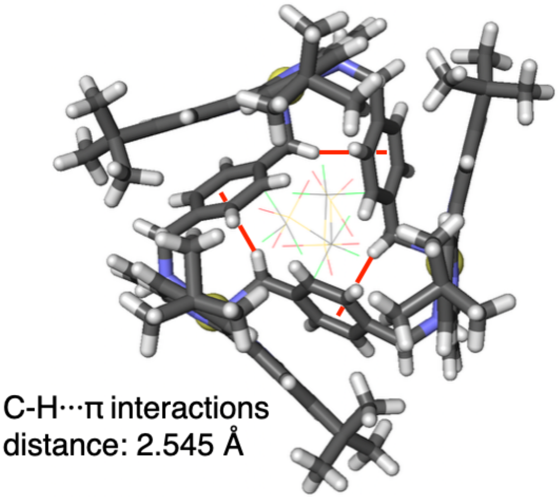

Supplementary Fig. 31 Crystal structure of $\mathbf{1}_{loose}$ with the distance between H atom and π plane.

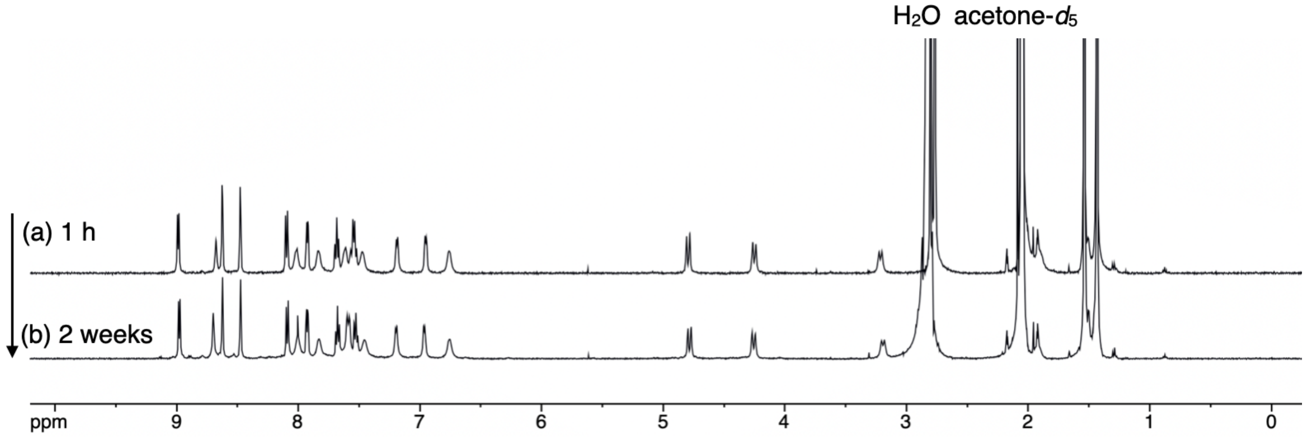

Supplementary Fig. 32 ^{1}H NMR spectra of $\mathbf{1}_{\mathbf{loose}}$ (500 MHz, acetone-*d*$_6$, 300 K) (a) 1 h and (b) 2 weeks at room temperature after dissolved in acetone-*d*$_6$.

2.4 Definition of the two twisted isomers of 1

The two twisted isomers of **1** are defined by the degree to which the *ortho*-phenylenediamine moieties are folded inside the macrocycle. The degree of folding is evaluated by the dihedral angle between the plane horizontal to the macrocyclic skeleton and the plane of the *ortho*-phenylenediamine ring. The dihedral angle in **L** before metal coordination is nearly 0°, indicating no twisting. One isomer of the twisted Pd^{II}_3-macrocycles with a dihedral angle of greater than 90° is defined as the tightly-twisted isomer, $\mathbf{1}_{\mathbf{tight}}$. In contrast, the other isomer with a dihedral angle of less than 90° is therefore defined as the loosely-twisted isomer, $\mathbf{1}_{\mathbf{loose}}$. As a result of the different twisting modes, the absolute configuration of the amine nitrogen atoms changes through metal coordination to (*all*-*R* or *all*-*S*) and (*alt*-*R*/*S*) for the tightly- and loosely-twisted isomers, respectively. Note that this definition differs from the typical definition of a helix, which is based on differences in helical pitch. This is because the structure of **1** is not a helix, but a twist with the *P*- and *M*-helicity.

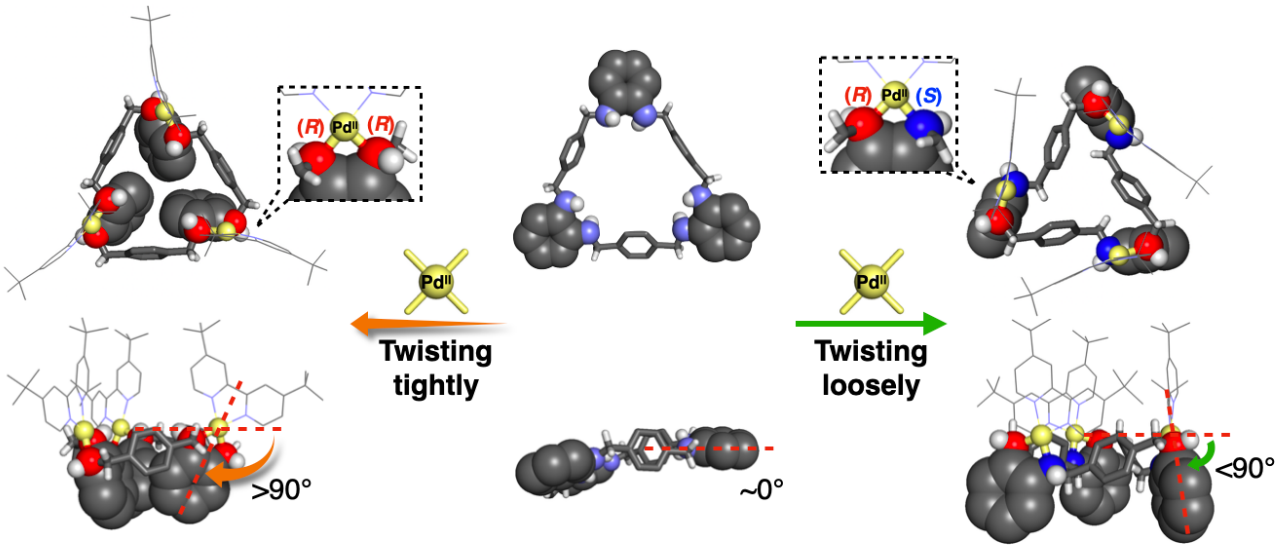

Supplementary Fig. 33 Illustration supporting the definition of the twisted isomers: (left) **L** is twisted tightly to form (*P*)-$\mathbf{1}_{\mathbf{tight}}$ forming the (*all*-*R*) configuration with *ortho*-phenylenediamine nitrogen atoms, and (right) **L** is twisted loosely to form (*P*)-$\mathbf{1}_{\mathbf{loose}}$ with the (*alt*-*R*/*S*) configuration with *ortho*-phenylenediamine nitrogen atoms.

2.5 Characterisation of 2

Characterisation of an intermediate species **2** obtained in the synthesis of $\mathbf{1}_{\mathbf{loose}}$ is shown below.
^{1}H NMR (500 MHz, acetone-d_6, 300 K): δ 9.12 (d, J = 6.0 Hz, 2H), 8.90 (s, 2H), 8.89 (s, 2H), 8.84 (d, J = 5.5 Hz, 2H), 8.79 (s, 2H), 8.27 (d, J = 10.0 Hz, 2H), 8.20 (d, J = 5.5 Hz, 2H), 8.10 (m, 4H), 7.66 (m, 6H), 7.26 (t, J = 7.5Hz, 2H), 6.25 (d, J = 7.5 Hz, 2H), 5.94 (m, 2H), 5.63 (m, 2H), 4.93 (d, J = 5.5 Hz, 2H), 4.91 (d, J = 14.5 Hz, 2H), 4.66 (d, J = 14.0 Hz, 2H), 4.46 (dd, J = 16.0, 6.0 Hz, 2H), 4.19 (d, J = 13.5 Hz, 2H), 4.01 (dd, J = 16.0, 5.0 Hz, 2H), 1.66 (t, J = 12.5 Hz, 2H), 1.53 (s, 36H).
HRMS (ESI-TOF): m/z = 689.2654 as $[Pd_2(H_{-2}\mathbf{L})(^t Bu_2 bpy)_2]^{2+}$ (calcd 689.2650). Assignment of H_c and H_h in the ^{1}H NMR spectrum was completed by 2D NMR analysis and H-D exchange of amine protons by adding D_2O into the acetone-d_6 solution of **2**. ^{13}C NMR of **2** was not measured in acetone-d_6 due to the low solubility and slow decomposition of **2**.

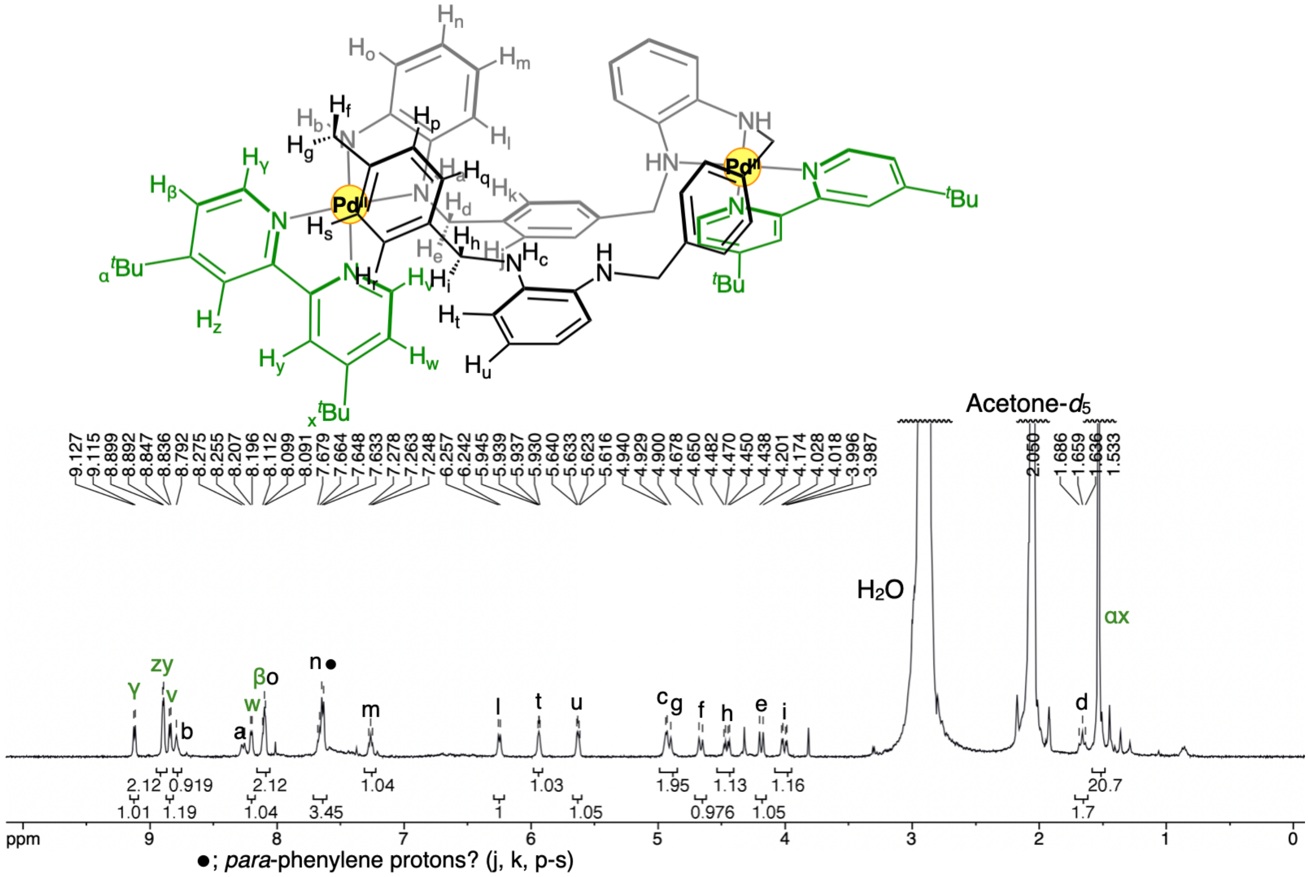

Supplementary Fig. 34 ^{1}H NMR spectrum of as-synthesised **2** (500 MHz, acetone-d_6, 300 K). The peaks of p-phenylene groups (j, k, p, q, r, s) highly broaden due to their rotational motion.

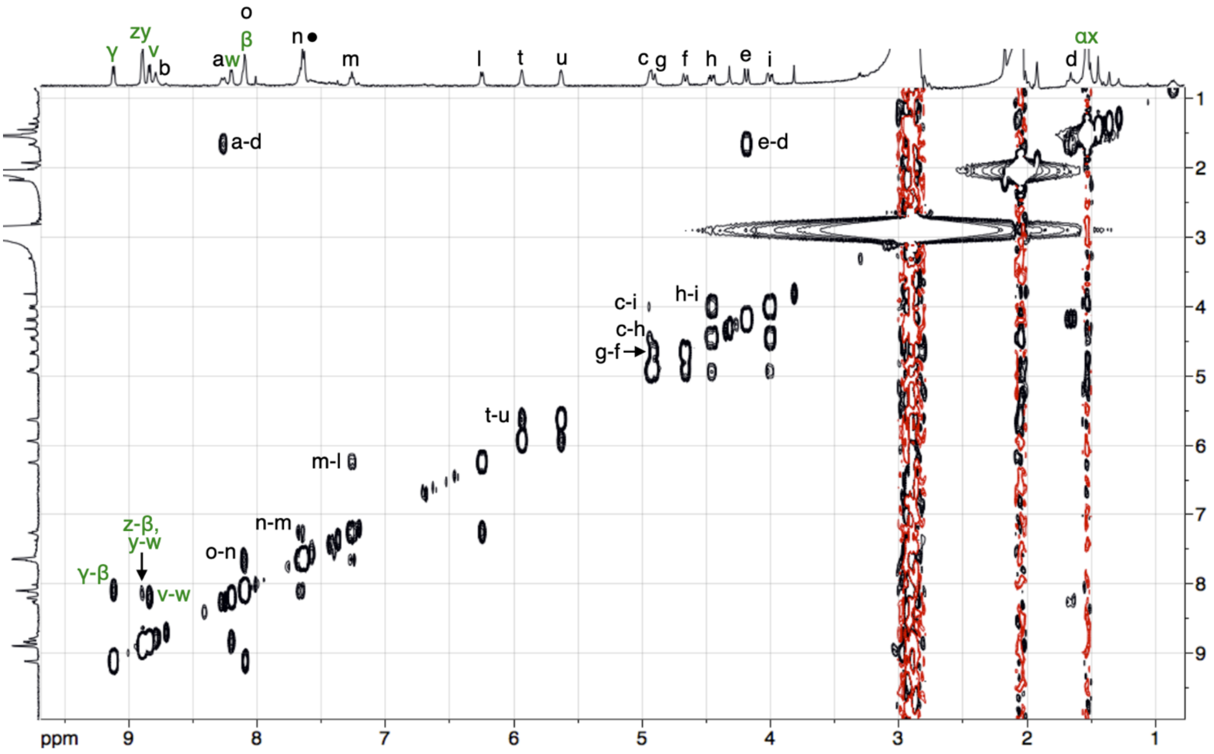

Supplementary Fig. 35 ^1H-^1H COSY NMR spectrum of as-synthesised **2** (500 MHz, acetone-d_6, 300 K).

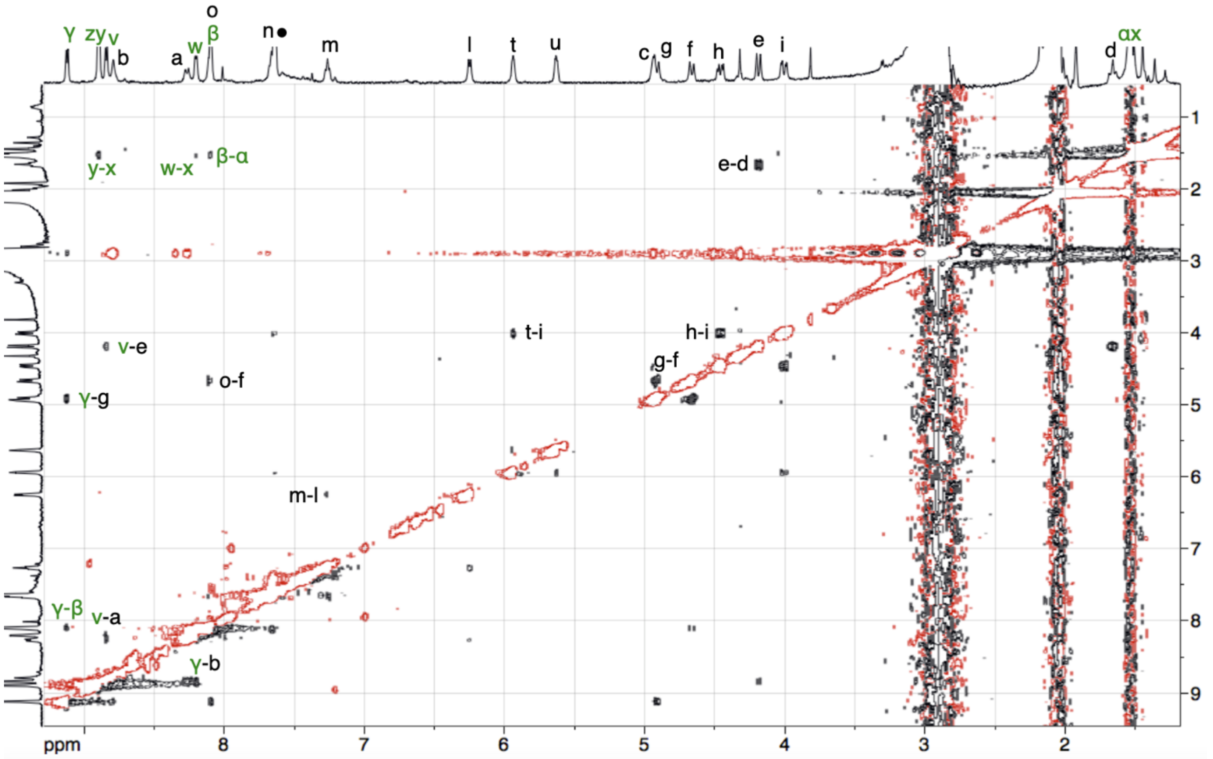

Supplementary Fig. 36 ^1H-^1H ROESY NMR spectrum of as-synthesised **2** (500 MHz, acetone-d_6, 300 K).

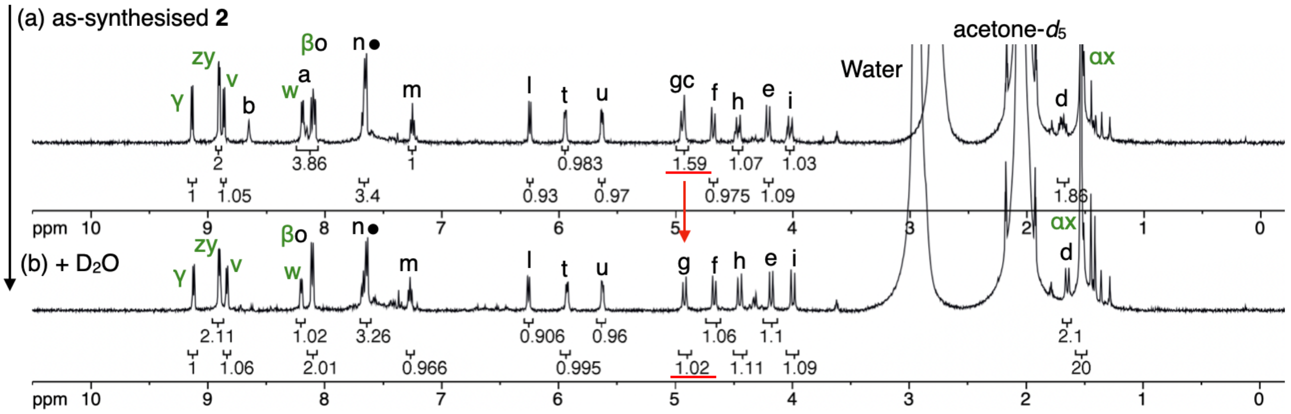

Supplementary Fig. 37 ^{1}H NMR spectra (500 MHz, acetone-d_6, 300 K) of (a) as-synthesised **2** and (b) as-synthesised **2** with D_2O.

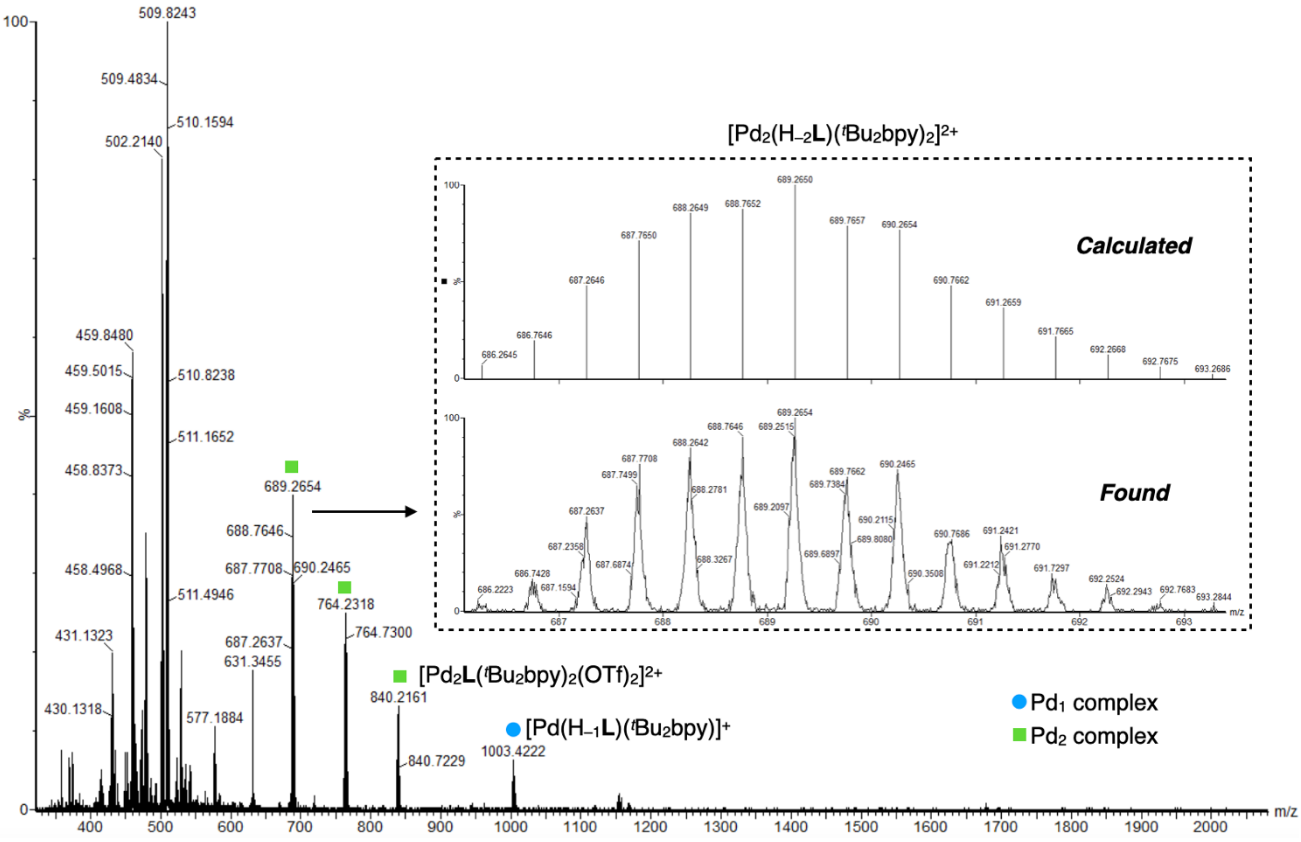

Supplementary Fig. 38 High resolution-ESI-TOF mass spectrum of **2** (positive, acetone).

2.6 Synthesis of [Pt(^{t}Bu$_2$bpy)(OH$_2$)$_2$](OTf)$_2$[7,8]

Water (3.8 mL) and HCl aq. (4 M, 0.2 mL) were added to K_2PtCl_4 (49.9 mg, 120 µmol, 1.0 equiv.) and ^{t}Bu$_2$bpy (32.3 mg, 120 µmol, 1.0 equiv.) placed in a 2-necked flask, and then the mixture was heated at reflux for 2 h. During the reaction, a yellow precipitate was formed. The yellow solid was collected by filtration and washed with water, MeOH and Et_2O in this order. The resulting solid was dried up to afford [PtCl$_2$(^{t}Bu$_2$bpy)] (57.3 mg, 107 µmol, 89%) as a yellow solid, which was then dissolved in CH_2Cl_2 (6.5 mL). To this solution was added AgOTf (58.4 mg, 227 µmol, 2.1 equiv.). The reaction solution was stirred at room temperature for 1 h. After removal of precipitated Ag salts by filtration, the filtrate was evaporated. The resulting pale yellow solid was dried under reduced pressure to afford [Pt(^{t}Bu$_2$bpy)(OH$_2$)$_2$](OTf)$_2$ (83.7 mg, 105 µmol, 87% in total) as a pale yellow solid. ^{1}H NMR (500 MHz, CDCl$_3$, 300 K): δ 8.57 (d, J = 6.0 Hz, 2H), 8.46 (brs, 4H), 7.91 (d, J = 1.5 Hz, 2H), 7.71 (dd, J = 6.0, 2.0 Hz, 2H), 1.50 (s, 18H). ^{13}C NMR (126 MHz, acetone-d_6, 300 K): δ 149.0, 125.2, 100.1, 36.6, 30.4, 30.2. Some of ^{13}C NMR signals were not observed due to the low solubility in CDCl$_3$.

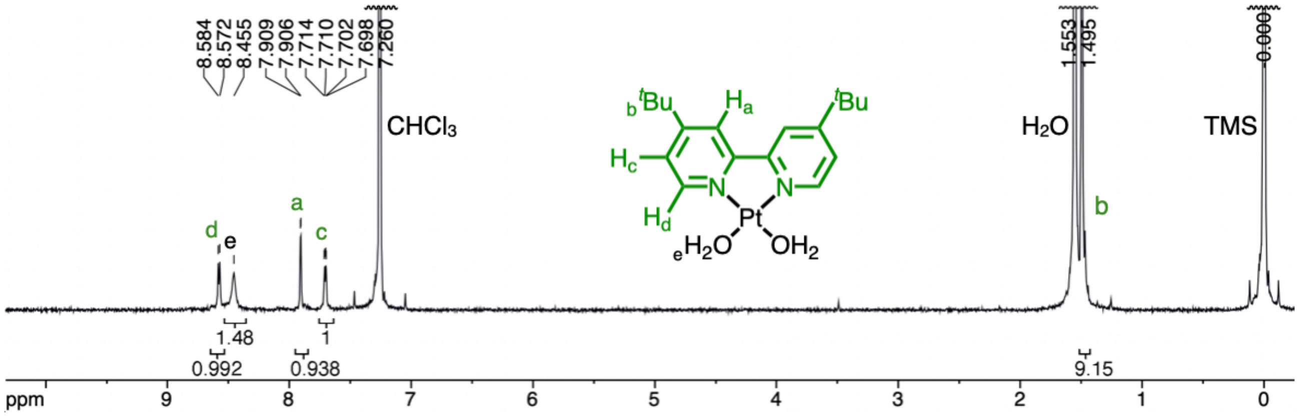

Supplementary Fig. 39 ^{1}H NMR spectrum of [Pt(^{t}Bu$_2$bpy)(OH$_2$)$_2$](OTf)$_2$ (500 MHz, 300 K, CDCl$_3$).

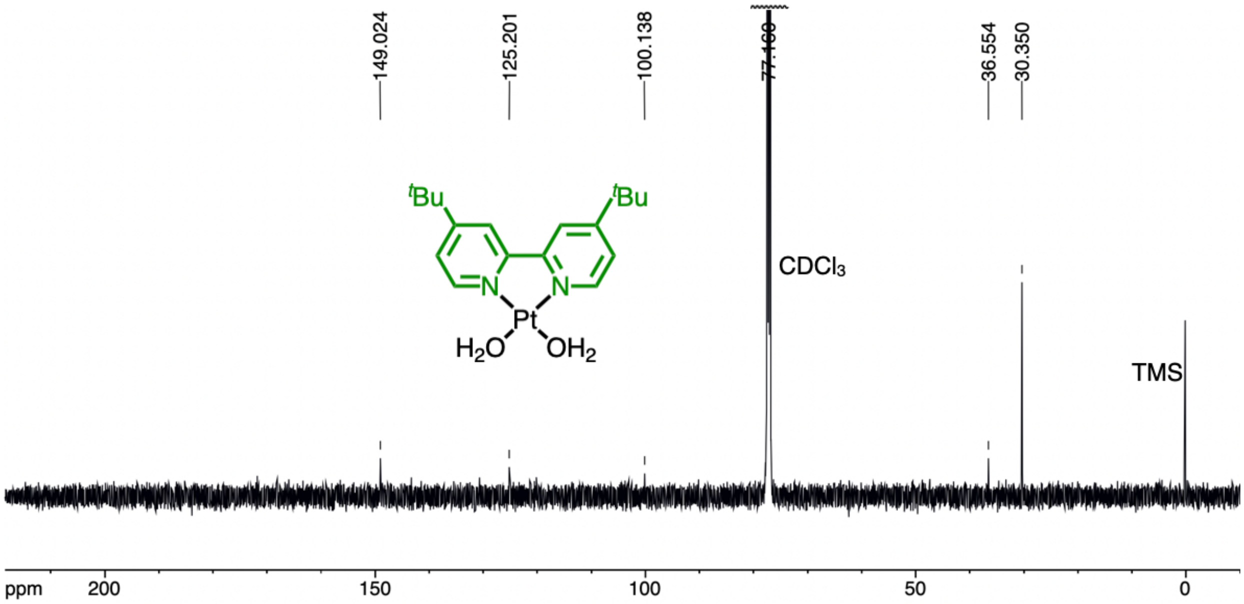

Supplementary Fig. 40 ^{13}C NMR spectrum of [Pt(^{t}Bu$_2$bpy)(OH$_2$)$_2$](OTf)$_2$ (126 MHz, 300 K, CDCl$_3$).

2.7 Synthesis and characterisation of [Pt$_2$L(^{t}Bu$_2$bpy)$_2$](OTf)$_4$

A dinuclear Pt^{II} complex, [Pt$_2$**L**(^{t}Bu$_2$bpy)$_2$](OTf)$_4$, was synthesised by the reaction of **L** with the Pt^{II} salt, [Pt(^{t}Bu$_2$bpy)(OH$_2$)$_2$](OTf)$_2$ similarly to the synthesis of **2**. A $CHCl_3$ solution (2.0 mL) of **L** (29.8 mg, 47.2 µmol, 1.0 equiv.) was mixed with a $CHCl_3$ solution (10.0 mL) of [Pt(^{t}Bu$_2$bpy)(OH$_2$)$_2$](OTf)$_2$ (68.4 mg, 85.8 µmol, 1.8 equiv.), and then stirred at 45 °C for 21.5 h. During the reaction, dark pink precipitate was formed. The resulting precipitate was collected by filtration and washed with $CHCl_3$ to afford [Pt$_2$**L**(^{t}Bu$_2$bpy)$_2$](OTf)$_4$ as a dark pink solid (46.98 mg, 21.8 µmol, 51%).

^{1}H NMR (500 MHz, $CDCl_3$, 300 K): δ 9.52 (s, 2H), 9.39 (d, J = 6.0 Hz, 2H), 9.12 (d, J = 6.5 Hz, 2H), 8.93 (s, 6H), 8.24 (dd, J = 6.0, 2.0 Hz, 2H), 8.18 (d, J = 8.0 Hz, 2H), 8.11 (dd, J = 6.0 1.5 Hz, 2H), 7.68 (t, J = 7.5 Hz, 2H), 7.59 (d, J = 7.5 Hz, 4H), 7.27 (t, J = 8.0 Hz, 2H), 6.34 (d, J = 8.0 Hz, 2H), 5.93 (m, 2H), 5.63 (m, 2H), 5.07 (d, J = 13.0 Hz, 2H), 4.93 (d, J = 12.0 Hz, 4H), 4.44 (dd, J = 16.0, 6.5 Hz, 2H), 4.33 (d, J = 13.5 Hz, 2H), 4.00 (dd, J = 15.5, 6.0 Hz, 2H), 1.65 (m, 2H), 1.55 (s, 36H), HRMS (ESI-TOF): m/z = 852.8032 as [Pt$_2$(H$_{-1}$**L**)(^{t}Bu$_2$bpy)$_2$(OTf)]$^{2+}$ (calcd 852.8052).

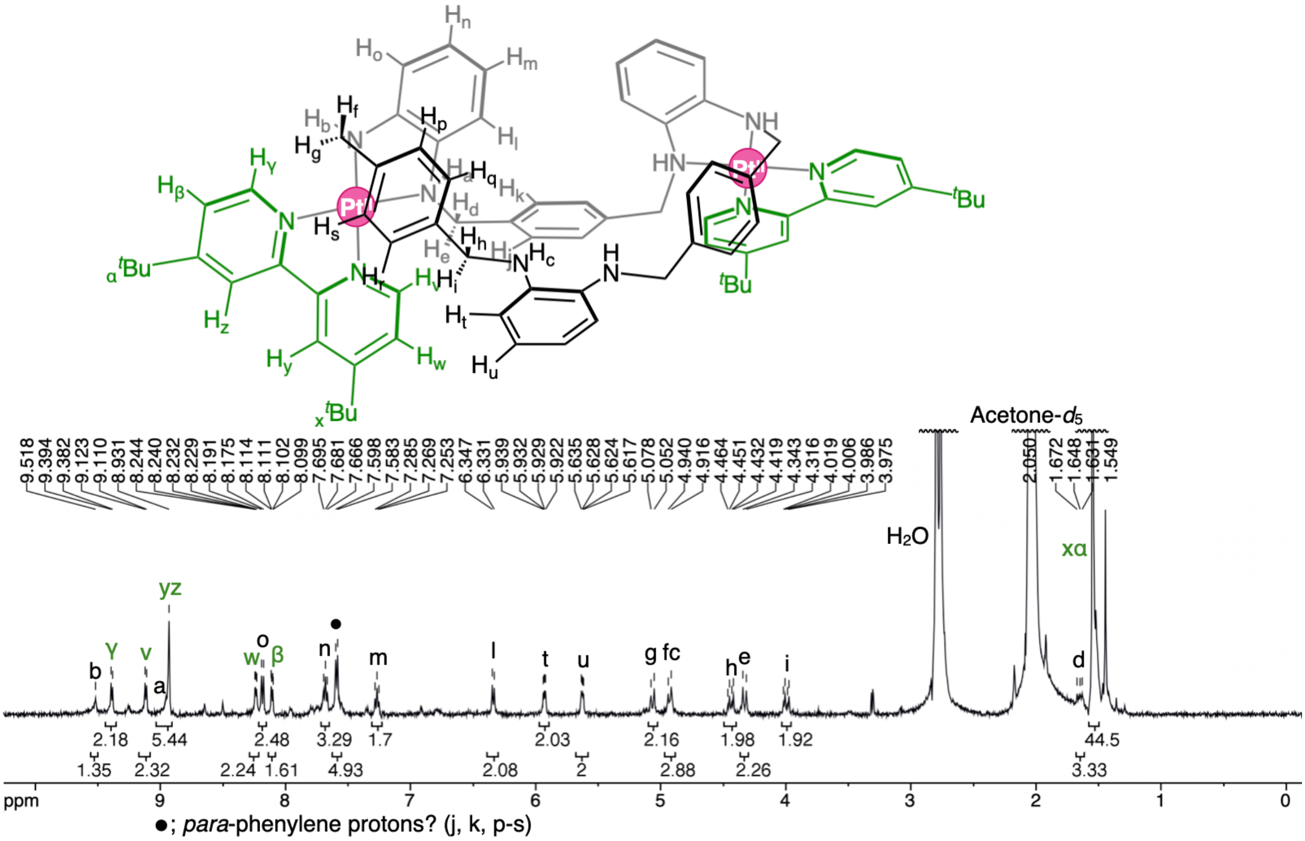

Supplementary Fig. 41 ^{1}H NMR spectrum of as-synthesised [Pt$_2$**L**(^{t}Bu$_2$bpy)$_2$](OTf)$_4$ (500 MHz, acetone-d_6, 300 K). The signals of p-phenylene groups (j, k, p, q, r, s) were highly broadened due to their rotational motion.

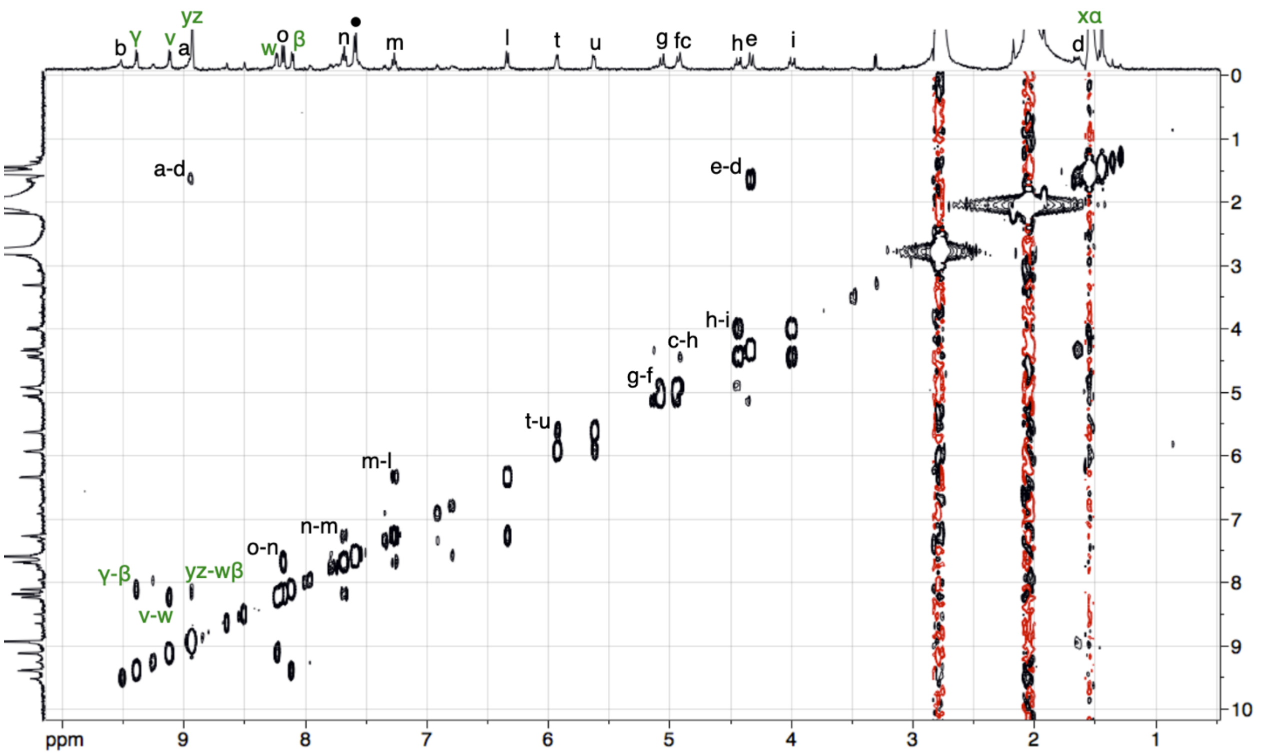

Supplementary Fig. 42 ^{1}H-^{1}H COSY NMR spectrum of as-synthesised $[Pt_2\mathbf{L}(^t\mathrm{Bu}_2\mathrm{bpy})_2](OTf)_4$ (500 MHz, acetone-d_6, 300 K).

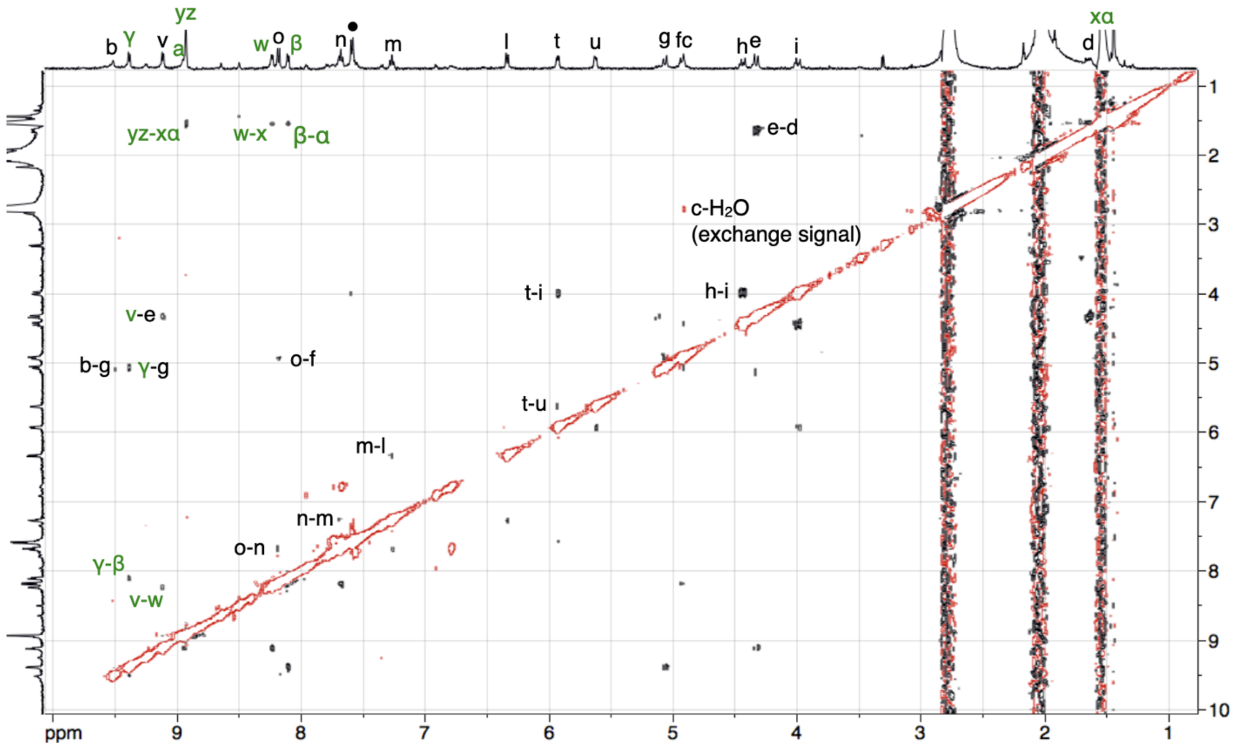

Supplementary Fig. 43 ^{1}H-^{1}H ROESY NMR spectrum of as-synthesised $[Pt_2\mathbf{L}(^t\mathrm{Bu}_2\mathrm{bpy})_2](OTf)_4$ (500 MHz, acetone-d_6, 300 K).

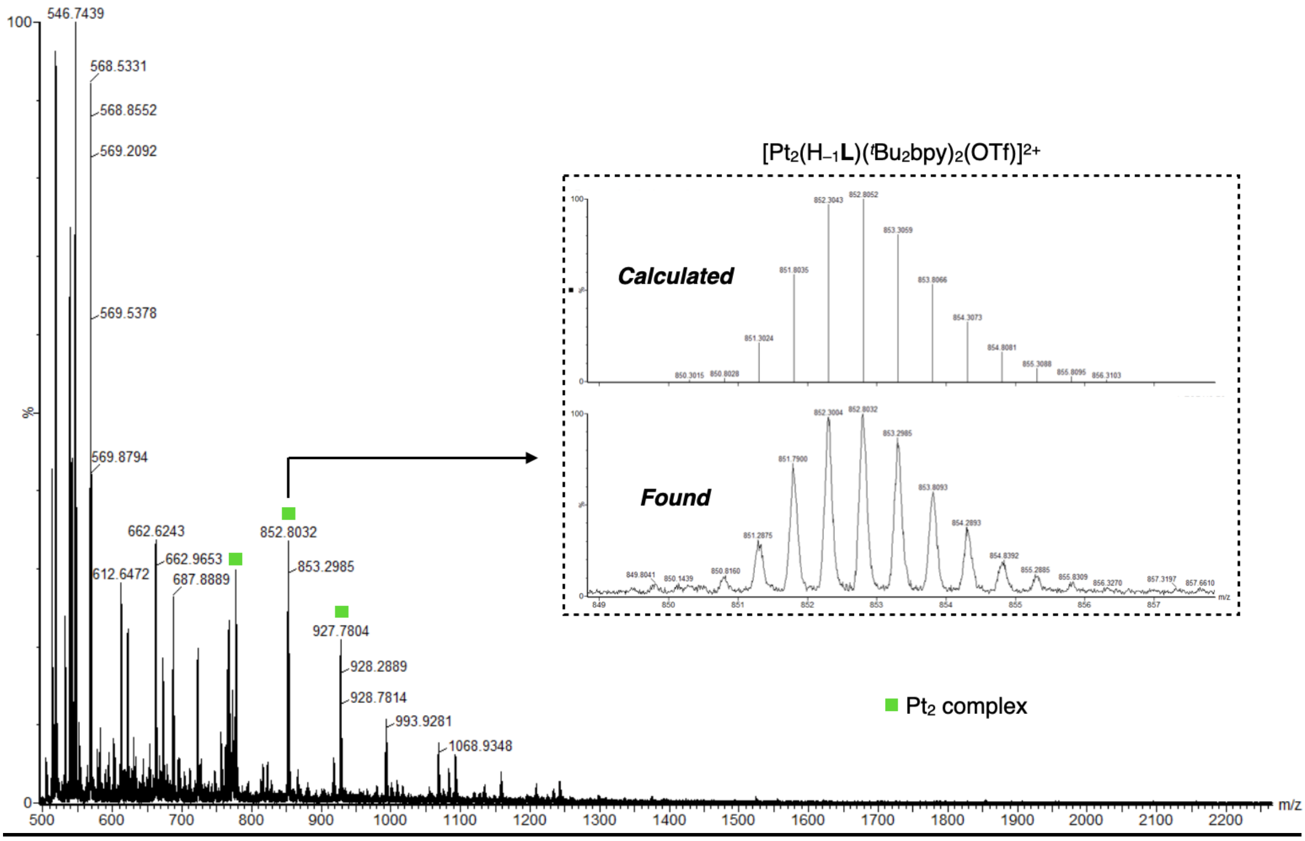

Supplementary Fig. 44 High resolution-ESI-TOF mass spectrum of [Pt_2**L**(^{t}Bu_2bpy)$_2$](OTf)$_4$ (positive, MeCN).

Single-crystal XRD analysis was performed on a crystal separately prepared by the following procedure. A $CHCl_3$ solution (0.25 mL) of **L** (1.48 mg, 2.34 µmol, 1.3 equiv.) was mixed with a $CHCl_3$ solution (0.5 mL) of [Pt(^{t}Bu_2bpy)(OH_2)$_2$](OTf)$_2$ (2.85 mg, 3.57 µmol, 2.0 equiv.), and the reaction solution was allowed to stand at room temperature for 2 weeks to afford [Pt_2**L**(^{t}Bu_2bpy)$_2$](OTf)$_4$ as red crystals, which were analysed by single-crystal XRD. The surface of this crystal was coloured red because the surface might be partially oxidised.

Crystal data for Pt_2**L**(^{t}Bu_2bpy)$_2$·(OTf)$_4$·(H_2O)$_{1.75}$·($CHCl_3$)$_{0.5}$: $C_{82.50}H_{90.50}Cl_{1.50}F_{12}N_{10}O_{13.75}Pt_2S_4$, F_w = 2241.74, crystal dimensions 0.142 × 0.08 × 0.069 mm^3, triclinic, space group *P*-1, a = 11.6403(6), b = 16.7015(6), c = 29.8560(12) Å, α = 74.874(3), β = 80.040(4), γ = 82.830(4)°, V = 5499.4(4) Å^3, Z = 2, ρ_{calcd} = 1.354 g cm^{-3}, μ = 63.79 cm^{-1}, T = 93 K, λ(CuKα) = 1.54187 Å, $2\theta_{max}$ = 145.734°, 50139/20687 reflections collected/unique (R_{int} = 0.0939), R_1 = 0.1150 ($I > 2\sigma(I)$), wR_2 = 0.3875 (for all data), GOF = 1.146, largest diff. peak and hole 1.904/–2.056 eÅ^{-3}. CCDC deposit number 2190132.

PLAT084_ALERT_3_B High wR2 Value (i.e. > 0.25) … 0.39

Response: The average intensity of reflections is relatively low due to the small size of the crystal.

PLAT242_ALERT_2_B Low 'MainMol' Ueq as Compared to Neighbors of C71

Response: This problem is caused by the large Ueq of the neighboring methyl groups in the tert-butyl substituent and inherently difficult to avoid.

PLAT306_ALERT_2_B Isolated Oxygen Atom (H-Missing?)

Response: Hydrogen atoms of water molecules could not be located in the difference electron density maps.

PLAT 342_ALERT_3_B Low Bond Precision on C-C Bonds … 0.03184 Ang.

Response: The average intensity of reflections is relatively low due to the small size of the crystal.

PLAT430_ALERT_2_B Short Inter D…A Contact

Response: The short contacts come from H_2O…OTf, though hydrogen atoms of the water molecule could not be located in the difference electron density maps.

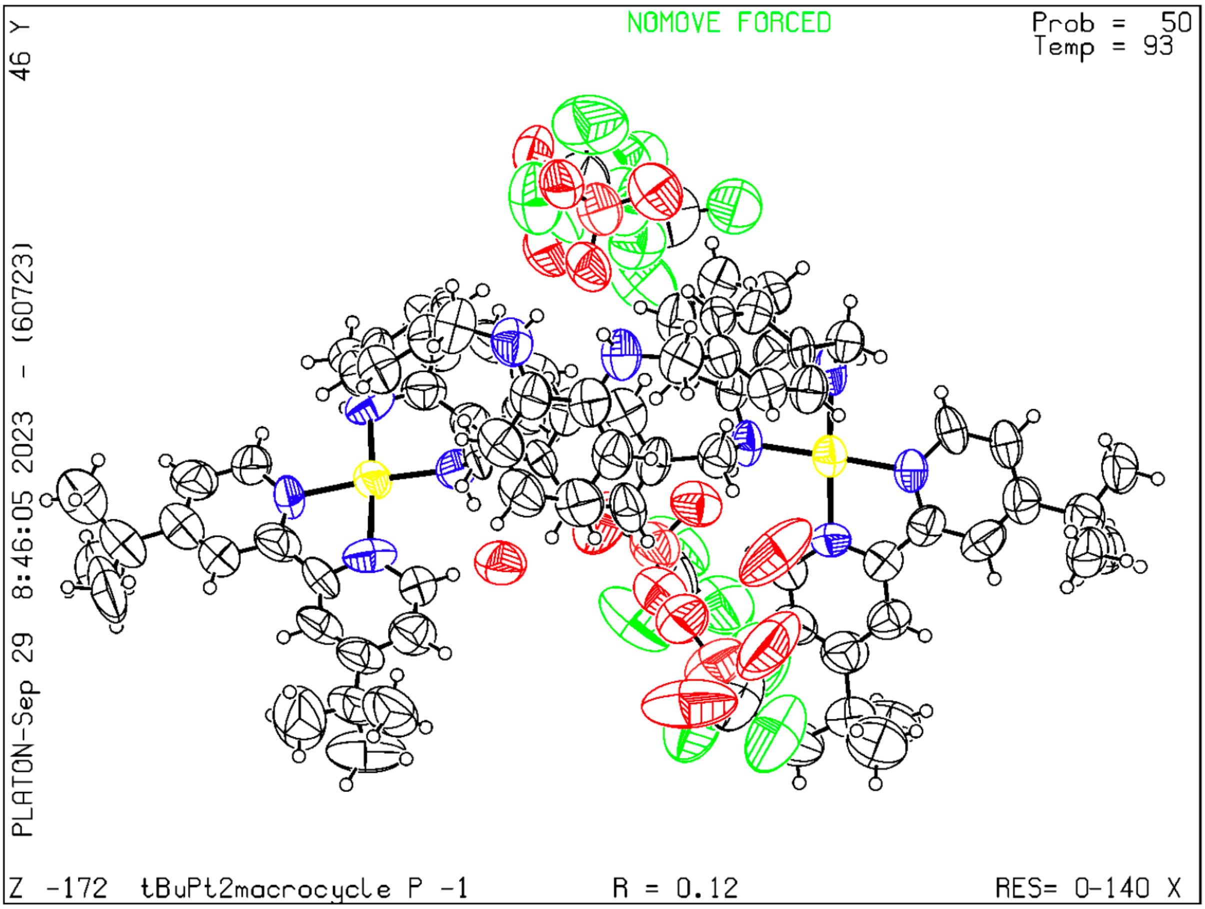

Supplementary Fig. 45 ORTEP drawing of [Pt$_2$**L**(^{t}Bu$_2$bpy)$_2$](OTf)$_4$ at the 50% probability level. Colour: C black, N blue, O red, F yellow green, S dark red and Pt yellow. CCDC deposit number of [Pt$_2$**L**(^{t}Bu$_2$bpy)$_2$](OTf)$_4$ is 2190132. This figure was produced by the checkCIF report of the International Union of Crystallography.

We have attempted to synthesise the Pt-analogue of **1**$_{\mathbf{loose}}$ and **1**$_{\mathbf{tight}}$ in a similar fashion for comparison, but have not yet succeeded in synthesising the Pt-analogue of **1**$_{\mathbf{tight}}$, probably due to the inherent difficulties associated with the kinetic process. Therefore, it is difficult at this time to discuss the differences in structure and dynamics of the Pd- and Pt-complexes of both twisted isomers due to the synthetic limitations.

3. Isomerisation from $\mathbf{1}_{\mathbf{tight}}$ to $\mathbf{1}_{\mathbf{loose}}$

3.1 Theoretical analysis of the rate constant of isomerisation from $\mathbf{1}_{\mathbf{tight}}$ to $\mathbf{1}_{\mathbf{loose}}$

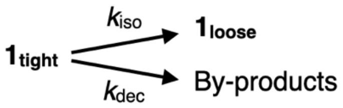

Supplementary Fig. 46 Scheme of isomerisation from $\mathbf{1}_{\mathbf{tight}}$ to $\mathbf{1}_{\mathbf{loose}}$ and decomposition to by-products.

We considered isomerisation reaction from $\mathbf{1}_{\mathbf{tight}}$ to $\mathbf{1}_{\mathbf{loose}}$ and decomposition reaction as pseudo first order reactions as shown in the scheme above. In this case, the differential equations are given below:

$$\frac{d}{dt}[\mathbf{1}_{\mathbf{tight}}] = -k_{iso}[\mathbf{1}_{\mathbf{tight}}] - k_{dec}[\mathbf{1}_{\mathbf{tight}}] \quad \cdots(3\text{-}1)$$

$$\frac{d}{dt}[\mathbf{1}_{\mathbf{loose}}] = k_{iso}[\mathbf{1}_{\mathbf{tight}}] \quad \cdots(3\text{-}2)$$

$$\frac{d}{dt}[\mathrm{B}] = k_{dec}[\mathbf{1}_{\mathbf{tight}}]$$

where $[\mathbf{1}_{\mathbf{tight}}]$, $[\mathbf{1}_{\mathbf{loose}}]$ and [B] are the concentrations of $\mathbf{1}_{\mathbf{tight}}$, $\mathbf{1}_{\mathbf{loose}}$ and by-product, respectively, and k_{iso} and k_{dec} are the rate constants of isomerisation from $\mathbf{1}_{\mathbf{tight}}$ to $\mathbf{1}_{\mathbf{loose}}$ and decomposition of $\mathbf{1}_{\mathbf{tight}}$, respectively. After rearrangement, the differential equation of equation (3-1) is solved:

$$\frac{d}{dt}[\mathbf{1}_{\mathbf{tight}}] = -(k_{iso} + k_{dec})[\mathbf{1}_{\mathbf{tight}}]$$

$$[\mathbf{1}_{\mathbf{tight}}] = [\mathbf{1}_{\mathbf{tight}}]_0 \cdot e^{-(k_{iso}+k_{dec})t} \quad \cdots(3\text{-}3)$$

where $[\mathbf{1}_{\mathbf{tight}}]_0$ is the initial concentration of $\mathbf{1}_{\mathbf{tight}}$. Then, the equation (3-3) is substituted into the equation (3-2) and a new differential equation of $[\mathbf{1}_{\mathbf{loose}}]$ is given and solved.

$$\frac{d}{dt}[\mathbf{1}_{\mathbf{loose}}] = k_{iso}[\mathbf{1}_{\mathbf{tight}}]_0 \cdot e^{-(k_{iso}+k_{dec})t}$$

$$[\mathbf{1}_{\mathbf{loose}}] = \frac{k_{iso}}{k_{iso}+k_{dec}}[\mathbf{1}_{\mathbf{tight}}]_0 \cdot (1 - e^{-(k_{iso}+k_{dec})t}) \quad \cdots(3\text{-}4)$$

The rate constant of isomerisation from $\mathbf{1}_{\mathbf{tight}}$ to $\mathbf{1}_{\mathbf{loose}}$ was estimated by curve fitting analysis of time course ^{1}H NMR spectroscopy based on equations (3-3) and (3-4). The fitting analysis and estimation of the standard error were conducted using KaleidaGraph software.

3.2 Time course ^{1}H NMR measurement to estimate the isomerisation rate from $\mathbf{1}_{\text{tight}}$ to $\mathbf{1}_{\text{loose}}$

$\mathbf{1}_{\text{tight}}$ (0.52 mg, 0.19 μmol) was dissolved in acetone-*d*6 (0.5 mL, 0.38 mM). To this solution was added 1,4-dioxane as an internal standard. Then, this solution was allowed to stand at 293 K for 9 days with monitored by ^{1}H NMR spectroscopy.

The rate constant of isomerisation from $\mathbf{1}_{\text{tight}}$ to $\mathbf{1}_{\text{loose}}$ was estimated by curve fitting analysis to be 5.7×10^{-6} s^{-1} at 293 K based on equations (3-3) and (3-4).

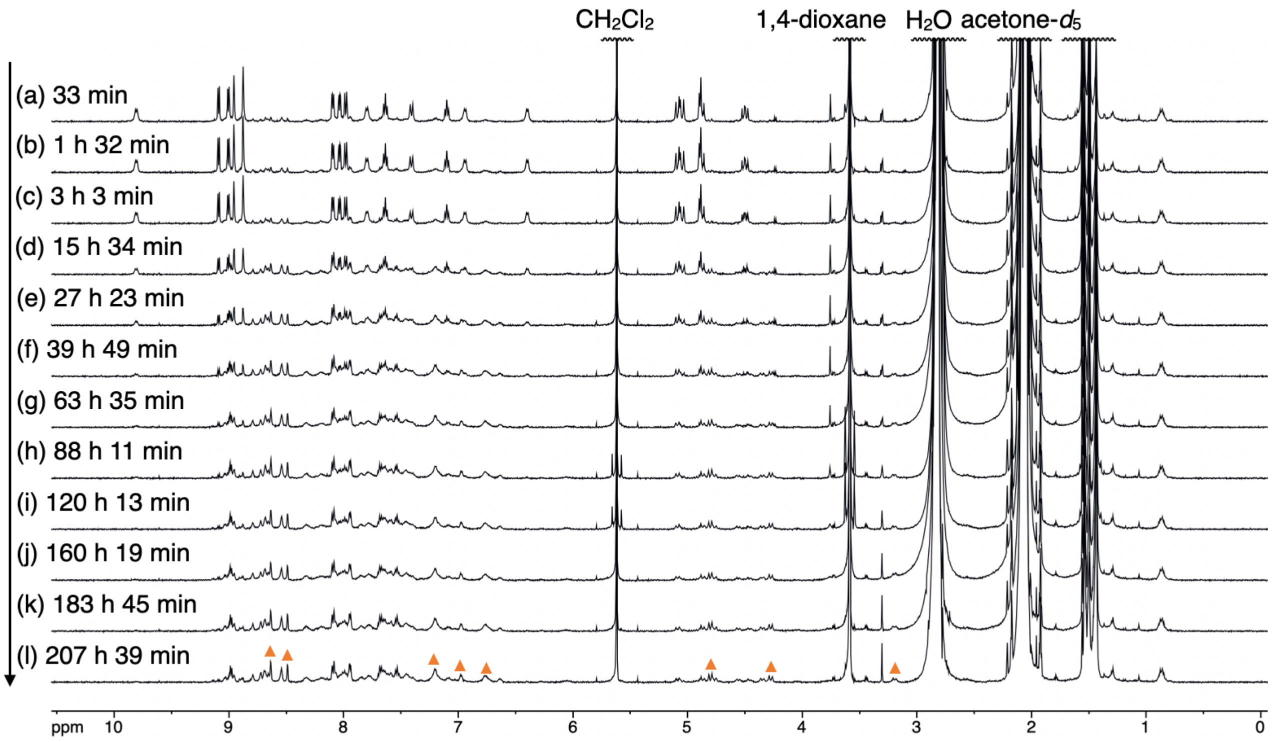

Supplementary Fig. 47 Time course ^{1}H NMR spectra of $\mathbf{1}_{\text{tight}}$ with 1,4-dioxane (a) 33 min, (b) 1 h 32 min, (c) 3 h 3 min, (d) 15 h 34 min, (e) 27 h 23 min, (f) 39 h 49 min, (g) 63 h 35 min, (h) 88 h 11 min, (i) 120 h 13 min, (j) 160 h 19 min, (k) 183 h 45 min and (l) 207 h 39 min at 293 K after dissolved in acetone-*d*6 (500 MHz, acetone-*d*6, 300 K).

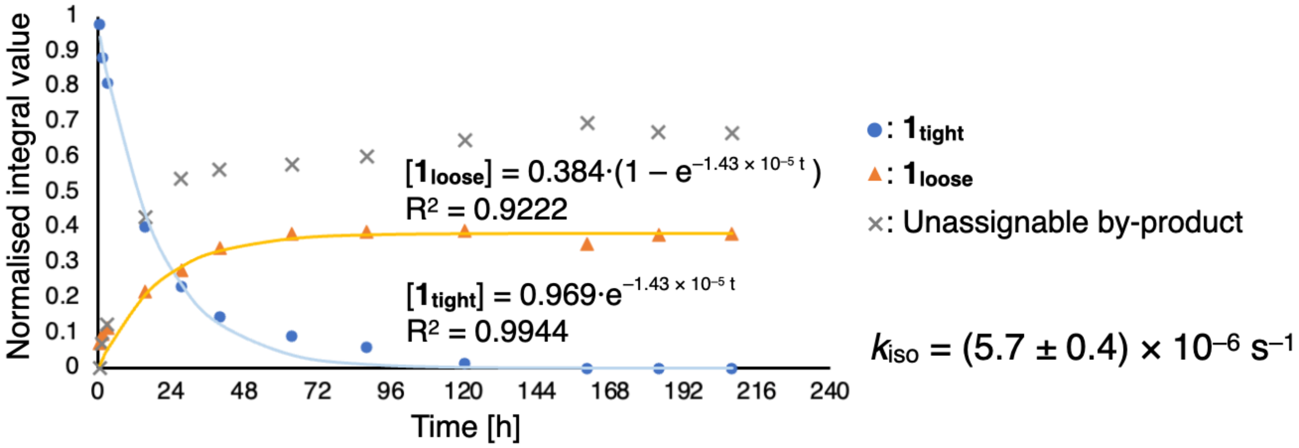

Supplementary Fig. 48 Plot of normalised integral values of $\mathbf{1}_{\text{tight}}$ and $\mathbf{1}_{\text{loose}}$ and unassignable by-products and their fitting curves based on the equations (3-3) and (3-4).

4. Estimation of the rate of helicity inversion by EXSY NMR

4.1 Theoretical analysis of the rate of helicity inversion based on ^{1}H-^{1}H EXSY NMR measurements[9]

^{1}H-^{1}H EXSY NMR measurement was conducted with a Bruker AVANCE500 (500 MHz) spectrometer at 300 K using the standard pulse sequence for phase-sensitive NOESY measurements programmed in XWinNMR Bruker software. Relaxation decay (D1) was set to 2 s, and appropriate mixing times (τ_m = 50–1050 ms) for each trinuclear metallocycle were chosen.

When populations of both exchangeable (*P*/*M*)-isomers were equal, rate constants of exchange processes between two spectroscopically-distinct states (*k*) are derived from according to the equations:

$$k = \frac{1}{\tau_m} \ln \frac{r+1}{r-1}$$

$$r = \frac{I_{AA}+I_{BB}}{I_{AB}+I_{BA}}$$

where τ_m is the mixing time of NOESY measurements, I_{AA} and I_{BB} are the integral values of diagonal peaks, and I_{AB} and I_{BA} are those of cross peaks. After phase and baseline were corrected in both dimensions, the integral values of the diagonal and cross peaks were calculated using iNMR software. Using these calculated integral values, the rate constant (*k*) was calculated from the slope of the plots of $\ln[(r + 1)/(r - 1)]$ versus mixing time (τ_m) by linear regression analysis. In these measurements, the rate constant of the helicity inversion (*k*) was defined as the sum of the rate constants of helicity inversion from (*M*)- to (*P*)-isomers (k_{MP}) and from (*P*)- to (*M*)-isomers (k_{PM}). Linear regression analysis and estimation of the standard error were conducted by StatPlus software[10].

4.2 Theoretical analysis of activation parameters of helicity inversion based on VT EXSY NMR measurements

The enthalpy of activation ($\Delta H^‡$) and the entropy of activation ($\Delta S^‡$) were derived according to the Eyring equation:

$$\ln\frac{k}{T} = -\frac{\Delta H^‡}{R}\frac{1}{T} + \ln\frac{k_B}{h} + \ln\frac{\Delta S^‡}{R}$$

where *R* is the gas constant, k_B is the Boltzmann constant and *h* is the Planck constant. Using these two activation parameters, the Gibbs energy of activation ($\Delta G^‡$) was calculated according to the equation:

$$\Delta G^‡ = \Delta H^‡ - T\Delta S^‡$$

The rate constants (*k*) of helicity inversion at various temperatures were estimated by VT EXSY measurements. Based on the plots of $\ln(k/T)$ versus $1/T$ (Eyring plot), $\Delta H^‡$ and $\Delta S^‡$ were estimated from the slope and the y-intercept of the plot, respectively.

4.3 VT EXSY NMR measurements of $\mathbf{1}_{\mathbf{loose}}$ in acetone-d_6 and estimation of the rate and activation parameters of the helicity inversion

To estimate the rate constant of helicity inversion, two sets of chemical exchange signals between H_d and H_f, and between H_o and H_v can be used. However, since the intensity of the chemical exchange signals between H_d and H_f were too weak to accurately calculate the integral value at lower temperatures, the signals between H_o and H_v were used to estimate the rate constant (k) and activation parameters. Moreover, estimation of the inversion rate was well reproduced at 300 K even using $\mathbf{1}_{\mathbf{loose}}$ obtained in different batches.

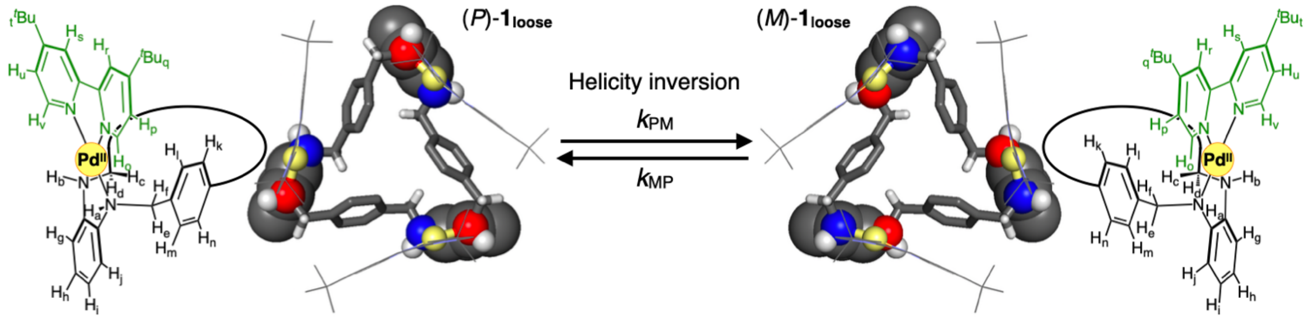

Supplementary Fig. 49 Helicity inversion between the (M)- and (P)-$\mathbf{1}_{\mathbf{loose}}$ with their chemical structural formula.

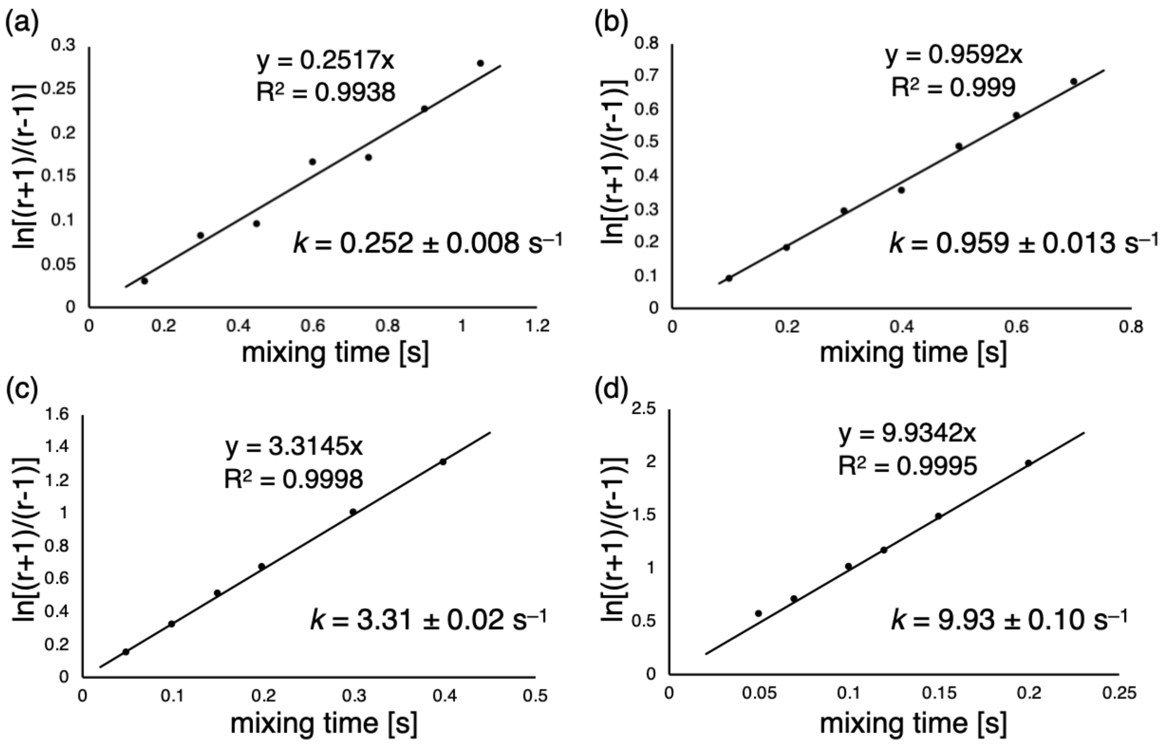

Supplementary Fig. 50 Plots of ln[(r + 1)/(r – 1)] versus mixing time τ_m for $\mathbf{1}_{\mathbf{loose}}$ in acetone-d_6 from ^{1}H-^{1}H VT EXSY spectra (500 MHz, acetone-d_6, 0.19 mM) recorded with different mixing times (0.05–1.05 s) and their fitting line with formula based on chemical exchange signals between H_o and H_v; (a) at 280 K, (b) 290 K, (c) 300 K and (d) 310 K.

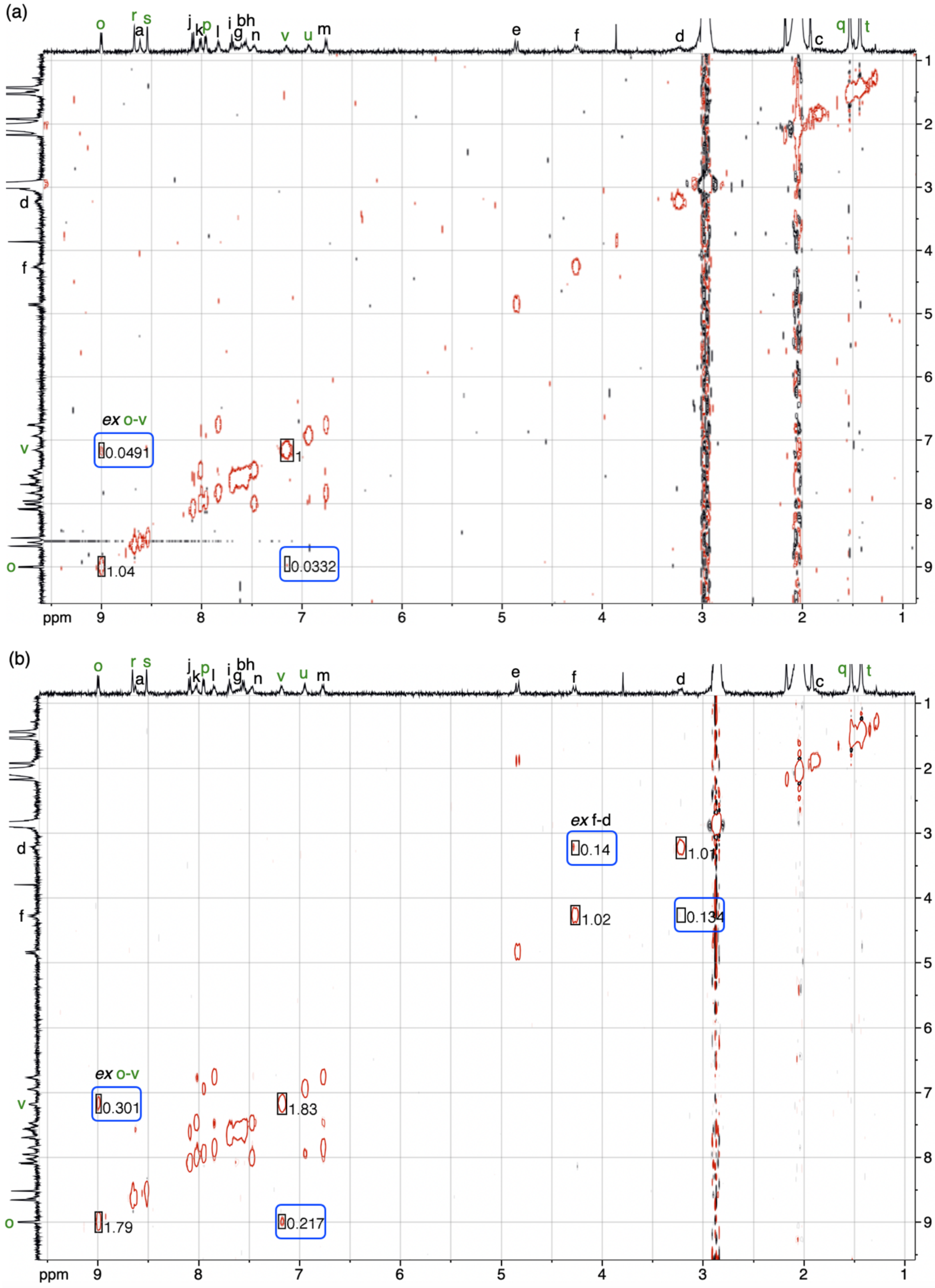
(a)
ex o-v
0.0491
1.04
1
0.0332
(b)
ex f-d
0.14
1.01
1.02
0.134
ex o-v
0.301
1.83
1.79
0.217
ppm

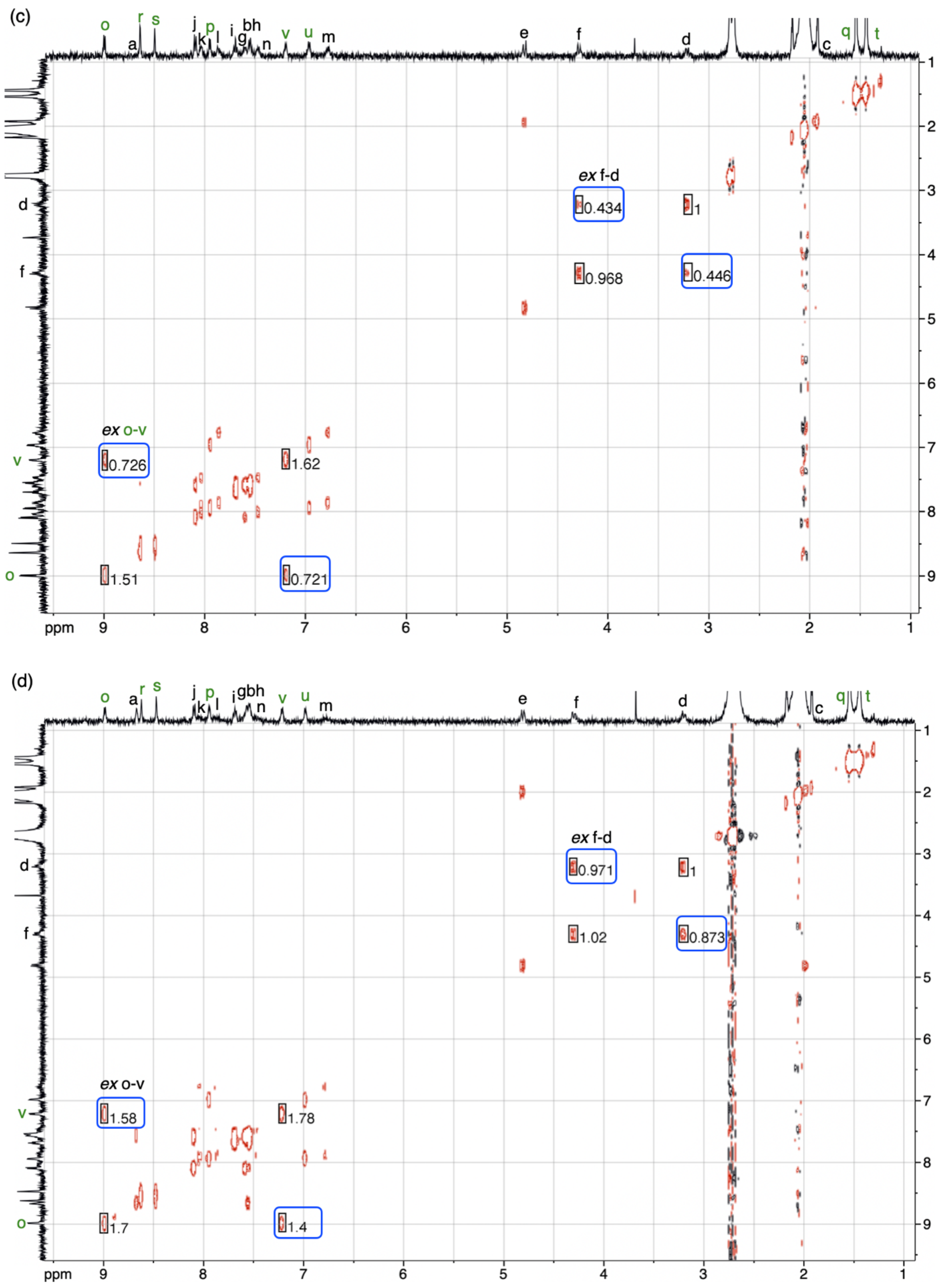

Supplementary Fig. 51 ^{1}H-^{1}H EXSY NMR spectra of $\mathbf{1}_{\mathbf{loose}}$ (500 MHz, acetone-d_6, mixing time τ_m = 0.3 s, 0.19 mM). Blue squares indicate the chemical exchange signals; (a) 280 K, (b) 290 K, (c) 300 K and (d) 310 K.

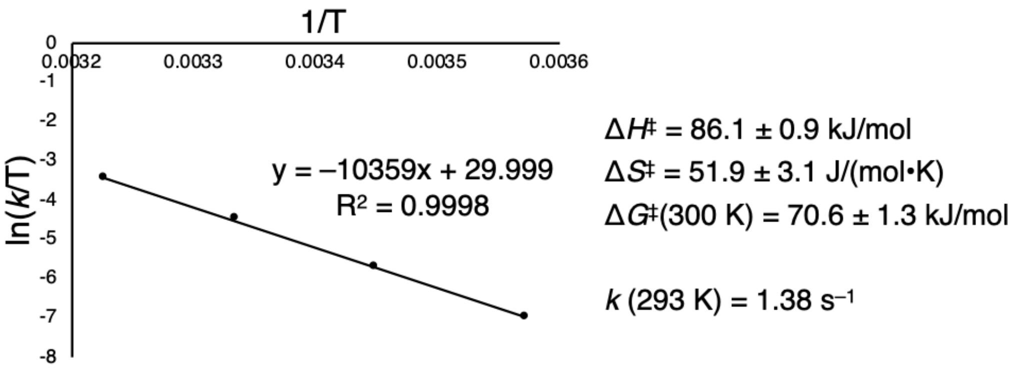

Supplementary Fig. 52 Eyring plot for $\mathbf{1}_{\mathbf{loose}}$ in acetone-d_6 and the enthalpy, entropy and Gibbs energy of activation at 300 K calculated from the plot.

4.4 EXSY NMR measurements of $\mathbf{1}_{\mathbf{tight}}$

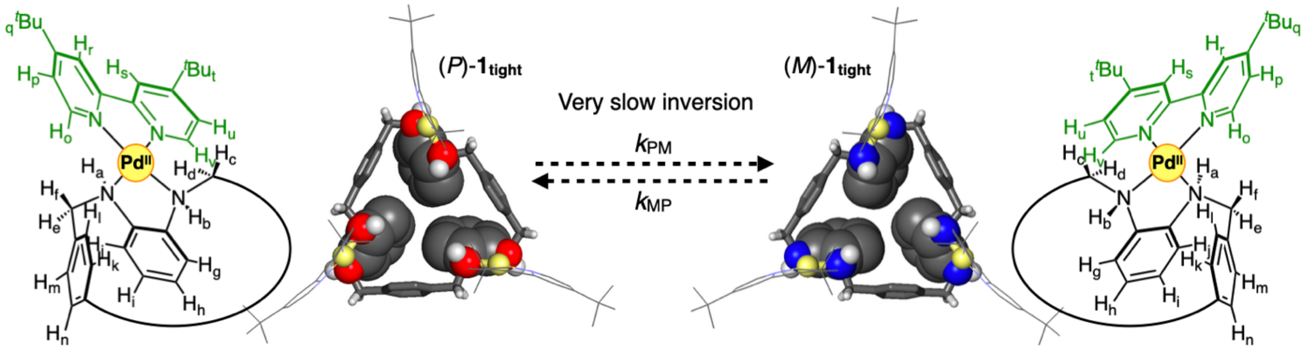

Supplementary Fig. 53 Helicity inversion between (*M*)- and (*P*)-$\mathbf{1}_{\mathbf{tight}}$ and their chemical structural formula.

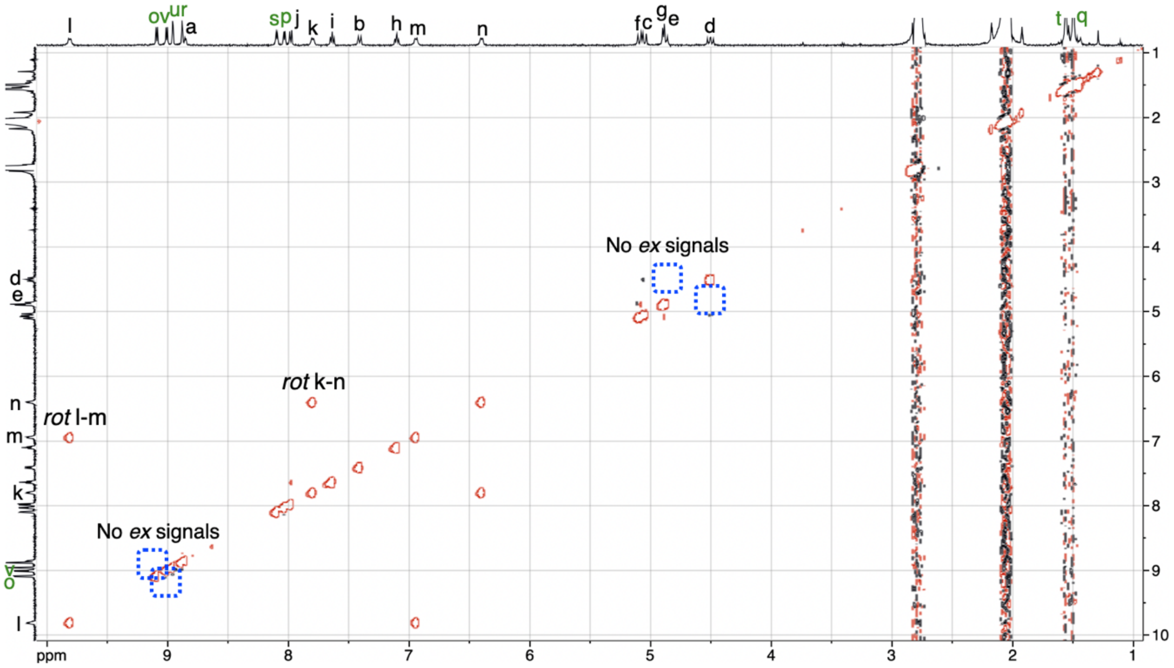

Supplementary Fig. 54 ^{1}H-^{1}H EXSY NMR spectrum of $\mathbf{1}_{\mathbf{tight}}$ (500 MHz, acetone-d_6, 300 K, mixing time τ_m = 0.3 s). Blue dotted squares indicate the absence of chemical exchange signals. The notes "*rot*" indicate chemical exchange signals between the two signals derived from the rotation of *para*-phenylene moieties.

5. Synthesis of chiral sulfoxides

5.1 Synthesis of methanesulfinyl chloride[11]

Dimethyl disulfide (0.4752 g, 5.05 mmol, 1.0 equiv.) and acetic acid (0.6031 g, 10.0 mmol, 2.0 equiv.) were placed in a flask and the atmosphere was then roughly changed to argon by flow. This reaction mixture was stirred and then cooled to –20 °C. To this solution was added sulfuryl chloride (2.1221 g, 15.7 mmol, 3.1 equiv.) dropwise over 8 min, and this reaction mixture was stirred at –20 °C for 3 h. The solution was coloured orange during the addition of sulfinyl chloride and then changed to pale yellow during stirring. Then, the reaction mixture was allowed to return to room temperature for 1 h, during which time sulfur dioxide and hydrochloric acid gases were observed. This reaction mixture was then warmed to 35 °C and stirred for 1 h. The mixture was brought to 105 torr under reduced pressure at room temperature to remove acetyl chloride to give crude methanesulfinyl chloride as a pale yellow liquid (1.1253 g). Since methanesulfinyl chloride is known to be highly unstable, it was used for the next reaction without further purification.

^{1}H NMR (500 MHz, $CDCl_3$, 300 K): δ 3.38 (s, 3H).

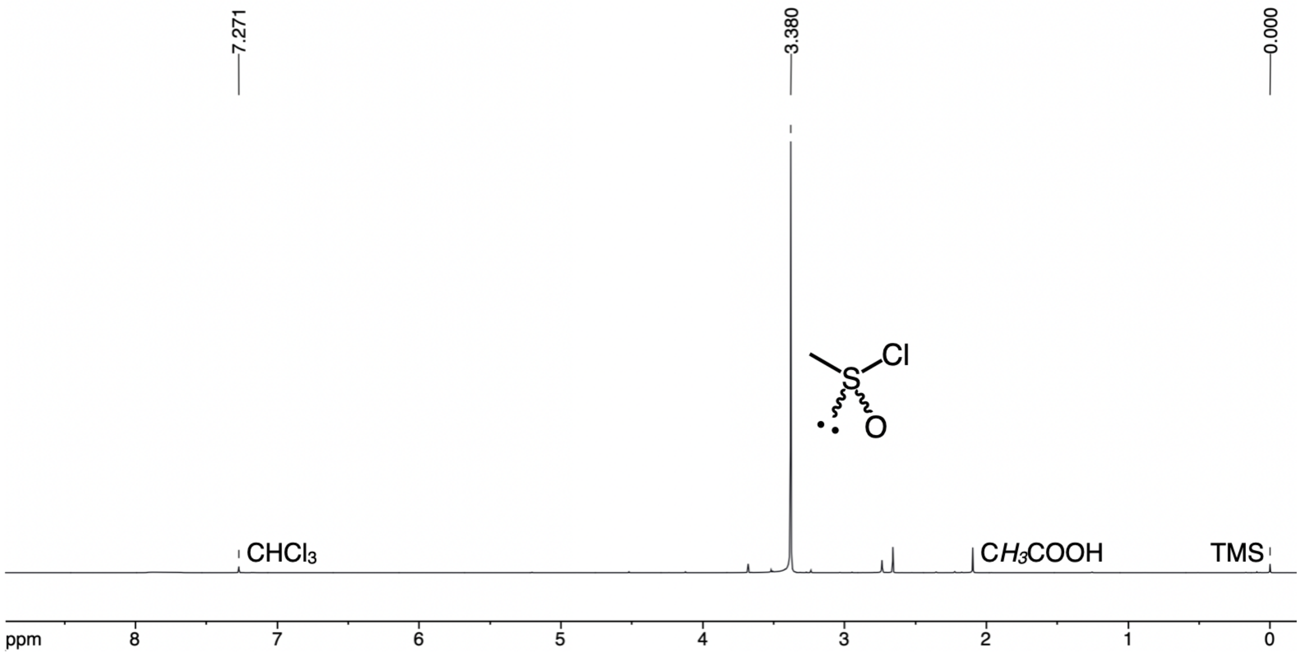

Supplementary Fig. 55 ^{1}H NMR spectrum of crude methanesulfinyl chloride (500 MHz, $CDCl_3$, 300 K).

5.2 Synthesis of 1,2,5,6-di-*O*-isopropylidene-*α*-D-glucofuranosyl (*S*)-methanesulfinate[12]

1,2,5,6-Di-*O*-isopropylidene-*α*-D-glucofuranose (diacetone-D-glucose, DAGOH) (2.4820 g, 9.54 mmol, 1.0 equiv.) and diisopropylethylamine (1.7 mL, 9.8 mmol, 1.0 equiv.) were placed in a flask and dry toluene (46 mL) was added under argon atmosphere. Then, a dry toluene solution (2 mL) of methanesulfinyl chloride (1.1253 g, 11.4 mmol 1.2 equiv.) was added dropwise to the solution over 8 min at –78 °C. This reaction mixture was stirred at –78 °C for 3 h and a colourless precipitate formed during the reaction. The reaction mixture was then brought to room temperature, quenched with water (about 15 mL) and diluted with CH_2Cl_2 (about 10 mL). The organic layer was washed with hydrochloric acid aq. (1 M), sodium hydrogen carbonate aq. (2wt%) and saturated NaCl aq., and then dried over anhydrous sodium sulfate. After evaporation, the resulting colourless solid was recrystallised from *n*-hexane three times to afford (*S*)-MeS(O)ODAG as colourless crystals (0.8392 g, 2.49 mmol, 26%).

^{1}H NMR (500 MHz, $CDCl_3$, 300 K): δ 5.92 (d, J = 3.5 Hz, 1H), 4.78 (d, J = 2.0 Hz, 1H), 4.62 (d, J = 3.5 Hz, 1H), 4.27 (m, 2H), 4.11 (m, 1H), 4.02 (m, 1H), 2.70 (s, 1H), 1.51 (s, 3H), 1.43 (s, 3H), 1.34 (s, 3H), 1.31 (s, 3H). MS (ESI-TOF): m/z = 345.10 as $[MeS(O)ODAG\cdot Na]^+$ (calcd 345.10).

Crystal data for (*S*)-MeS(O)ODAG: $C_{13}H_{22}O_7S$, F_w = 322.36, crystal dimensions 0.262 × 0.051 × 0.021 mm^3, orthorhombic, space group $P2_12_12_1$, a = 5.5406(6), b = 15.7441(18), c = 17.755(2) Å, V = 1548.8(3) Å^3, Z = 4, ρ_{calcd} = 1.382 g cm^{-3}, μ = 21.39 cm^{-1}, T = 93.15 K, λ(CuKα) = 1.54184 Å, $2\theta_{max}$ = 136.394°, 5632/2713 reflections collected/unique (R_{int} = 0.0779), R_1 = 0.1366 ($I > 2\sigma(I)$), wR_2 = 0.3536 (for all data), Flack parameter = 0.07(8), GOF = 1.070, largest diff. peak and hole 1.658/–0.405 eÅ^{-3}. CCDC deposit number 2190133.

PLAT094_ALERT_2_B Ratio of Maximum / Minimum Residual Density … 4.09

Response: The atom type is correct and there is no evidence of twinning. The large residual density may be due to an anomalous dispersion effect and has no chemical significance.

PLAT097_ALERT_2_B Large Reported Max. (Positive) Residual Density … 1.66 eA^{-3}

Response: The atom type is correct and there is no evidence of twinning. The large residual density may be due to an anomalous dispersion effect and has no chemical significance.

PLAT340_ALERT_3_B Low Bond Precision on C-C Bonds … 0.01789 Ang.

Response: The average intensity of reflections is relatively low due to small size of the crystal.

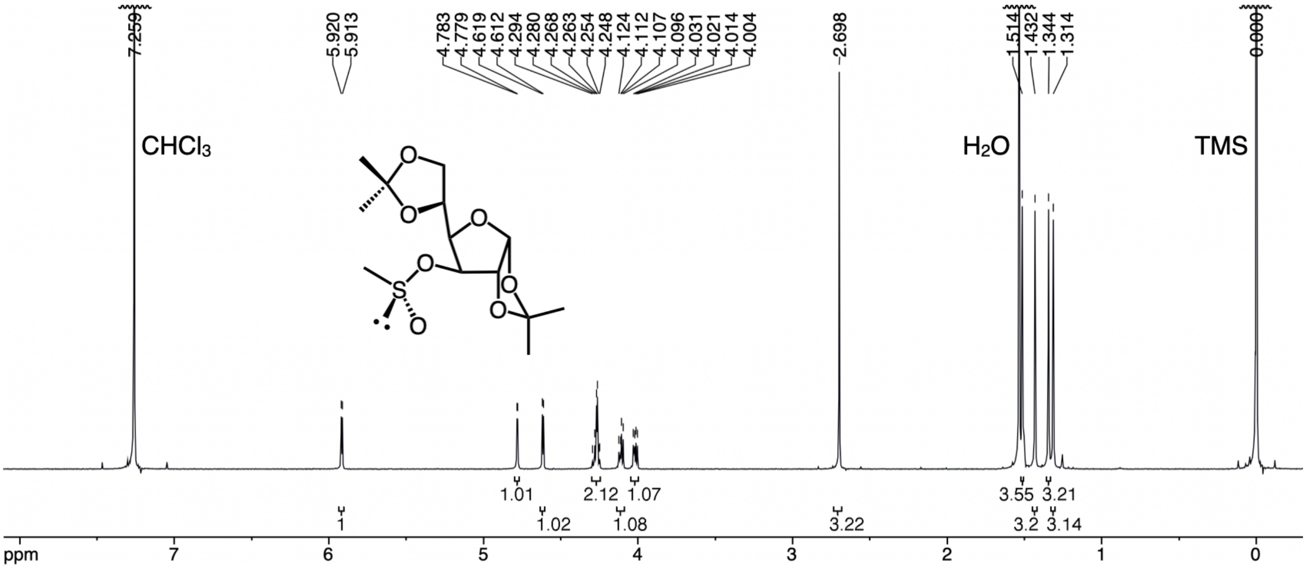

Supplementary Fig. 56 ^{1}H NMR spectrum of (*S*)-MeS(O)ODAG (500 MHz, $CDCl_3$, 300 K).

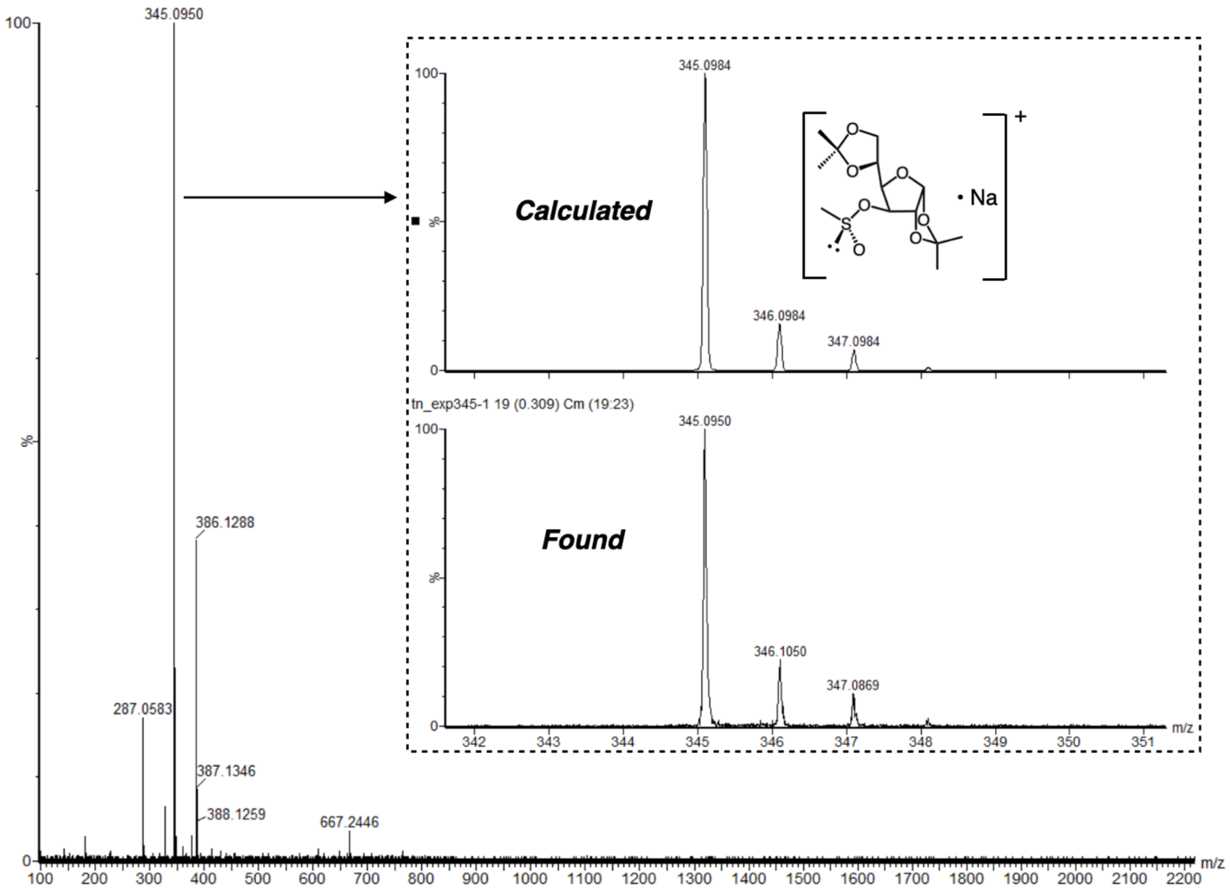

Supplementary Fig. 57 ESI-TOF mass spectrum of (*S*)-MeS(O)ODAG (positive, MeOH).

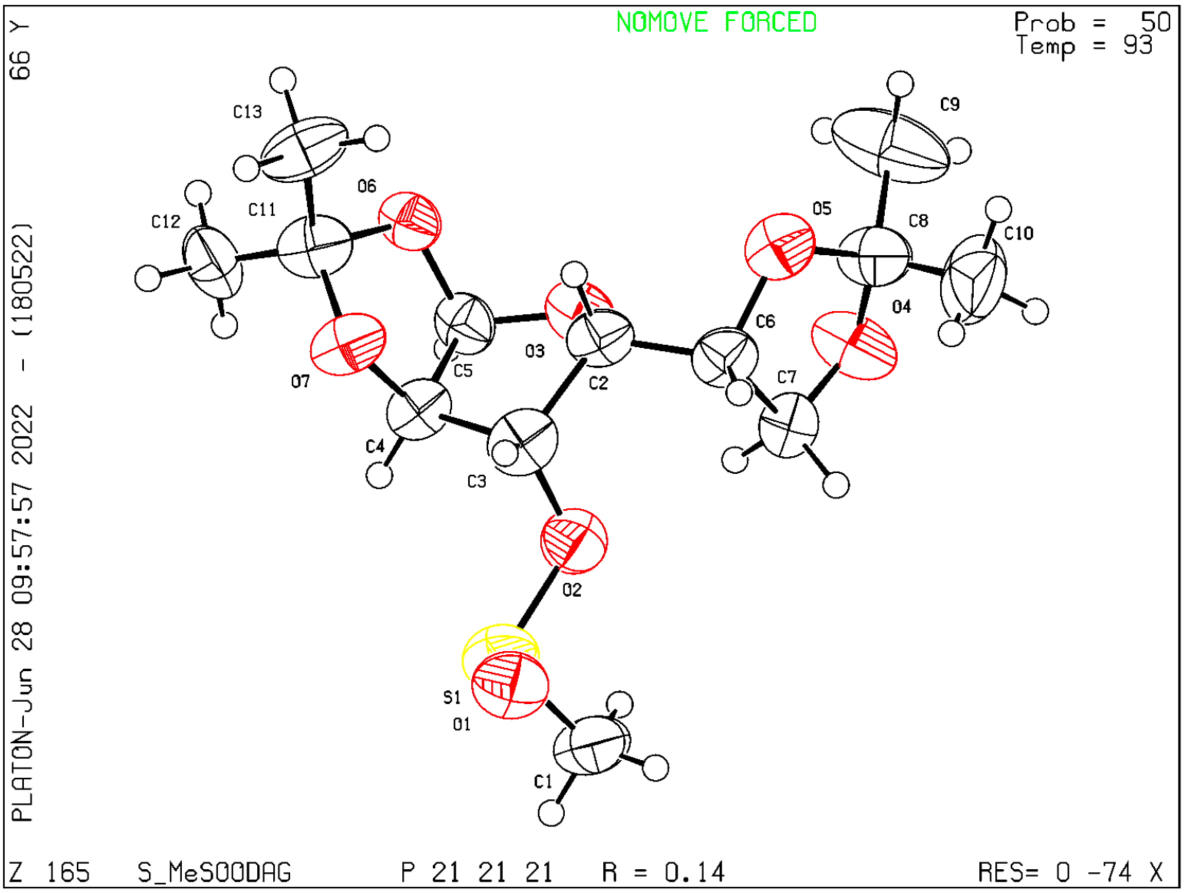

Supplementary Fig. 58 ORTEP drawing of (*S*)-MeS(O)ODAG at the 50% probability level. Colour: C black, O red and S yellow. CCDC deposit number of 1,2,5,6-di-*O*-isopropylidene-α-D-glucofuranosyl (*S*)-methanesulfinate ((*S*)-MeS(O)ODAG) is 2190133. This figure was produced by the checkCIF report of the International Union of Crystallography.

5.3 Synthesis of (*S*)-3[12]

The compound was synthesised with reference to a report of similar compounds[12]. Mg turnings (140.8 mg, 5.79 mmol, 1.0 equiv.) was added to an oven-dried flask under argon atmosphere, an then dry THF (6.0 mL) was poured into the flask. To this mixture, 1,2-dibromoethane (120 µL, 1.39 mmol, 0.24 equiv.) was added with stirring as a reaction initiator, followed by addition of mesityl bromide (770 µL, 5.03 mmol, 0.87 equiv.) dropwise over 10 min. This reaction mixture was heated at reflux for 2 h to obtain a grey suspension of the desired Grignard reagent, 2-mesitylmagnesium bromide.

(*S*)-MeS(O)ODAG (399.9 mg, 1.19 mmol) was placed in an oven-dried flask under argon atmosphere and dry toluene (36 mL) was poured into the flask. After cooling down to 0 °C, the suspension of 2-mesitylmagnesium bromide was added to this solution dropwise over 12 min, and the reaction mixture was stirred at 0 °C for 75 min. After quenching with sat. NH_4Cl aq., the reaction mixture was diluted with CH_2Cl_2. The organic layer was dried over anhydrous sodium sulfate and evaporated. The crude product was then purified by silica gel column chromatography (eluent: AcOEt: *n*-hexane = 1:1). After removal of the solvent, the desired sulfoxide (*S*)-**3** was obtained as a colourless oil (173.9 mg, 0.954 mmol, 80% yield, 96% ee). The optical purity of the product was determined by chiral HPLC analysis as shown in Supplementary Fig. 66a.

^{1}H NMR (500 MHz, $CDCl_3$, 300 K): δ 6.87 (s, 2H), 2.85 (s, 3H), 2.56 (s, 6H), 2.29 (s, 3H). MS (ESI-TOF): m/z = 205.06 as [MesS(O)Me·Na]$^+$ (calcd 205.07). $[\alpha]$ = –234.7 (c = 0.01735, $CHCl_3$, 20.6 °C). The obtained value of the specific rotation was consistent with the reported value ($[\alpha]$ = –241 (c = 1, $CHCl_3$, 98% ee for (*R*)-**3**))[13].

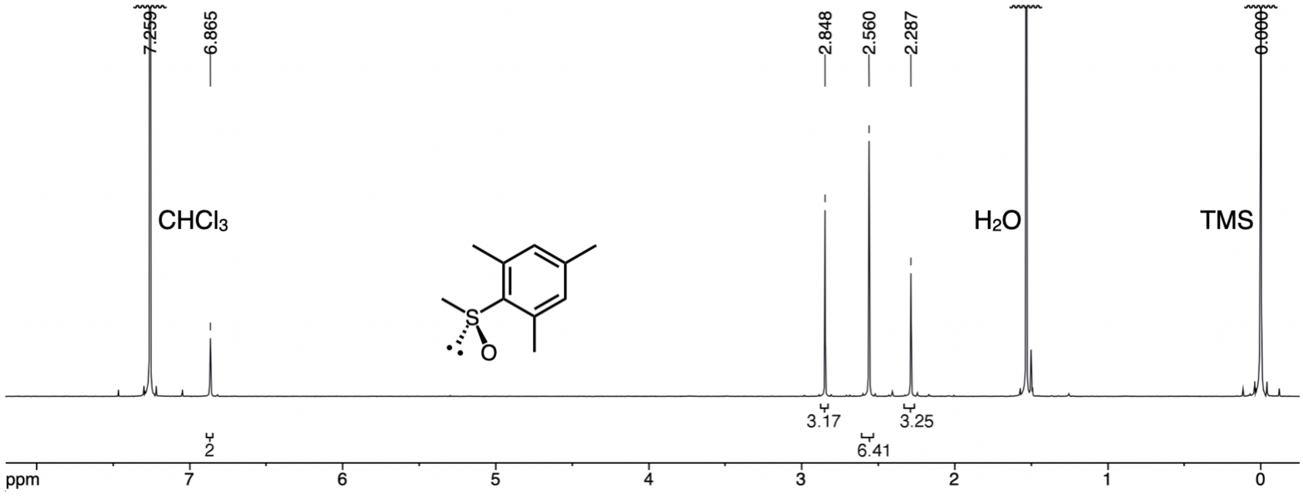

Supplementary Fig. 59 ^{1}H NMR spectrum of (*S*)-**3** (500 MHz, $CDCl_3$, 300 K).

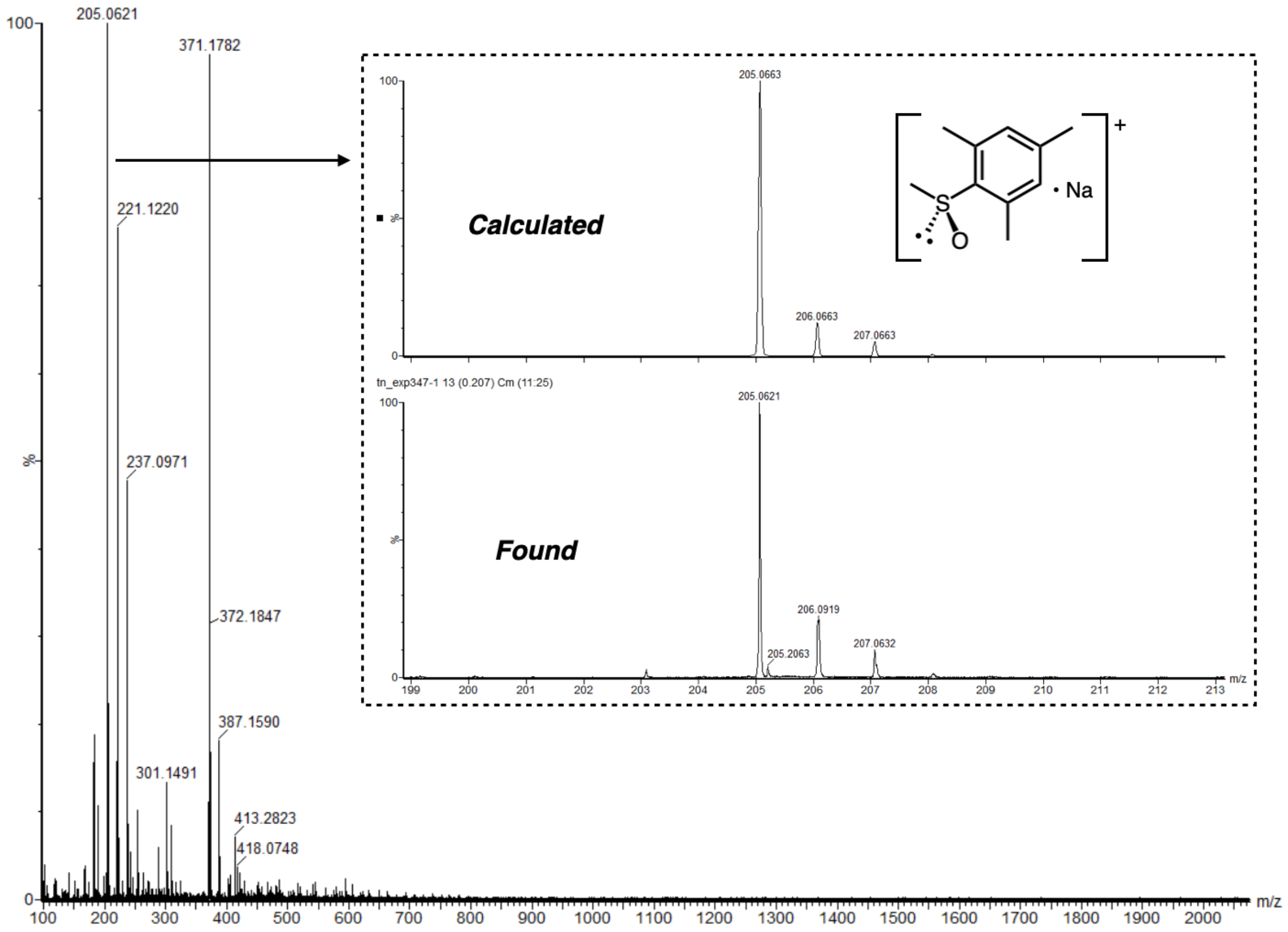

Supplementary Fig. 60 ESI-TOF mass spectrum of (*S*)-**3** (positive, MeOH).

5.4 Synthesis of 1,2,5,6-di-*O*-isopropylidene-*α*-D-glucofuranosyl (*R*)-methanesulfinate[12]

Diacetone-D-glucose (DAGOH, 4.661 g, 17.9 mmol, 1.0 equiv.) and pyridine (1.84 mL, 22.8 μmol, 1.3 equiv.) were placed in a flask and dry THF (90 mL) was poured into the flask under argon atmosphere. After cooling to –78 °C, a THF solution (5 mL) of methanesulfinyl chloride (2.1148 g, 21.5 mmol, 1.2 equiv.) was added to the reaction mixture dropwise over 8 min (some of the methanesulfinyl chloride was accidentally added at room temperature). This reaction mixture was stirred at –78 °C for 3 h and a colourless precipitate was formed during the reaction. After bringing the reaction mixture to come to room temperature, the reaction mixture was quenched with water (about 50 mL) and diluted with CH_2Cl_2 (about 100 mL). The organic layer was washed with hydrochloric acid aq. (1 M), sodium hydrogen carbonate aq. (2wt%) and saturated NaCl aq., and then dried over anhydrous sodium sulfate. After evaporation, the resulting pale yellow oil was purified by column chromatography twice (eluent: *n*-hexane:2-propanol = 15:1) to obtain a mixture of (*R*)-MeS(O)ODAG and diacetone-D-glucose as a colourless oil (3.8074 g, 8.62 mmol for (*R*)-MeS(O)ODAG, 48% (estimated by ^{1}H NMR)), since (*R*)-MeS(O)ODAG was gradually decomposed during purification to form diacetone-D-glucose. The ratio of (*R*)-MeS(O)ODAG to diacetone-D-glucose in the product varied from batch to batch of synthesis.

^{1}H NMR (500 MHz, $CDCl_3$, 300 K): δ 5.95 (d, J = 3.5 Hz, 1H, DAGOH), 5.92 (d, J = 3.5 Hz, 1H, (*R*)-MeS(O)ODAG), 4.76 (d, J = 3.5 Hz, 1H, (*R*)-MeS(O)ODAG), 4.72 (d, J = 3.0 Hz, 1H, (*R*)-MeS(O)ODAG), 4.54 (d, J = 3.5 Hz, 1H, DAGOH), 4.36-4.32 (m, 2H, DAGOH), 4.19-4.10 (m, 3H and 1H, (*R*)-MeS(O)ODAG and DAGOH), 4.08 (dd, J = 7.5, 2.5 Hz, 1H, DAGOH), 4.02-3.97 (m, 1H and 1H, (*R*)-MeS(O)ODAG and DAGOH) 2.70 (s, 3H, (*R*)-MeS(O)ODAG), 2.45 (d, J = 3.5 Hz, 1H, DAGOH), 1.50 (s, 3H and 3H, (*R*)-MeS(O)ODAG and DAGOH), 1.45 (s, 3H, DAGOH), 1.42 (s, 3H, (*R*)-MeS(O)ODAG), 1.36 (s, 3H, DAGOH), 1.33 (s, 3H, (*R*)-MeS(O)ODAG), 1.32 (s, 3H, DAGOH), 1.30 (s, 3H, (*R*)-MeS(O)ODAG). MS (ESI-TOF): m/z = 345.09 as $[\mathrm{MeS(O)ODAG{\cdot}Na}]^+$ (calcd 345.10).

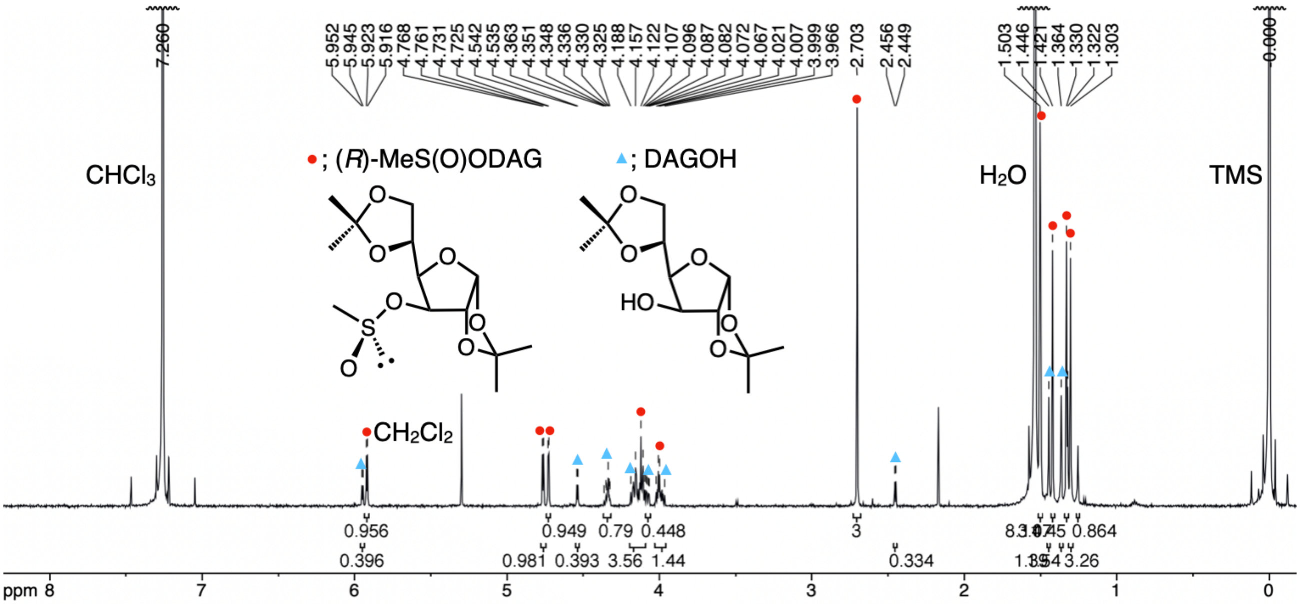

Supplementary Fig. 61 ^{1}H NMR spectrum (500 MHz, $CDCl_3$, 300 K) of a mixture of (*R*)-MeS(O)ODAG and diacetone-D-glucose (500 MHz, $CDCl_3$, 300 K).

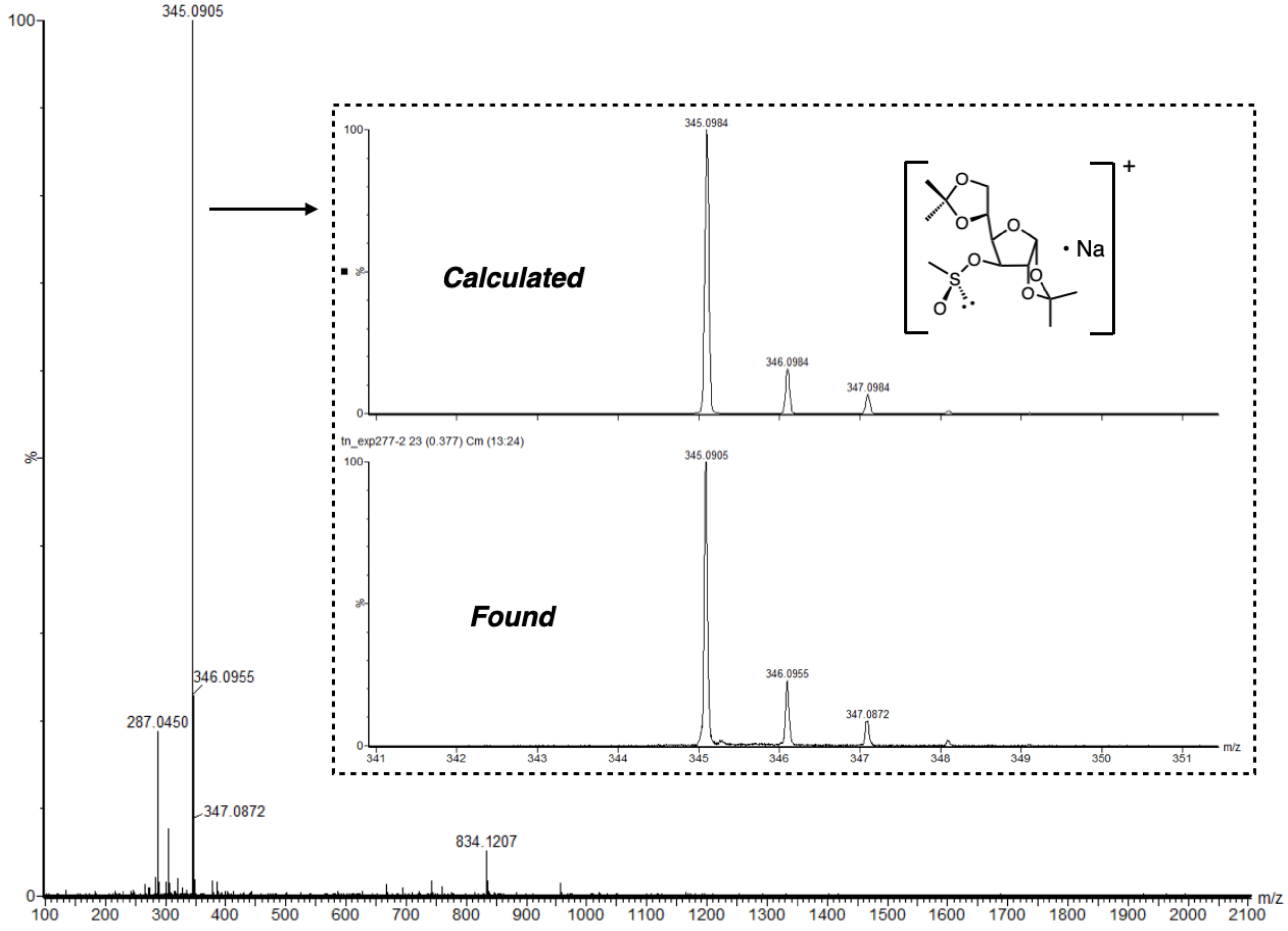

Supplementary Fig. 62 ESI-TOF mass spectrum of (*R*)-MeS(O)ODAG (positive, MeOH).

5.5 Synthesis of (*R*)-3[12]

The compound was synthesised with reference to a report of similar compounds[12]. Mg turnings (200.9 mg, 8.27 mmol, 1.0 equiv.) was placed in an oven-dried flask and dry THF (8.2 mL) was poured into the flask under argon atmosphere. To this suspension was added with stirring 1,2-dibromoethane (170 µL, 1.97 mmol, 0.24 equiv.) as a reaction initiator. Then, mesityl bromide (1.10 mL, 7.19 mmol, 0.87 equiv.) was added dropwise over 15 min. This reaction mixture was heated at reflux for 2 h to obtain a grey suspension of the desired Grignard reagent, 2-mesitylmagnesium bromide.

(*R*)-MeS(O)ODAG (a mixture of (*R*)-MeS(O)ODAG and diacetone-D-glucose estimated from ^{1}H NMR to be in the molar ratio of 5:2; 745.1 mg, 1.56 mmol for (*R*)-MeS(O)ODAG) was placed in an oven-dried flask under argon atmosphere and dry toluene (34 mL) was poured into the flask. After cooling to 0 °C, the suspension of 2-mesitylmagnesium bromide was added to the solution dropwise over 30 min. This reaction mixture was stirred at 0 °C for 70 min. After quenching with sat. NH_4Cl aq., the reaction mixture was diluted with CH_2Cl_2. The organic layer was dried over anhydrous sodium sulfate and evaporated. The crude product was then purified by silica gel column chromatography twice (eluent: $CHCl_3$:AcOEt = 20:1 or AcOEt:*n*-hexane = 1:1). After removal of the solvent, (*R*)-**3** was obtained as a colourless oil (167.6 mg, 0.919 mmol, 58%, 96% ee). The optical purity of the product was determined by chiral HPLC analysis as shown in Supplementary Fig. 66b.

^{1}H NMR (500 MHz, $CDCl_3$, 300 K): δ 6.87 (s, 2H), 2.85 (s, 3H), 2.56 (s, 6H), 2.29 (s, 3H). ^{13}C NMR (126 MHz, $CDCl_3$, 300 K): δ 141.0, 137.8, 136.0, 130.9, 38.4, 21.0, 18.9. MS (ESI-TOF): *m*/*z* = 205.06 as $[MesS(O)Me\cdot Na]^+$ (calcd 205.07). [α] = +235.07 (*c* = 0.01634, 23.6 °C, $CHCl_3$). The obtained value of the specific rotation was consistent with the reported value ([α] = +243 (*c* = 1, $CHCl_3$, 97% ee for (*R*)-**3**))[13].

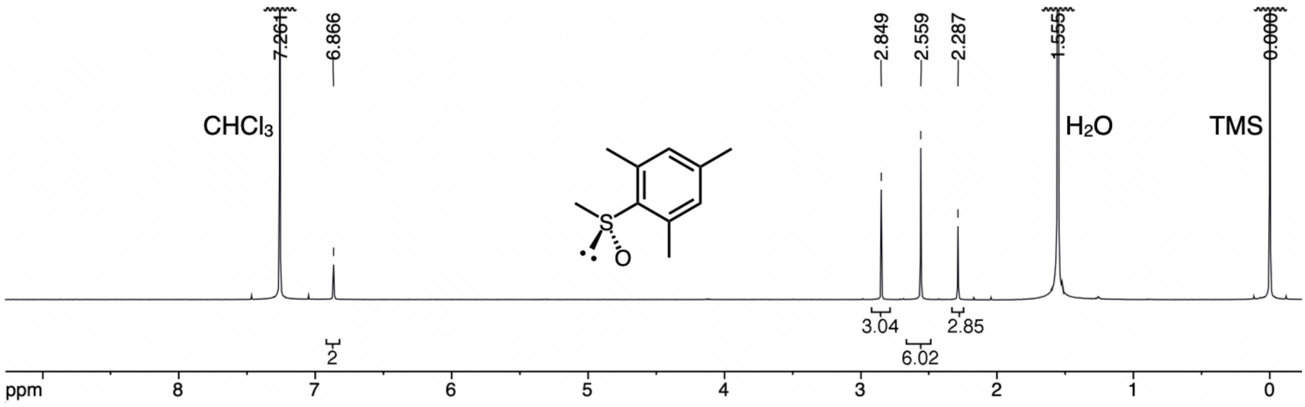

Supplementary Fig. 63 ^{1}H NMR spectrum of (*R*)-**3** (500 MHz, $CDCl_3$, 300 K).

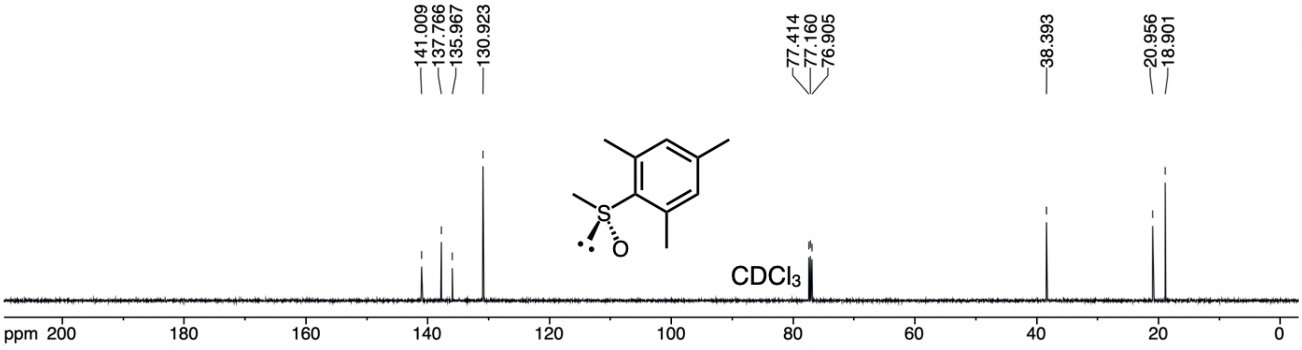

Supplementary Fig. 64 ^{13}C NMR spectrum of (*R*)-**3** (126 MHz, $CDCl_3$, 300 K).

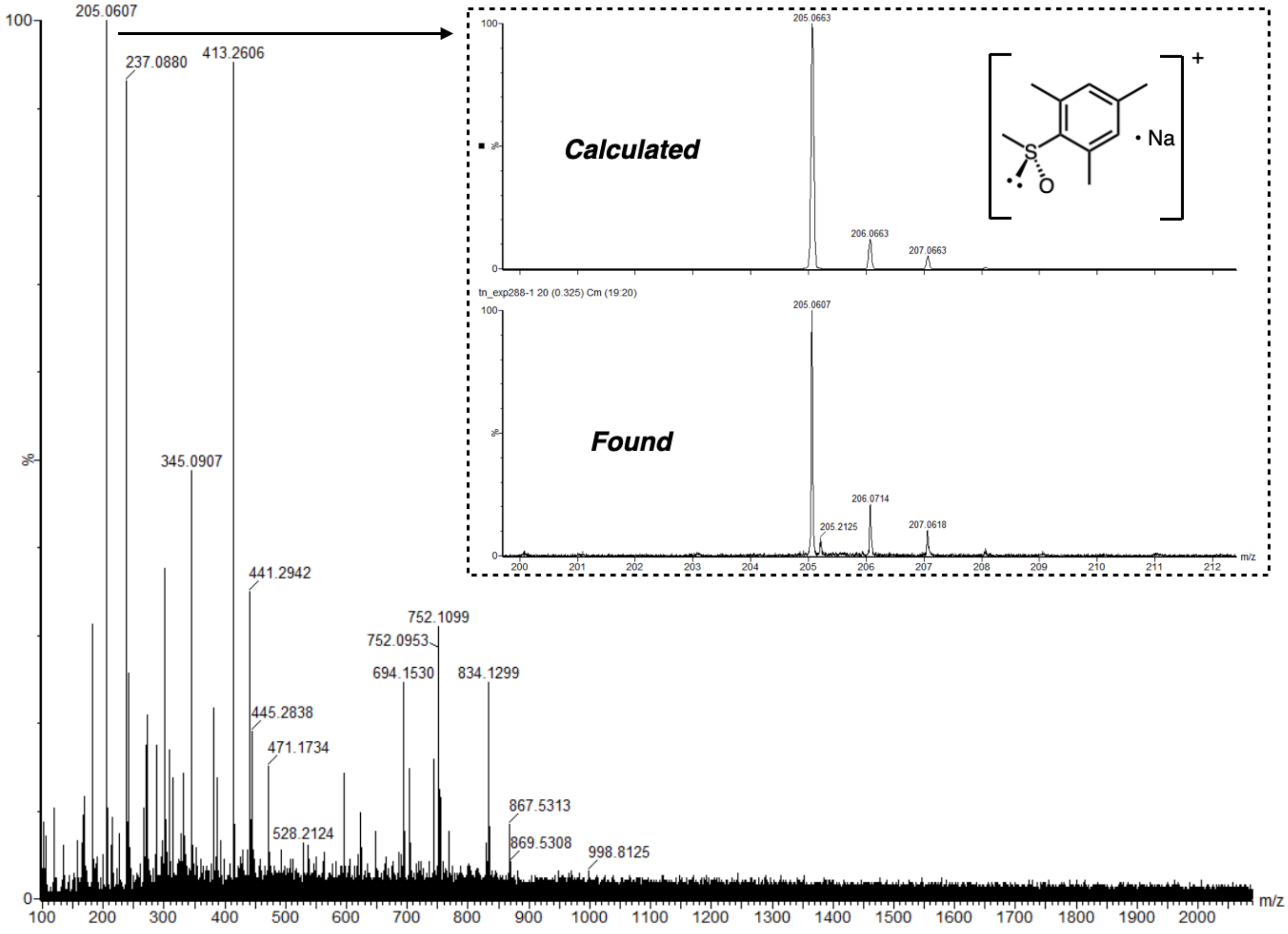

Supplementary Fig. 65 ESI-TOF mass spectrum of (*R*)-**3** (positive, MeOH).

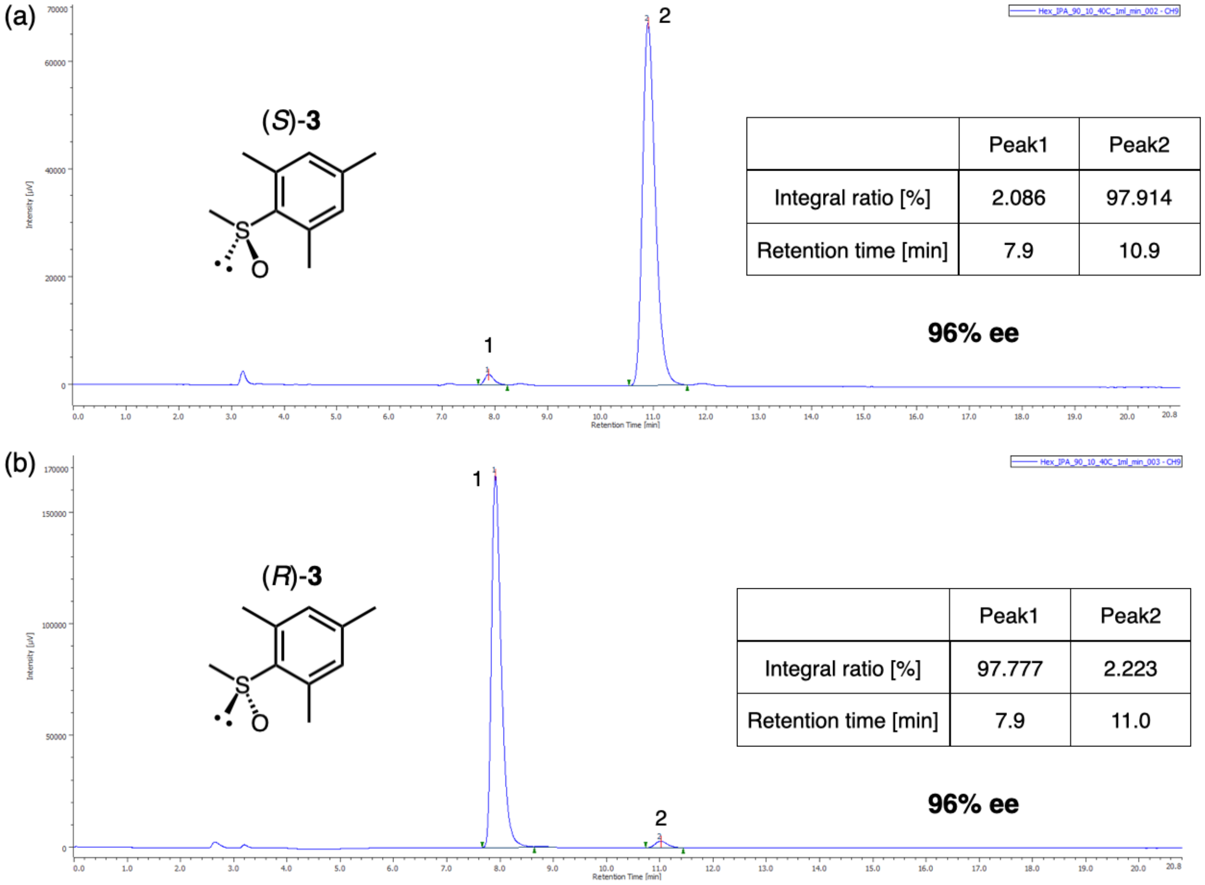

Supplementary Fig. 66 Chiral HPLC traces of (a) (*S*)-**3** and (b) (*R*)-**3** (CHIRALPAK OD-H column, *n*-hexane:2-propanol = 90:10, 40 °C, 1 mL/min, detected with photodiode array at 240 nm).

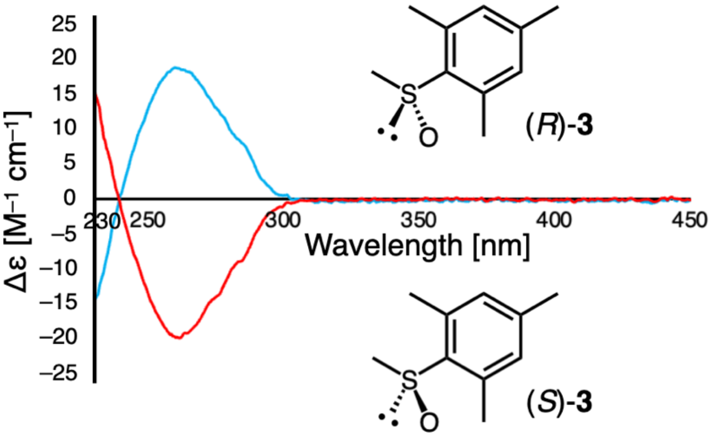

Supplementary Fig. 67 CD spectra (CH_2Cl_2, 293 K, l = 1.0 cm) of (blue line) (*R*)-**3** (0.11 mM) and (red line) (*S*)-**3** (0.12 mM).

5.6 Synthesis of (*R*)-methyl phenyl sulfoxide[12]

Mg turnings (181.7 mg, 7.48 mmol, 1.0 equiv.) was placed in an oven-dried flask and dry THF (7.4 mL) was poured into the flask under argon atmosphere. To this suspension was added bromobenzene (650 μL, 6.21 mmol, 0.83 equiv.) dropwise over 10 min. This reaction mixture was heated at reflux for 2 h to obtain a grey suspension of the desired Grignard reagent, phenylmagnesium bromide.

(*R*)-MeS(O)ODAG (a mixture of (*R*)-MeS(O)ODAG and diacetone-D-glucose estimated from ^{1}H NMR to be in the molar ratio of 5:4; 405.4 mg, 0.743 mmol for (*R*)-MeS(O)ODAG) was placed in an oven-dried flask and dry toluene (31 mL) was poured into the flask under argon atmosphere. After cooling to 0 °C, the suspension of phenylmagnesium bromide was added to the solution dropwise over 7 min. The reaction mixture was stirred at 0 °C for 60 min. After quenching with sat. NH_4Cl aq., the reaction mixture was diluted with CH_2Cl_2. The organic layer was dried over anhydrous sodium sulfate and evaporated. The crude product was then purified by silica gel column chromatography twice (eluent: $CHCl_3$:AcOEt = 5:2 or CH_2Cl_2:AcOEt = 1:1). After removal of the solvent, (*R*)-methyl phenyl sulfoxide was obtained as a colourless oil (40.4 mg, 0.288 mmol, 39%, 93% ee).

^{1}H NMR (500 MHz, $CDCl_3$, 300 K): δ 7.66 (dd, J = 7.5, 1.5 Hz, 2H), 7.53 (m, 3H), 2.73 (s, 3H). ^{13}C NMR (126 MHz, $CDCl_3$, 300 K): δ 145.8, 131.1, 129.5, 123.6, 44.0. MS (ESI-TOF): m/z = 141.02 as $[MeS(O)Ph{\cdot}H]^+$ (calcd 141.04). [α] = +124.2 (c = 0.00372, acetone, 25.4 °C). The obtained value of the specific rotation was consistent with the reported value ([α] = +137 (c = 1, acetone, 93% ee for (*R*)-methyl phenyl sulfoxide))[13].

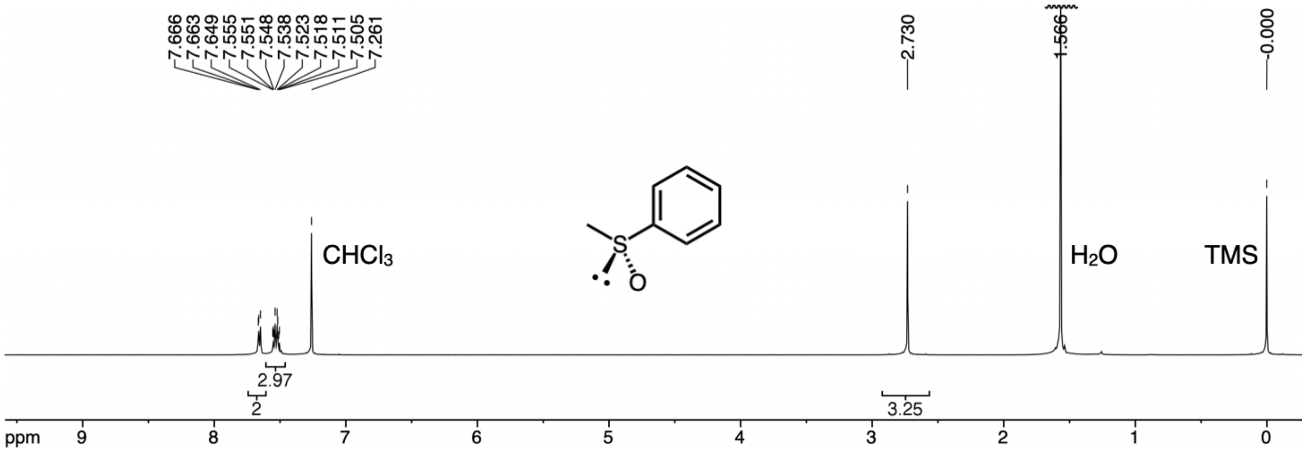

Supplementary Fig. 68 ^{1}H NMR spectrum of (*R*)-methyl phenyl sulfoxide (500 MHz, $CDCl_3$, 300 K).

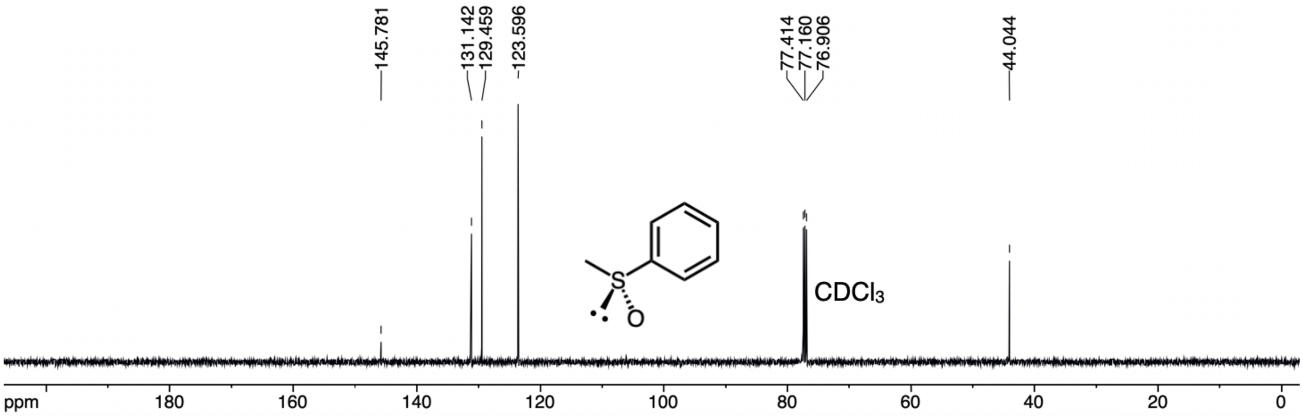

Supplementary Fig. 69 ^{13}C NMR spectrum of (*R*)-methyl phenyl sulfoxide (126 MHz, $CDCl_3$, 300 K).

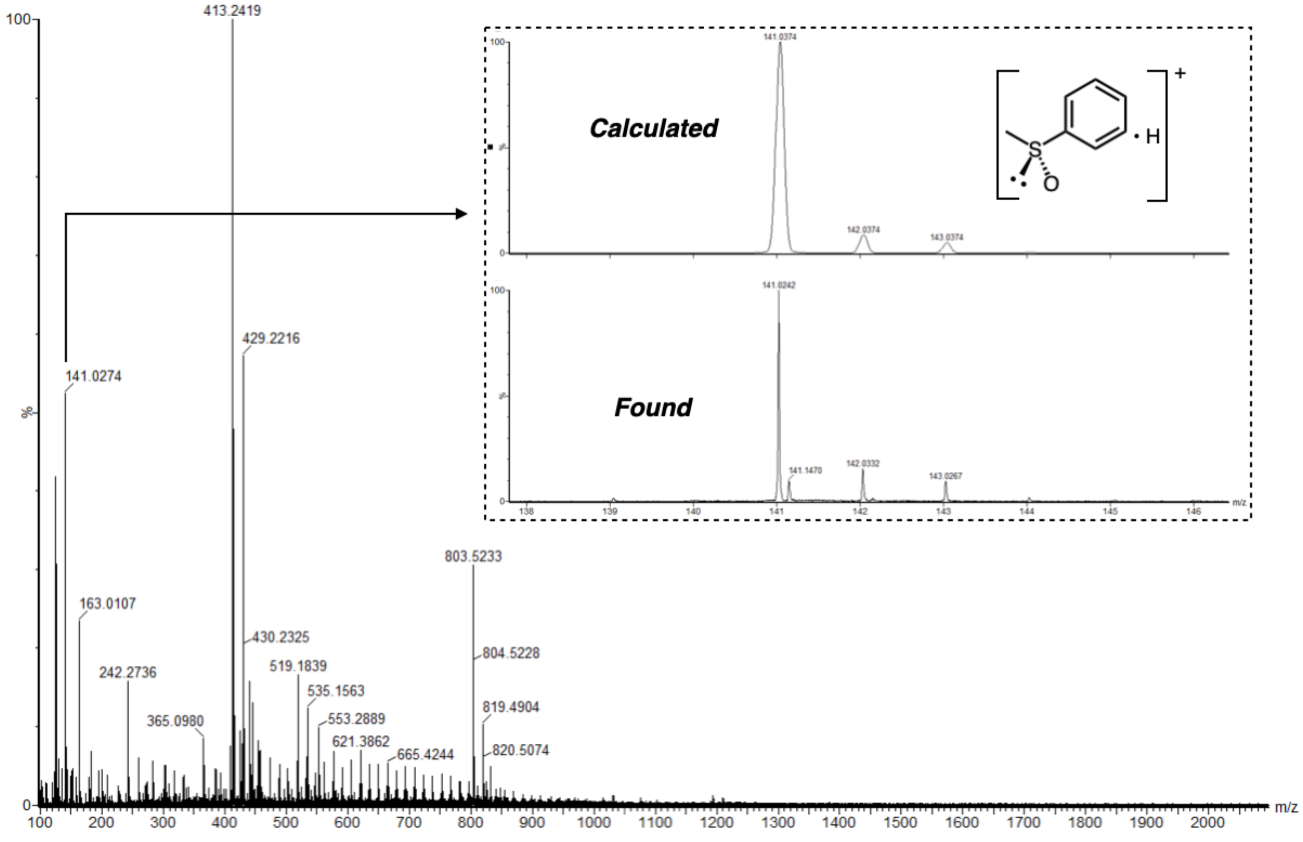

Supplementary Fig. 70 ESI-TOF mass spectrum of (*R*)-methyl phenyl sulfoxide (positive, MeOH).

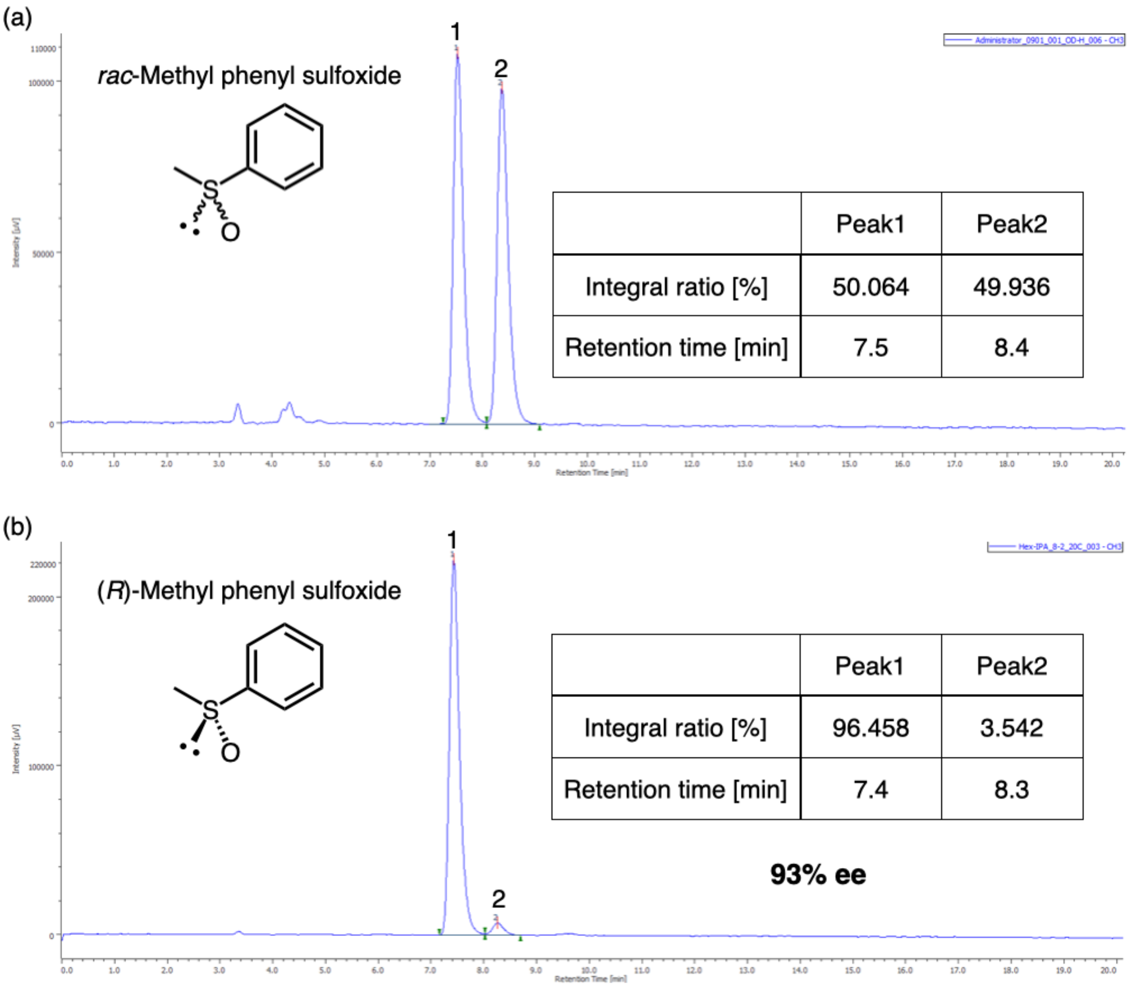

Supplementary Fig. 71 Chiral HPLC traces of (a) *rac*-methyl phenyl sulfoxide and (b) (*R*)-methyl phenyl sulfoxide (CHIRALPAK OD-H column, *n*-hexane:2-propanol = 80:20, 20 °C, 1 mL/min, detected with photodiode array at 240 nm).

5.7 Synthesis of (*R*)-*p*-anisyl methyl sulfoxide[12]

The synthesis of this compound was performed with reference to a report of similar compounds[12]. Mg turnings (201.7 mg, 8.30 mmol, 1.0 equiv.) was placed in an oven-dried flask and dry THF (8.2 mL) was poured into the flask under argon atmosphere. To this suspension was added with stirring 1,2-dibromoethane (95 µL, 1.10 mmol, 0.13 equiv.) as a reaction initiator. Then, *p*-anisyl bromide (1080 µL, 8.63 mmol, 1.04 equiv.) was added dropwise over 30 min and the reaction mixture was heated at reflux for 2 h to obtain a grey suspension of the desired Grignard reagent, *p*-anisylmagnesium bromide.

(*R*)-MeS(O)ODAG (a mixture of (*R*)-MeS(O)ODAG and diacetone-D-glucose estimated from ^{1}H NMR to be in the molar ratio of 5:2; 427.3 mg, 0.969 mmol for (*R*)-MeS(O)ODAG) was placed in an oven-dried flask and dry toluene (30 mL) was poured into the flask under argon atmosphere. After cooling to 0 °C, the suspension of *p*-anisylmagnesium bromide was added to the solution dropwise over 15 min. This reaction mixture was stirred at 0 °C for 60 min. After quenching with sat. NH_4Cl aq., the reaction mixture was diluted with CH_2Cl_2. The organic layer was dried over anhydrous sodium sulfate and evaporated. The crude product was then purified by silica gel column chromatography twice (eluent: from EtOAc:*n*-hexane = 1:1 to EtOAc, or $CHCl_3$:MeOH = 50:1). After removal of the solvent, the resulting pale yellow oil was crystallized in *n*-hexane to afford (*R*)-*p*-anisyl methyl sulfoxide as colourless crystals (39.74 mg, 0.233 mmol, 24%, 97% ee).

^{1}H NMR (500 MHz, $CDCl_3$, 300 K): δ 7.59 (AA'XX' spin system, 2H), 7.04 (dt, J = 9.0, 2.0 Hz, 2H), 3.86 (s, 3H), 2.70 (s, 3H). ^{13}C NMR (126 MHz, $CDCl_3$, 300 K): δ 162.1, 136.7, 125.6, 115.0, 55.7, 44.1. MS (ESI-TOF): m/z = 193.03 as $[AnisS(O)Me\cdot Na]^+$ (calcd 193.03). $[\alpha]$ = +156.67 (c = 0.003974, 26.4 °C, $CHCl_3$). The obtained value of the specific rotation was consistent with the reported value ($[\alpha]$ = +165.9 (c = 0.38, $CHCl_3$, 99.5% ee for (*R*)-*p*-anisyl methyl sulfoxide))[14].

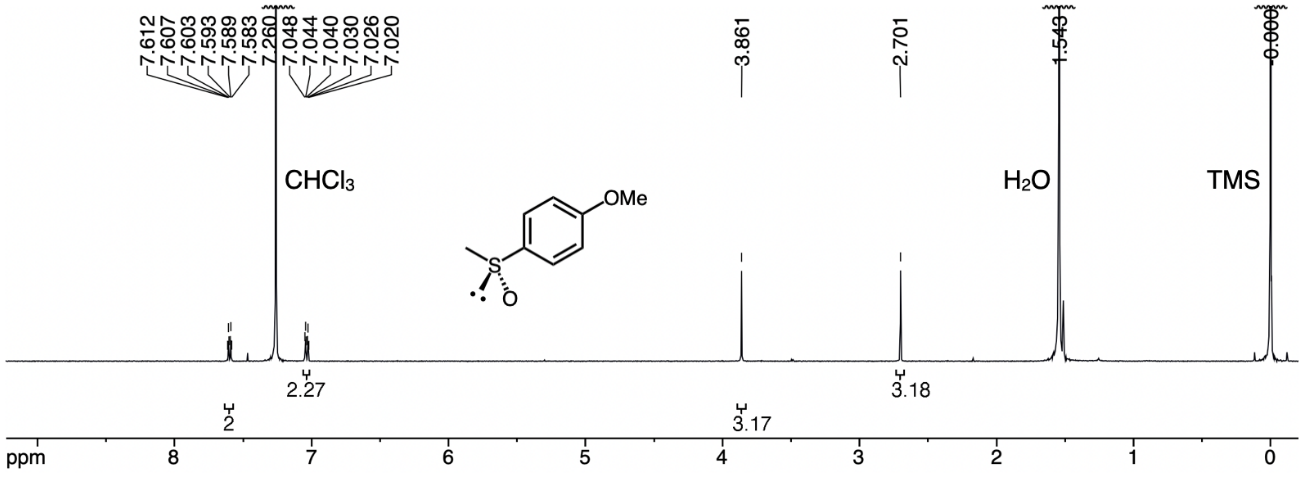

Supplementary Fig. 72 ^{1}H NMR spectrum of (*R*)-*p*-anisyl methyl sulfoxide (500 MHz, $CDCl_3$, 300 K).

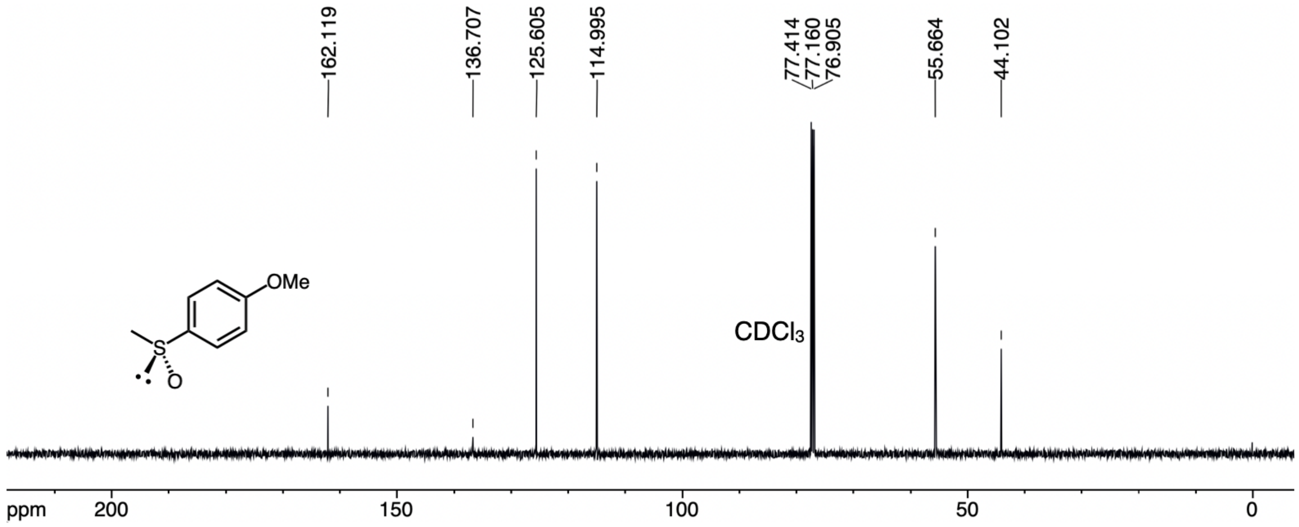

Supplementary Fig. 73 ^{13}C NMR spectrum of (*R*)-*p*-anisyl methyl sulfoxide (126 MHz, $CDCl_3$, 300 K).

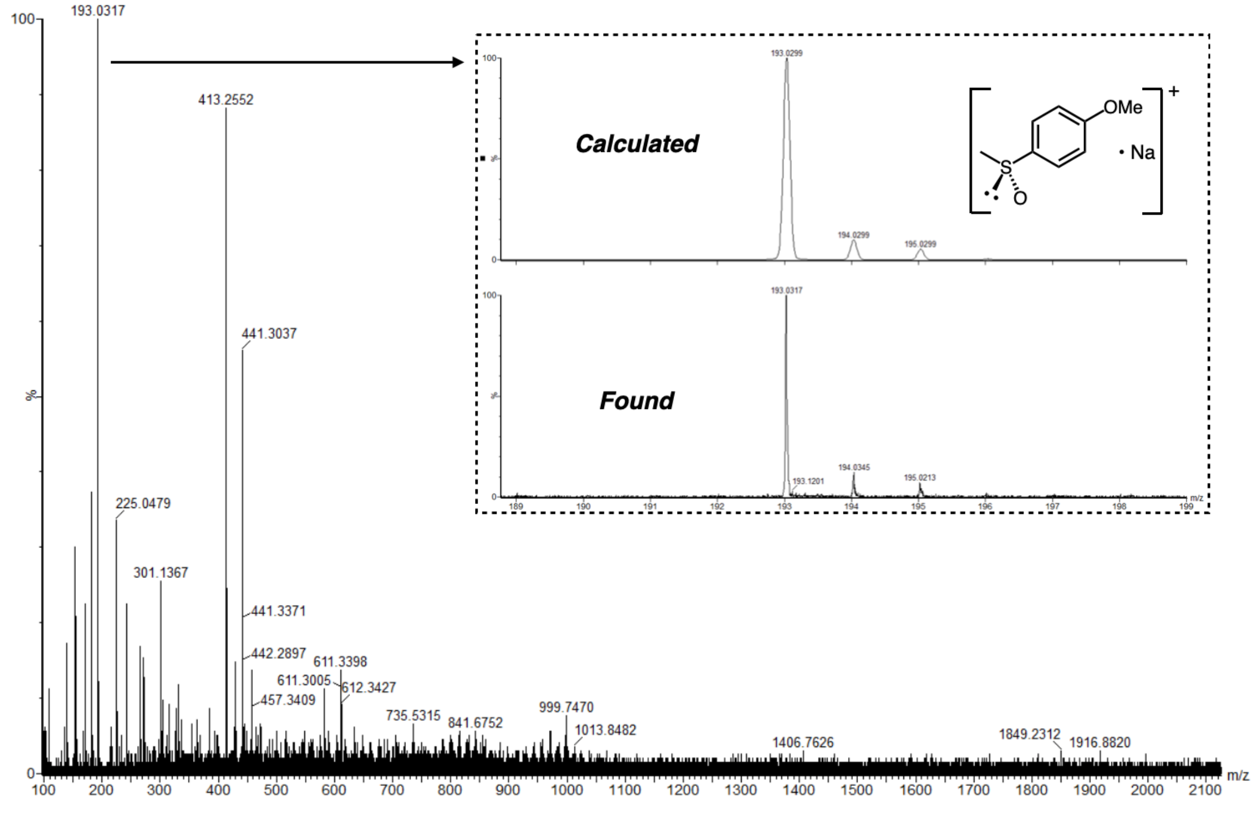

Supplementary Fig. 74 ESI-TOF mass spectrum of (*R*)-*p*-anisyl methyl sulfoxide (positive, MeOH).

For HPLC analysis of the product, *rac*-*p*-anisyl methyl sulfoxide, was prepared by racemisation of (*R*)-*p*-anisyl methyl sulfoxide[15]. To a 1,4-dioxane solution (0.08 mL) of (*R*)-*p*-anisyl methyl sulfoxide (4.0 mg, 23 μmol) was added aqueous hydrogen chloride (0.04 mL, 12 M). The reaction solution was stirred at room temperature for 9 h under argon atmosphere. The reaction solution was then neutralised with aqueous Na_2CO_3 (0.5 mL, 2.0 M) and the sulfoxide was extracted with diethyl

ether. The organic layer was dried over anhydrous sodium sulfate and evaporated to afford *rac*-*p*-anisyl methyl sulfoxide (2.1 mg, 12 μmol, 53%).

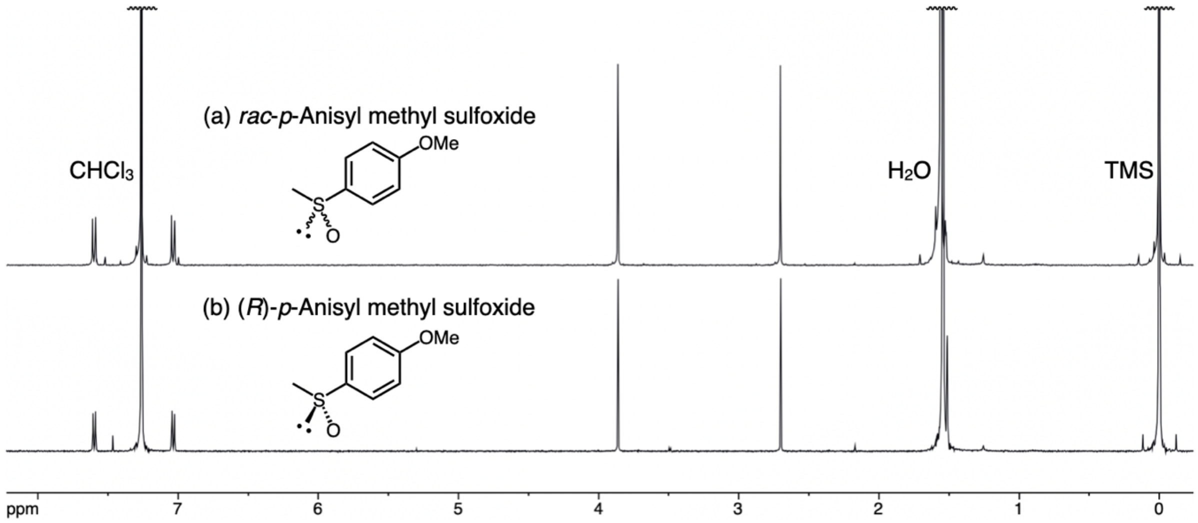

Supplementary Fig. 75 ^{1}H NMR spectra ($CDCl_3$, 300 K) of (a) *rac*-*p*-anisyl methyl sulfoxide (400 MHz) and (b) (*R*)-*p*-anisyl methyl sulfoxide (500 MHz).

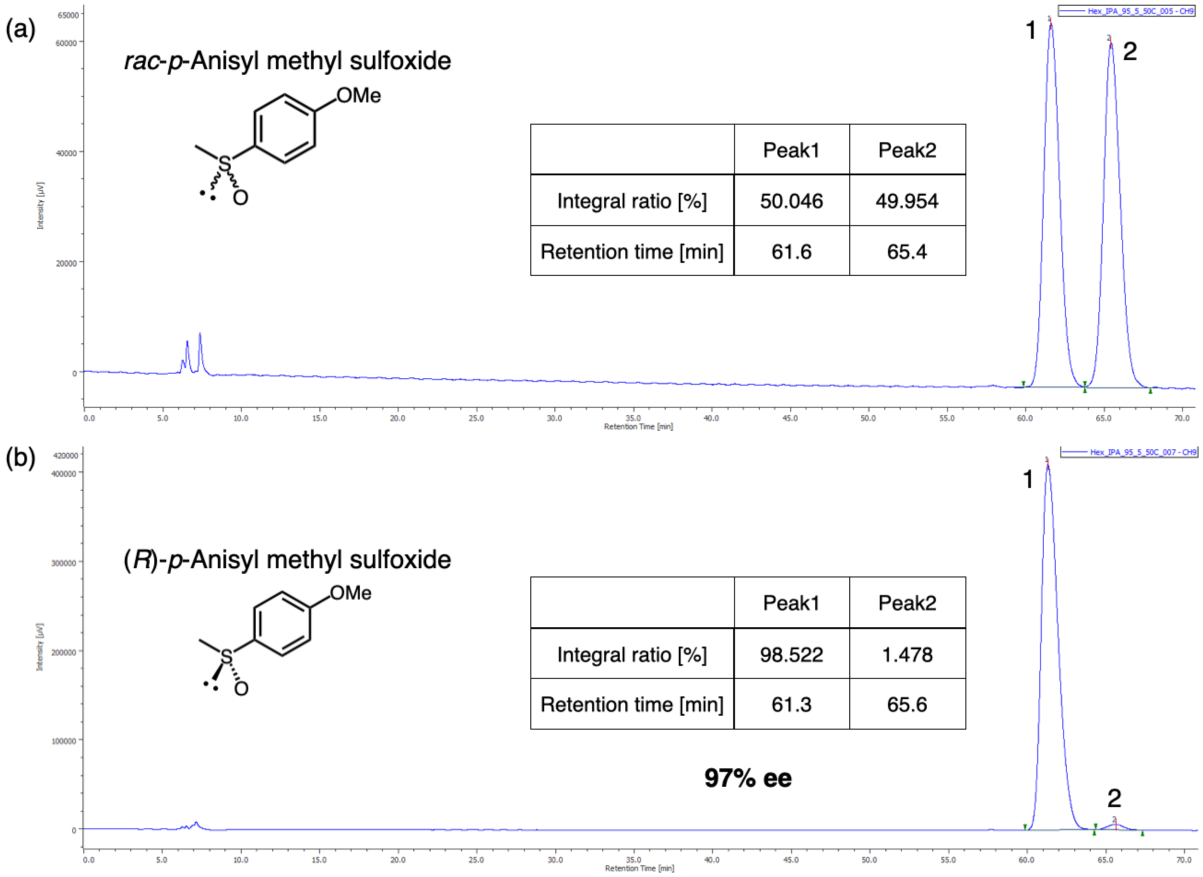

(a)

	Peak1	Peak2
Integral ratio [%]	50.046	49.954
Retention time [min]	61.6	65.4

(b)

	Peak1	Peak2
Integral ratio [%]	98.522	1.478
Retention time [min]	61.3	65.6

Supplementary Fig. 76 Chiral HPLC traces of (a) *rac*-*p*-anisyl methyl sulfoxide and (b) (*R*)-*p*-anisyl methyl sulfoxide (CHIRALPAK OD-H column, *n*-hexane:2-propanol = 97:3, 50 °C, 0.5 mL/min, detected with photodiode array at 240 nm).

5.8 Synthesis of (*R*)-*o*-anisyl methyl sulfoxide[12]

The compound was synthesised with reference to a report of similar compounds[12]. Mg turnings (270.4 mg, 11.1 mmol, 1.0 equiv.) was placed in an oven-dried flask and dry THF (12.3 mL) was poured into the flask under argon atmosphere. To this suspension was added with stirring 1,2-dibromoethane (100 μL, 1.16 mmol, 0.10 equiv.) as a reaction initiator. *o*-Anisylbromide (1130 μL, 9.03 mmol, 0.81 equiv.) was then added to the solution dropwise over 20 min. The reaction mixture was heated at reflux for 2 h to afford a grey suspension of the desired Grignard reagent, *o*-anisylmagnesium bromide.

(*R*)-MeS(O)ODAG (a mixture of (*R*)-MeS(O)ODAG and diacetone-D-glucose estimated from ^{1}H NMR to be in the molar ratio of 5:4; 591 mg, 1.08 mmol for (*R*)-MeS(O)ODAG) was placed in an oven-dried flask and dry toluene (45 mL) was poured into the flask under argon atmosphere. After cooling to 0 °C, the suspension of *o*-anisylmagnesium bromide was added to the solution dropwise over 16 min. The reaction mixture was stirred at 0 °C for 60 min. After quenching with sat. NH_4Cl aq., the reaction mixture was diluted with CH_2Cl_2. The organic layer was dried over anhydrous sodium sulfate and evaporated. The crude product was then purified by silica gel column chromatography (eluent: EtOAc/*n*-hexane = 3:1). After removal of the solvent, the resulting colourless solid was recrystallised from *n*-hexane to afford (*R*)-*o*-anisyl methyl sulfoxide as colourless crystals (96.3 mg, 0566 mmol, 52%, > 99% ee).

^{1}H NMR (500 MHz, $CDCl_3$, 300 K): δ 7.83 (dd, J = 8.0, 2.0 Hz, 1H), 7.46 (dt, J = 8.0, 1.5 Hz, 1H), 7.20 (dt, J = 7.5, 0.5 Hz, 1H), 6.92 (d, J = 8.0 Hz, 1H), 3.89 (s, 3H), 2.28 (s, 3H). ^{13}C NMR (126 MHz, $CDCl_3$, 300 K): δ 154.9, 133.2, 132.0, 124.7, 121.8, 110.7, 55.8, 41.3. MS (ESI-TOF): m/z = 171.04 as $[AnisS(O)Me{\cdot}H]^{+}$ (calcd 171.05). $[\alpha]$ = +341.2 (c = 0.002623, 20.6 °C, acetone). The obtained value of the specific rotation was consistent with the reported value ($[\alpha]$ = +340 (c = 1, acetone, 95.3% ee for (*R*)-*o*-anisyl methyl sulfoxide))[14].

Crystal data for (*R*)-*o*-anisyl methyl sulfoxide: $C_8H_{10}O_2S$, F_w = 170.22, crystal dimensions 0.2 × 0.07 × 0.05 mm^3, monoclinic, space group *P*21, a = 5.9039(7), b = 9.1343(15), c = 8.3223(10) Å, β = 110.587(4)°, V = 420.14(10) Å^3, Z = 2, ρ_{calcd} = 1.346 g cm^{-3}, μ = 3.31 cm^{-1}, T = 113(2) K, λ(MoKα) = 0.71075 Å, $2\theta_{max}$ = 54.930°, 4131/1895 reflections collected/unique (R_{int} = 0.0515), R_1 = 0.0491 ($I > 2\sigma(I)$), wR_2 = 0.1726 (for all data), Flack parameter = –0.05(5), GOF = 1.321, largest diff. peak and hole 0.917/–1.190 eÅ^{-3}. CCDC deposit number 2190134.

PLAT340_ALERT_3_B Low Bond Precision on C-C Bonds … 0.013333 Ang.

Response: The average intensity of reflections is relatively low due to small size of the crystal.

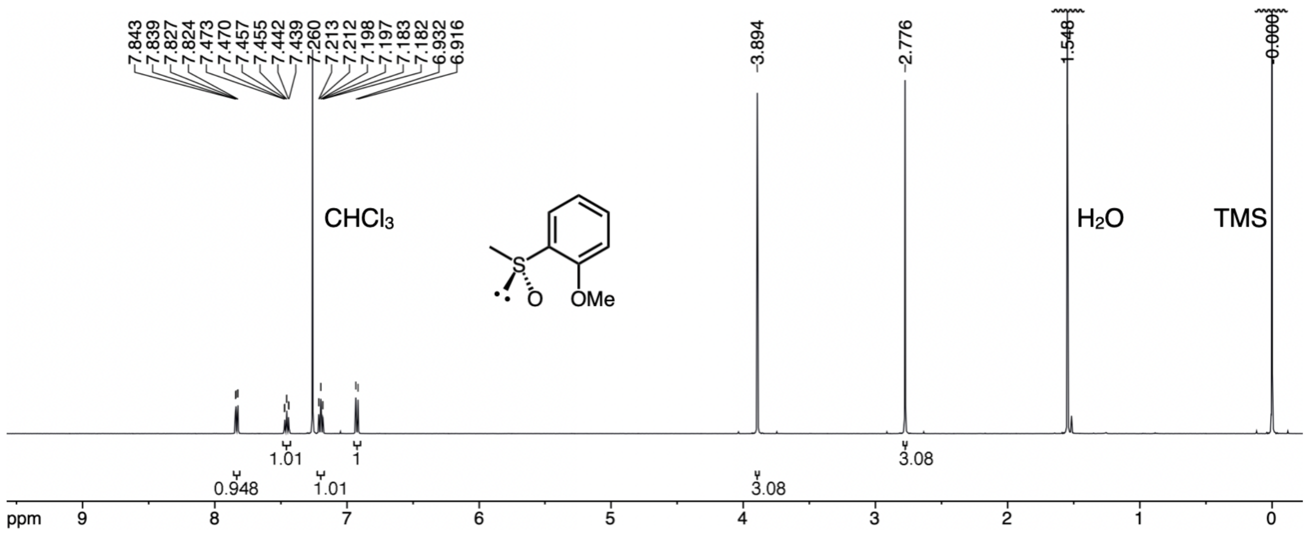

Supplementary Fig. 77 ^{1}H NMR spectrum of (*R*)-*o*-anisyl methyl sulfoxide (500 MHz, $CDCl_3$, 300 K).

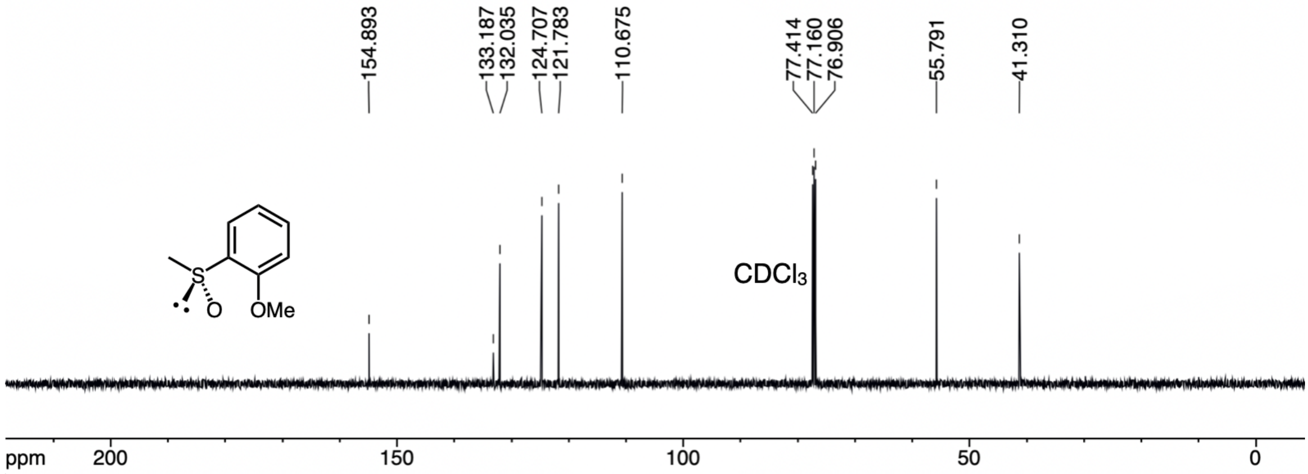

Supplementary Fig. 78 ^{13}C NMR spectrum of (*R*)-*o*-anisyl methyl sulfoxide (126 MHz, $CDCl_3$, 300 K).

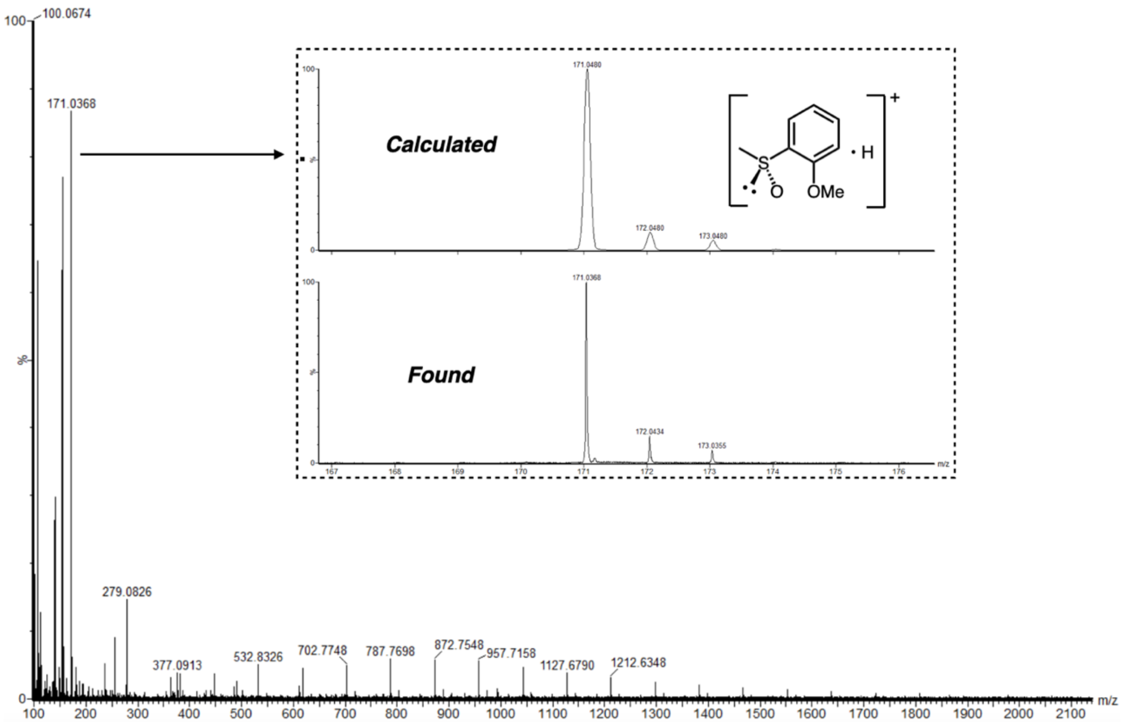

Supplementary Fig. 79 ESI-TOF mass spectrum of (*R*)-*o*-anisyl methyl sulfoxide (positive, MeOH).

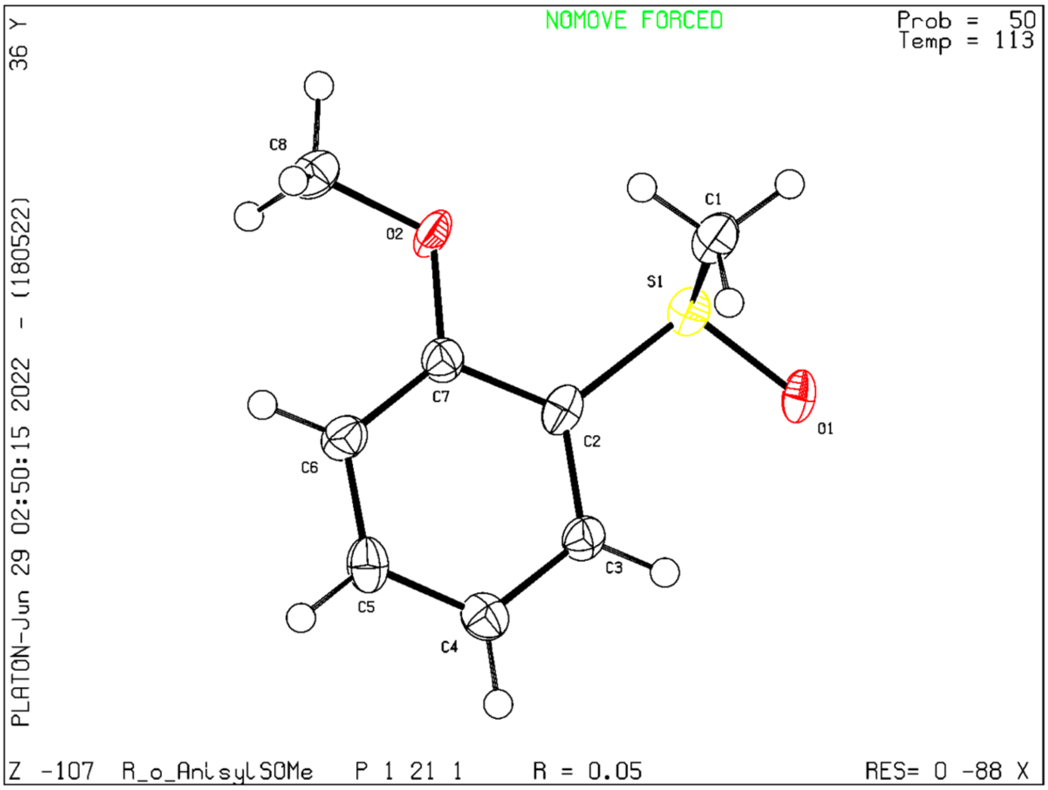

Supplementary Fig. 80 ORTEP drawing of (*R*)-*o*-anisyl methyl sulfoxide at the 50% probability level. Colour: C black, O red and S yellow. CCDC deposit number of (*R*)-*o*-anisyl methyl sulfoxide is 2190134. This figure was produced by the checkCIF report of the International Union of Crystallography.

For HPLC analysis of the product, *rac*-*o*-anisyl methyl sulfoxide was prepared by racemisation of (*R*)-*o*-anisyl methyl sulfoxide[15]. To a 1,4-dioxane solution (0.13 mL) of (*R*)-*o*-anisyl methyl sulfoxide (3.9 mg, 23 μmol) was added aqueous hydrogen chloride (0.06 mL, 12 M). The reaction solution was stirred at room temperature for 15 h under argon atmosphere. Then, the reaction solution was neutralised with aqueous Na_2CO_3 (0.5 mL, 1.4 M) and the sulfoxide was extracted with diethyl ether. The organic layer was dried over anhydrous sodium sulfate and evaporated to afford *rac*-*o*-anisyl methyl sulfoxide (2.0 mg, 12 μmol, 51%).

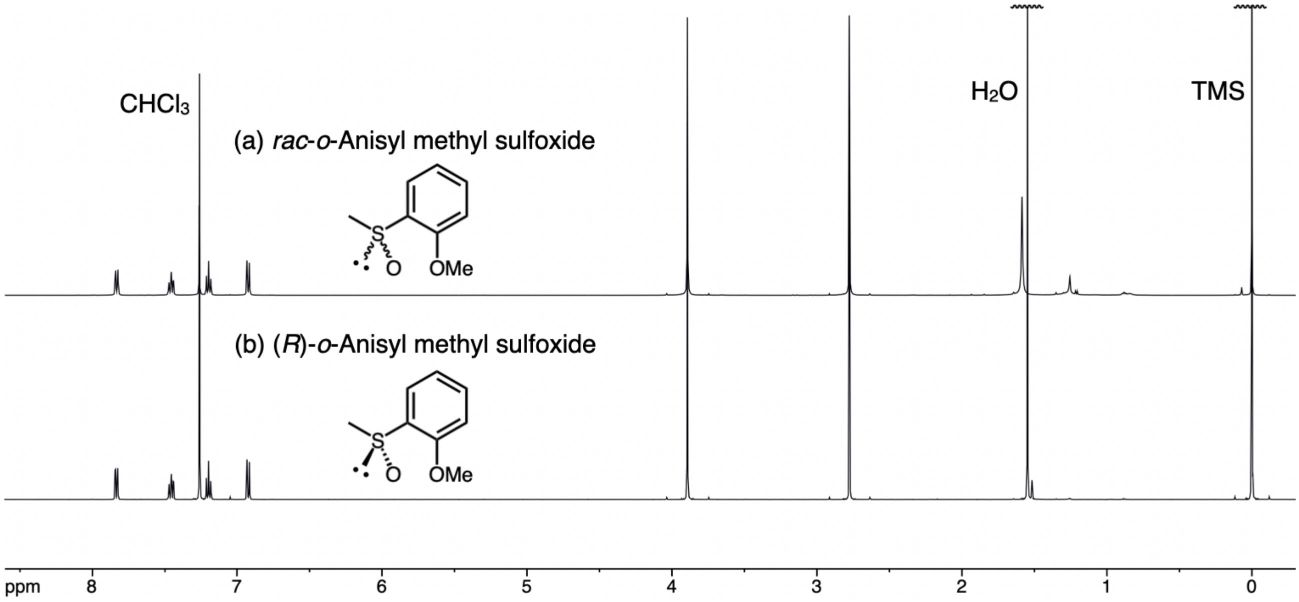

Supplementary Fig. 81 ^{1}H NMR spectra of (a) *rac*-*o*-anisyl methyl sulfoxide and (b) (*R*)-*o*-anisyl methyl sulfoxide (500 MHz, $CDCl_3$, 300 K).

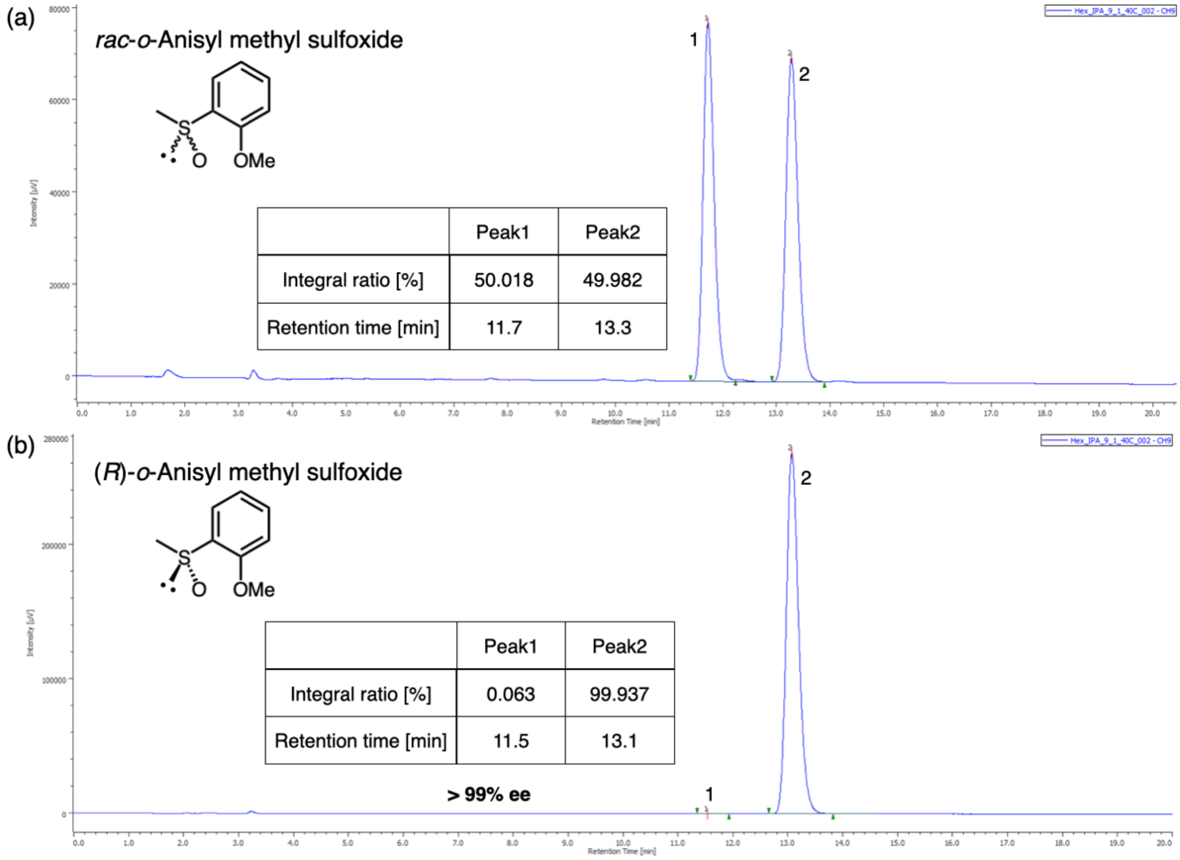

Supplementary Fig. 82 Chiral HPLC traces of (a) *rac*-*o*-anisyl methyl sulfoxide and (b) (*R*)-*o*-anisyl methyl sulfoxide (CHIRALPAK AD-H column, *n*-hexane:2-propanol = 90:10, 40 °C, 1.0 mL/min, detected with photodiode array at 240 nm).

5.9 Synthesis of 2-propanesulfinyl chloride[11]

Diisopropyl disulfide (1.5034 g, 10.0 mmol, 1.0 equiv.) and acetic acid (1.2117 g, 20.2 mmol, 2.0 equiv.) were placed in a flask under argon atmosphere. This reaction mixture was stirred and then cooled to –20 °C. To this solution was added sulfuryl chloride (5.017 g, 37.2 mmol, 3.7 equiv.) dropwise over 10 min, and this reaction mixture was stirred at –20 °C for 3 h. The solution was coloured yellow during addition of 2-propanesulfinyl chloride and then changed to pale yellow. The reaction mixture was allowed to return to room temperature for 1.5 h, during which time sulfur dioxide and hydrochloric acid gases were observed. This reaction mixture was then warmed to 35 °C and stirred for 1 h. Removal of acetyl chloride from the mixture at 49.5 torr under reduced pressure at room temperature afforded crude 2-propanesulfinyl chloride as a pale yellow liquid (2.3773 g, 18.8 mmol, 94%). Since 2-propanesulfinyl chloride is known to be very unstable, it was used for the next reaction without further purification.

^{1}H NMR (500 MHz, $CDCl_3$, 300 K): δ 3.30 (septet, J = 7.0 Hz, 1H), 1.46 (d, J = 7.0 Hz, 3H), 1.44 (d, J = 7.0 Hz, 3H).

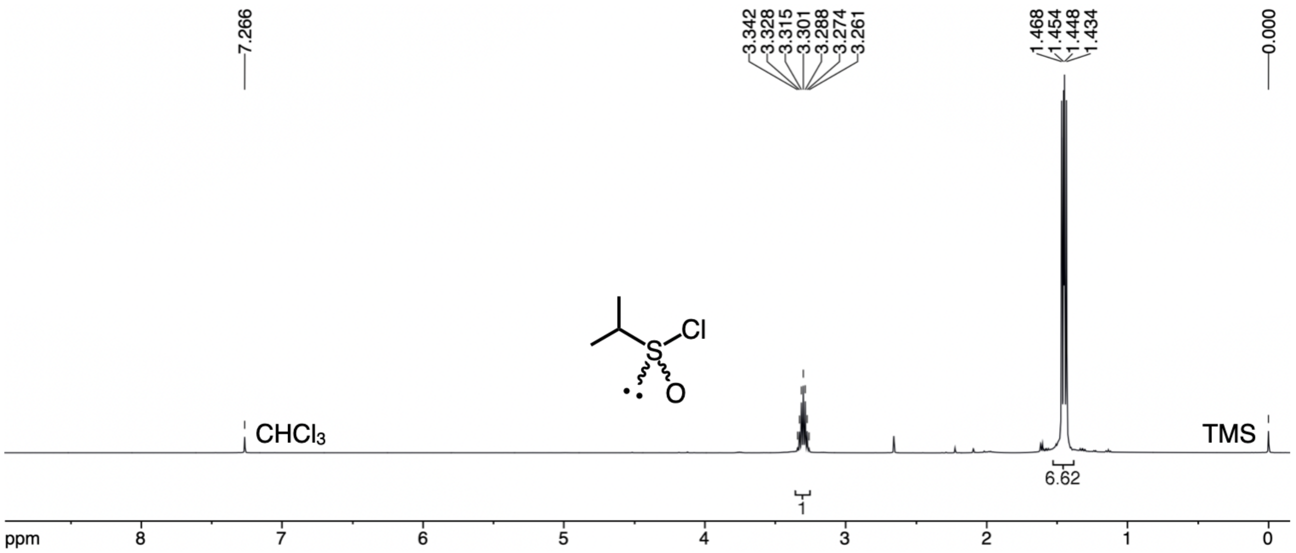

Supplementary Fig. 83 ^{1}H NMR spectrum of the crude 2-propyl sulfinyl chloride (500 MHz, $CDCl_3$, 300 K).

5.10 Synthesis of 1,2,5,6-di-*O*-2-propylidene-*α*-D-glucofuranosyl (*R*)-2-propylsulfinate[12]

Diacetone-D-glucose (2.4462 g, 9.40 mmol, 1.0 equiv.) and pyridine (0.90 mL, 11 μmol, 1.2 equiv.) were placed in a flask and dry THF (45 mL) was poured into the flask under argon atmosphere. After cooling to –78 °C, a THF solution (5 mL) of 2-propyl sulfinyl chloride (2.3773 g, 18.8 mmol, 2.0 equiv.) was added to the reaction mixture dropwise over 15 min at –78 °C. This reaction mixture was stirred at –78 °C for 3 h and a colourless precipitate was formed during the reaction. After bringing the reaction mixture to room temperature, the reaction mixture was quenched with water and diluted with CH_2Cl_2. The organic layer was washed with hydrochloric acid aq. (1 M), sodium hydrogen carbonate aq. (3wt%) and saturated NaCl aq., and then dried over anhydrous sodium sulfate. After evaporation, the resulting pale yellow oil was purified by column chromatography (eluent: diethyl ether:*n*-hexane = 3:7) to afford (*R*)-^{i}PrS(O)ODAG as a colourless oil (1.1814 g, 3.37 mmol, 36%).
^{1}H NMR (500 MHz, $CDCl_3$, 300 K): δ 5.91 (d, J = 3.5 Hz, 1H), 4.80 (d, J = 3.5 Hz, 1H), 4.71 (d, J = 1.5 Hz, 1H), 4.14 (m, 3H), 3.97 (dd, J = 8.0, 4.0 Hz, 1H), 2.82 (septet, J = 7.0 Hz, 1H), 1.50 (s, 3H), 1.42 (s, 3H), 1.31 (s, 3H), 1.31 (s, 3H), 1.27 (d, J = 7.0 Hz, 3H), 1.27 (d, J = 7.0 Hz, 3H). MS (ESI-TOF): m/z = 373.10 as [^{i}PrS(O)ODAG·Na]$^+$ (calcd 373.13).

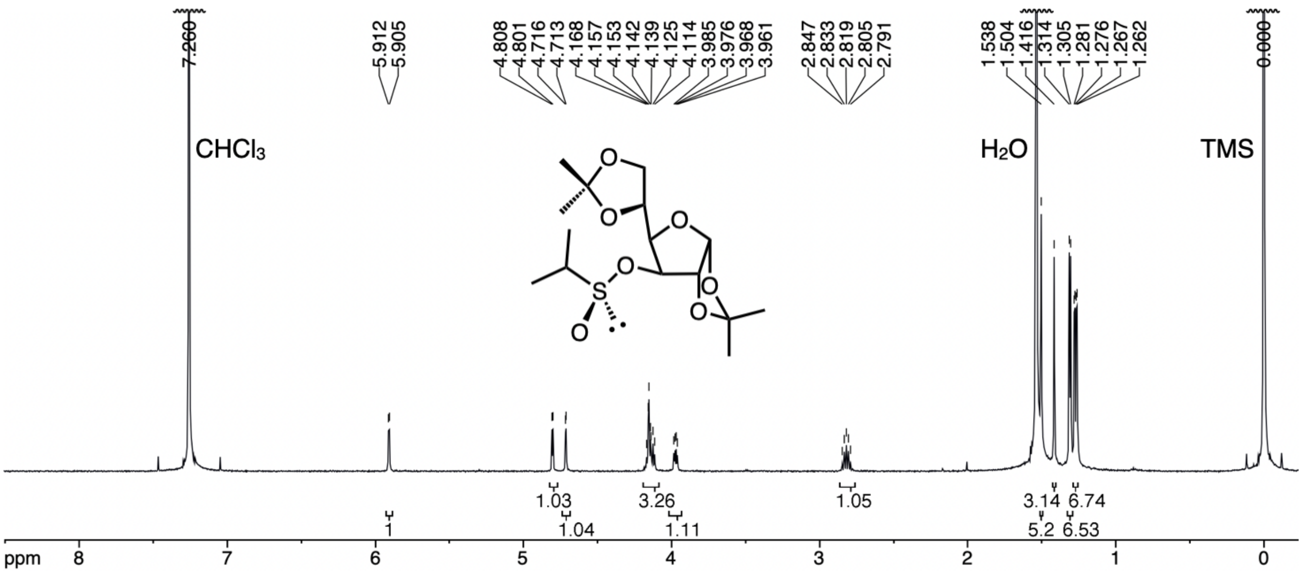

Supplementary Fig. 84 ^{1}H NMR spectrum of (*R*)-^{i}PrS(O)ODAG (500 MHz, $CDCl_3$, 300 K).

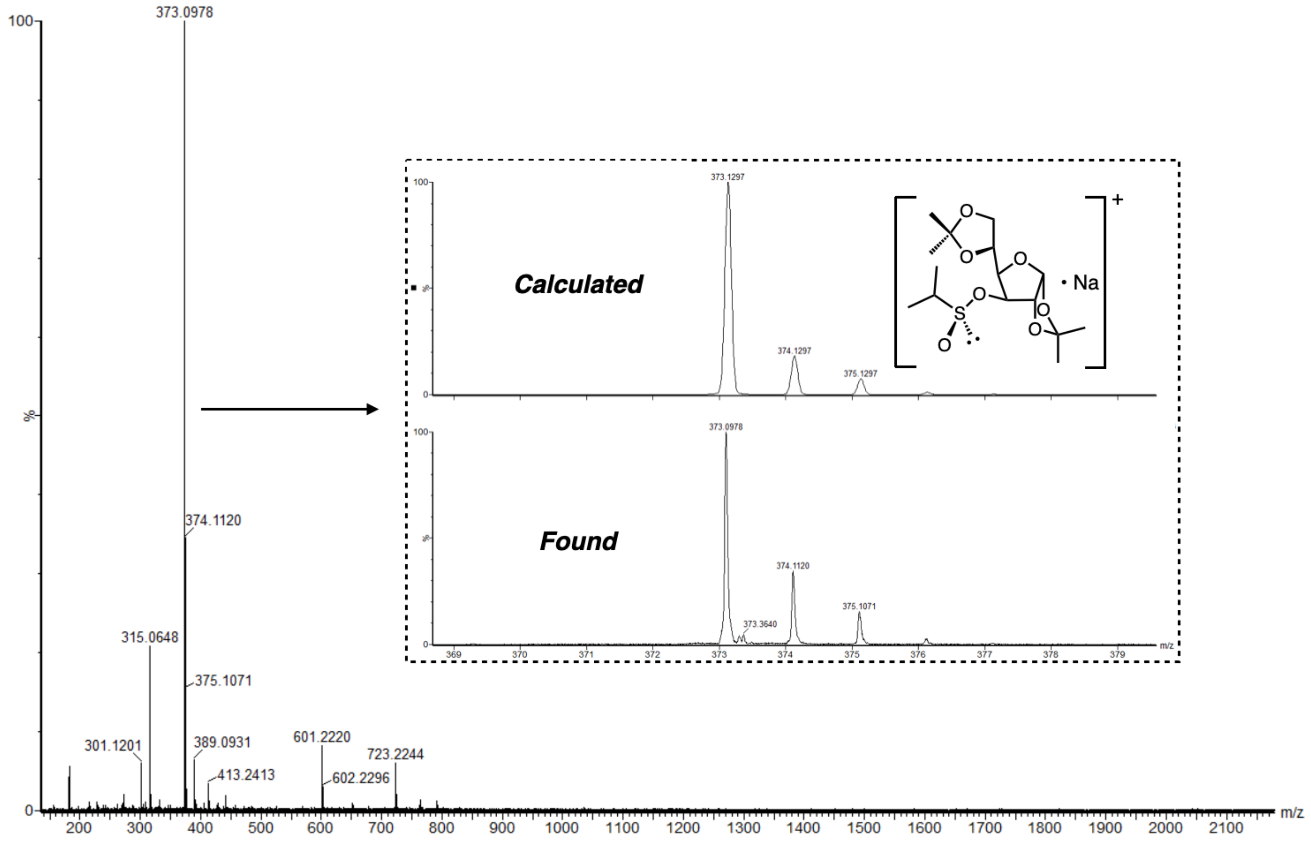

Supplementary Fig. 85 ESI-TOF mass spectrum of (*R*)-^{i}PrS(O)ODAG (positive, MeOH).

5.11 Synthesis of (*R*)-isopropyl *p*-tolyl sulfoxide[12]

Mg turnings (48.7 mg, 2.00 mmol, 1.0 equiv.) was placed in an oven-dried flask and dry diethyl ether (2.0 mL) was poured into the flask under argon atmosphere. *p*-Bromotoluene (341.2 mg, 1.99 mmol, 1.0 equiv.) was added with stirring to the solution over 6 min. 1,2-Dibromoethane (55 µL, 0.64 mmol, 0.32 equiv.) was then added to the mixture to accelerate the formation of the desired Grignard reagent, *p*-tolylmagnesium bromide.

(*R*)-^{i}PrS(O)ODAG (155.8 mg, 0.445 mmol) was added to an oven-dried flask and the atmosphere was changed to argon. After dry toluene (10 mL) was added to the flask the reaction solution was cooled to 0 °C. To this solution, the suspension of *p*-tolylmagnesium bromide was added dropwise over 5 min and this suspension was stirred for 75 min at 0 °C. After quenched with sat. NH_4Cl aq., the reaction solution was diluted with CH_2Cl_2. The organic layer was dried over anhydrous sodium sulfate and evaporated. Then, the crude product was purified by silica gel column chromatography (eluent: *n*-hexane to EtOAc:*n*-hexane = 1:2 to 1:1). After removal of the solvent (*R*)-2-propyl *p*-tolyl sulfoxide was obtained as a colourless oil (13.3 mg, 0.073 mmol, 16%, 76% ee determined by specific rotation). ^{1}H NMR (500 MHz, $CDCl_3$, 300 K): δ 7.48 (d, J = 8.0 Hz, 2H), 7.31 (d, J = 8.0 Hz, 2H), 2.81 (septet, J = 7.0 Hz, 2H), 2.41 (s, 3H), 1.20 (d, J = 7.0 Hz, 3H), 1.15 (d, J = 7.0 Hz, 3H). MS (ESI-TOF): m/z = 205.05 as [^{i}PrS(O)Tol·Na]$^+$ (calcd 205.07). [α] = +144 (c = 0.00093, 22.4 °C, ethanol). The reported value of specific rotation was [α] = +188 (c = 4.0, ethanol, 100% ee for (*R*)-2-propyl *p*-tolyl sulfoxide)[12].

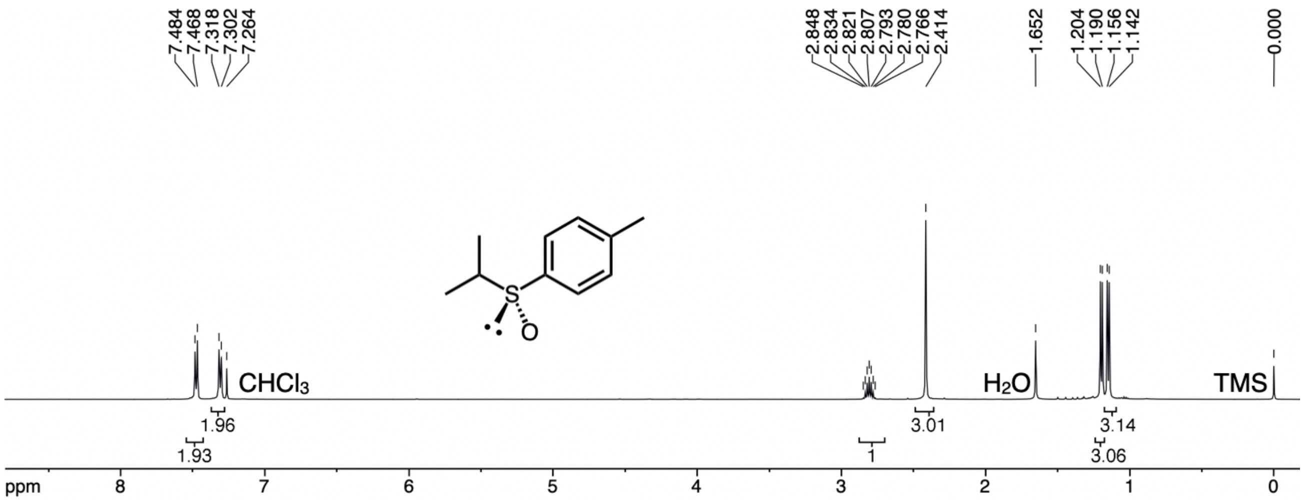

Supplementary Fig. 86 ^{1}H NMR spectrum of (*R*)-2-propyl *p*-tolyl sulfoxide (500 MHz, $CDCl_3$, 300 K).

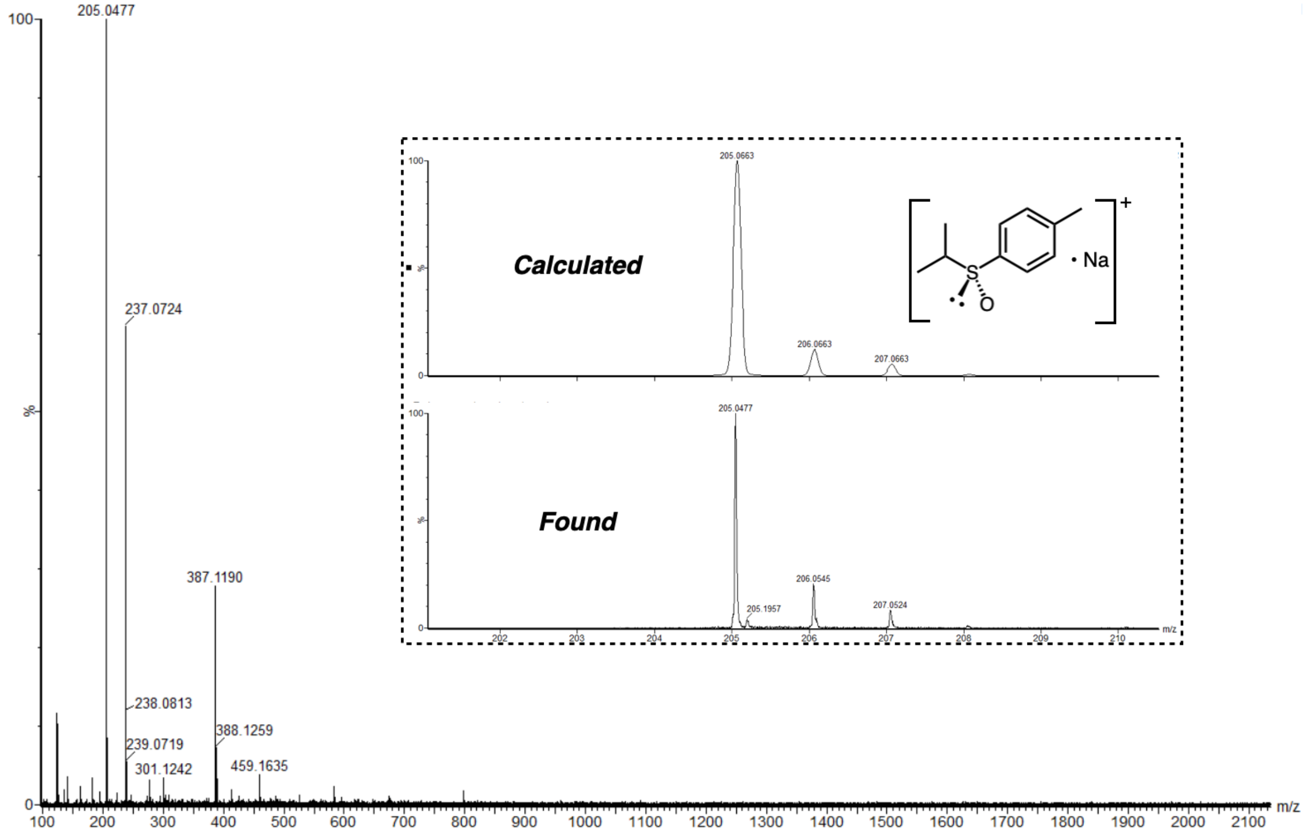

Supplementary Fig. 87 ESI-TOF mass spectrum of (*R*)-2-propyl *p*-tolyl sulfoxide (positive, MeOH).

6. Asymmetric synthesis of 1_{tight}

6.1 Synthesis of (*M*)-enantio-enriched 1_{tight} from (*S*)-3

A CH_2Cl_2 solution (0.5 mL) of (*S*)-**3** (96% ee, 93.5 mg, 513 μmol, 65 equiv.) was added to a CH_2Cl_2 solution (1.0 mL) of [Pd(^{t}Bu$_2$bpy)(OH_2)$_2$](OTf)$_2$·(H_2O)$_2$ (19.0 mg, 25.5 μmol, 3.2 equiv.). To this solution was added a CH_2Cl_2 solution (1.0 mL) of **L** (5.00 mg, 7.93 μmol, 1.0 equiv.) at –70 °C. This reaction mixture was stirred at –70 °C for 4 h. After bringing the reaction mixture to room temperature, Et_2O was added to the reaction mixture and filtered. The filtrate was evaporated under reduced pressure to afford (*S*)-**3** (87.9 mg, 482 μmol, 94% recovery yield, 95% ee). The precipitate was dissolved in CH_2Cl_2 and filtered to remove insoluble residue. The filtrate was evaporated and dried *in vacuo*. After the resulting solid was washed with $CHCl_3$, (*M*)-enantio-enriched $\mathbf{1}_{tight}$ was obtained as a pale yellow solid (3.15 mg, 1.19 μmol, 15% yield, 25% ee based on the deconvolution analysis using an iNMR software). The enantiomeric excess was estimated using Δ-**4** as a chiral shift reagent.

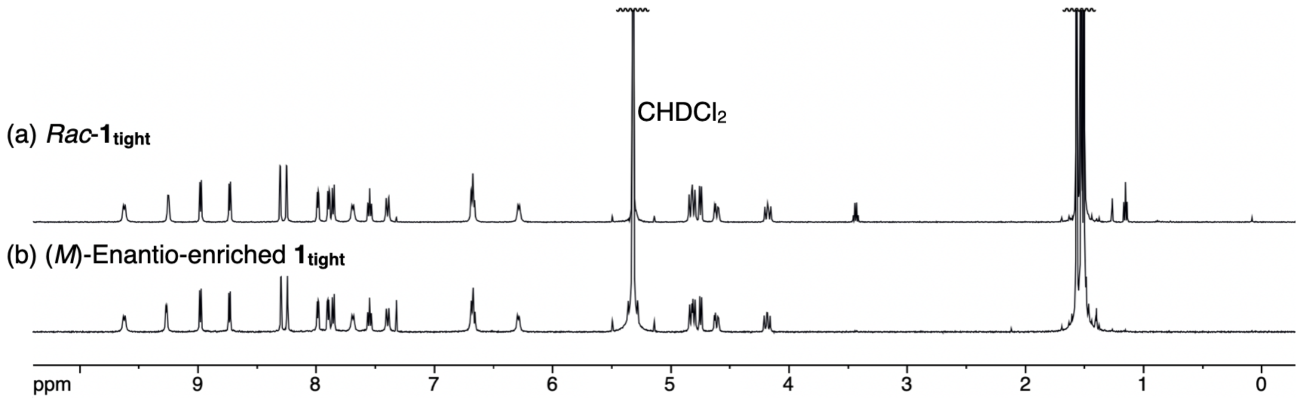

Supplementary Fig. 88 ^{1}H NMR spectra of (a) *rac*-$\mathbf{1}_{tight}$ and (b) (*M*)-enantio-enriched $\mathbf{1}_{tight}$ synthesised with (*S*)-**3** (500 MHz, CD_2Cl_2, 300 K).

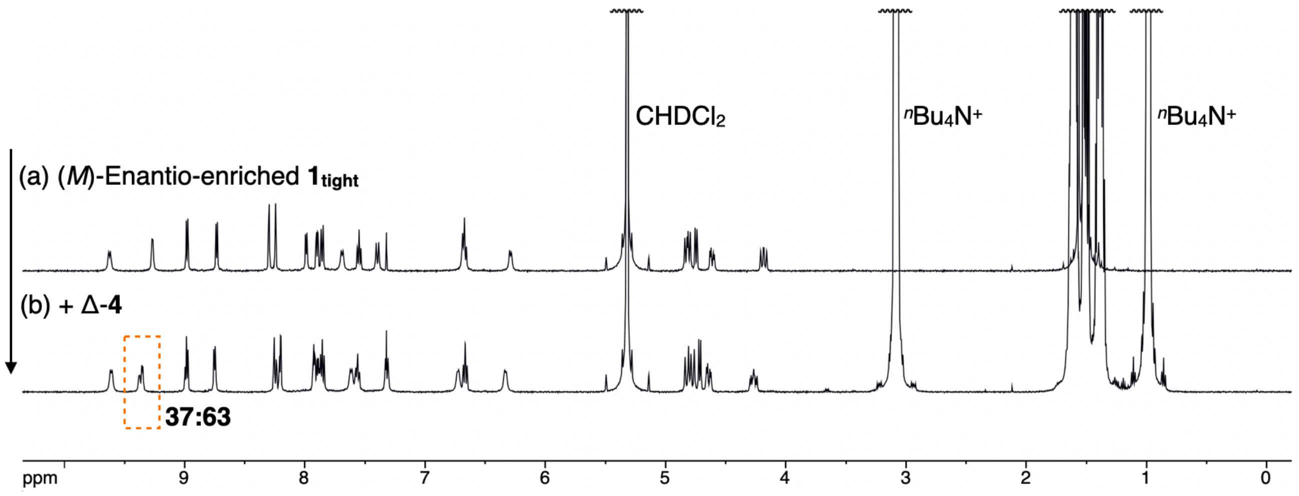

Supplementary Fig. 89 (a),(b) ^{1}H NMR spectra of aforementioned (*M*)-enantio-enriched $\mathbf{1}_{tight}$ (a) before and (b) after addition of Δ-**4** as a chiral shift reagent (500 MHz, CD_2Cl_2, 300 K).

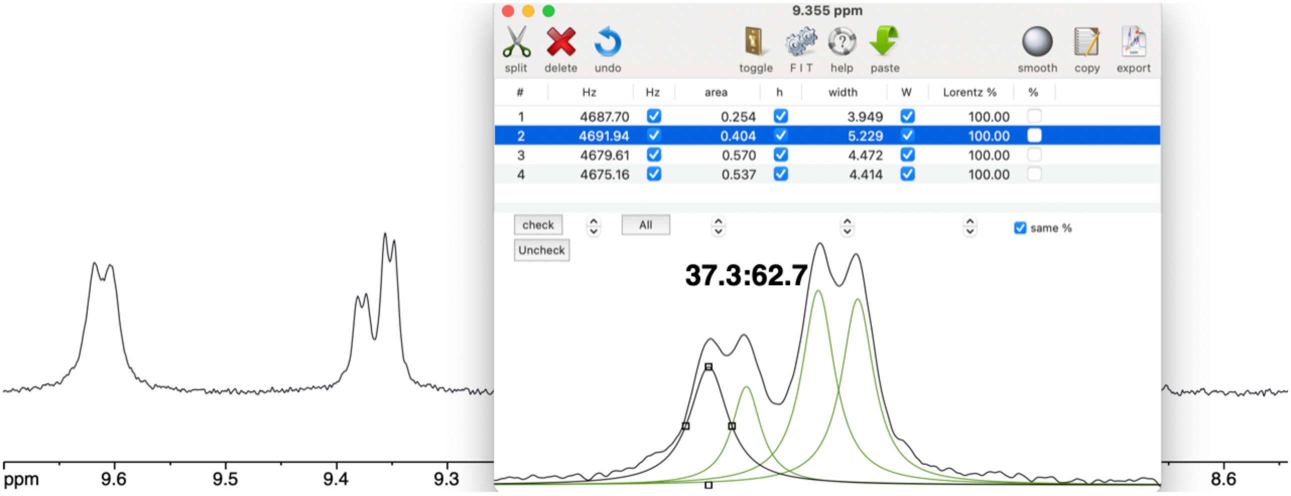

Supplementary Fig. 90 Deconvolution analysis of ^{1}H NMR spectrum of the (*M*)-enantio-enriched **1**$_{\mathbf{tight}}$ with Δ-**4**, shown in Supplementary Fig. 89b.

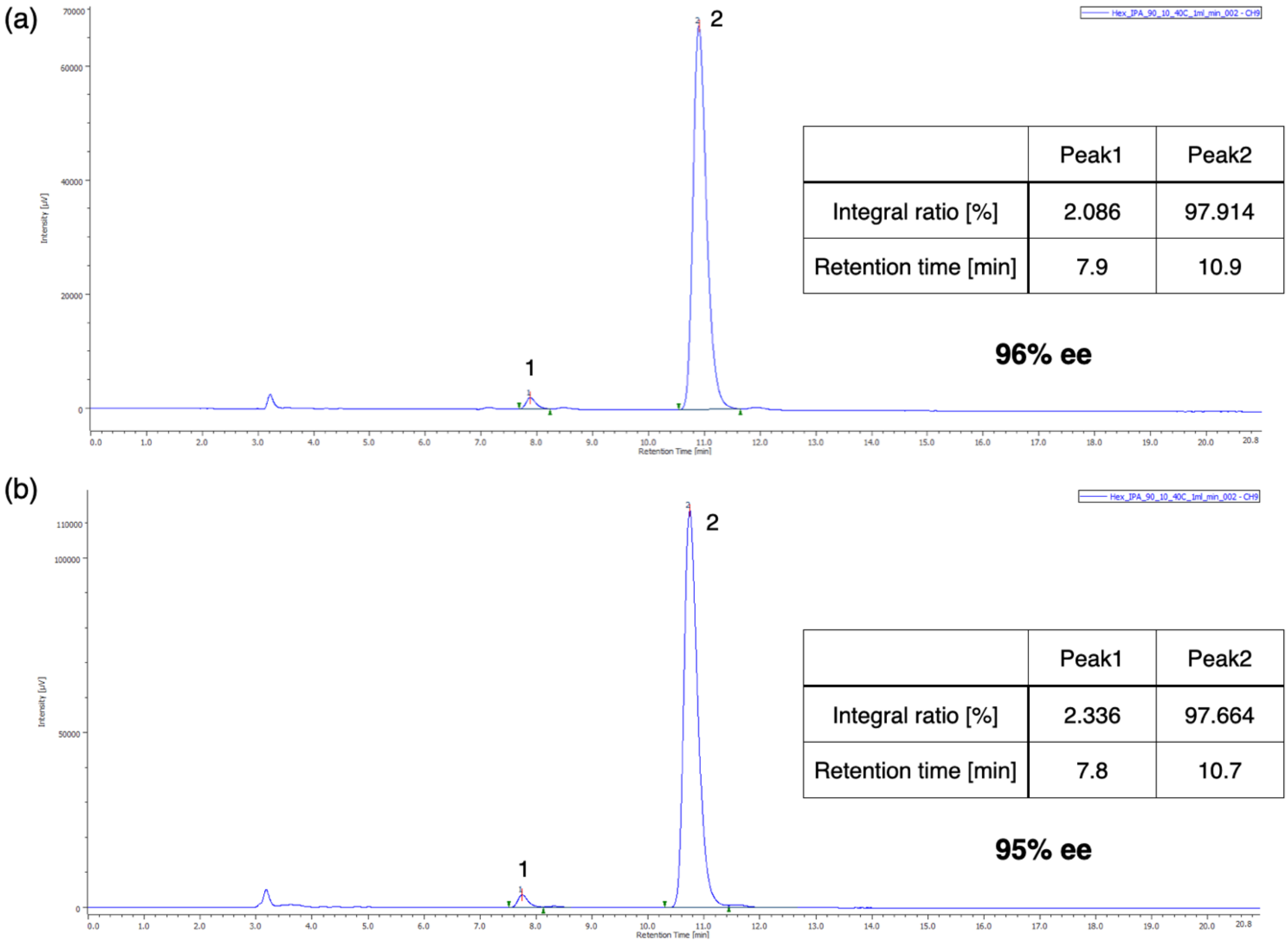

Supplementary Fig. 91 Chiral HPLC traces of (*S*)-**3** (a) before and (b) after use for asymmetric synthesis of **1**$_{\mathbf{tight}}$ (CHIRALPAK OD-H column, eluent: *n*-hexane:2-propanol = 90:10, 40 °C, 1 mL/min, detected with photodiode array at 240 nm).

6.2 Synthesis of (*P*)-enantio-enriched $\mathbf{1_{tight}}$ from (*R*)-3

A CH_2Cl_2 solution (0.2 mL) of (*R*)-**3** (96% ee, 37.5 mg, 206 µmol, 65 equiv.) was added to a CH_2Cl_2 solution (0.4 mL) of [Pd(^{t}Bu$_2$bpy)(OH$_2$)$_2$](OTf)$_2$·(H$_2$O)$_2$ (7.64 mg, 10.3 µmol, 3.2 equiv.). To this solution was added a CH_2Cl_2 solution (0.4 mL) of **L** (2.01 mg, 3.19 µmol, 1.0 equiv.) at –70 °C. This reaction mixture was stirred at –70 °C for 4 h. After bringing the reaction mixture to room temperature, Et_2O was added to the reaction mixture and filtered. The filtrate was evaporated under reduced pressure to afford (*R*)-**3** (33.5 mg, 184 µmol, 89% recovery yield, 95% ee). The precipitate was dissolved in CH_2Cl_2 and filtered to remove insoluble residue. The filtrate was evaporated and dried *in vacuo*. After the resulting solid was washed with $CHCl_3$, (*P*)-enantio-enriched $\mathbf{1_{tight}}$ was obtained as a pale yellow solid (1.13 mg, 0.426 µmol, 13% yield, 31% ee based on the deconvolution analysis using an iNMR software). The enantiomeric excess was estimated using Δ-**4** salt as a chiral shift reagent.

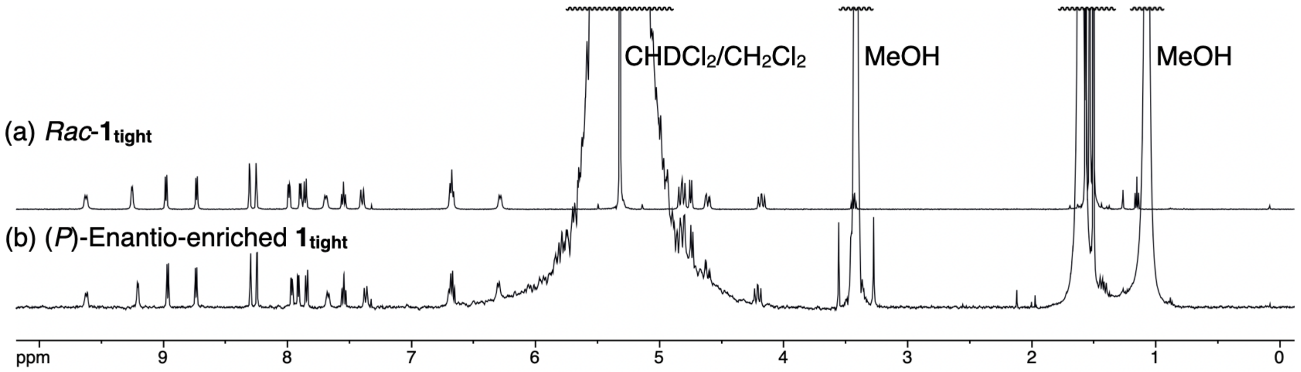

Supplementary Fig. 92 ^{1}H NMR spectra of (a) *rac*-$\mathbf{1_{tight}}$ and (b) (*P*)-enantio-enriched $\mathbf{1_{tight}}$ synthesised with (*R*)-**3** (500 MHz, CD_2Cl_2/CH_2Cl_2, 300 K). For the analysis of (*P*)-enantio-enriched $\mathbf{1_{tight}}$, a mixture of CD_2Cl_2 and CH_2Cl_2 was used as the solvent to check its stability in CH_2Cl_2 for spectrochemical analysis that contains methanol as a stabiliser.

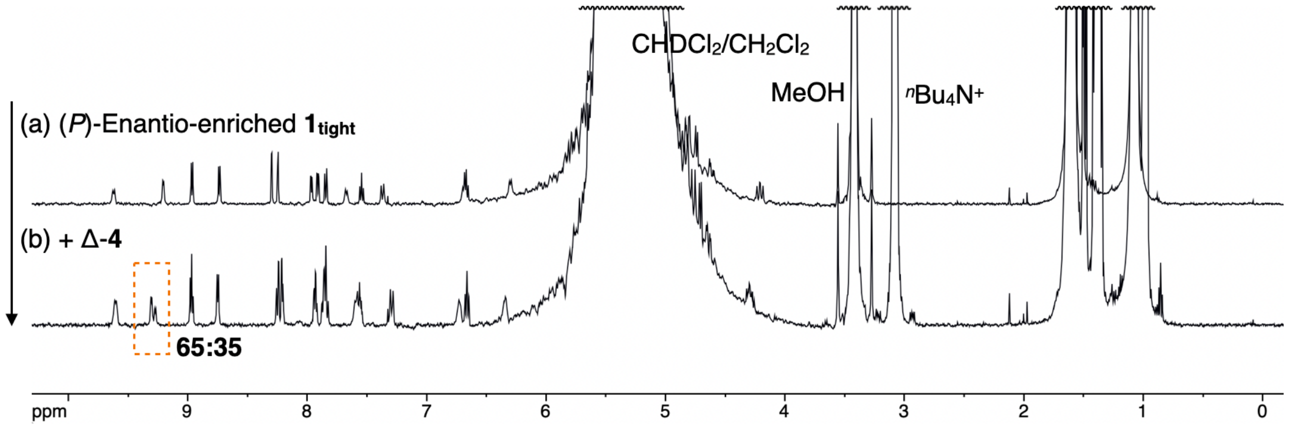

Supplementary Fig. 93 (a),(b) ^{1}H NMR spectra of aforementioned (*P*)-enantio-enriched $\mathbf{1_{tight}}$ (a) before and (b) after addition of Δ-**4** as a chiral shift reagent (500 MHz, CD_2Cl_2/CH_2Cl_2, 300 K). In this

measurement, a mixture of CD_2Cl_2 and CH_2Cl_2 was used as the solvent to check the stability in CH_2Cl_2 for spectrochemical analysis that contains methanol as a stabiliser.

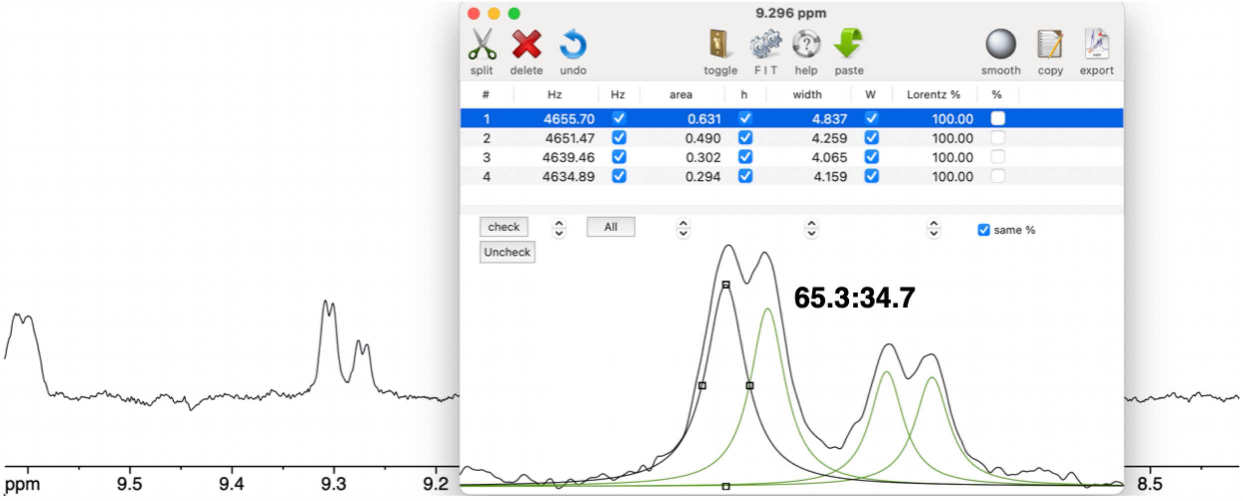

Supplementary Fig. 94 Deconvolution analysis of ^{1}H NMR spectrum of the (*P*)-enantio-enriched **1**$_{\mathbf{tight}}$ with Δ-**4**, shown in Supplementary Fig. 93b.

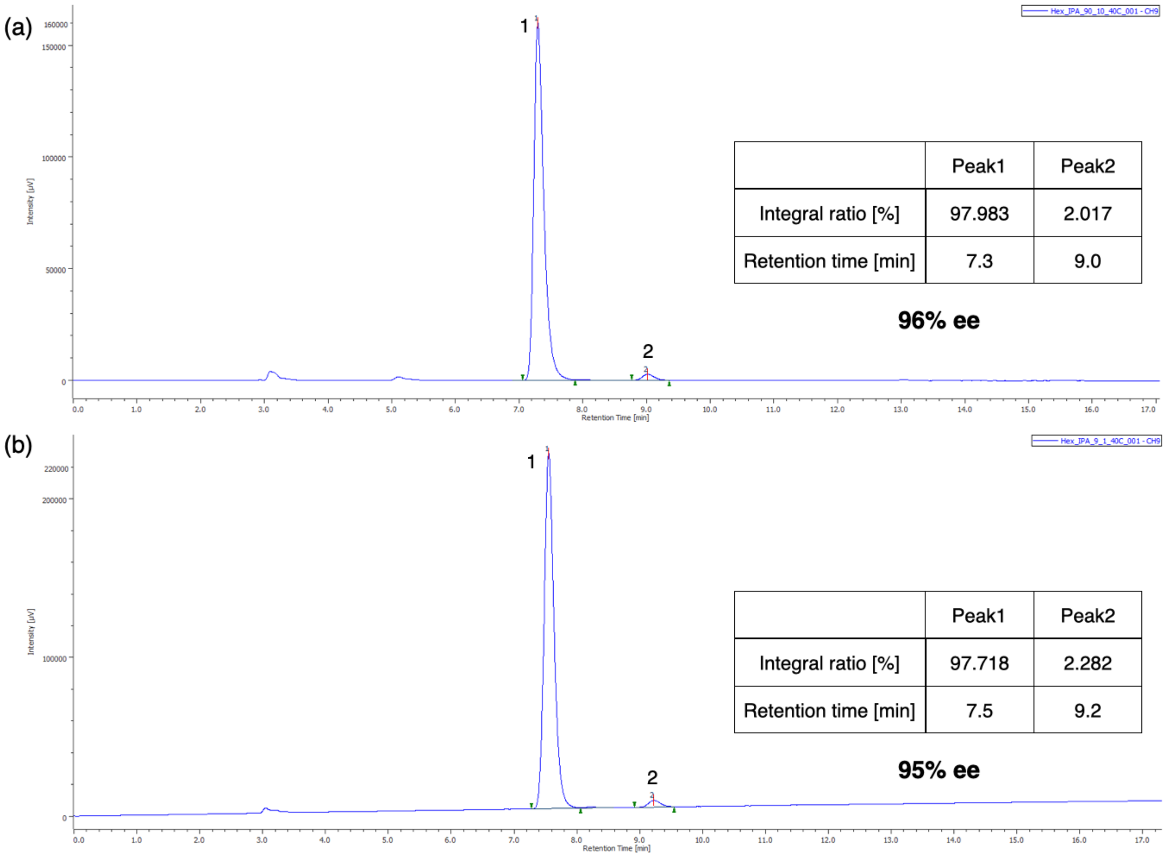

Supplementary Fig. 95 Chiral HPLC traces of (*R*)-**3** (a) before and (b) after use for asymmetric synthesis of **1**$_{\mathbf{tight}}$ (CHIRALPAK OD-H column, eluent: *n*-hexane:2-propanol = 90:10, 40 °C, 1 mL/min, detected with photodiode array at 240 nm).

6.3 Summary of asymmetric synthesis of $\mathbf{1}_{\mathbf{tight}}$

For the asymmetric synthesis of $\mathbf{1}_{\mathbf{tight}}$, several chiral sulfoxides were examined as shown in the Supplementary Fig. 96. These reactions were conducted at –70 °C because the enantiomeric excess at this temperature was expected to become higher than that at room temperature. Since the preliminary asymmetric reaction with (*R*)-2-propyl *p*-tolyl sulfoxide gave much lower selectivity than (*R*)-methyl *p*-tolyl sulfoxide, we mainly examined substitution effects of the aromatic ring of the chiral sulfoxides through this study. Among the screening reaction with various chiral sulfoxides, **3** gave the higher yield and enantiomeric excess and therefore was used as an additive for asymmetric synthesis of $\mathbf{1}_{\mathbf{tight}}$.

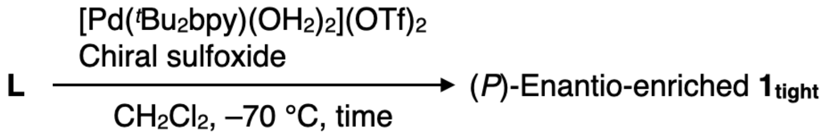

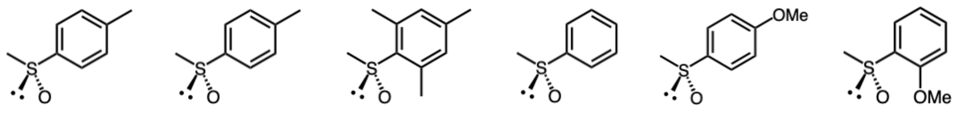

Chiral sulfoxides						
equiv. to Pd salt	2.2	20	20	20	20	20
Reaction time	4	4	4	4	24	4
Enantiomeric excess [%]	17	64	31*	17	17	–21**
Yield [%]	22	Trace	15	6.5	3.7	12

Supplementary Fig. 96 Asymmetric synthesis of $\mathbf{1}_{\mathbf{tight}}$ and the screening results of chiral sulfoxides to optimise the asymmetric synthesis. *: Enantiomeric excess of enantio-enriched $\mathbf{1}_{\mathbf{tight}}$ synthesised with (*R*)-**3** was estimated by the integral ratio of the outer amine protons (H_a, around 9.3 ppm), while that with other (*R*)-sulfoxides were estimated by one bipyridine protons (H_s, 8.2 ppm) because the intensity or splitting of the amine proton signals were not enough to clearly determine the integral value by deconvolution analysis. **: The asymmetric reaction with (*R*)-*o*-anisyl methyl sulfoxide preferentially afforded opposite enantiomers to the other (*R*)-sulfoxides.

6.4 Synthesis of $\mathbf{1_{tight}}$ with (*R*)-methyl *p*-tolyl sulfoxide (2.2 equiv.)

A CH_2Cl_2 solution (0.2 mL) of (*R*)-methyl *p*-tolyl sulfoxide (3.49 mg, 22.6 µmol, 2.2 equiv. to the Pd salt, 7.1 equiv. to **L**) was added to a CH_2Cl_2 solution (0.4 mL) of [Pd(^{t}Bu$_2$bpy)(OH_2)$_2$](OTf)$_2$·(H_2O)$_2$ (7.67 mg, 10.3 µmol, 3.2 equiv. to **L**). To this solution was added a CH_2Cl_2 solution (0.4 mL) of **L** (2.00 mg, 3.17 µmol, 1.0 equiv.) at –70 °C. This reaction mixture was stirred at –70 °C for 4 h, brought to room temperature, and Et_2O was added to the reaction mixture and filtered. The filtrate was evaporated under reduced pressure to afford (*R*)-methyl *p*-tolyl sulfoxide. The precipitate was dissolved in CH_2Cl_2 and filtered to remove insoluble matters. The filtrate was evaporated and dried *in vacuo*. After the resulting solid was washed with $CHCl_3$, (*P*)-enantio-enriched $\mathbf{1_{tight}}$ was obtained as a pale yellow solid (1.85 mg, 0.70 µmol, 22% yield, 17% ee based on the deconvolution analysis using an iNMR software). The enantiomeric excess was estimated with Δ-**4** as a chiral shift reagent.

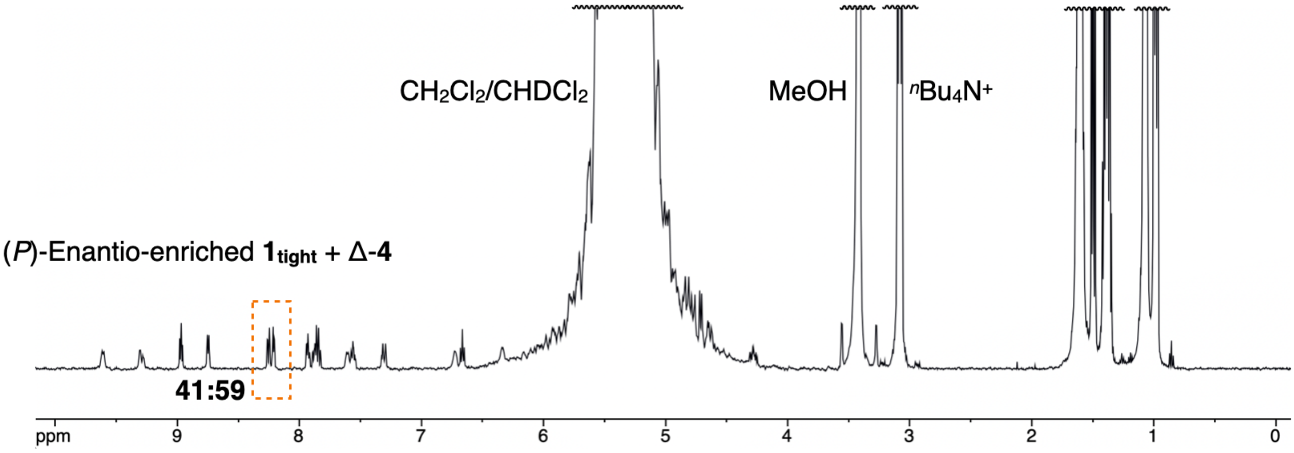

Supplementary Fig. 97 ^{1}H NMR spectrum of (*P*)-enantio-enriched $\mathbf{1_{tight}}$ synthesised with (*R*)-methyl *p*-tolyl sulfoxide (2.2 equiv.) at –70 °C and Δ-**4** as a chiral shift reagent (500 MHz, CD_2Cl_2/CH_2Cl_2, 300 K). (*P*)-enantio-enriched $\mathbf{1_{tight}}$ was dissolved in a mixture of CD_2Cl_2 and CH_2Cl_2.

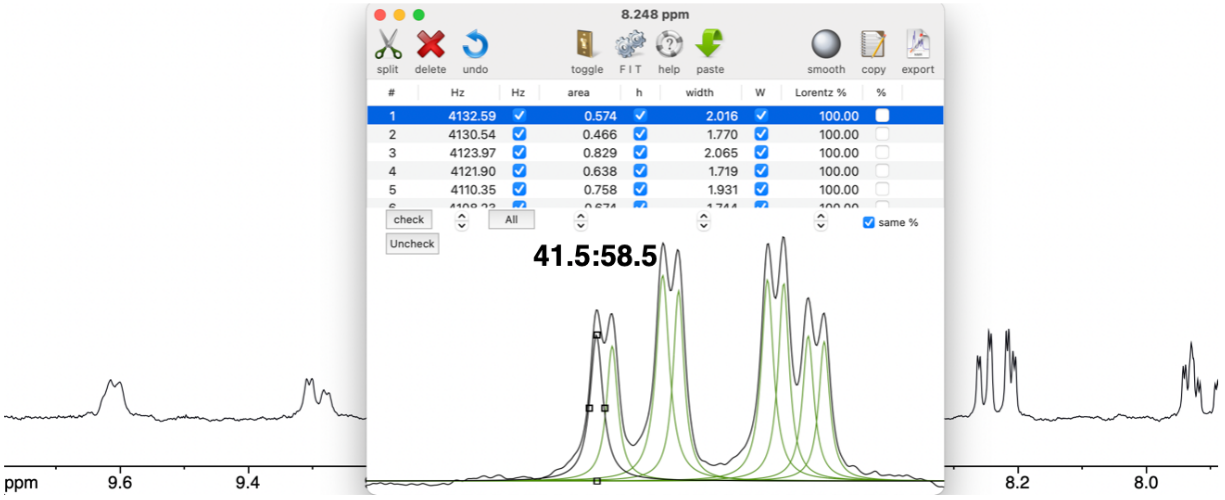

Supplementary Fig. 98 Deconvolution analysis of ^{1}H NMR spectrum of the (*P*)-enantio-enriched $\mathbf{1_{tight}}$ with Δ-**4**, shown in Supplementary Fig. 97.

6.5 Synthesis of $\mathbf{1_{tight}}$ with (*R*)-methyl *p*-tolyl sulfoxide (20 equiv.)

A CH_2Cl_2 solution (0.2 mL) of (*R*)-methyl *p*-tolyl sulfoxide (31.7 mg, 206 μmol, 20 equiv. to the Pd salt, 64 equiv. to **L**) was added to a CH_2Cl_2 solution (0.4 mL) of $[Pd(^tBu_2bpy)(OH_2)_2](OTf)_2{\cdot}(H_2O)_2$ (7.64 mg, 10.3 μmol, 3.2 equiv. to **L**). To this solution was added a CH_2Cl_2 solution (0.4 mL) of **L** (2.02 mg, 3.20 μmol, 1.0 equiv.) at –70 °C. This reaction solution was stirred at –70 °C for 4 h. After the reaction mixture was allowed to return to room temperature, the reaction mixture was filtered to remove insoluble matters. The filtrate was evaporated and dried *in vacuo*. After the resulting solid was washed with $CHCl_3$, a trace amount of (*P*)-enantio-enriched $\mathbf{1_{tight}}$ was obtained as a pale pink solid (64% ee based on the deconvolution analysis using an iNMR software). The enantiomeric excess was estimated with Δ-**4** as a chiral shift reagent.

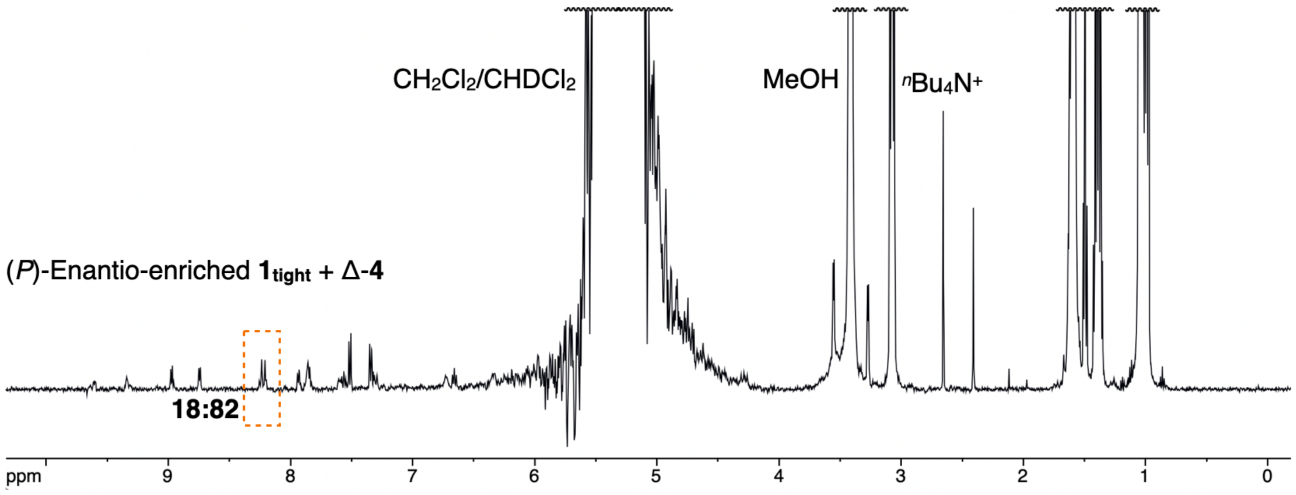

Supplementary Fig. 99 ^{1}H NMR spectrum of (*P*)-enantio-enriched $\mathbf{1_{tight}}$ synthesised with (*R*)-methyl *p*-tolyl sulfoxide (20 equiv. to the Pd salt) at –70 °C and Δ-**4** as a chiral shift reagent (500 MHz, CD_2Cl_2/CH_2Cl_2, 300 K). (*P*)-enantio-enriched $\mathbf{1_{tight}}$ were dissolved in a mixture of CD_2Cl_2 and CH_2Cl_2.

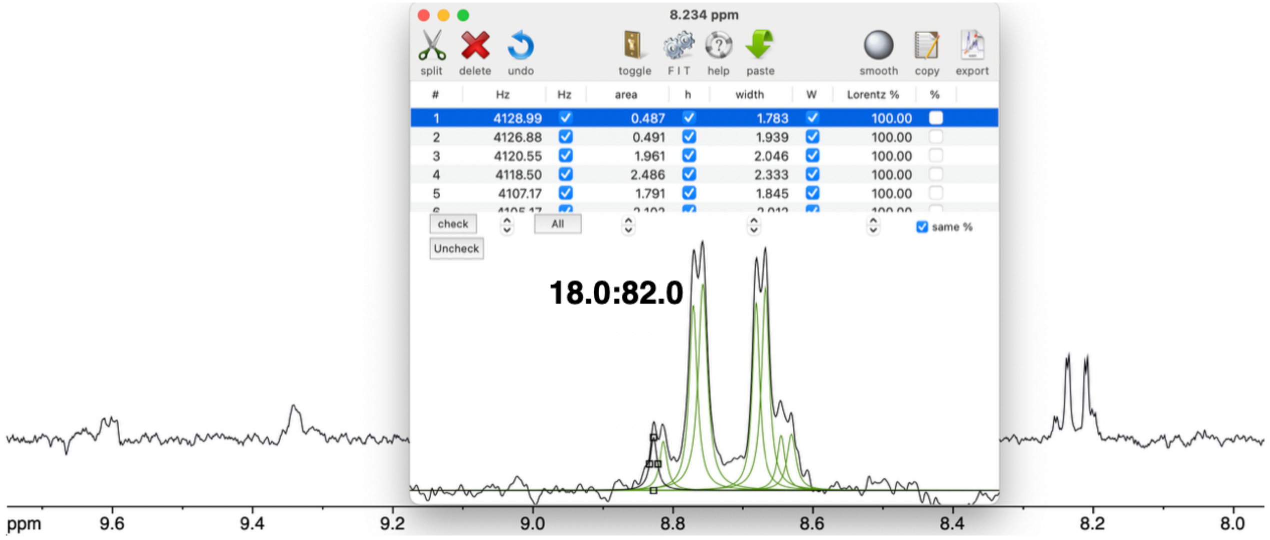

Supplementary Fig. 100 Deconvolution analysis of ^{1}H NMR spectrum of the (*P*)-enantio-enriched $\mathbf{1_{tight}}$ with Δ-**4**, shown in Supplementary Fig. 99.

6.6 Synthesis of $\mathbf{1_{tight}}$ with (*R*)-methyl phenyl sulfoxide

A CH_2Cl_2 solution (0.2 mL) of (*R*)-methyl phenyl sulfoxide (93% ee, 28.5 mg, 203 µmol, 20 equiv. to the Pd salt, 66 equiv. to **L**) was added to a CH_2Cl_2 solution (0.4 mL) of $[Pd(^tBu_2bpy)(OH_2)_2](OTf)_2{\cdot}(H_2O)_2$ (7.60 mg, 10.2 µmol, 3.3 equiv. to **L**). To this solution was added a CH_2Cl_2 solution (0.4 mL) of **L** (1.95 mg, 3.09 µmol, 1.0 equiv.) at –70 °C. This reaction solution was stirred at –70 °C for 4 h and was allowed to return to room temperature. The colour of the solution was changed from beige to purple, as the temperature approached to room temperature. To this solution was added Et_2O and the reaction mixture was filtered. The filtrate was evaporated under reduced pressure to afford (*R*)-methyl phenyl sulfoxide (25.3 mg, 180 µmol, 89% recovery yield). The precipitate was dissolved in CH_2Cl_2 and filtered to remove insoluble matters. The filtrate was evaporated and dried *in vacuo*. After the resulting solid was washed with $CHCl_3$, (*P*)-enantio-enriched $\mathbf{1_{tight}}$ was obtained as a pale pink solid (0.53 mg, 0.20 µmol, 6.5% yield, 17% ee based on the deconvolution analysis using an iNMR software). The enantiomeric excess was estimated with Δ-**4** as a chiral shift reagent.

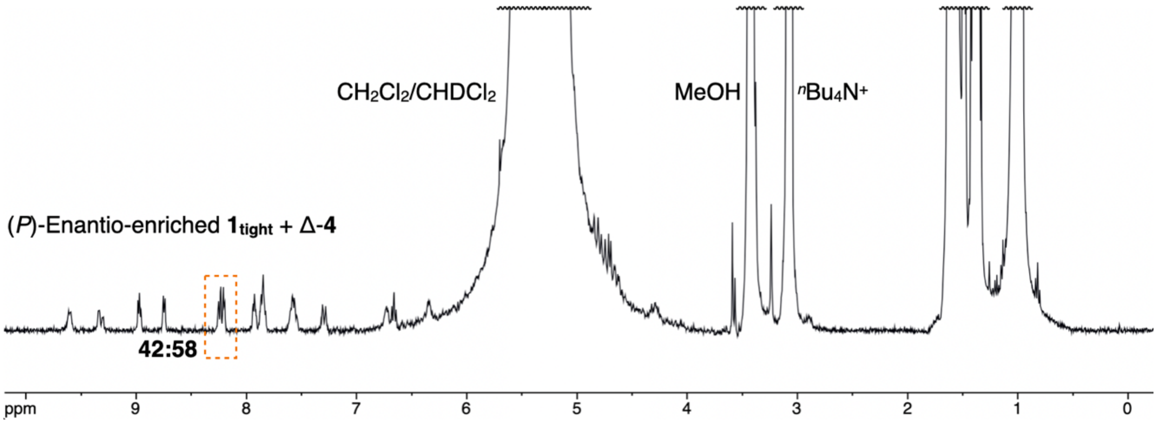

Supplementary Fig. 101 ^{1}H NMR spectrum of (*P*)-enantio-enriched $\mathbf{1_{tight}}$ synthesised with (*R*)-methyl phenyl sulfoxide (20 equiv. to the Pd salt) at –70 °C and Δ-**4** as a chiral shift reagent (400 MHz, CD_2Cl_2/CH_2Cl_2, 298 K). (*P*)-Enantio-enriched $\mathbf{1_{tight}}$ was dissolved in a mixture of CD_2Cl_2 and CH_2Cl_2.

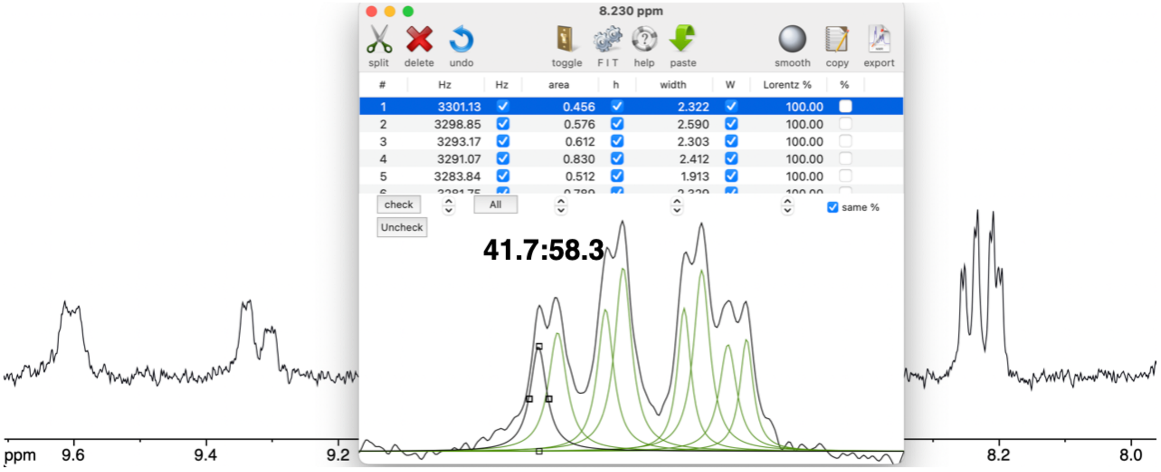

Supplementary Fig. 102 Deconvolution analysis of ^{1}H NMR spectrum of the (*P*)-enantio-enriched $\mathbf{1_{tight}}$ with Δ-**4**, shown in Supplementary Fig. 101.

6.7 Synthesis of 1_{tight} with (*R*)-*p*-anisyl methyl sulfoxide

A CH_2Cl_2 solution (0.2 mL) of (*R*)-*p*-anisyl methyl sulfoxide (97% ee, 34.8 mg, 204 μmol, 20 equiv. to the Pd salt, 65 equiv. to **L**) was added to a CH_2Cl_2 solution (0.4 mL) of $[Pd(^tBu_2bpy)(OH_2)_2](OTf)_2{\cdot}(H_2O)_2$ (7.67 mg, 10.3 μmol, 3.3 equiv.). To this solution was added a CH_2Cl_2 solution (0.4 mL) of **L** (1.99 mg, 3.15 μmol, 1.0 equiv.) at –70 °C. This reaction mixture was stirred at –70 °C for 24 h. The colour of the solution was changed from yellow to pink orange during the reaction. After the reaction mixture was allowed to return to room temperature, Et_2O was added and the reaction mixture was filtered. The filtrate was evaporated under reduced pressure to afford (*R*)-*p*-anisyl methyl sulfoxide (32.6 mg, 192 μmol, 94% recovery yield). The precipitate was dissolved in CH_2Cl_2 and filtered to remove insoluble matters. The filtrate was evaporated and dried *in vacuo*. After the resulting solid was washed with $CHCl_3$, (*P*)-enantio-enriched $\mathbf{1}_{tight}$ was obtained as a pale pink orange solid (0.31 mg, 0.12 μmol, 3.7% yield, 17% ee based on the deconvolution analysis using an iNMR software). The enantiomeric excess was estimated with Δ-**4** as a chiral shift reagent.

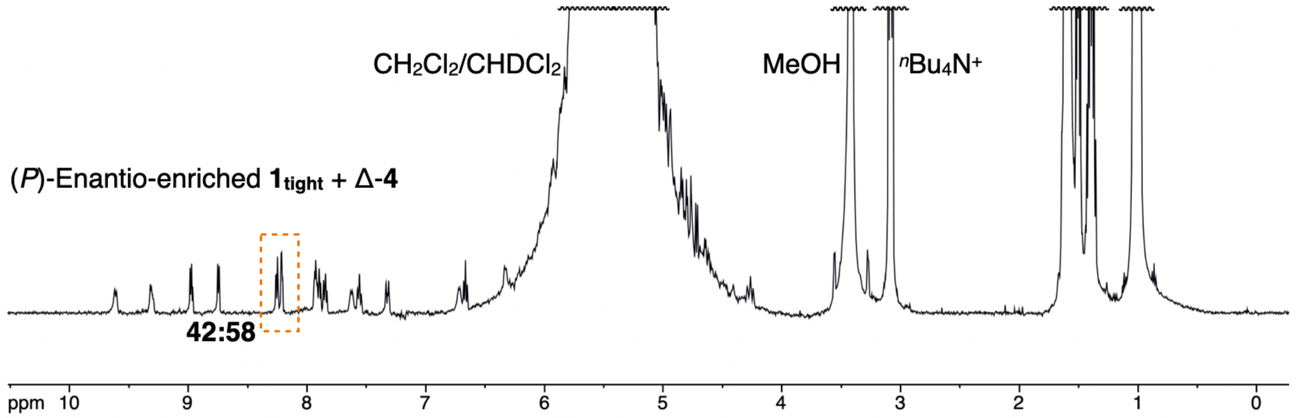

Supplementary Fig. 103 ^{1}H NMR spectrum of (*P*)-enantio-enriched $\mathbf{1}_{tight}$ synthesised with (*R*)-*p*-anisyl methyl sulfoxide (20 equiv. to the Pd salt) at –70 °C and Δ-**4** as a chiral shift reagent (500 MHz, CD_2Cl_2/CH_2Cl_2, 300 K). (*P*)-Enantio-enriched $\mathbf{1}_{tight}$ was dissolved in a mixture of CD_2Cl_2 and CH_2Cl_2.

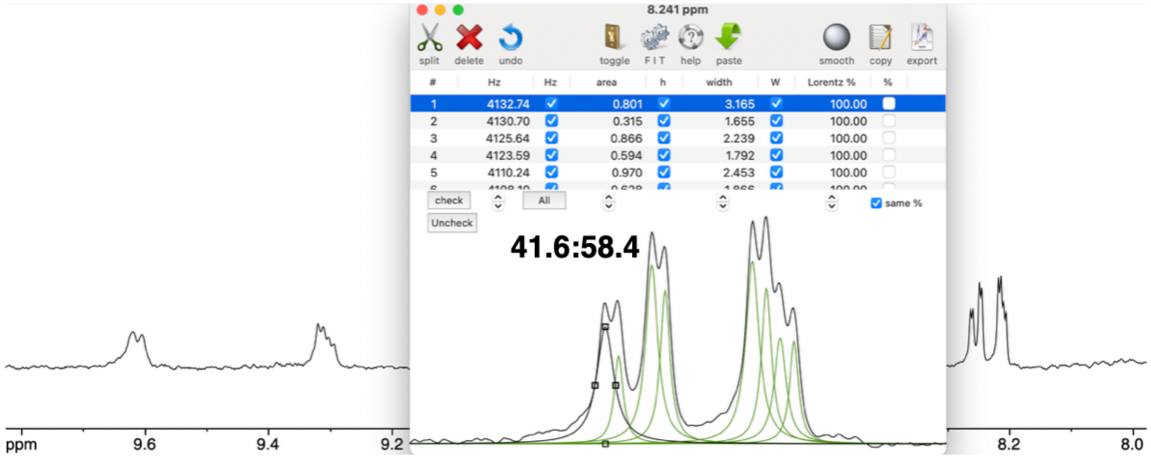

Supplementary Fig. 104 Deconvolution analysis of ^{1}H NMR spectrum of the (*P*)-enantio-enriched $\mathbf{1}_{tight}$ with Δ-**4**, shown in Supplementary Fig. 103.

6.8 Synthesis of $\mathbf{1_{tight}}$ with (*R*)-*o*-anisyl methyl sulfoxide

A CH_2Cl_2 solution (0.2 mL) of (*R*)-*o*-anisyl methyl sulfoxide (> 99% ee, 34.4 mg, 202 µmol, 20 equiv. to the Pd salt, 65 equiv. to **L**) was added to a CH_2Cl_2 solution (0.4 mL) of $[Pd(^tBu_2bpy)(OH_2)_2](OTf)_2{\cdot}(H_2O)_2$ (7.61 mg, 10.2 µmol, 3.3 equiv.). To this solution was added a CH_2Cl_2 solution (0.4 mL) of **L** (1.96 mg, 3.11 µmol, 1.0 equiv.) at –70 °C. This reaction mixture was stirred at –70 °C for 4 h. After the reaction mixture was allowed to come to room temperature, Et_2O was added and the reaction mixture was filtered. The filtrate was evaporated under reduced pressure to afford (*R*)-*o*-anisyl methyl sulfoxide. The precipitate was dissolved in CH_2Cl_2 and filtered to remove insoluble matters. The filtrate was evaporated and dried *in vacuo*. After the resulting solid was washed with $CHCl_3$, (*M*)-enantio-enriched $\mathbf{1_{tight}}$ was obtained as a pale yellow solid (1.00 mg, 0.377 µmol, 12% yield, 21% ee based on the deconvolution analysis using an iNMR software). The enantiomeric excess was estimated with Δ-**4** as a chiral shift reagent.

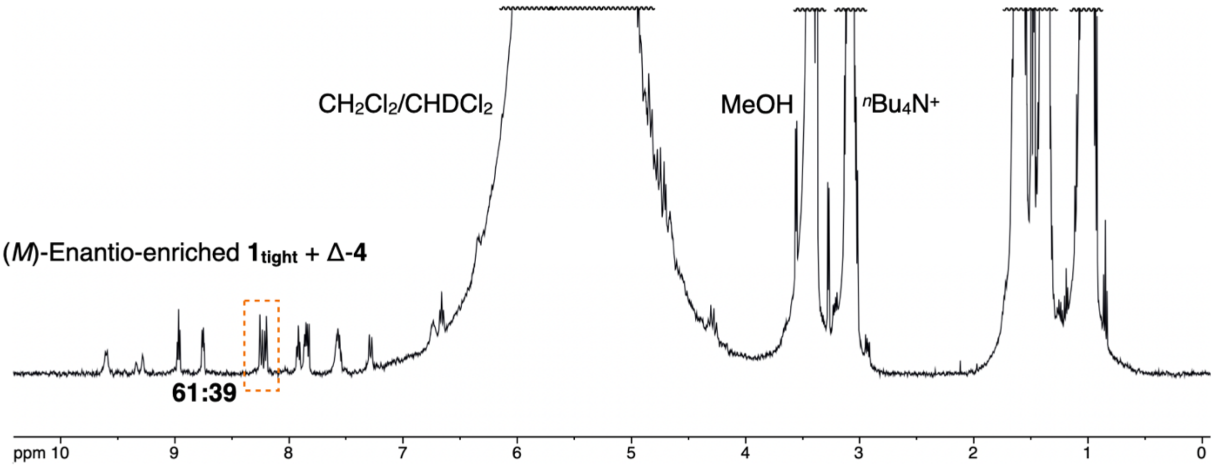

Supplementary Fig. 105 ^{1}H NMR spectrum of (*M*)-enantio-enriched $\mathbf{1_{tight}}$ synthesised with (*R*)-*o*-anisyl methyl sulfoxide (20 equiv. to the Pd salt) at –70 °C and Δ-**4** as a chiral shift reagent (500 MHz, CD_2Cl_2/CH_2Cl_2, 300 K). (*M*)-Enantio-enriched $\mathbf{1_{tight}}$ was dissolved in a mixture of CD_2Cl_2 and CH_2Cl_2.

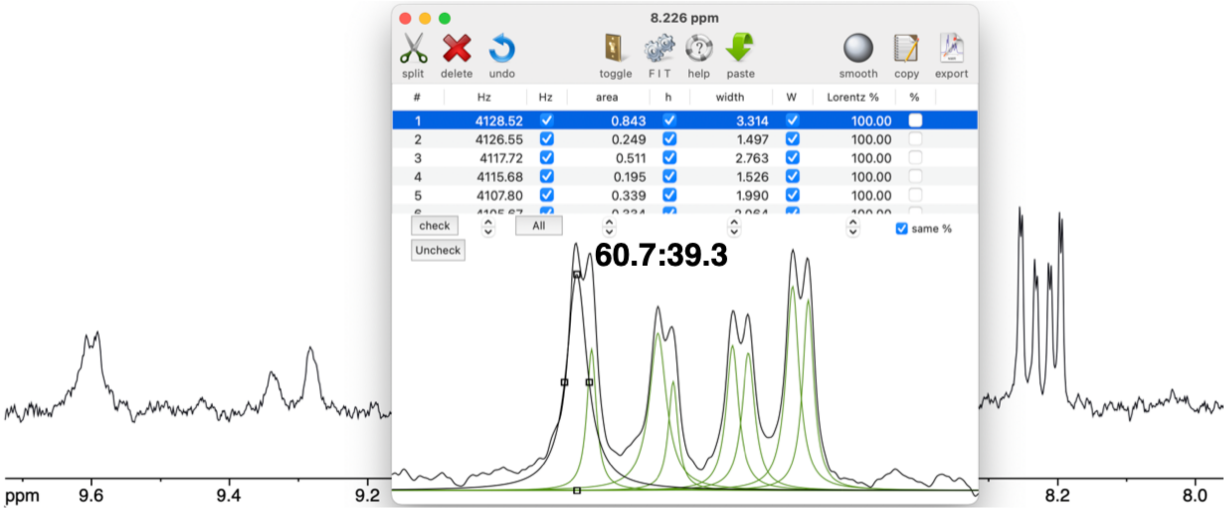

Supplementary Fig. 106 Deconvolution analysis of ^{1}H NMR spectrum of the (*M*)-enantio-enriched $\mathbf{1_{tight}}$ with Δ-**4**, shown in Supplementary Fig. 105.

7. Evaluation of the helicity inversion rate of $\mathbf{1}_{\mathbf{tight}}$

7.1 Theoretical analysis of the rate constant of the helicity inversion of $\mathbf{1}_{\mathbf{tight}}$

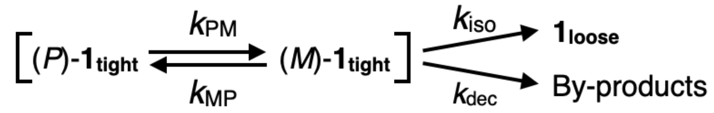

Supplementary Fig. 107 Scheme of the helicity inversion of $\mathbf{1}_{\mathbf{tight}}$, isomerisation from $\mathbf{1}_{\mathbf{tight}}$ to $\mathbf{1}_{\mathbf{loose}}$ and decomposition of $\mathbf{1}_{\mathbf{tight}}$ with the rate constant of each reaction.

We considered the inversion and isomerisation reaction as pseudo first order reactions, as shown in the scheme above. In this case, the differential equations are given below:

$$\frac{d}{dt}\left[(P)\text{-}\mathbf{1}_{\mathbf{tight}}\right] = -k_{PM}\left[(P)\text{-}\mathbf{1}_{\mathbf{tight}}\right] + k_{MP}\left[(M)\text{-}\mathbf{1}_{\mathbf{tight}}\right] - (k_{iso} + k_{dec})\left[(P)\text{-}\mathbf{1}_{\mathbf{tight}}\right] \quad \cdots(7\text{-}1)$$

$$\frac{d}{dt}\left[(M)\text{-}\mathbf{1}_{\mathbf{tight}}\right] = -k_{MP}\left[(M)\text{-}\mathbf{1}_{\mathbf{tight}}\right] + k_{PM}\left[(P)\text{-}\mathbf{1}_{\mathbf{tight}}\right] - (k_{iso} + k_{dec})\left[(M)\text{-}\mathbf{1}_{\mathbf{tight}}\right] \quad \cdots(7\text{-}2)$$

where $[(P)\text{-}\mathbf{1}_{\mathbf{tight}}]$ and $[(M)\text{-}\mathbf{1}_{\mathbf{tight}}]$ are the concentration of (*P*)- and (*M*)-$\mathbf{1}_{\mathbf{tight}}$, respectively, k_{PM} and k_{MP} are the rate constants of helicity inversion from (*P*)- to (*M*)- and from (*M*)- to (*P*)-$\mathbf{1}_{\mathbf{tight}}$, respectively, k_{iso} is the rate constant of isomerisation from $\mathbf{1}_{\mathbf{tight}}$ to $\mathbf{1}_{\mathbf{loose}}$ and k_{dec} is the rate constant of decomposition of $\mathbf{1}_{\mathbf{tight}}$. We supposed that k_{iso} and k_{dec} of (*P*)-$\mathbf{1}_{\mathbf{tight}}$ were the same as those of (*M*)-$\mathbf{1}_{\mathbf{tight}}$. By subtracting equation (7-1) from (7-2) and solving the differential equation, we get the difference between $[(M)\text{-}\mathbf{1}_{\mathbf{tight}}]$ and $[(P)\text{-}\mathbf{1}_{\mathbf{tight}}]$, which corresponds to enantiomeric excess, as below:

$$\frac{d}{dt}\left(\left[(M)\text{-}\mathbf{1}_{\mathbf{tight}}\right] - \left[(P)\text{-}\mathbf{1}_{\mathbf{tight}}\right]\right) = -(k + k_{iso} + k_{dec})\left(\left[(M)\text{-}\mathbf{1}_{\mathbf{tight}}\right] - \left[(P)\text{-}\mathbf{1}_{\mathbf{tight}}\right]\right)$$

$$\left(\left[(M)\text{-}\mathbf{1}_{\mathbf{tight}}\right] - \left[(P)\text{-}\mathbf{1}_{\mathbf{tight}}\right]\right) = Const.\cdot e^{-(k+k_{iso}+k_{dec})t} \quad \cdots(7\text{-}3)$$

where $2k_{PM} = 2k_{MP} = k$ and *Const.* is a constant of integration. In the time course experiments, (*M*)-enantio-enriched $\mathbf{1}_{\mathbf{tight}}$ with E_0% ee was used, indicating that

$$\left(\left[(M)\text{-}\mathbf{1}_{\mathbf{tight}}\right]_0 - \left[(P)\text{-}\mathbf{1}_{\mathbf{tight}}\right]_0\right) = \left(\left[(M)\text{-}\mathbf{1}_{\mathbf{tight}}\right]_0 + \left[(P)\text{-}\mathbf{1}_{\mathbf{tight}}\right]_0\right)\cdot\frac{E_0}{100} \quad \cdots(7\text{-}4)$$

where $[(P)\text{-}\mathbf{1}_{\mathbf{tight}}]_0$ and $[(M)\text{-}\mathbf{1}_{\mathbf{tight}}]_0$ are the initial concentrations of (*P*)- and (*M*)-$\mathbf{1}_{\mathbf{tight}}$. By combining equations (7-3) and (7-4) where $t = 0$, *Const.* is determined. Therefore, the equation (7-5) is given below:

$$\left[(M)\text{-}\mathbf{1}_{\mathbf{tight}}\right] - \left[(P)\text{-}\mathbf{1}_{\mathbf{tight}}\right] = \left(\left[(M)\text{-}\mathbf{1}_{\mathbf{tight}}\right]_0 + \left[(P)\text{-}\mathbf{1}_{\mathbf{tight}}\right]_0\right)\cdot\frac{E_0}{100}\cdot e^{-(k+k_{iso}+k_{dec})t} \quad \cdots(7\text{-}5)$$

On the other hand, the decrease in the concentration of $\mathbf{1}_{\mathbf{tight}}$ is given by the sum of equations (7-1) and (7-2) and solving the differential equation:

$$\left[(M)\text{-}\mathbf{1}_{\mathbf{tight}}\right] + \left[(P)\text{-}\mathbf{1}_{\mathbf{tight}}\right] = \left(\left[(M)\text{-}\mathbf{1}_{\mathbf{tight}}\right]_0 + \left[(P)\text{-}\mathbf{1}_{\mathbf{tight}}\right]_0\right)\cdot e^{-(k_{iso}+k_{dec})t} \quad \cdots(7\text{-}6)$$

If the enantiomeric excess was decreased to E_1% ee T hours after the (M)-enantio-enriched $\mathbf{1}_{\mathbf{tight}}$ was dissolved in acetone-d_6, the difference between [(M)-$\mathbf{1}_{\mathbf{tight}}$] and [($P$)-$\mathbf{1}_{\mathbf{tight}}$] is given below:

$$\left(\left[(M)\text{-}\mathbf{1}_{\mathbf{tight}}\right] - \left[(P)\text{-}\mathbf{1}_{\mathbf{tight}}\right]\right) = \left(\left[(M)\text{-}\mathbf{1}_{\mathbf{tight}}\right] + \left[(P)\text{-}\mathbf{1}_{\mathbf{tight}}\right]\right) \cdot \frac{E_1}{100} \quad \cdots(7\text{-}7)$$

Then, substitution of equations (7-5) and (7-6) for (7-7) and simplification gives:

$$\left(\left[(M)\text{-}\mathbf{1}_{\mathbf{tight}}\right]_0 + \left[(P)\text{-}\mathbf{1}_{\mathbf{tight}}\right]_0\right) \cdot \frac{E_0}{100} \cdot e^{-(k+k_{iso}+k_{dec})3600T}$$

$$= \left(\left[(M)\text{-}\mathbf{1}_{\mathbf{tight}}\right]_0 + \left[(P)\text{-}\mathbf{1}_{\mathbf{tight}}\right]_0\right) \cdot \frac{E_1}{100} \cdot e^{-(k_{iso}+k_{dec})3600T}$$

$$E_0 \cdot e^{-3600kT} = E_1$$

$$k = \frac{1}{3600T} \ln\left(\frac{E_0}{E_1}\right) \quad \cdots(7\text{-}8)$$

The equation (7-8) indicates that isomerisation from $\mathbf{1}_{\mathbf{tight}}$ to $\mathbf{1}_{\mathbf{loose}}$ and decomposition of $\mathbf{1}_{\mathbf{tight}}$ can be ignored in estimation of the rate constant of the helicity inversion between (P)- and (M)-$\mathbf{1}_{\mathbf{tight}}$.

7.2 Time course ^{1}H NMR analysis to evaluate the racemisation rate of (*M*)-enantio-enriched $\mathbf{1}_{\mathbf{tight}}$ in acetone-d_6

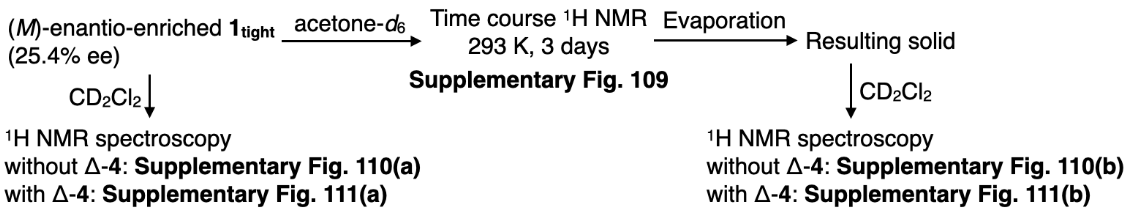

Supplementary Fig. 108 Scheme of time course analysis of (*M*)-enantio-enriched $\mathbf{1}_{\mathbf{tight}}$.

(*M*)-Enantio-enriched $\mathbf{1}_{\mathbf{tight}}$ (0.28 mg, 0.11 µmol, 25.4% ee) was dissolved in acetone-d_6 (500 µL, 0.21 mM) and this solution was allowed to stand at 293 K for 3 days as monitored by ^{1}H NMR spectroscopy. This solution was then evaporated under reduced pressure at room temperature. The resulting solid was dissolved in CD_2Cl_2 and the enantiomeric excess of $\mathbf{1}_{\mathbf{tight}}$ was estimated by ^{1}H NMR spectroscopy in the presence of Δ-**4** as a chiral shift reagent.

Although the change in the enantiomeric excess of $\mathbf{1}_{\mathbf{tight}}$ was not observed in the ^{1}H NMR spectra, the inversion rate was evaluated by the equation (7-8) to be less than 8.5×10^{-7} s^{-1} in acetone-d_6 at 293 K from the decrease in the enantiomeric excess by 5% ee (from 25.4% to 20.4%) after 72 h. This evaluated rate of inversion between (*P*)- and (*M*)-$\mathbf{1}_{\mathbf{tight}}$ was slower than the rate of isomerisation from $\mathbf{1}_{\mathbf{tight}}$ to $\mathbf{1}_{\mathbf{loose}}$ (5.7×10^{-6} s^{-1}), which is consistent with the discussion regarding the absolute configuration of the amine nitrogen atoms of **L**.

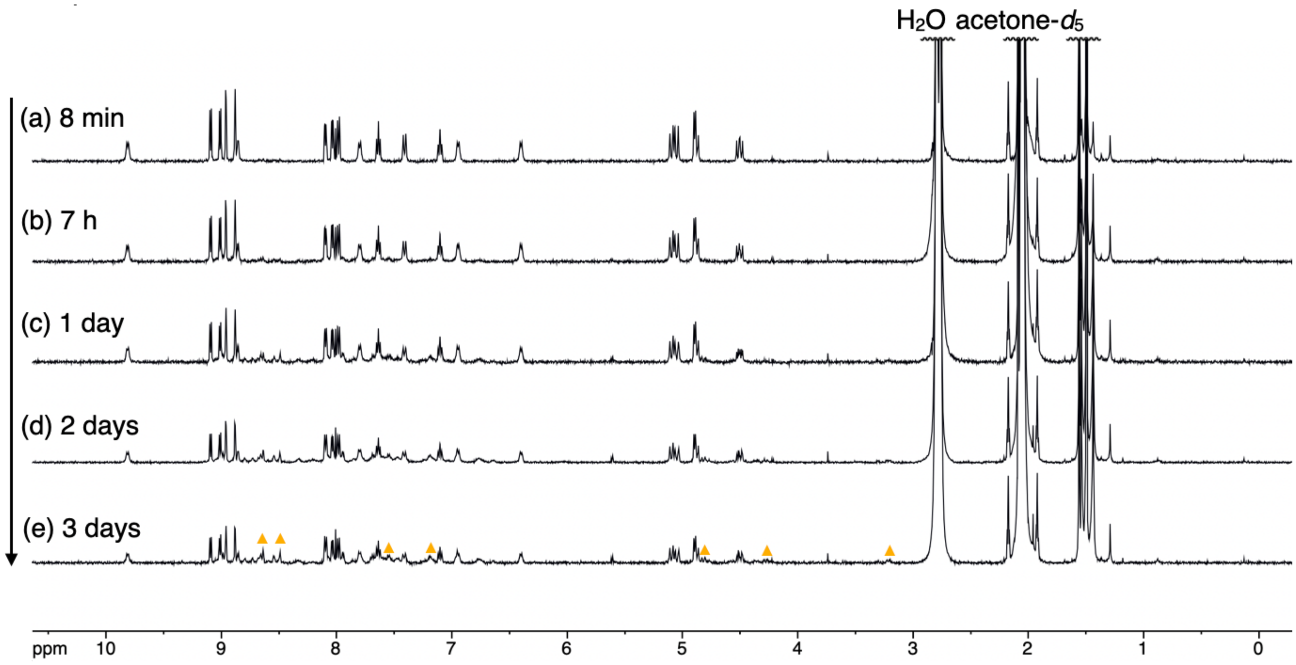

Supplementary Fig. 109 Time course ^{1}H NMR spectra of (*M*)-enantio-enriched $\mathbf{1}_{\mathbf{tight}}$ (a) 8 min, (b) 7 h, (c) 1 day, (d) 2 days and (e) 3 days at 293 K after dissolved in acetone-d_6 (500 MHz, acetone-d_6,

300 K). Orange triangles indicate that $\mathbf{1}_{\mathbf{loose}}$ gradually formed. The rate of isomerisation and decomposition appears to be slightly different from the rate estimated in Supplementary Fig. 48 probably due to different conditions such as purity of the products. Specifically, *rac*-$\mathbf{1}_{\mathbf{tight}}$ was purified by recrystallisation, while the (*M*)-enantio-enriched $\mathbf{1}_{\mathbf{tight}}$ was not recrystallised to prevent changes in enantiomeric excess, as described in the text.

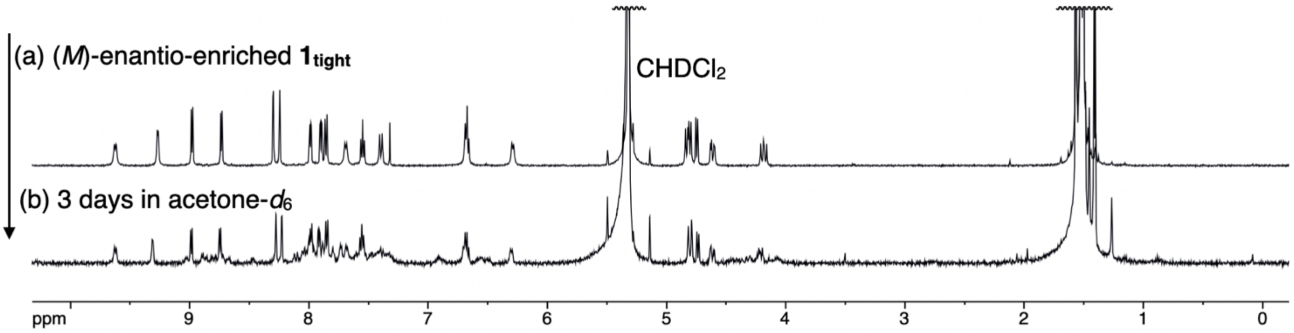

Supplementary Fig. 110 ^{1}H NMR spectra of (a) (*M*)-enantio-enriched $\mathbf{1}_{\mathbf{tight}}$ in CD_2Cl_2 and (b) (*M*)-enantio-enriched $\mathbf{1}_{\mathbf{tight}}$ 3 days after dissolved in acetone-d_6, evaporated and then redissolved in CD_2Cl_2 (500 MHz, CD_2Cl_2, 300 K).

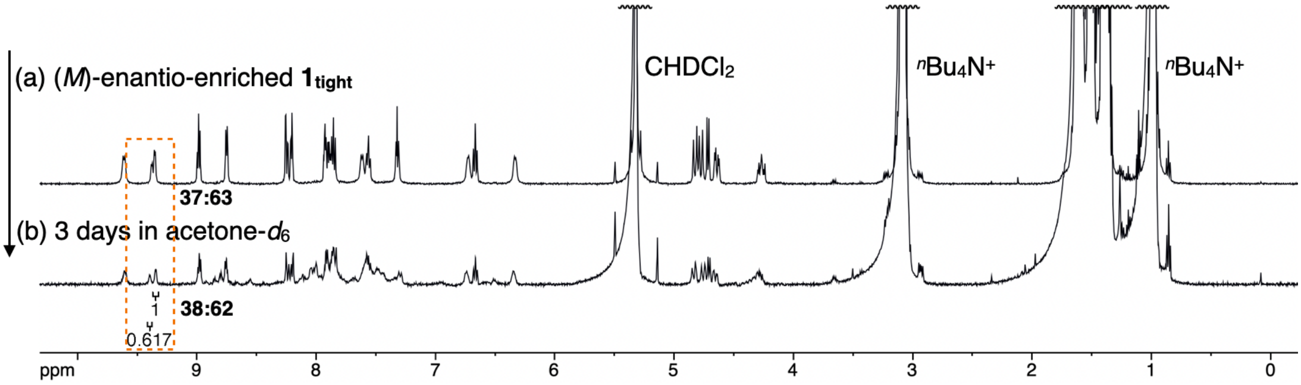

Supplementary Fig. 111 (a),(b) ^{1}H NMR spectra of the NMR samples shown in Supplementary Fig. 110, respectively, after addition of an excess amount of Δ-**4** as a chiral shift reagent (500 MHz, CD_2Cl_2, 300 K).

7.3 Time course ^{1}H NMR analysis to evaluate the racemisation rate of (*M*)-enantio-enriched $\mathbf{1}_{\mathbf{tight}}$ in CD_2Cl_2

(*M*)-Enantio-enriched $\mathbf{1}_{\mathbf{tight}}$ (0.26 mg, 0.098 μmol, 25.4% ee) was dissolved in CD_2Cl_2 (500 μL, 0.20 mM) and this solution was allowed to stand at 293 K for 10 days as monitored by ^{1}H NMR spectroscopy. The enantiomeric ratio of $\mathbf{1}_{\mathbf{tight}}$ was then estimated by ^{1}H NMR spectroscopy in the presence of Δ-**4** as a chiral shift reagent.

Although the change in the enantiomeric excess of $\mathbf{1}_{\mathbf{tight}}$ was not observed in the ^{1}H NMR spectra, the inversion rate was evaluated by the equation (7-8) to be less than 9.61×10^{-8} s^{-1} in CD_2Cl_2 at 293 K from the decrease in the enantiomeric excess by 5% ee (from 25.4% ee to 20.4% ee) after 10 days.

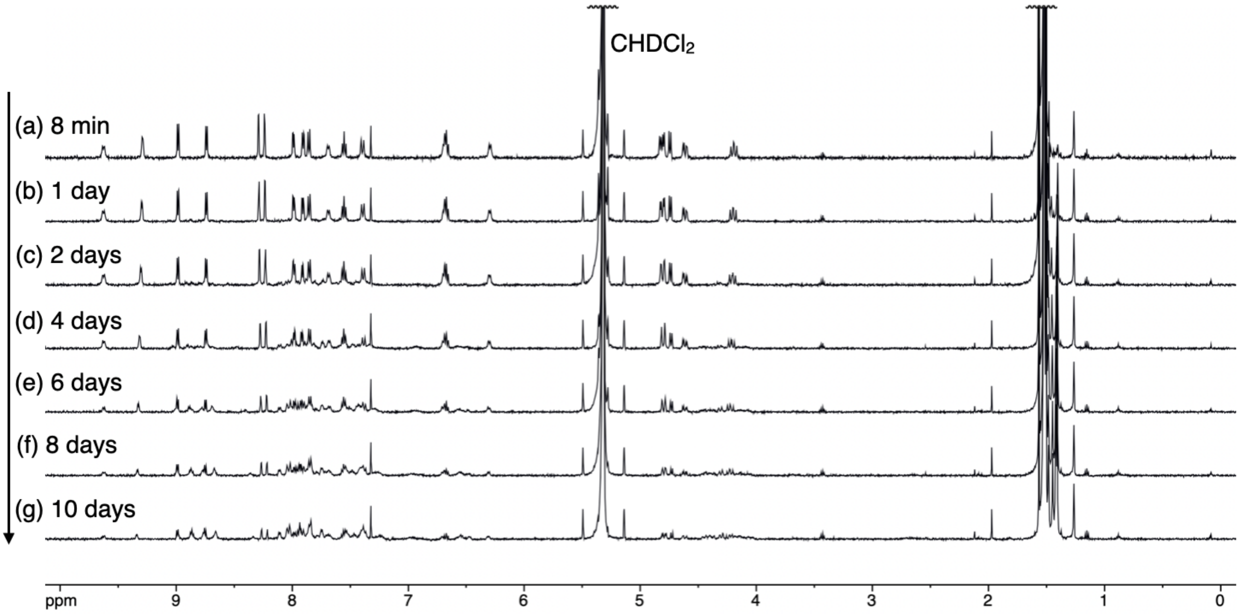

Supplementary Fig. 112 Time course ^{1}H NMR spectra of (*M*)-enantio-enriched $\mathbf{1}_{\mathbf{tight}}$ (a) 8 min (b) 1 day, (c) 2 days, (d) 4 days, (e) 6 days, (f) 8 days and (g) 10 days at 293 K after dissolved in CD_2Cl_2 (500 MHz, CD_2Cl_2, 300 K).

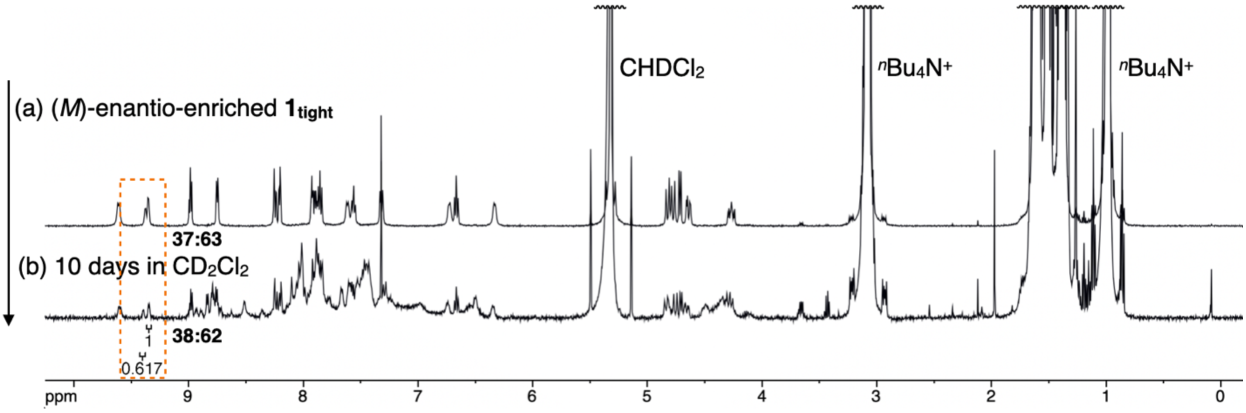

Supplementary Fig. 113 ^{1}H NMR spectra of (a) (*M*)-enantio-enriched $\mathbf{1}_{\mathbf{tight}}$ 30 min after dissolved in CD_2Cl_2 with an excess amount of Δ-**4** as a chiral shift reagent and (b) (*M*)-enantio-enriched $\mathbf{1}_{\mathbf{tight}}$ 10 days at 293 K after dissolved in CD_2Cl_2. The spectra were measured just after addition of Δ-**4** (500 MHz, CD_2Cl_2, 300 K).

7.4 Time course ^{1}H NMR analysis of *rac*-$\mathbf{1}_{tight}$ in CD_2Cl_2 with Δ-4

To a CD_2Cl_2 solution (450 μL) of *rac*-$\mathbf{1}_{tight}$ (0.50 mg, 0.19 μmol, 0.42 mM) was added Δ-**4** (49 equiv. calculated by the integral ratio). This solution was allowed to stand at room temperature for 14 days as monitored by ^{1}H NMR spectroscopy. The diastereomeric ratio was estimated by the integral ratio in the ^{1}H NMR spectra. Though the ratio between enantiomers of $\mathbf{1}_{tight}$ was slightly changed after 14 days in the presence of 49 equiv. of Δ-**4**, the ratio remained around 50 to 50 in 7 days, indicating that Δ-**4** did not affect the enantiomeric excess of $\mathbf{1}_{tight}$ in several days and can work as a chiral shift reagent.

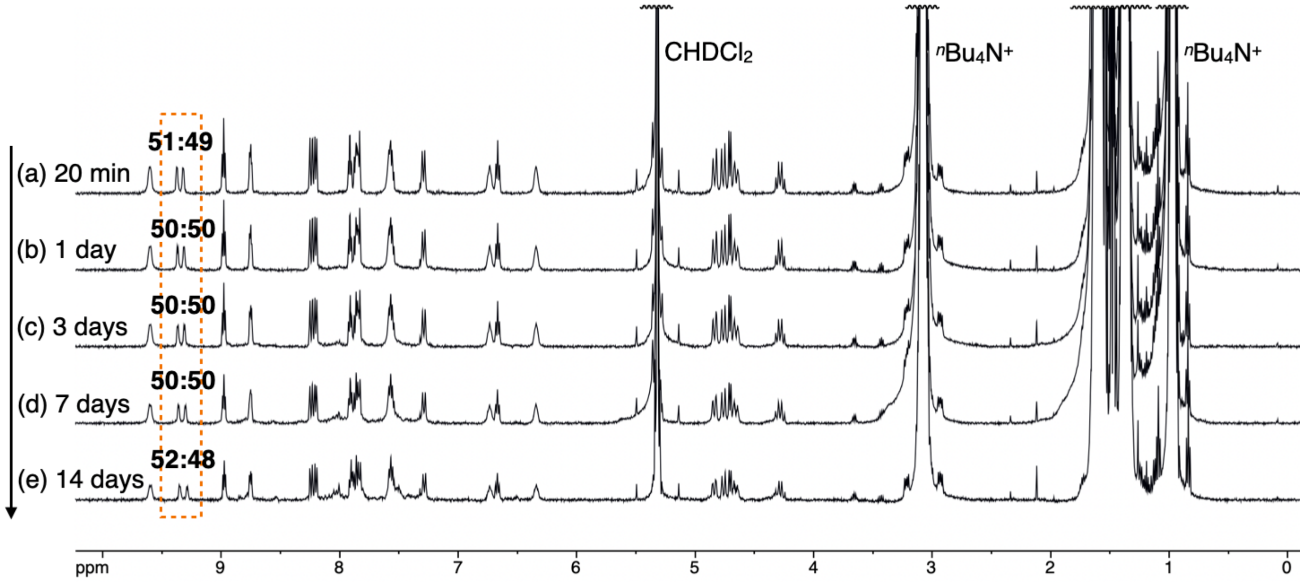

Supplementary Fig. 114 ^{1}H NMR spectra of *rac*-$\mathbf{1}_{tight}$ with Δ-**4** (49 equiv.) (500 MHz, CD_2Cl_2, 300 K); (a) 20 min after addition of Δ-**4**, (b) 1 day, (c) 3 days, (d) 7 days and (e) 14 days. The reaction solution was allowed to stand at room temperature.

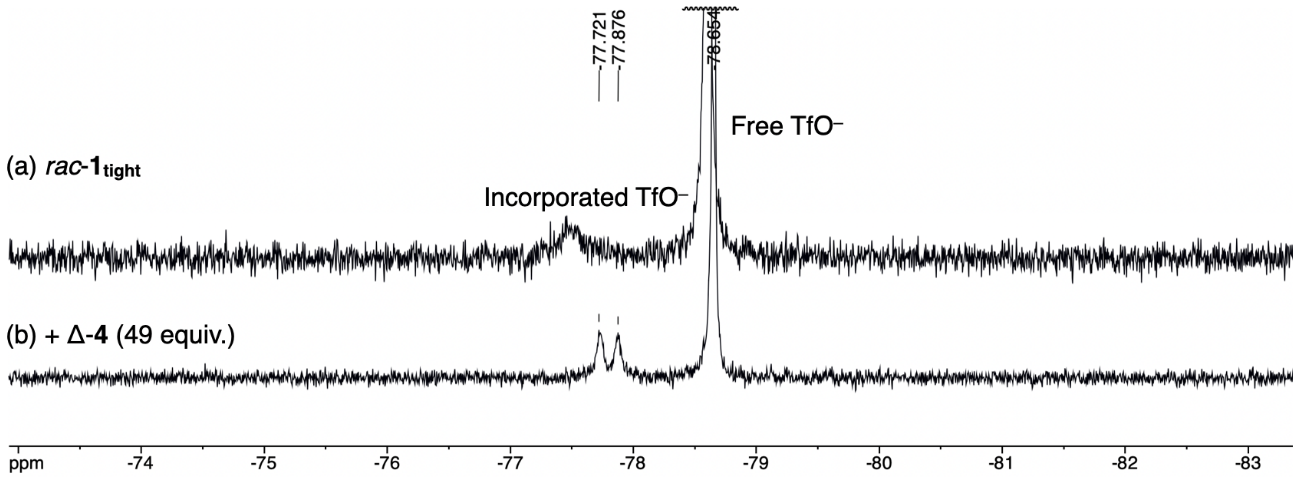

Supplementary Fig. 115 ^{19}F NMR spectra of (a) *rac*-$\mathbf{1}_{tight}$, (b) *rac*-$\mathbf{1}_{tight}$ with Δ-**4** (49 equiv.) (471 MHz, CD_2Cl_2, 300 K).

7.5 Time course ^{1}H NMR analysis of (*M*)-enantio-enriched $\mathbf{1}_{\mathbf{tight}}$ in CD_2Cl_2 with Δ-4

To a CD_2Cl_2 solution (400 µL) of (*M*)-enantio-enriched $\mathbf{1}_{\mathbf{tight}}$ (0.59 mg, 0.22 µmol, 0.56 mM, 25% ee) was added Δ-**4** (17 equiv. calculated by the integral ratio). This solution was allowed to stand at 293 K for 14 days as monitored by ^{1}H NMR spectroscopy. The diastereomeric ratio was estimated based on the deconvolution analysis using an iNMR software.

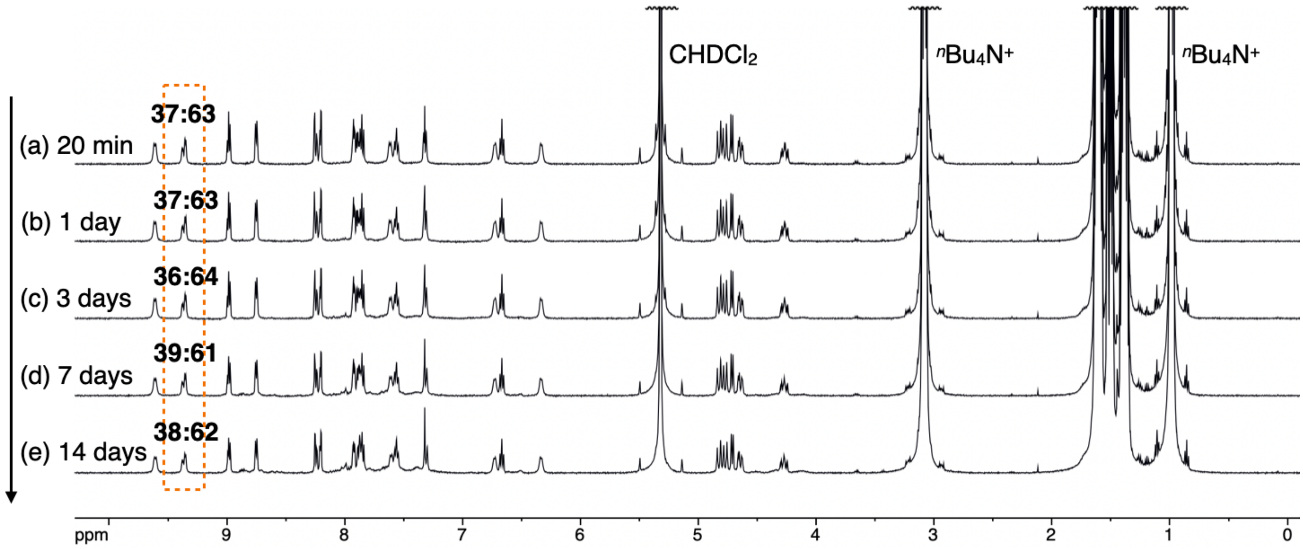

Supplementary Fig. 116 ^{1}H NMR spectra of (*M*)-enantio-enriched $\mathbf{1}_{\mathbf{tight}}$ with Δ-**4** (17 equiv.) (500 MHz, CD_2Cl_2, 300 K); (a) 20 min after addition of Δ-**4**, (b) 1 day, (c) 3 days, (d) 7 days and (e) 14 days. The reaction solution was allowed to stand at room temperature.

(a)

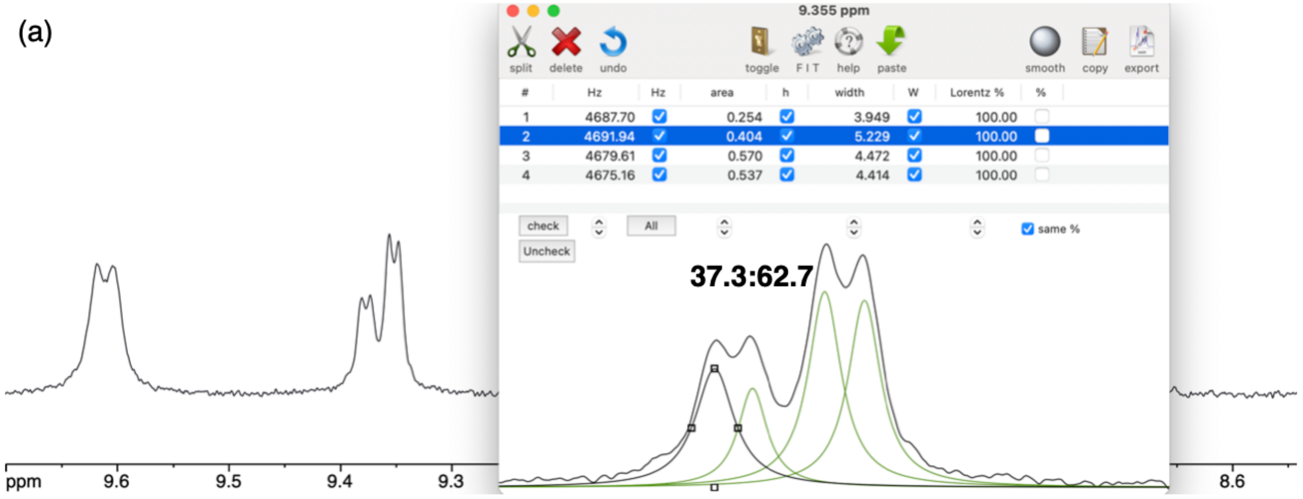

(b)

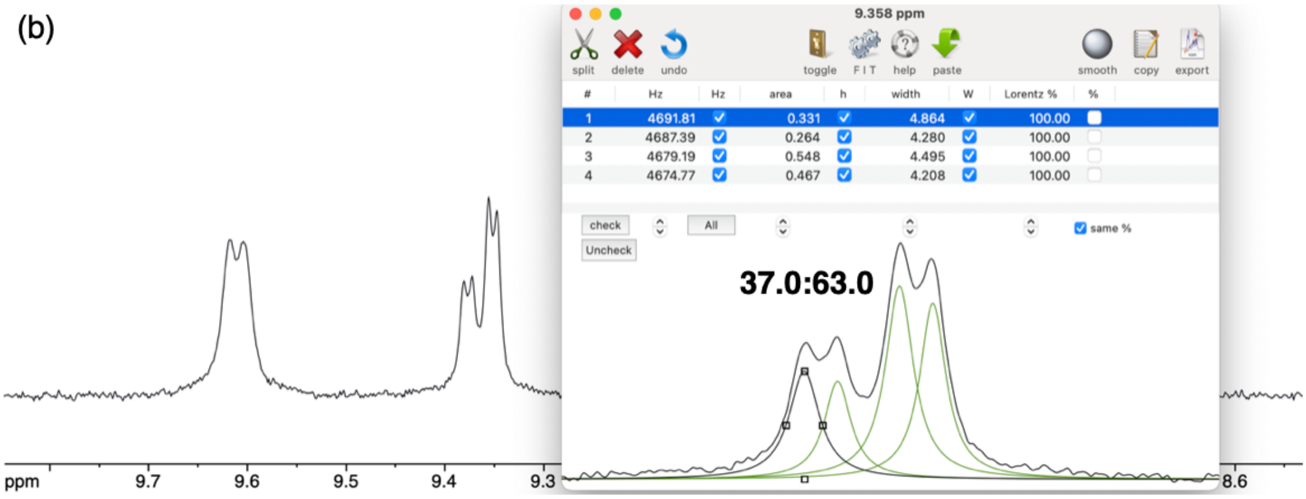
9.358 ppm
split delete undo toggle FIT help paste smooth copy export
Hz Hz area h width W Lorentz % %
1 4691.81 0.331 4.864 100.00
2 4687.39 0.264 4.280 100.00
3 4679.19 0.548 4.495 100.00
4 4674.77 0.467 4.208 100.00
check All same %
Uncheck
37.0:63.0
ppm 9.7 9.6 9.5 9.4 9.3 8.6

(c)

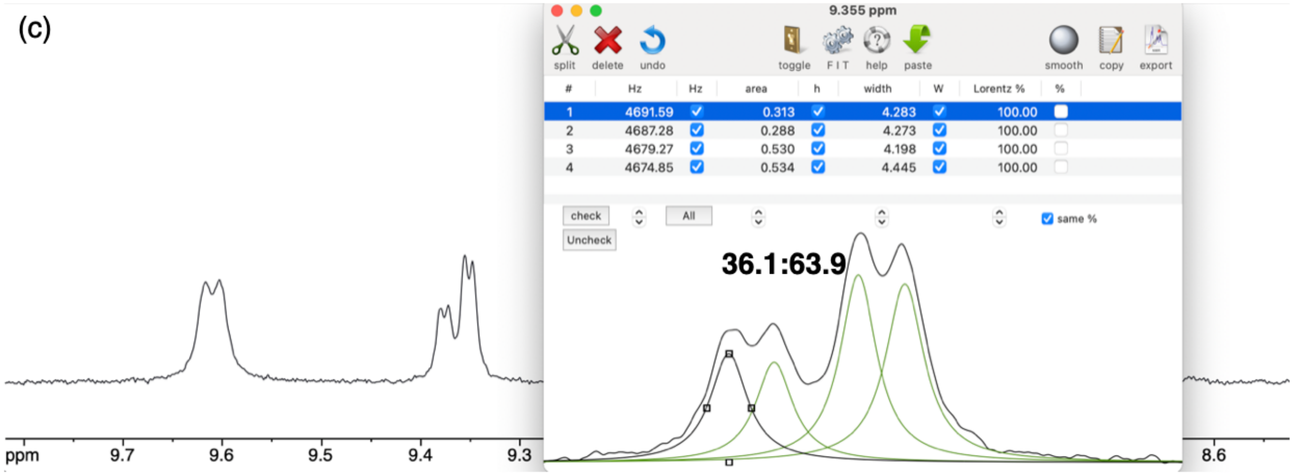
9.355 ppm
split delete undo toggle FIT help paste smooth copy export
Hz Hz area h width W Lorentz % %
1 4691.59 0.313 4.283 100.00
2 4687.28 0.288 4.273 100.00
3 4679.27 0.530 4.198 100.00
4 4674.85 0.534 4.445 100.00
check All same %
Uncheck
36.1:63.9
ppm 9.7 9.6 9.5 9.4 9.3 8.6

(d)

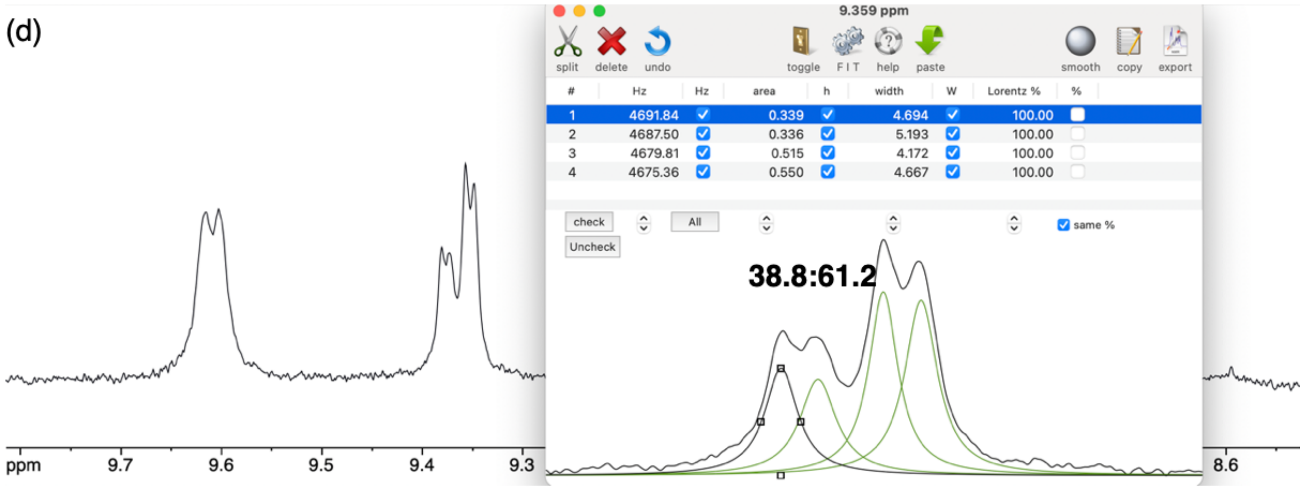
9.359 ppm
split delete undo toggle FIT help paste smooth copy export
Hz Hz area h width W Lorentz % %
1 4691.84 0.339 4.694 100.00
2 4687.50 0.336 5.193 100.00
3 4679.81 0.515 4.172 100.00
4 4675.36 0.550 4.667 100.00
check All same %
Uncheck
38.8:61.2
ppm 9.7 9.6 9.5 9.4 9.3 8.6

(e)

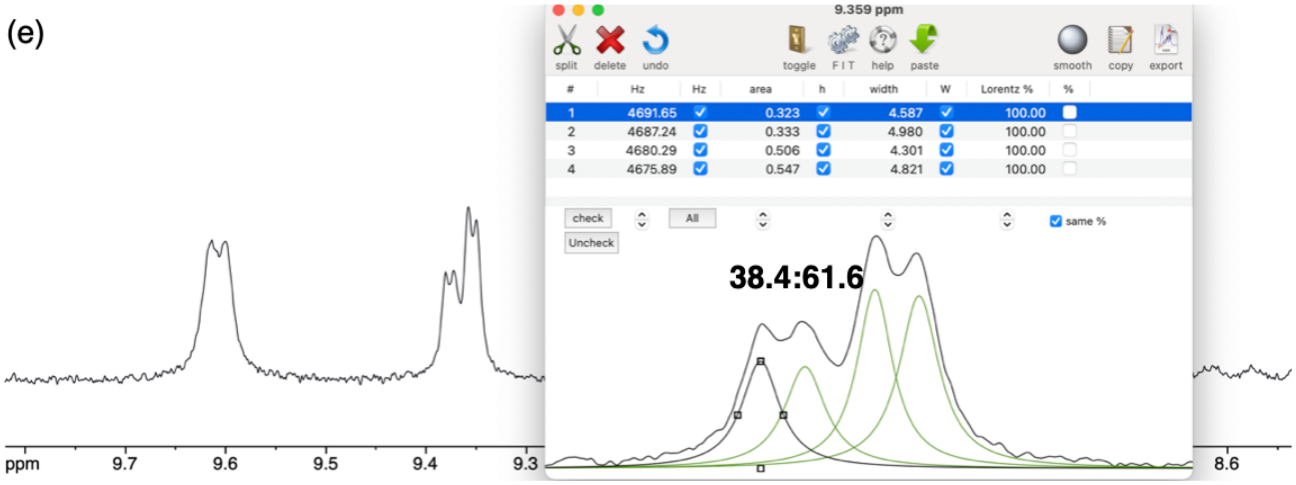

Supplementary Fig. 117 Deconvolution analysis of ^{1}H NMR spectra of the (*M*)-enantio-enriched $\mathbf{1}_{\mathbf{tight}}$ with Δ-**4**; (a) 20 min after addition of Δ-**4**, (b) 1 day, (c) 3 days, (d) 7 days and (e) 14 days, shown in Supplementary Fig. 116.

8. Computational study

DFT calculation

All density functional theory calculations were performed using Gaussian 16 [rev.C01] and [rev.C02] program[16]. The m06-D3 functional[17,18] and mixed basis sets of def2svp[19] for Pd and 6-31G(d)[20,21] for other atoms were used for calculation of (*P*)-$\mathbf{1}_{\mathbf{tight}}$ and (*P*)-$\mathbf{1}_{\mathbf{loose}}$. No imaginary frequency was found for all optimised structures, confirming that each optimised structure reached a local minimum. The result of TD-DFT calculation was convoluted using GaussSum software to create simulated UV-vis and CD spectra[22]. To check the dependence of the simulated CD spectra of (*P*)-$\mathbf{1}_{\mathbf{tight}}$ on the functional and the basis sets, the B3LYP-D3[23,24], TPSSh[25,26] and wB97[27] functionals and the 6-31+G(d)[28] basis set were used.

NBO calculation

Natural bond orbital (NBO) calculations of the optimised structure were performed using NBO7.0 package[29] embedded in the Gaussian16 software. The NBOs were visualised with the Avogadro1.2 program[30].

NCI plot calculation

Noncovalent interaction (NCI) plot calculations were performed using NCIPLOT4 program[31,32]. The NCI plots were visualised with VMD1.9 software[33].

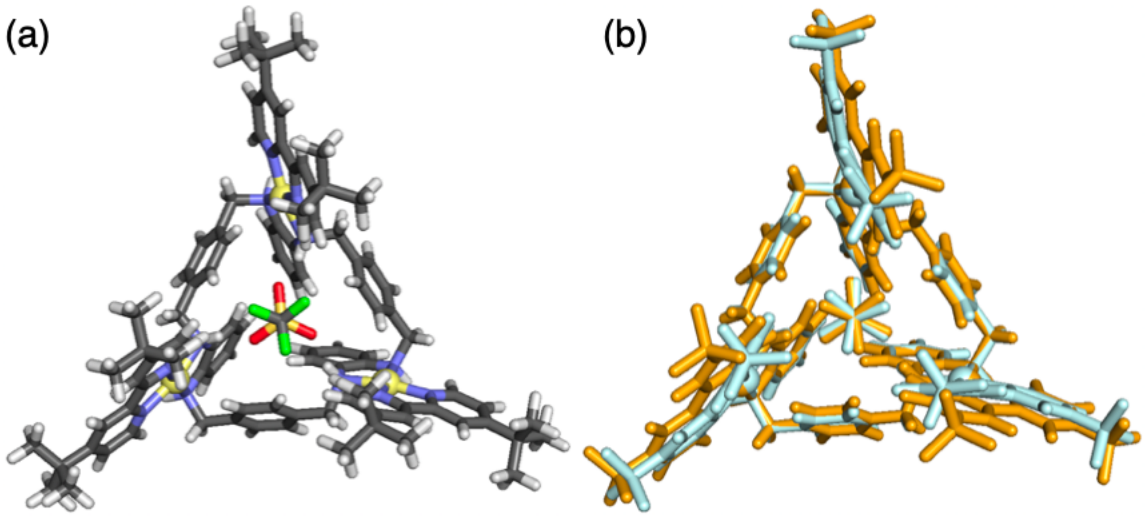

Supplementary Fig. 118 (a) Optimised structure of (*P*)-$\mathbf{1}_{\mathbf{tight}}$ with a triflate anion inside of the macrocycle and (b) overlaid image of (blue) the crystal and (orange) the optimised structure; protons of *tert*-butyl groups are omitted for clarity.

		Pd-N1	Pd-N2	Pd-N3	Pd-N4	N1-O	Pd-HC	Pd-Pd’
Crystal structure		2.055	2.063	2.029	2.040	2.785	2.736	9.000
m06-D3	def2svp for Pd<br>6-31G* for others	2.094	2.088	2.060	2.042	2.810	2.561	9.290
	def2svp for Pd<br>6-31+G* for O, F<br>6-31G* for others	2.100	2.087	2.058	2.046	2.835	2.577	9.334
B3LYP-D3	def2svp for Pd<br>6-31G* for others	2.099	2.092	2.057	2.042	2.821	2.647	9.330
TPSSh	def2svp for Pd<br>6-31G* for others	2.112	2.075	2.052	2.039	2.900	2.749	9.496
wB97	def2svp for Pd<br>6-31G* for others	2.093	2.080	2.053	2.040	2.847	2.605	9.372

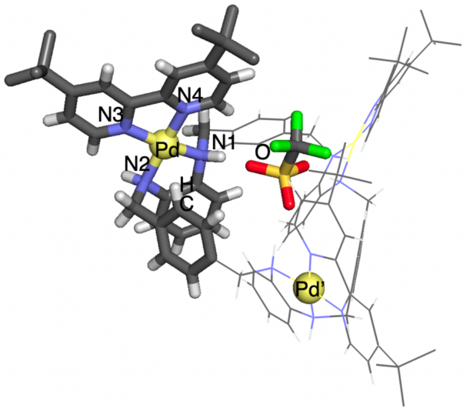

Supplementary Fig. 119 (left) Table of the distances in crystal and DFT-optimised structures between a Pd atom and N atoms, the N1 atom and the O atom forming hydrogen bonds, the Pd atom and the H atom forming C-H···Pd interaction and two Pd atoms. (right) a structure of $\mathbf{1}_{\mathbf{tight}}$ indicating the atom labels.

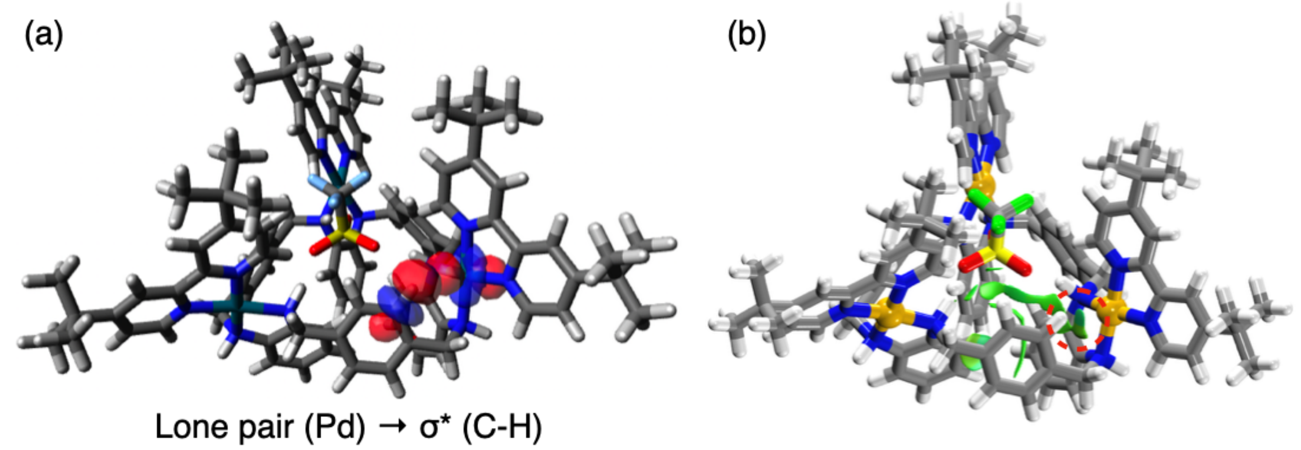

Supplementary Fig. 120 (a) Superposition of a donor (lone pair (Pd)) and an acceptor (σ* (C-H)) NBOs (isovalue = 0.04) of (*P*)-$\mathbf{1}_{\mathbf{tight}}$ with a stabilising energy of 1.07 kcal/mol. (b) NCI plot of (*P*)-$\mathbf{1}_{\mathbf{tight}}$ (the isosurface is generated for σ = 0.3 au and –0.06 < r < 0.06 au); the interactions between one *para*-phenylene ring and other moiety were performed for clarity.

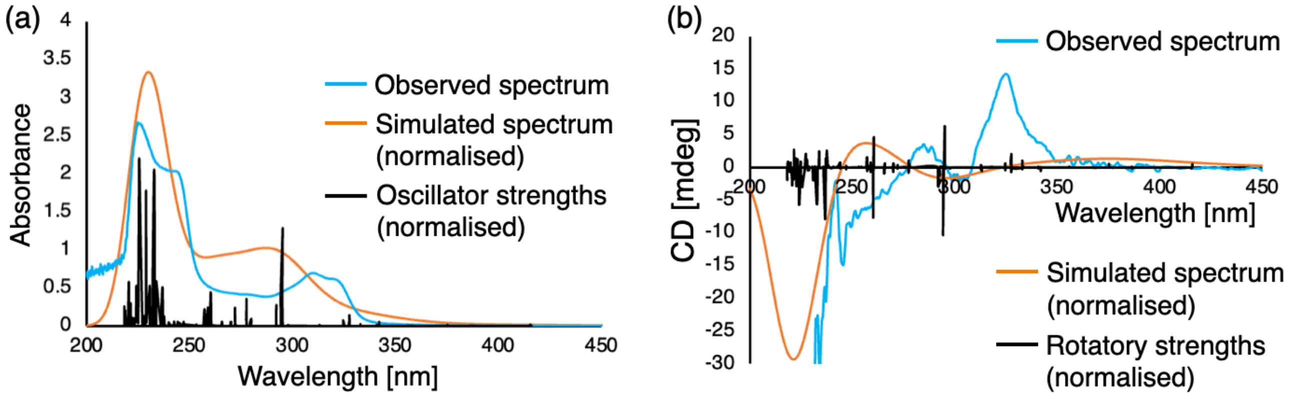

Supplementary Fig. 121 (a) UV-vis spectra and (b) CD spectra; (blue line) observed spectra (CH_2Cl_2, 293 K, l = 0.2 cm for UV-vis spectrum and 1.0 cm for CD spectrum) of synthesised (*P*)-enantio-enriched $\mathbf{1}_{\mathbf{tight}}$, (orange line) simulated spectra of DFT-optimised (*P*)-$\mathbf{1}_{\mathbf{tight}}$ [m06-D3/def2svp for Pd/6-31G(d) for others] and (black line) oscillator strengths in UV-vis spectrum and rotatory strengths in CD spectrum simulated by TD-DFT calculation.

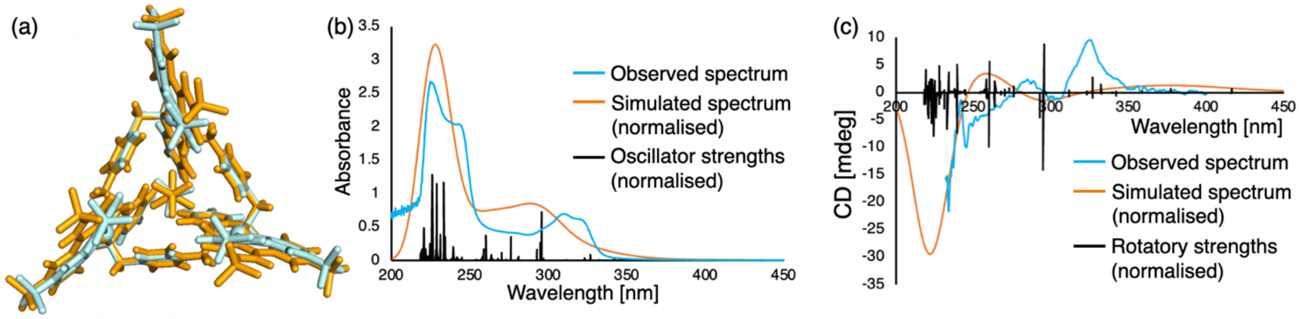

Supplementary Fig. 122 (a) Overlaid image of (blue) the crystal and (orange) the optimised structure of (*P*)-$\mathbf{1}_{\mathbf{tight}}$ [m06-D3/def2svp for Pd/6-31+G(d) for O, F/6-31G(d) for others]; protons of *tert*-butyl groups are omitted for clarity. (b) UV-vis spectra and (c) CD spectra; (blue line) observed spectra (CH_2Cl_2, 293 K, l = 0.2 cm for UV-vis spectrum and 1.0 cm for CD spectrum) of synthesised (*P*)-enantio-enriched $\mathbf{1}_{\mathbf{tight}}$, (orange line) simulated spectra of DFT-optimised (*P*)-$\mathbf{1}_{\mathbf{tight}}$ [m06-D3/def2svp for Pd/6-31+G(d) for O, F/6-31G(d) for others] and (black line) oscillator strengths in UV-vis spectrum and simulated rotatory strengths in CD spectrum simulated by TD-DFT calculation.

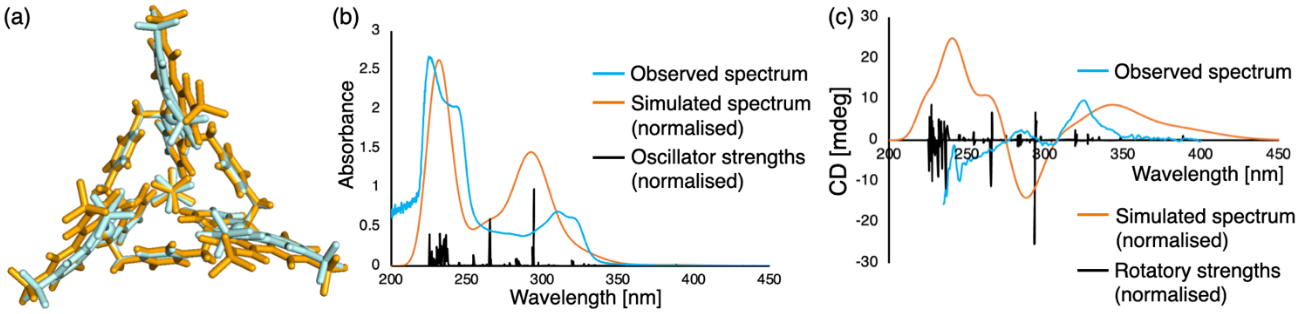

Supplementary Fig. 123 (a) Overlaid image of (blue) the crystal and (orange) the optimised structure of (*P*)-$\mathbf{1}_{\mathbf{tight}}$ [B3LYP-D3/def2svp for Pd/6-31G(d) for others]; protons of *tert*-butyl groups are omitted for clarity. (b) UV-vis spectra and (c) CD spectra; (blue line) observed spectra (CH_2Cl_2, 293 K, l = 0.2 cm for UV-vis spectrum and 1.0 cm for CD spectrum) of synthesised (*P*)-enantio-enriched $\mathbf{1}_{\mathbf{tight}}$, (orange line) simulated spectra of DFT-optimised (*P*)-$\mathbf{1}_{\mathbf{tight}}$ [B3LYP-D3/def2svp for Pd/6-31G(d) for others] and (black line) oscillator strengths in UV-vis spectrum and simulated rotatory strengths in CD spectrum simulated by TD-DFT calculation.

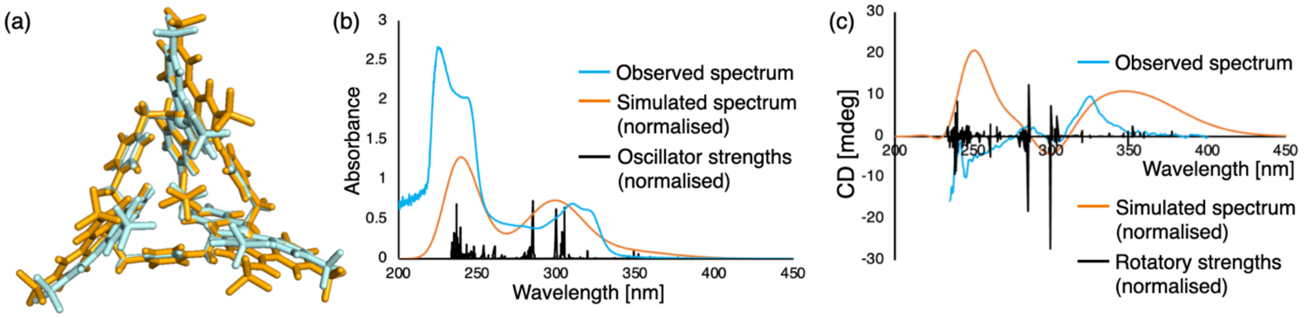

Supplementary Fig. 124 (a) Overlaid image of (blue) the crystal and (orange) the optimised structure of (*P*)-$\mathbf{1}_{\mathbf{tight}}$ [TPSSh/def2svp for Pd/6-31G(d) for others]; protons of *tert*-butyl groups are omitted for clarity. (b) UV-vis spectra and (c) CD spectra; (blue line) observed spectra (CH_2Cl_2, 293 K, l = 0.2 cm for UV-vis spectrum and 1.0 cm for CD spectrum) of synthesised (*P*)-enantio-enriched $\mathbf{1}_{\mathbf{tight}}$, (orange line) simulated spectra of DFT-optimised (*P*)-$\mathbf{1}_{\mathbf{tight}}$ [TPSSh/def2svp for Pd/6-31G(d) for others] and (black line) oscillator strengths in UV-vis spectrum and simulated rotatory strengths in CD spectrum simulated by TD-DFT calculation.

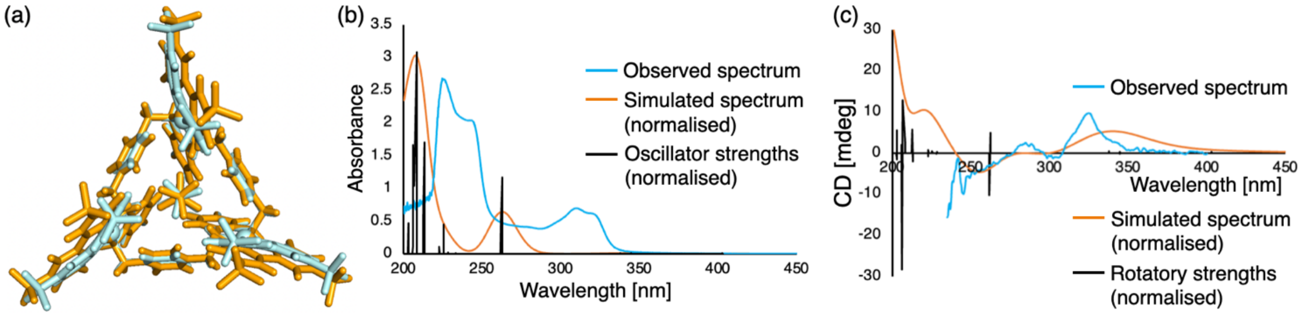

Supplementary Fig. 125 (a) Overlaid image of (blue) the crystal and (orange) the optimised structure of (*P*)-$\mathbf{1}_{\mathbf{tight}}$ [wB97/def2svp for Pd/6-31G(d) for others]; protons of *tert*-butyl groups are omitted for clarity. (b) UV-vis spectra and (c) CD spectra; (blue line) observed spectra (CH_2Cl_2, 293 K, l = 0.2 cm for UV-vis spectrum and 1.0 cm for CD spectrum) of synthesised (*P*)-enantio-enriched $\mathbf{1}_{\mathbf{tight}}$, (orange line) simulated spectra of DFT-optimised (*P*)-$\mathbf{1}_{\mathbf{tight}}$ [wB97/def2svp for Pd/6-31G(d) for others] and (black line) oscillator strengths in UV-vis spectrum and simulated rotatory strengths in CD spectrum simulated by TD-DFT calculation.

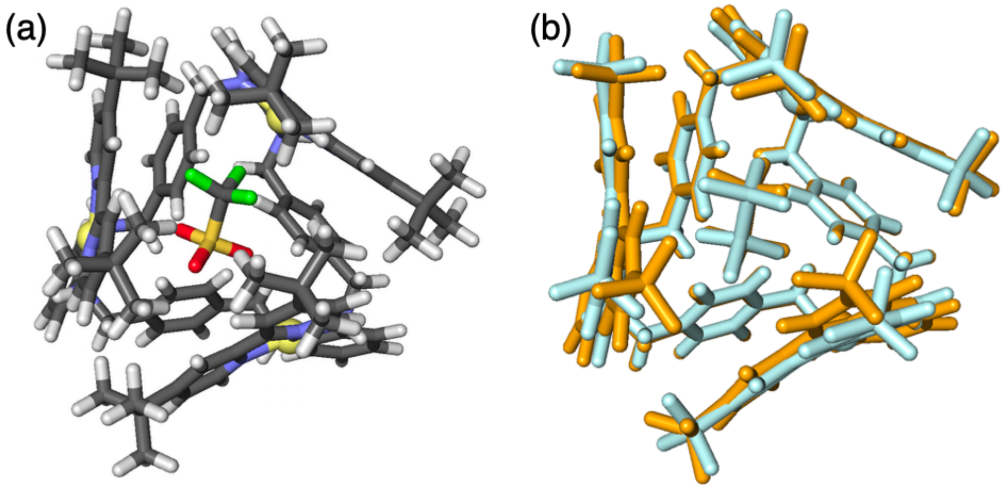

Supplementary Fig. 126 (a) Optimised structure of (*P*)-**1**$_{\mathbf{loose}}$ with a triflate anion inside of the macrocycle and (b) overlaid image of (blue) the crystal and (orange) the optimised structure; protons of *tert*-butyl groups are omitted for clarity.

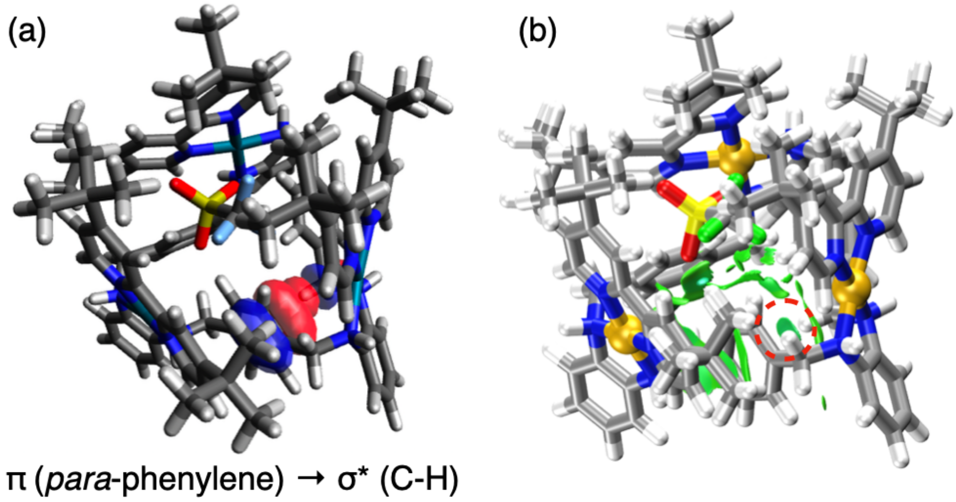

Supplementary Fig. 127 (a) Superposition of a donor (π (*para*-phenylene)) and an acceptor (σ* (C-H)) NBOs (isovalue = 0.05) of (*P*)-**1**$_{\mathbf{loose}}$ with a stabilising energy of 1.26-1.88 kcal/mol. (b) NCI plot of (*P*)-**1**$_{\mathbf{loose}}$ (the isosurface is generated for σ = 0.25 au and –0.07 < r < 0.07 au); the interactions between one *para*-phenylene ring and other moiety were performed for clarity.

9. References

1. Hayashi, R., Tashiro, S., Asakura, M., Mitsui, S. & Shionoya, M. Effector-dependent structural transformation of a crystalline framework with allosteric effects on molecular recognition ability. *Nat. Commun.* **14**, 4490 (2023).
2. Dolomanov, O.V., Bourhis, L. J., Gildea, R. J., Howard, J. A. K. & Puschmann, H. *OLEX2*: a complete structure solution, refinement and analysis program. *J. Appl. Cryst.* **42**, 339–341 (2009).
3. Sheldrick, G. M. *SHELXL-97: Program for refinement of crystal structure.* University of Göttingen, Göttingen, Germany (1997); Sheldrick, G. M. *SHELXL-2013*. University of Göttingen, Göttingen, Germany (2013).
4. Spek, A. L. *PLATON: A multipurpose crystallographic tool.* Utrecht University, Utrecht, The Netherlands (2001).
5. Qin, Z., Jennings, M. C. & Puddephatt, R. J. Molecular triangle of palladium(II) and its anion binding properties. *Inorg. Chem.* **41**, 3967–3974 (2002).
6. Singh, A., Anandhi, U., Cinellu, M. A. & Sharp, P. R. Diimine supported group 10 hydroxo, oxo, amido, and imido complexes. *Dalton Trans*. 2314-2327 (2008).
7. Zhong, F. & Zhao, J. An N^N Platinum(II) bis(acetylide) complex with naphthalimide and pyrene ligands: synthesis, photophysical properties, and application in triplet-triplet annihilation upconversion. *Eur. J. Inorg Chem.* 5196–5204 (2017).
8. McBee, J. L., Bell, A. T. & Tilley, T. D. Mechanistic studies of the hydroamination of norbornene with electrophilic platinum complexes: the role of proton transfer. *J. Am. Chem. Soc.* **130**, 16562–16571 (2008).
9. Perrin, C. L. & Dwyer, T. J. Application of two-dimensional NMR to kinetics of chemical exchange. *Chem. Rev.* **90**, 935–967 (1990).
10. StatPlus:mac, AnalystSoft Inc. - statistical analysis program for macOS. Version v8.
11. Youn, J.-H. & Herrmann, R. A simple and efficient preparation of sulfinyl chlorides from disulfides and sulfuryl chloride. *Tetrahedron Lett.* **27**, 1493–1494 (1986).
12. Fernández, I., Khiar, N., Llera, J. M. & Alcudia, F. Asymmetric synthesis of alkane- and arenesulfinates of diacetone-D-glucose (DAG): an improved and general route to both enantiomerically pure sulfoxides. *J. Org. Chem.* **57**, 6789–6796 (1992).
13. Ruano, J. L. G., Alemparte, C., Aranda, M. T. & Zarzuelo, M. M. A general and expeditious one-pot synthesis of sulfoxides in high optical purity from norephedrine-derived sulfamidites. *Org. Lett.* **5**, 75–78 (2003).
14. Brunel, J.-M., Diter, P., Duetsch, M. & Kagan, H. B. Highly enantioselective oxidation of sulfides mediated by a chiral titanium complex. *J. Org. Chem.* **60**, 8086–8088 (1995).
15. Mislow, K., Simmons, T., Melillo, J. T. & Ternay, Jr., A. L. The hydrogen chloride-catalyzed racemization of sulfoxides. *J. Am. Chem. Soc.* **86**, 1452–1453 (1964).
16. Gaussian 16, Revision C.01, Frisch, M. J., Trucks, G. W., Schlegel, H. B., Scuseria, G. E., Robb, M. A., Cheeseman, J. R., Scalmani, G., Barone, V., Petersson, G. A., Nakatsuji, H., Li, X., Caricato, M., Marenich, A. V., Bloino, J., Janesko, B. G., Gomperts, R., Mennucci, B., Hratchian, H. P., Ortiz, J. V., Izmaylov, A. F., Sonnenberg, J. L., Williams-Young, D., Ding, F., Lipparini, F., Egidi, F.,

Goings, J., Peng, B., Petrone, A., Henderson, T., Ranasinghe, D., Zakrzewski, V. G., Gao, J., Rega, N., Zheng, G., Liang, W., Hada, M., Ehara, M., Toyota, K., Fukuda, R., Hasegawa, J., Ishida, M., Nakajima, T., Honda, Y., Kitao, O., Nakai, H., Vreven, T., Throssell, K., Montgomery, Jr. J. A., Peralta, J. E., Ogliaro, F., Bearpark, M. J., Heyd, J. J., Brothers, E. N., Kudin, K. N., Staroverov, V. N., Keith, T. A., Kobayashi, R., Normand, J., Raghavachari, K., Rendell, A. P., Burant, J. C., Iyengar, S. S., Tomasi, J., Cossi, M., Millam, J. M., Klene, M., Adamo, C., Cammi, R., Ochterski, J. W., Martin, R. L., Morokuma, K., Farkas, O., Foresman, J. B. & Fox, D. J. Gaussian, Inc., Wallingford CT (2019).

17. Zhao, Y. & Truhlar, D. G. The M06 suite of density functionals for main group thermochemistry, thermochemical kinetics, noncovalent interactions, excited states, and transition elements: two new functionals and systematic testing of four M06-class functionals and 12 other functionals. *Theor. Chem. Acc*. **120**, 215–241 (2008).
18. Grimme, S., Antony, J., Ehrlich, S. & Krieg, H. A consistent and accurate *ab initio* parametrization of density functional dispersion correction (DFT-D) for the 94 elements H-Pu. *J. Chem. Phys.* **132**, 154104 (2010).
19. Weigend, F. & Ahlrichs, R. Balanced basis sets of split valence, triple zeta valence and quadruple zeta valence quality for H to Rn: design and assessment of accuracy. *Phys. Chem. Chem. Phys.* **7**, 3297–3305 (2005).
20. Hehre, W. J., Ditchfield, R. & Pople, J. A. Self-consistent molecular orbital methods. XII. Further extensions of Gaussian-type basis sets for use in molecular orbital studies of organic molecules. *J. Chem. Phys.* **56**, 2257–2261 (1972).
21. Hariharan, P. C. & Pople, J. A. The influence of polarization functions on molecular orbital hydrogenation energies. *Theoret. Chim. Acta* **28**, 213–222 (1973).
22. O’Boyle, N. M., Tenderholt, A. L. & Langner, K. M. Cclib: a library for package-independent computational chemistry algorisms. *J. Comp. Chem.* **29**, 839–845 (2008).
23. Becke, A. D. Density-functional thermochemistry. III. The role of exact exchange. *J. Chem. Phys.* **98**, 5648–5652 (1993).
24. Stephens, P. J., Devlin, F. J., Chabalowski, C. F. & Frisch, M. J. *Ab initio* calculation of vibrational absorption and circular dichroism spectra using density functional force field. *J. Chem. Phys.* **98**, 11623–11627 (1994).
25. Tao, J., Perdew J. P., Staroverov, V. N. & Scuseria, G. E. Climbing the density functional ladder: nonempirical meta-generalized gradient approximation designed for molecules and solids. *Phys. Rev. Lett.* **91**, 146401 (2003).
26. Staroverov, V. N., Scuseria, G. E., Tao, J. & Perdew J. P. Comparative assessment of a nonempirical density functional: molecules and hydrogen-bonded complexes. *J. Chem. Phys.* **119**, 12129–12137 (2003).
27. Chai, J.-D. & Head-Gordon M. Systematic optimization of long-range corrected hybrid density functionals. *J. Chem. Phys.* **128**, 084106 (2008).

28. Clark, T., Chandrasekhar, J. Spitznagel, G. W. & Schleyer, P. v. R. Efficient diffuse function-augmented basis sets for anion calculations. III.* The 3-21+G basis set for first-row elements, Li-F. *J. Comput. Chem.* **4**, 294–301 (1983).
29. NBO 7.0. Glendening, E. D., Badenhoop, J. K., Reed, A. E., Carpenter, J. E., Bohmann, J. A., Morales, C. M., Karafiloglou, P., Landis, C. R., & Weinhold, F. Theoretical Chemistry Institute, University of Wisconsin, Madison, WI (2018).
30. Hanwell, M. D., Curtis, D. E., Lonie, D. C., Vandermeersch, T., Zurek, E. & Hutchison, G. R. Avogadro: an advanced semantic chemical editor, visualization, and analysis platform. *Journal of Cheminformatics*, **4**, 17 (2012).
31. Johnson, E. R., Keinan, S., Mori-Sánchez, P., Contreras-García, J., Cohen, A. J. & Yang, W. Revealing noncovalent interactions. *J. Am. Chem. Soc.* **132**, 6498–6506 (2010).
32. Boto, R. A., Peccati, F., Laplaza, R., Quan, C., Carbone, A., Piquemal, J.-P., Maday, Y. & Contreras-García, J. NCIPLOT4 : fast, robust, and quantitative analysis of noncovalent interactions. *J. Chem. Theory Comput.* **16**, 4150–4158 (2020).
33. Humphrey, W., Dalke, A. & Schulten, K. VMD: visual molecular dynamics. *J. Molec. Graphics* **14**, 33–38 (1996).
